# On the variation of the belt and chiral screw and spring conformations of substituted regioregular HT undecathiophenes†

**DOI:** 10.1039/c7ra12777d

**Published:** 2018-01-09

**Authors:** Jan Cz. Dobrowolski, Małgorzata E. Jamróz

**Affiliations:** Institute of Chemistry and Nuclear Technology 16 Dorodna Street 03-195 Warsaw Poland j.dobrowolski@nil.gov.pl +48 22 811 1917 +48 22 504 1086

## Abstract

The stability of a belt- and two chiral screw- and spring-conformers of HT regioregular substituted undecathiophenes was calculated. For many substituents these conformers can possibly coexist. The averaged SCCS torsion angle and HOMA index characterized well the geometry and π-electron delocalization. The substituent volume appeared to shape the conformer form and stability.

## Abstract 2

The stability and characteristics of shape and π-electron delocalization of a belt- and two chiral screw- and spring-conformers of HT regioregular substituted undecathiophenes was calculated.

## Graphical abstract

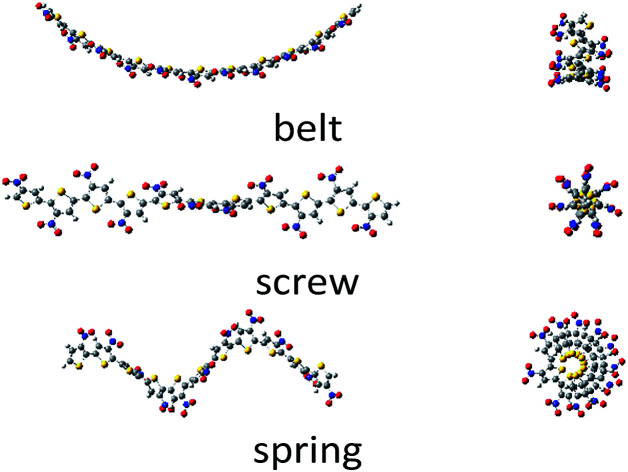

Polythiophenes were among the first studied conjugated polymers and they are still in the group of the most highly investigated polymers for applications in organic electronics.^1^ Indeed, they are efficient in charge transport, can be produced at low cost, and are compatible with flexible substrates.^2,3^ Among diverse polythiophenes, the chiral ones have been studied since 1988.^4^ Combination of chirality with conductivity can be used in electrochemical chiral sensing, asymmetric synthesis, enantioselective membranes or chiral micro- and nano-dimensional fibres useful in the design and development of specific molecular devices.^4,5^

Chirality of polythiophenes can originate from four main, independent, factors: (1) constitutional, (2) conformational, (3) aggregational, and (4) external. The constitutional arises from chirality elements^6^ present in substituted thiophene monomers and building blocs incorporated into the oligothiophene chain. The polythiophene conformational chirality is connected to the chain,^7^ which in limit cases can form a helix twisted along the backbone as in a screw or a helix twisted as in a spring. The polythiophene screws or springs may occur in all-transoid or in all-cisoid structures, respectively.^7^ The aggregational chirality can be driven by polythiophene interchain stacking interactions which, under several circumstances, can lead to chains aggregation in a helix form which manifests itself by a significant increase of circular dichroism signals.^8^ Formation of polythiophene aggregates can also be a result of system ordering by a liquid–crystalline phase promoting substituent^9^ or interactions with liquid crystal solvents.^10^ Finally, an external chiral molecule or an ion can induce both conformational and aggregational changes of polythiophenes.^11,12^

The structural insight into the polymers in thin layers is difficult. Yet, already in 1985 a “coil structure” of 1 : 2 anion doped poly-3-methyl-thiophene was proposed based on electron diffraction and X-ray data despite the polymer was crystallized in only about 5%.^13^ In 1989 and 1990, the helical structures of doped polypyrroles and polythiophenes were evidenced using scanning tunnelling microscopy.^14–16^

In early studies, researchers often concentrated on deducing chiral polythiophene features from presence of chiral substituents. Then, the focus was rather shifted towards conformational and aggregational chirality. Now, it seems that the main attention is turned towards designing the polythiophene chirality by influencing the way of aggregation through variation of temperature, solvent, pH, presence of other (macro)molecules including liquid–crystal solvents.

The semiempirical MNDO calculations of “rod” and “coil” conformers of polythiophene, polypyrrole and their 3-methyl derivatives performed in late 1980s supported early experimental findings on relative stability of the helical forms.^17^

In mid 1990s, studies of planar all-*trans* and helical all-*cis* polythiophenes were performed using kind of special molecular mechanics force fields dedicated to crystal structure calculations, and confirmed that also in crystal lattices the former conformation is more stable than the latter and that the helix characteristics visibly depend on dopant added.^18,19^ Interestingly, it was argued that anions like BF_4_^−^ revealed an almost constant energy path through the channel formed inside the helix.^19^ Transport of the same anion in helical poly(3-octyl thiophene) simulated using molecular dynamics approach, indicated that the energy barrier for the ion movement was lower than 2 kcal mol^−1^.^20^ The rate of the transport was predicted to increase with oxidation but after oxidation of 50% it became hindered.

The band gaps of undoped all-*trans* and helical all-*cis* polythiophene, polyfurane and polypyrrole, calculated at carefully selected B3LYP/6-31G* level,§The computations were performed using the B3LYP functional^39,40^ combined with the 6-31G** basis set^41,42^ as implemented in Gaussian 09 suite of programs.^43^ Correlations were done using SigmaPlot program.^44^were estimated to be as in semiconductors. In all conformations, they exhibited excellent π-electron delocalization and dense valence and conduction bands.^21^ Moreover, it was demonstrated that doping with both electron donor or acceptor species reduced the band gaps and pushed behaviour of the systems towards those typical for metals.^21^

Oligothiophenes, oligofurans and oligopyrroles with vinylene or azomethine ring linkers have been recently studied at the B3LYP/6-31G** level combined with and without Grimme's D3 correction.^22^ Strikingly, the relative stability of the conformers and difference in electronic transitions depended on the linker, but for the azomethine linker also on the heterocycle unit.^22^ Quite recently motions of ions in the channels in 100-ring polythiophene helical structures in aqueous solution have been studied by using molecular dynamics.^23^

Despite all those studies, the energetic and structural differences between different polythiophene conformations have never been widely studied. Here, belt, screw and spring conformations (Fig. 1) of regioregular HT (head-to-tail) undecathiophenes^24^ substituted with 26 substituents, covering relatively full range of substituent effects on both σ- and π-electron system of the thiophene valence electrons, were examined by using computational chemistry methods. Such an approach enables consideration of a wide range of interesting substituents which would be very difficult to examine experimentally.

**Fig. 1:**
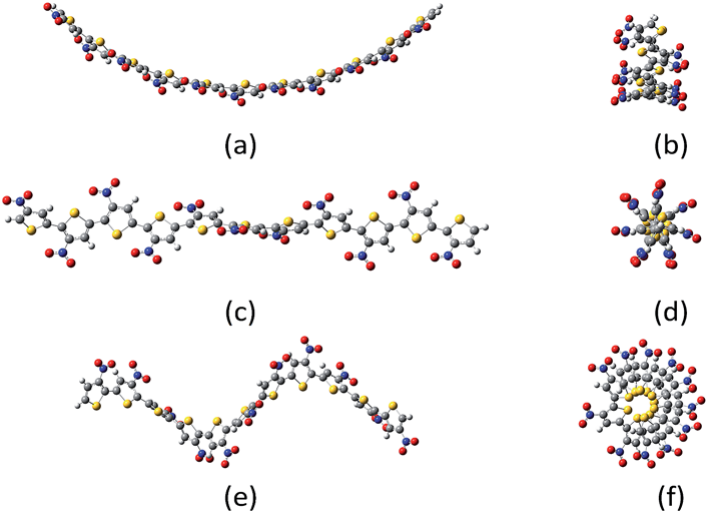
Example of belt (a, b); screw (c, d) and spring (e, f) conformations of undecanitro undecathiophenes optimized at the B3LYP/D3/6-31G** level.§

The undecathiophenes chain has relatively large degree of freedom. Indeed, even if only transoid and cisoid positions of neighbouring rings would be considered then 2^10^ different conformations could be possible. Moreover, for already adopted *cis*–*trans* conformation, the whole “belt” is flexible and undulate (Fig. 2). The more monomers in an oligomer the more waves is possible. For the planar undecathiophene as much as five imaginary frequencies is predicted at the B3LYP/6-31G** level. Hence, the planar undecathiophene structure is not a local minimum at the potential energy surface, and a structure with several extrema can be the most stable belt conformation. However, a conclusive answer to the question “how many waves has the global minimum?” cannot be easily done first and foremost because energy difference between conformers such as C- and S-shaped structures is within the confidence level of DFT calculations regardless the basis set and the method (Table 1, ESI†). Since here we cannot overcome these and many other methodological constrains, from now on, we consider only the C-shaped structures because, out of belt conformers, they can be the most uniformly defined. Still, an uncertainty related to the conformational freedom of the substituents remains. However, addressing this issue in the studied systems goes far beyond the aim of this communication.

**Fig. 2:**
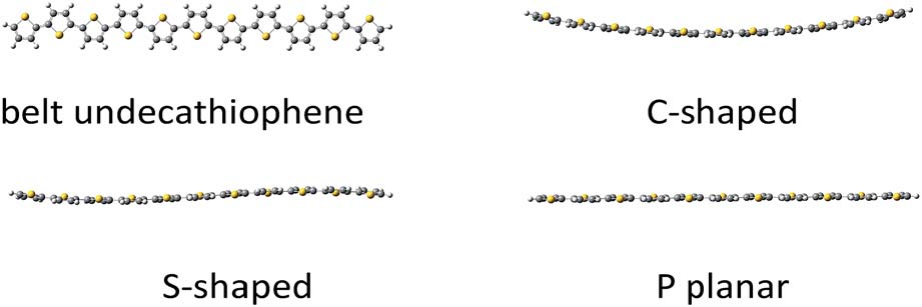
Types of the belt conformations of unsubstituted undecathiophenes: C-shaped, S-shaped, and P planar exhibiting several imaginary frequencies in calculations.

The screw conformations (Fig. 1) seem to be very strained. Thus, it was very surprising that for quite a number of systems, the starting belt structure converged either to the C-shaped belts or to the screws depend on how the substituents were initially, but only slightly, distorted. Nevertheless, for some substituents the screw conformation converged to the belt one and for some others, to obtain the screw conformation, it was necessary to start computations from already screwed structure.

It turned out that the Gibbs free energy difference between the screw and C-shaped belt forms is not that big (Tables 1–4, ESI†). In fact, for the studied systems the difference is below 2 kcal mol^−1^. Even more, for some substituents (COOH, *i*Pr, Li, *n*Bu, NO_2_, Ph, SH, SMe and *t*Bu) the screw conformations are predicted to be more stable than the C-shaped belts (Table 1). It is clear that Li is a purely hypothetical substituent and *t*Bu can hardly be introduced in such the HT regioregular way. Yet, for *i*Pr, SH and SMe, this is not so sure. For several substituents (Br, CCH, Cl, CN, F, H, NMe_2_, OH and OMe) the introductory screw conformations converged to the C-shaped belt ones.

**Table 1:** The Gibbs free energy differences Δ*G* (kcal mol^−1^) between screw (scr) and spring (spr) *vs.* C-shaped belt conformations of substituted undecathiophenes, and mean of modules of the *τ*(SCCS) torsion angles (deg) and the HOMA geometric aromaticity indices over the conjugated π-electron structure in the three types of conformations^a^

X=	Δ*G*		|*τ*_av_(SCCS)|			HOMA		
	scr	spr	Belt	scr	spr	Belt	scr	spr
BF_2_	1.4	2.1	137.1	137.1	30.6	0.688	0.671	0.694
BH_2_	1.3	3.2	144.8	145.5	32.1	0.704	0.691	0.693
Br	→ b	11.5	179.3	→ b	25.0	0.808	→ b	0.776
CBr_3_	1.3	−3.1	127.6	125.4	34.3	0.661	0.639	0.673
CCH	→ b	3.2	180.0	→ b	17.3	0.789	→ b	0.770
CCl_3_	0.3	1.8	122.2	119.7	36.3	0.639	0.619	0.617
CF_3_	0.5	0.3	143.0	141.5	29.9	0.742	0.724	0.743
CH_3_	→ b	5.5	161.4	→ b	31.2	0.791	→ b	0.732
CHO	0.3	−8.9	140.0	169.6	6.4	0.715	0.774	0.785
Cl	→ b	6.9	176.6	→ b	27.4	0.809	→ b	0.769
CN	→ b	3.5	177.3	→ b	18.0	0.795	→ b	0.782
COOH	−10.1	−6.0	130.3	156.2	17.5	0.671	0.733	0.721
F	→ b	2.4	180.0	→ b	9.3	0.845	→ b	0.850
H	→ b	10.2	166.3	→ b	24.9	0.810	→ b	0.792
*i*Pr	−0.7	3.7	138.8	136.8	34.8	0.693	0.674	0.668
Li	−1.5	4.3	151.6	155.9	37.7	0.705	0.684	0.613
*n*Bu	−5.9	−2.6	151.5	135.6	35.4	0.758	0.669	0.662
NH_2_	2.1	4.9	157.5	152.0	30.7	0.819	0.783	0.784
NMe_2_	→ b	20.1	177.3	177.4	22.1	0.792	0.790	0.781
NO_2_	−0.7	13.7	154.2	152.6	30.8	0.763	0.755	0.718
OH	0.1	5.9	150.9	153.1	7.4	0.804	0.798	0.853
OMe	→ b	6.1	180.0	→ b	6.4	0.865	→ b	0.857
Ph	−0.9	2.2	136.6	136.5	32.7	0.697	0.685	0.693
SH	−1.0	3.1	161.3	159.8	31.0	0.787	0.781	0.750
SiH_3_	0.2	2.2	145.3	145.3	33.2	0.727	0.708	0.697
SMe	−1.5	2.9	167.9	165.2	30.1	0.781	0.774	0.731
*t*Bu	−0.5	−2.2	108.7	107.6	38.9	0.551	0.543	0.520

a → b – the starting screw structure converges to the belt conformer. The data in italic correspond to not fully optimized forms.

Comparison of energetics of the C-shaped belt and screw conformers of eleven-substituted undecathiophenes indicates that for some substituents probably: (i) the screw structures can be formed, (ii) the screw and belt forms can co-exist, and maybe rarely, (iii) the screw conformers may be dominant. However, one should remember that the level of theory used here allows only for semi-quantitative considerations as the accuracy of the performed DFT predictions is about a few kcal mol^−1^.^25,26^

If our predictions were inferred based only on behaviour of the unsubstituted undecathiophenes, for which the spring form is *ca.* 10 kcal mol^−1^ less stable than the belt one, then we would think that existence of spring conformers is highly improbable.

However, for quite a few substituents the energetic distance between the belt and spring forms is below 5 kcal mol^−1^ (Table 1) and formation of springs cannot be fully ignored. Surprisingly, among substituents stabilizing the spring structures there are some (CF_3_, CCl_3_, CBr_3_, CHO, COOH, *n*Bu and *t*Bu) for which this very conformation is predicted to be either more or only a bit less stable than the belt one (Table 1).

Even if we skip considering *t*Bu for the reasons given above, one should not disregard stabilization of the springs produced by trihalomethanes and the CHO and COOH groups. Thus, it seems not impossible that the substituted undecathiophenes: (i) exist in the spring conformations, (ii) may co-exist with the screw and even the belt conformations (CF_3_, F, *n*Bu and COOH), and also one cannot exclude that for some substituents (iii) the spring conformers may be dominant.

At the end of this section let us remind that in nature interaction with a liquid crystalline or a chiral solvent can be the driving force for a helix formation. This process can be supported by the adequate temperature, solubility, (chiral) additives *etc.* So, the energetics presented above indicates that apparently strange screw and spring forms must not always be very improbable.

Now, let us consider geometrical characteristics of the studied structures. Four parameters seem to be useful for comparison conformations of undecathiophene belts, screws and springs: (a) the distance between the ends, (b) the average of the module of the SCCS torsion angles, (c) the HOMA index of geometrical aromaticity,^27–29^ and (d) the sum of distances of the ten C–C bond linking the thiophene rings.

The first two parameters well characterize the conformer curvature and the degree of twist. Interestingly, the distance between the ends varies remarkably not only for belts but also for springs for which the number of twists changes a lot with the substituent. Use of the module operation is useful because the sign of the successive SCCS torsion angles alters for the belts, and although for the screws and springs it has always the same sign, it inverts with the change of the conformer chirality, *i.e.*, with the change of the handedness of the twist. Taking the average is also important because the end rings have more degrees of freedom and deviate from the values found for the internal units. Moreover, the studied structures are not very rigid. Thus, for each substituent several local minima occur and a diffusion of energy and geometrical parameters exist.

The last two parameters express the π-electron delocalization. Recently, we demonstrated that the geometrical aromaticity HOMA index is describing much more than only aromaticity and can reflect character of cyclic and acyclic saturated or partially unsaturated compounds.^30^ Besides, it can be used for describing the π-electron delocalization in conducting polymers.^31^ Indeed, the closer the HOMA index to 1.0, the closer is its π-electron structure to that in the reference benzene molecule, and the more perfectly the π-electron charge is delocalized.^27–31^ On the other hand, it is important that increase of the HOMA index on the other hand, associated with the decrease of the distances between ten thiophene rings denotes increase of both their double bond character and the π-electron delocalization of the studied structure.

The above two groups of parameters seemed to characterize two aspects of the studied molecules: the shape and the π-electron delocalization. Unexpectedly, all these characteristics are mutually correlated (Fig. 3). Thus, only *τ*(SCCS) and HOMA descriptors, more universal of the each group, are shown (Table 1) while the others are gathered in Table 4, ESI.†

**Fig. 3:**
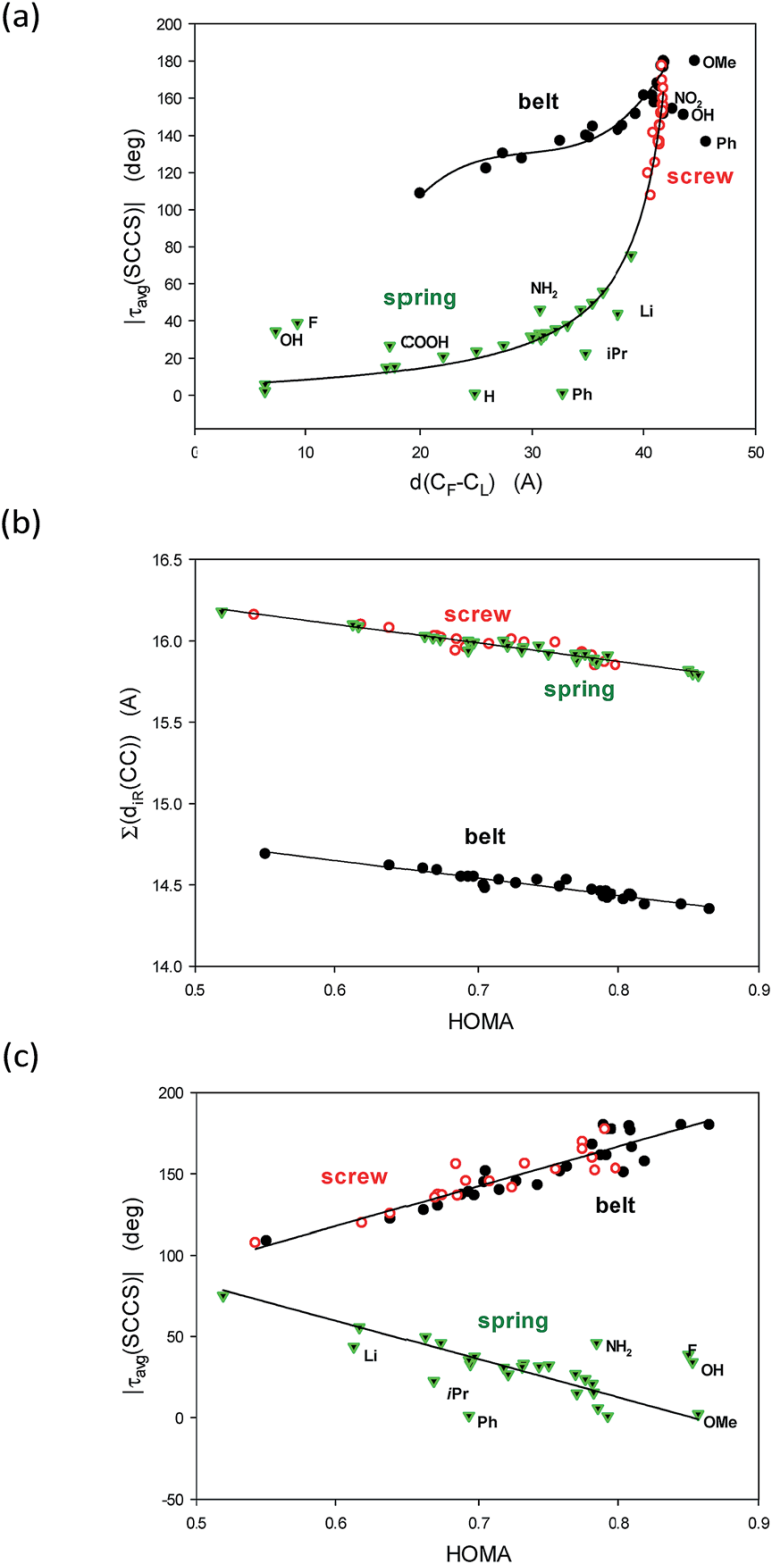
Correlations between (a) the distance between the ends *d*(C_1_–C_43_) (A) and the average of the module of the SCCS torsion angles |*τ*_avg_(SCCS)| (deg); (b) the HOMA index over the entire conjugated system and the sum of the ten C–C inter thiophene rings (iTR) bonds Σ(*d*_iTR_(C–C)) and (c) between the HOMA index and the |*τ*_av_(SCCS)| angle.

To understand the graphs shown in Fig. 3 let us remind that |*τ*_avg_(SCCS)| is close to 180 deg for flat all-transoid while it is close to 0 deg for flat all-cisoid structures. So, |*τ*_avg_(SCCS)| *ca.* 180 deg denotes a small deformation in belt and screw structures while for springs a small deformation is connected to |*τ*_avg_(SCCS)| approaching 0 deg. On the other hand, the distance between the ends decreases with an increase of the bow-bending of the belt and with a drop of the number of twists in the spring, while an increase of the twists in the screw. So, indeed |*τ*_avg_(SCCS)| and *d*(C_1_–C_43_) express concordantly, yet non-linearly, the conformer shape and deformation.

Fig. 3b is easy to interpret because HOMA approaching 1.0 denotes by definition the perfect π-electron delocalization which is connected to decrease of the sum of distances of the inter-thiophene C–C bonds, and simultaneously to increase of their double bond character. Probably the most striking is Fig. 3c demonstrating linear correlations between HOMA index and the |*τ*_avg_(SCCS)| torsion angles characteristic. It unequivocally shows evidence that the π-electron delocalization declines with the accumulation of the deformation in the structure would it be of the belt, the screw or the spring type.

The motivation for this project was to find a substituent able to stabilize a chiral screw or spring conformation and to conserve a good π-electron delocalization over the structure. Such a substituent could possibly be used in devices exploiting chirality-related properties and the circular flow of electrons in enantioselective: chemo- and bio-sensors, molecular switches, modulators or non-linear optic devices, *etc.* This is why we considered two dozens of substituents differing in their action on both σ- and π-electron system of aromatic molecules.

Since almost a decade we have been constructing sEDA and pEDA descriptors of the substituent and heteroatom incorporation effects.^32–35^ The sEDA and pEDA descriptors have simple physical sense: they show the amount of electrons donated to or withdrawn by the substituent or incorporant from the σ- and π-valence orbitals. However, the correlations between any of the structural parameters considered and the sEDA or the pEDA descriptors were not significant. The sEDA and pEDA descriptors were constructed based on substituted benzenes but were shown to be general enough to reveal the substituent effect in any similar system.^36^ Nevertheless, trying to find out why they do not reflect the effect in the substituted undecathiophenes, we tailored the descriptors based exactly on the 3-substituted thiophene core (sEDA_T_, pEDA_T_ and (s + p)EDA_T_, Table 5, ESI†). The modified sEDA_T_ and pEDA_T_ descriptors are also based on referencing the electron populations at the σ and π valence orbitals in the 3-substituted to the unsubstituted ring. However, to better extract the significant part of the substituent effect, the calculations were truncated only to the ring C-atoms responsible for the π-electron delocalization. Still, the new correlations seemed to be insignificant (Fig. 1, ESI†), although, for the HOMA index, after elimination of deviating points corresponding to bulky substituents, the *R*^2^ correlation coefficients increased from *ca.* 0.15 to over 0.5. This is especially true for the correlation of HOMA with sEDA_T_ for which *R*^2^ increased to *ca.* 0.65 (Fig. 1, ESI†).

Still, it remains striking that the shape of the studied systems undergoes dramatic but concordant changes with the substituent (Fig. 4a and b). Notice that the values for the belts are on the abscissae while for the screws and springs are on the ordinates. So, what characteristic of the substituent is governing these changes?

**Fig. 4:**
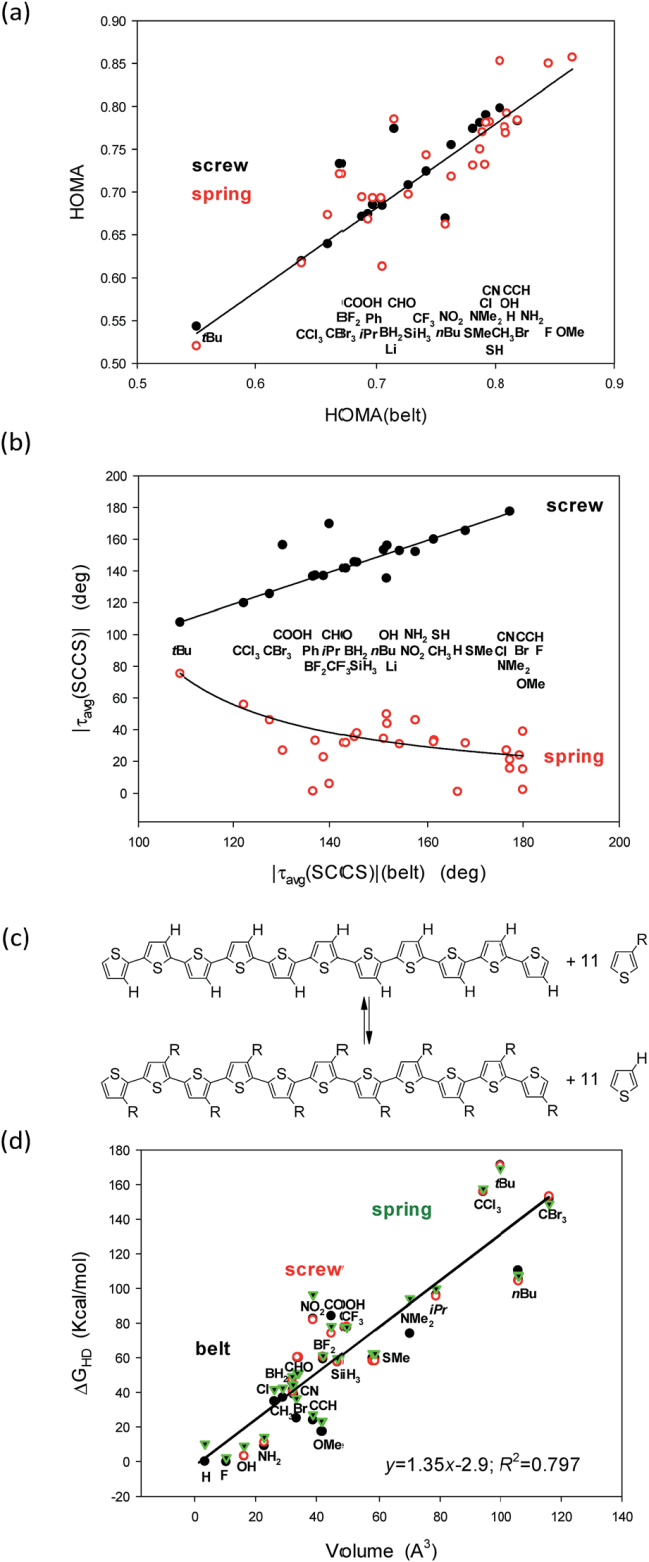
Correlations between (a) HOMA and (b) the |*τ*_av_(SCCS)| torsion angles in the belt conformers (*X* axis) and the screw and spring structures (*Y* axis). Notice that for some substituents the screw conformers converge to the belt ones and are absent. Hyperhomodesmotic reaction (c) and (d) the correlation between the energy effect of such a reaction and the volume of the substituent.

A closer inspection into correlations between the HOMA and |*τ*(SCCS)| parameters for the belts with the corresponding values for the screw and spring conformers allows to perceive that the largest deviations from HOMA = 1.0 and from |*τ*_avg_(SCCS)| = 180.0 or 0.0 deg increase with the substituent volume or hindrance (*t*Bu, CCl_3_, and CBr_3_). Indeed, for small substituents, the HOMA values are closer to 1.0 and the torsion angles approach 0 or 180 deg for springs or screws, respectively, while for bulky groups they strongly deviate from these values. Still, the substituent order is not fully clear (Fig. 4a and b), *e.g.*, why CCl_3_ is located between *t*Bu and CBr_3_ while it is less bulky than both of them? Further, why COOH is between CBr_3_ and Ph, while it is much smaller then each of them? And, after all, why H is between CH_3_ and OMe groups?

In search for the more precise answer to the question about the rule governing the HOMA and torsion angle changes we calculated the Gibbs free energies of the hyperhomodesmotic (Δ*G*_HD_) reactions (Fig. 4c and Table 3, ESI†). The energies of such reactions would give quite exact values if we could use a higher level of theory and make an extensive conformational study.^37^ They estimate the energetic difference between the eleven substituted single thiophene molecules and the C-shaped undecathiophene substituted in the regioregular HT way.^24^ Hence, one can expect that Δ*G*_HD_ contains contributions from the substituents interaction with: (1) the entire undecathiophene π-electron system; (2) the undecathiophene σ-electron skeleton; and (3) with the ten adjacent rings. The latter contacts can have very complex nature as they can be both attractive and repulsive and related to both the substituent volume, hindrance as well as formation of a hydrogen-, halogen-, chalcogen-, *etc.* bond. Notice that in the belt and screw conformations the substituent interacts with the S-atom of the adjacent ring, while in the spring ones with the CH group in the position 4.

As a result of attempts for correlations of Δ*G*_HD_ with sEDA_T_, pEDA_T_, (s + p)EDA_T_ substituent effect parameters and substituent volume, surface, and widths estimated using simple approximations (Table 6, ESI†) it turned out that the substituent volume explains in 80% variations of Δ*G*_HD_ of each of the studied conformers (Fig. 4d). In the next step we tested the correlations against both: the volume and sEDA_T_ descriptor, which explained quite a lot of changes if the data of some bulky substituents were eliminated. Nevertheless, none of the two parameter correlations was stronger than that presented in Fig. 4d even if some points were ignored. After all, the order of the substituents perturbing only slightly the systems suggests that the π-electron donating substituents (*e.g.*, OMe, NH_2_, SMe) support high value of HOMA in spring conformers (Fig. 4a). Unfortunately, majority of those substituents seem to have too small volume to stabilize the screw forms (Table 1). They also promote structure flattening and only a very weak spring twist. Thus we can guess that quite unsophisticated SMe and SH groups, for which synthesis of eleven-substituted undecathiophene seems to be feasible, can stabilize both screw and spring helices conserving at the same time a fair π-electron delocalization with HOMA close to 0.78 (Fig. 4, Table 1).

One of the reviewers of this letter rightly pointed out that to characterize helices, the helix pitch, helix radius and number of units per turn as well as bond length alternation (BLA) are worth to be given.^21^ Finding the helix parameters require fitting the helix coordinates by a least square routine.^38^ The pitch, radius, and number of thiophene rings per turn for the studied helical structures (Table 7, ESI†) were found here using the HELFIX program^38^ operating on coordinates of 11 S-atoms. It appeared that there are nice quadratic correlations between these parameters and |*τ*_avg_(SCCS)|. Also, the number of thiophene rings per turn and the radius as well as BLA and HOMA are significantly correlated (Fig. 2, ESI†). However, a significant spread of points exists and it has the same reasons as the spreads presented in figures above. Therefore, we believe that proposed presentation of the substituent effect problem could be equivalent to such in which the helix parameters would play a central role.

## Conclusions

The belt- and two chiral: screw- and spring- helical conformations of regioregular head-to-tail undecathiophenes substituted with 26 different functional groups were calculated at the B3LYP/6-31G** level. Comparison of the conformer energies suggests that the screw and spring structures can be formed, can often co-exist, and can sometimes be even more stable than the belt ones. The four geometrical characteristics considered, the averaged SCCS torsion angle, the HOMA geometrical aromaticity index, the sum of distances of bonds linking the rings, and the distance between the conformers ends appeared to be intercorrelated, yet, not always linearly. Especially first two parameters well characterize the geometry and the π-electron delocalization. Correlation of the energy of the hyperhomodesmotic reaction estimated for all the conformers and the substituent volume demonstrated that the volume is in *ca.* 80% responsible for the conformer shape. Based on the geometry-derived parameters we hypothesize that some common substituents like SMe or SH can support helical conformation of undecathiophenes with simultaneous conservation of a fair π-electron delocalization.

## Conflicts of interest

There are no conflicts to declare.

## Supplementary Material

RA-008-C7RA12777D-s001

## References

[cit1] Zhang L., Colella N. S., Cherniawski B. P., Mannsfeld S. C., Briseno A. L. (2014). ACS Appl. Mater. Interfaces.

[cit2] Beaujuge P. M., Frechet J. M. (2011). J. Am. Chem. Soc..

[cit3] Heeney M., Bailey C., Genevicius K., Shkunov M., Soarrow D., Tierney S., McCulloch I. (2005). J. Am. Chem. Soc..

[cit4] Lemaire M., Delabouglise D., Garreau R., Guy A., Roncali J. (1988). J. Chem. Soc., Chem. Commun..

[cit5] Kane-Maguire L. A. P., Wallace G. G. (2010). Chem. Soc. Rev..

[cit6] Moss G. P. (1996). Pure Appl. Chem..

[cit7] Langeveld-Voss B. M. W., Janssen R. A. J., Meijer E. W. (2000). J. Mol. Struct..

[cit8] Padula D., Santoro F., Pescitelli G. (2016). RSC Adv..

[cit9] Goto H., Akagi K., Dai X., Narihiro H. (2007). Ferroelectrics.

[cit10] Ohkawa S., Yang F., Kawabata K., Goto H. (2013). J. Appl. Polym. Sci..

[cit11] Tsuchiya T., Yamamoto S., Shinkai T., Shiraki A., Dawn Y. (2012). Chem. Commun..

[cit12] Kameta N., Masuda M., Shimizu T. (2016). Chem. Commun..

[cit13] Garnier F., Tourillon G., Barraud J. Y., Dexpert H. (1985). J. Mater. Sci..

[cit14] Yang R., Dalsin K. M., Evans D. F., Christensen L., Hendrickson W. A. (1989). J. Phys. Chem..

[cit15] Yang R., Evans D. F., Christensen L., Hendrickson W. A. (1990). J. Phys. Chem..

[cit16] Caple G., Wheeler B. L., Swift R., Porter T. L., Jeffers S. (1990). J. Phys. Chem..

[cit17] Cui C. X., Kertesz M. (1989). Phys. Rev. B: Condens. Matter Mater. Phys..

[cit18] Corish J., Morton-Blake D. A., Veluri K., Bénière F. (1995). Mol. Simul..

[cit19] Corish J., Morton-Blake D. A., Veluri K., Bénière F. (1995). Radiat. Eff. Defects Solids.

[cit20] O'Farrell R., O'Dwyer S., Morton-Blake D. A. (2004). Mol. Simul..

[cit21] Sahu H., Panda A. N. (2015). J. Phys. Chem. C.

[cit22] Morton-Blake D. A. (2016). Diffus. Found..

[cit23] McCullough R. D. (1998). Adv. Mater..

[cit24] Peverati R., Truhlar D. G. (2011). Philos. Trans. R. Soc., A.

[cit25] Medvedev M. G., Bushmarinov I. S., Sun J., Perdew J. P., Lyssenko K. A. (2017). Science.

[cit26] Kruszewski J., Krygowski T. M. (1972). Tetrahedron Lett..

[cit27] Cyrański M. K. (2005). Chem. Rev..

[cit28] Krygowski T. M., Szatylowicz H., Stasyuk O. A., Dominikowska J., Palusiak M. (2014). Chem. Rev..

[cit29] Ostrowski S., Dobrowolski J. Cz. (2014). RSC Adv..

[cit30] Dobrowolski J. Cz., Ostrowski S. (2015). RSC Adv..

[cit31] Enkhbayar P., Damdinsuren S., Osaki M., Matsushima N. (2008). Comput. Biol. Chem..

[cit32] Ozimiński W. P., Dobrowolski J. Cz. (2009). J. Phys. Org. Chem..

[cit33] Mazurek A., Dobrowolski J. Cz. (2012). J. Org. Chem..

[cit34] Mazurek A., Dobrowolski J. Cz. (2013). Org. Biomol. Chem..

[cit35] Mazurek A., Dobrowolski J. Cz. (2015). J. Phys. Org. Chem..

[cit36] Mazurek A. (2016). Acta Pol. Pharm..

[cit37] Wheeler S. E., Houk K. N., Schleyer P. v. R., Allen W. D. (2009). J. Am. Chem. Soc..

[cit38] Enkhbayar P., Damdinsuren S., Osaki M., Matsushima N. (2008). Comput. Biol. Chem..

[cit39] Becke A. D. (1993). J. Chem. Phys..

[cit40] Lee C., Yang W., Parr R. G. (1988). Phys. Rev. B: Condens. Matter Mater. Phys..

[cit41] Ditchfield R., Hehre W. J., Pople J. A. (1971). J. Chem. Phys..

[cit42] Frisch M. J., Pople J. A., Binkley J. S. (1984). J. Chem. Phys..

[cit43] Frisch M. J. , Trucks G. W., Schlegel H. B., Scuseria G. E., Robb M. A., Cheeseman J. R., Scalmani G., Barone V., Mennucci B., Petersson G. A., Nakatsuji H., Caricato M., Li X., Hratchian H. P., Izmaylov A. F., Bloino J., Zheng G., Sonnenberg J. L., Hada M., Ehara M., Toyota K., Fukuda R., Hasegawa J., Ishida M., Nakajima T., Honda Y., Kitao O., Nakai H., Vreven T., Montgomery J. A., Peralta J. E., Ogliaro F., Bearpark M., Heyd J. J., Brothers E., Kudin K. N., Staroverov V. N., Kobayashi R., Normand J., Raghavachari K., Rendell A., Burant J. C., Iyengar S. S., Tomasi J., Cossi M., Rega N., Millam N. J., Klene M., Knox J. E., Cross J. B., Bakken V., Adamo C., Jaramillo J., Gomperts R., Stratmann R. E., Yazyev O., Austin A. J., Cammi R., Pomelli C., Ochterski J. W., Martin R. L., Morokuma K., Zakrzewski V. G., Voth G. A., Salvador P., Dannenberg J. J., Dapprich S., Daniels A. D., Farkas Ö., Foresman J. B., Ortiz J. V., Cioslowski J. and Fox D. J., Gaussian 09, Revision C.01, Gaussian, Inc., Wallingford CT, 2009

[cit44] SigmaPlot 13, Copyright © 2017, Systat Software, Inc.

