# Supplementary material for: On the variation of the belt and chiral screw and spring conformations of substituted regioregular HT undecathiophenes
Source: RSC Adv. 2018 Jan 9;8(4):2116–22. doi: 10.1039/c7ra12777d (PMC9077278; doi:10.1039/c7ra12777d)
Supplement: RA-008-C7RA12777D-s001 [file RA-008-C7RA12777D-s001.pdf]

Electronic Supplementary Material (ESI) for RSC Advances.
This journal is © The Royal Society of Chemistry 2018

Supplementary Information file to the article

On variation of the belt and chiral screw and spring conformations of substituted regioregular HT undecathiophenes

by Jan Cz. Dobrowolski* and Małgorzata E. Jamróz

Object	Caption	Page
**Table 1ESI.**	The B3LYP and LC-ωB97XD total energies and Gibbs free energy differences (ΔE or ΔG298, kcal/mol) for the S-shaped, C-shaped and P-planar dodecathiophenes calculated using 6-31G**, cc-pVTZ and cc-pVQZ basis sets (composed of 989, 2030, and 3789 basis functions, respectively). For B3LYP/6-31G** and B3LYP/cc-pVTZ calculations the role of Grimme's D3 correction for dispersion interactions was also checked. ΔEC and ΔG298,C denote that the appropriate energies of the C conformer were subtracted. nc stands for not converged	**3**
**Table 2ESI.**	Gibbs free energy and total energy (hartree) of the belt, screw and spring conformers of undecathiophenes and 3-substituted thiophenes calculated at the B3LYP/6-31G** level	**4**
**Table 3ESI.**	Gibbs free energy and total energy and energy of hyperhomodesmotic reaction (kcal/mol) for the belt, screw and spring conformers of undecathiophenes calculated at the B3LYP/6-31G** level. Gibbs free energy and total energy (hartree) of the 3-substituted thiophenes calculated at the B3LYP/6-31G** level.	**5**
**Table 4ESI.**	Four parameters applied to characterize the substituted belt, screw and spring conformers of undecathiophenes calculated at the B3LYP/6-31G** level. HOMA is the index calculated based on 43 CC bonds, 1st-last is the distance (in angstroms) between the first and 43rd C-atom, tau is the mean of the absolute values of all SCCS torsion angles, and Σ(c=c) is the sum of all CC distances linking the thiophene rings (in angstroms).	**6**
**Table 4A-ESI.**	The subsequent CC distances (Å) in the studied belt structures calculated at the B3LYP/6-31G** level	**7**
**Table 4B-ESI.**	The subsequent CC distances (Å) in the studied screw structures calculated at the B3LYP/6-31G** level	**10**
**Table 4C-ESI.**	The subsequent CC distances (Å) in the studied spring structures calculated at the B3LYP/6-31G** level	**13**
**Table 5ESI.**	Population of the σ- and π- valence orbitals in 3-substituted thiophenes and substituent effect descriptors based on the thiophene ring C-atoms based on NBO populations calculated from B3LYP/6-31G** optimized structures.	**16**
**Table 6ESI.**	Volume of 3-substituted thiophenes and pure substituents estimated based on cavity volume or surface calculated assuming the scrf= (iefpcm,solvent=water, read) key and PCMDoc PDens=1.0 as the "read" parameters using the single point at the 6-31G** calculations of the structures optimized in vacuum. Index "rel" means that unsubstituted and thiophene with abstracted hydrogen in position 3 was subtracted. L_{subst} denotes length of the substituent from the ipso C-atom to the most distanced atom of the substituent or the position of the face defined by atoms as in CF_3 or CBr_3 groups. W is the substituent width calculated from the cavity, and surface, assuming that the substituent volume is equal to the volume of a cylinder, or a cuboid or with one dimension equal to two radii of the bulkiest atom of the substituent.	**17**
**Table 7ESI.**	Parameters of helix fitted using HELFIT program1 based on coordinates of eleven S-atoms of each screw and spring conformers of the substituted undecathiophenes. The helix equation is expressed as follows: $x=a\cdot\cos(t)$; $y=a\cdot\sin(t)$; $z=b\cdot t$, where a is the helix radius and pitch is defined as $2\cdot\pi\cdot b$, a and pitch are in angstroms. Number of thiophene rings per turn is denoted as NTRT. Bond length alternation (BLA), defined as the difference between the average long and short C-C distances in the conjugated π system.	**18**
**Fig. 1ESI.**	Correlations between substituent effect descriptors sEDAT (a, b), pEDAT (c,d) and (s+p)EDAT and HOMA index (a, c, e) and averaged module of the torsion SCCS angle (b, d, f) of the belt (black), screw (red) and spring (green) conformations of eleven substituted undecathiophenes	**19**
**Fig. 2ESI.**	Correlations between the averaged module of the torsion SCCS angle and helix radius (a) and helix pitch (b) and between the helix radius and number of thiophene rings per turn NTRT (c) for screw and spring undecathiophenes. Linear correlations between HOMA index and the bond length alternation BLA (d). The spread of points have the same reasons as the spread of points in figures presented in the main text.	**20**
**Coordinates**	BELTS XYZ	**21**
**Coordinates**	SCREWS XYZ	**93**
**Coordinates**	SPRINGS XYZ	**141**

Table 1ESI. The B3LYP and LC-ωB97XD total energies and Gibbs free energy differences (ΔE or ΔG_{298}, kcal/mol) for the S-shaped, C-shaped and P-planar dodecathiophenes calculated using 6-31G**, cc-pVTZ and cc-pVQZ basis sets (composed of 989, 2030, and 3789 basis functions, respectively). For B3LYP/6-31G** and B3LYP/cc-pVTZ calculations the role of Grimme's D3 correction for dispersion interactions was also checked. ΔE_C and $\Delta G_{298,C}$ denote that the appropriate energies of the C conformer were subtracted. nc stands for not converged

		B3LYP					LC-ωB97XD				
System		6-31G** (989)		cc-pVTZ (2030)		cc-pVQZ (3789)	6-31G** (989)		cc-pVTZ (2030)		cc-pVQZ (3789)
		ΔE_C	$\Delta G_{298,C}$	ΔE_C	$\Delta G_{298,C}$	ΔE_C	ΔE_C	$\Delta G_{298,C}$	ΔE_C	$\Delta G_{298,C}$	ΔE_C
S		0.038	-2.260	0.001	-0.016	0.003	-0.038	-0.334	0.051	0.741	-0.065
C		0.000	0.000	0.000	0.000	0.000	0.000	0.000	0.000	0.000	0.000
P		0.140	5.412	0.019	3.368	0.053	2.081	12.961	1.351	nc	1.498
S	D3	0.059	-0.465	0.013	-1.037						
C	D3	0.000	0.000	0.000	0.000						
P	D3	0.292	5.737	0.082	1.189						

Table 2ESI. Gibbs free energy and total energy (hartree) of the belt, screw and spring conformers of undecathiophenes and3-substituted thiophenes calculated at the B3LYP/6-31G** level

Subst	belt		screw		spring			3-substituted-thiophene	
	ΔG	ΔE	ΔG	ΔE	ΔG	ΔE		ΔG	ΔE
BF_2	-8535.5480870	-8535.9360783	-8535.5459310	-8535.9327523	-8535.5448200	-8535.9364377	BF_2	-777.0534730	-777.0881418
BH_2	-6350.5271780	-6351.0765442	-6350.5251570	-6351.0734292	-6350.5220960	-6351.072251	BH_2	-578.4132970	-578.4622941
Br	-34353.0002650	-34353.2948990			-34352.9819360	-34353.27996	Br	-3124.0896280	-3124.1158144
CBr_3	-91349.5206880	-91349.8223856	-91349.5186010	-91349.8202268	-91349.5256800	-91349.82092	CBr_3	-8305.6098710	-8305.6350900
CCH	-6908.2746460	-6908.7805500			-6908.2695160	-6908.773595	CCH	-629.1144160	-629.1599574
CCl_3	-21669.5596600	-21669.9090831	-21669.5592060	-21669.9074551	-21669.5568600	-21669.90778	CCl_3	-1971.0685280	-1971.0986725
CF_3	-9778.0955560	-9778.5334639	-9778.0947430	-9778.5307904	-9778.0950520	-9778.534968	CF_3	-890.0149860	-890.0526552
CH_3	-6502.9836040	-6503.7054537		-6503.7052797	-6502.9747840	-6503.70089	CH_3	-592.2716740	-592.3365891
CHO	-7317.1965550	-7317.7096827	-7317.1960230	-7317.7186540	-7317.2106980	-7317.722105	CHO	-666.2943600	-666.3402677
Cl	-11126.3623440	-11126.6705674			-11126.3513580	-11126.66383	Cl	-1012.5785150	-1012.6062667
CN	-7085.4176990	-7085.809503			-7085.4121110	-7085.804476	CN	-645.2204810	-645.2559164
COOH	-8144.9065230	-8145.462681	-8144.9226660	-8145.4841384	-8144.9161230	-8145.476469	COOH	-741.5442350	-741.5944736
F	-7162.3510040	-7162.690718			-7162.3472060	-7162.67995	F	-652.2087780	-652.2388880
H	-6070.7582050	-6071.193693			-6070.7419900	-6071.178945	H	-552.9730920	-553.0125893
*i*Pr	-7367.3380120	-7368.649262	-7367.3391580	-7368.6487730	-7367.3320880	-7368.647787	*i*Pr	-670.8579020	-670.9763847
Li	-6146.9336200	-6147.2444284	-6146.9360230	-6147.2480919	-6146.9267430	-6147.239798	Li	-559.8848060	-559.9112166
*n*Bu	-7799.5318800	-7801.1317538	-7799.5413470	-7801.1339014	-7799.5360690	-7801.132937	*n*Bu	-710.1503170	-710.2946631
NH_2	-6679.4957700	-6680.1080085	-6679.4924130	-6680.1026437	-6679.4879410	-6680.103471	NH_2	-608.3141800	-608.3687223
NMe_2	-7543.6809460	-7544.8587845			-7543.6488970	-7544.841367	NMe_2	-686.8858760	-686.9931638
NO_2	-8320.1538540	-8320.5823314	-8320.1549810	-8320.5819176	-8320.1321000	-8320.554414	NO_2	-757.4755720	-757.5136943
OH	-6898.1069530	-6898.5831067	-6898.1068340	-6898.5832095	-6898.0975230	-6898.563847	OH	-628.1870670	-628.2292842
OMe	-7330.1718260	-7330.934451			-7330.1621090	-7330.91896	OMe	-667.4677450	-667.5391982
Ph	-8611.5948130	-8612.8521383	-8611.5963010	-8612.8501504	-8611.5913100	-8612.84877	Ph	-783.9734500	-784.0871025
SH	-10450.8153390	-10451.2100589	-10450.8169170	-10451.2111114	-10450.8103970	-10451.21138	SH	-951.1660680	-951.2015018
SiH_3	-9268.2753050	-9268.848469	-9268.2749990	-9268.8452129	-9268.2717300	-9268.8481234	SiH_3	-843.6647770	-843.7155198
SMe	-10883.0018570	-10883.7029210	-10883.0041940	-10883.7048198	-10882.9972060	-10883.6948447	SMe	-990.4584180	-990.5210681
*t*Bu	-7799.4289830	-7801.0399397	-7799.4298360	-7801.0413442	-7799.4324640	-7801.039914	*t*Bu	-710.1498150	-710.2949276

Table 3ESI. Gibbs free energy and total energy and energy of hyperhomodesmotic reaction (kcal/mol) for the belt, screw and spring conformers of undecathiophenes calculated at the B3LYP/6-31G** level. Gibbs free energy and total energy (hartree) of the 3-substituted thiophenes calculated at the B3LYP/6-31G** level.

Substituent	Energy difference with respect to belt						hyperhomodesmotic					
	belt		screw		spring		belt		screw		spring	
	ΔG	ΔE	ΔG	ΔE	ΔG	ΔE	ΔG	ΔE	ΔG	ΔE	ΔG	ΔE
BF_2	0.0	0.0	1.4	2.1	2.1	-0.2	59.2	55.7	60.5	57.7	61.2	55.4
BH_2	0.0	0.0	1.3	2.0	3.2	2.7	46.0	40.1	47.3	42.1	49.2	42.8
Br	0.0	0.0	$\rightarrow$ b		11.5	9.4	25.0	21.5			36.5	30.9
CBr_3	0.0	0.0	1.3	1.4	-3.1	0.9	151.9	137.3	153.2	138.7	148.8	138.2
CCH	0.0	0.0	$\rightarrow$ b		3.2	4.4	23.9	21.5			27.1	25.8
CCl_3	0.0	0.0	0.3	1.0	1.8	0.8	155.8	145.3	156.1	146.3	157.6	146.1
CF_3	0.0	0.0	0.5	1.7	0.3	-0.9	77.5	63.3	78.0	65.0	77.8	62.4
CH_3	0.0	0.0	$\rightarrow$ b	0.1	5.5	2.9	37.0	32.8			42.6	35.6
CHO	0.0	0.0	0.3	-5.6	-8.9	-7.8	60.0	55.5	60.3	49.9	51.1	47.7
Cl	0.0	0.0	$\rightarrow$ b		6.9	4.2	34.8	33.6			41.7	37.8
CN	0.0	0.0	$\rightarrow$ b		3.5	3.2	38.8	38.1			42.3	41.3
COOH	0.0	0.0	-10.1	-13.5	-6.0	-8.7	84.2	82.7	74.1	69.2	78.2	74.0
F	0.0	0.0	$\rightarrow$ b		2.4	6.8	-0.2	-4.9			2.2	1.9
H	0.0	0.0	$\rightarrow$ b		10.2	9.3	0.0	0.0			10.2	9.3
*i*Pr	0.0	0.0	-0.7	0.3	3.7	0.9	96.1	91.7	95.4	92.0	99.8	92.7
Li	0.0	0.0	-1.5	-2.3	4.3	2.9	-92.0	-104.1	-93.5	-106.4	-87.7	-101.2
*n*Bu	0.0	0.0	-5.9	-1.3	-2.6	-0.7	110.3	103.4	104.4	102.0	107.7	102.6
NH_2	0.0	0.0	2.1	3.4	4.9	2.8	9.0	2.0	11.1	5.3	14.0	4.8
NMe_2	0.0	0.0	$\rightarrow$ b		20.1	10.9	74.0	76.1			94.1	87.0
NO_2	0.0	0.0	-0.7	0.3	13.7	17.5	82.6	77.5	81.9	77.8	96.3	95.0
OH	0.0	0.0	0.1	-0.1	5.9	12.1	3.1	-3.6	3.2	-3.7	9.0	8.5
OMe	0.0	0.0	$\rightarrow$ b		6.1	9.7	17.3	32.6			23.4	42.3
Ph	0.0	0.0	-0.9	1.2	2.2	2.1	105.0	101.2	104.1	102.4	107.2	103.3
SH	0.0	0.0	-1.0	-0.7	3.1	-0.8	41.2	38.7	40.2	38.0	44.3	37.9
SiH_3	0.0	0.0	0.2	2.0	2.2	0.2	57.4	48.6	57.6	50.7	59.6	48.8
SMe	0.0	0.0	-1.5	-1.2	2.9	5.1	59.6	52.7	58.1	51.5	62.5	57.8
*t*Bu	0.0	0.0	-0.5	-0.9	-2.2	0.0	171.4	162.8	170.9	161.9	169.2	162.8

Table 4ESI. Four parameters applied to characterize the substituted belt, screw and spring conformers of undecathiophenes calculated at the B3LYP/6-31G** level. HOMA is the index calculated based on 43 CC bonds, 1st-last is the distance (in angstroms) between the first and 43rd C-atom, tau is the mean of the absolute values of all SCCS torsion angles, and Σ(c=c) is the sum of all CC distances linking the thiophene rings (in angstroms).

Substituent	belt				screw				spring			
	HOMA	1st-last	tau	Σ(c=c)	HOMA	1st-last	tau	Σ(c=c)	HOMA	1st-last	tau	Σ(c=c)
BF_2	0.688	32.5	137.1	14.55	0.671	41.34	137.1	16.03	0.694	30.64	32.9	15.98
BH_2	0.704	35.4	144.8	14.50	0.691	41.46	145.5	15.96	0.693	32.08	35.4	15.94
Br	0.808	41.9	179.3	14.44					0.776	25.02	23.7	15.92
CBr_3	0.661	29.1	127.6	14.60	0.639	41.06	125.4	16.08	0.673	34.31	45.9	16.01
CCH	0.789	41.8	180.0	14.43					0.770	17.25	14.9	15.88
CCl_3	0.639	25.9	122.2	14.62	0.619	40.41	119.7	16.10	0.617	36.32	55.6	16.09
CF_3	0.742	37.7	143.0	14.53	0.724	40.87	141.5	16.01	0.743	29.91	31.5	15.97
CH_3	0.791	40.8	161.4	14.46					0.732	31.21	33.2	15.96
CHO	0.715	34.8	140.0	14.53	0.774	41.67	169.6	15.93	0.785	6.42	5.8	15.87
Cl	0.809	41.8	176.6	14.44					0.769	27.44	26.8	15.92
CN	0.795	41.7	177.3	14.44					0.782	17.98	15.4	15.89
COOH	0.671	27.4	130.3	14.59	0.733	41.69	156.2	15.99	0.721	17.54	26.7	15.97
F	0.845	41.8	180.0	14.38					0.850	9.29	38.7	15.82
H	0.810	41.4	166.3	14.43					0.792	24.86	0.9	15.91
*i*Pr	0.693	35.1	138.8	14.55	0.674	41.46	136.8	16.02	0.668	34.75	22.5	16.02
Li	0.705	41.8	151.6	14.48	0.684	41.78	155.9	15.94	0.613	37.68	43.6	16.10
*n*Bu	0.758	39.3	151.5	14.49	0.669	41.44	135.2	16.03	0.662	35.36	49.6	16.03
NH_2	0.819	41.0	157.5	14.38	0.783	41.59	152.0	15.85	0.784	30.69	45.9	15.87
NMe_2	0.792	41.6	177.3	14.42	0.790	41.64	177.4	15.87	0.781	22.06	20.9	15.89
NO_2	0.763	42.6	154.2	14.53	0.755	41.68	152.6	15.99	0.718	30.79	30.7	16.00
OH	0.804	43.6	150.9	14.41	0.798	41.73	153.1	15.85	0.853	7.37	34.2	15.80
OMe	0.865	44.6	180.0	14.35					0.857	6.41	2.2	15.79
Ph	0.697	45.6	136.6	14.55	0.685	41.34	136.5	16.01	0.693	32.69	1.2	16.00
SH	0.787	40.06	161.3	14.46	0.781	41.73	159.8	15.91	0.750	30.99	32.2	15.92
SiH_3	0.727	38.11	145.3	14.51	0.708	41.50	145.3	15.98	0.697	33.16	37.6	15.99
SMe	0.781	41.24	167.9	14.47	0.774	41.78	165.2	15.92	0.731	30.05	31.3	15.94
*t*Bu	0.551	20.00	108.7	14.69	0.543	40.66	107.6	16.16	0.520	38.90	75.2	16.18

Table 4A-ESI. The subsequent CC distances (Å) in the studied belt structures calculated at the B3LYP/6-31G** level

BF_2	BH_2	Br	CBr_3	CCH	CCl_3	CF_3	CH_3	CHO	Cl	CN	COOH	F	H
1.38065	1.38859	1.36305	1.36273	1.37994	1.36345	1.36908	1.37017	1.37702	1.36649	1.37975	1.37585	1.36619	1.36770
1.43063	1.43541	1.42390	1.43416	1.42950	1.43287	1.42293	1.42825	1.42755	1.41917	1.42588	1.42598	1.41722	1.42285
1.37698	1.37636	1.38156	1.39241	1.37992	1.38993	1.37742	1.37951	1.37419	1.38095	1.38007	1.37458	1.37784	1.37936
1.45723	1.45201	1.44729	1.46109	1.44605	1.46308	1.45597	1.45045	1.45466	1.44806	1.44706	1.46024	1.44137	1.44677
1.39559	1.40582	1.37941	1.37410	1.39674	1.37367	1.38624	1.38786	1.39239	1.38241	1.39641	1.39194	1.38030	1.37985
1.42881	1.43192	1.41211	1.42640	1.42442	1.42651	1.41712	1.42007	1.42435	1.41335	1.42106	1.42457	1.40786	1.41505
1.37716	1.37681	1.38235	1.39419	1.37859	1.39106	1.37943	1.38158	1.37409	1.38068	1.37902	1.37423	1.37897	1.38101
1.45480	1.44919	1.44361	1.45971	1.44226	1.46221	1.45301	1.44574	1.45248	1.44386	1.44341	1.45873	1.43726	1.44251
1.39674	1.40731	1.38109	1.37571	1.39806	1.37459	1.38800	1.39026	1.39348	1.38424	1.39771	1.39293	1.38213	1.38151
1.42875	1.43152	1.41113	1.42540	1.42351	1.42583	1.41650	1.41832	1.42397	1.41210	1.42019	1.42428	1.40673	1.41364
1.37681	1.37682	1.38302	1.39451	1.37919	1.39115	1.37962	1.38246	1.37412	1.38100	1.37942	1.37428	1.37973	1.38174
1.45476	1.44867	1.44295	1.45930	1.44169	1.46197	1.45252	1.44429	1.45210	1.44294	1.44293	1.45838	1.43636	1.44157
1.39660	1.40752	1.38140	1.37596	1.39831	1.37466	1.38837	1.39117	1.39367	1.38464	1.39794	1.39306	1.38256	1.38192
1.42844	1.43153	1.41076	1.42521	1.42323	1.42586	1.41632	1.41771	1.42390	1.41176	1.41994	1.42448	1.40635	1.41327
1.37728	1.37681	1.38320	1.39431	1.37937	1.39132	1.37963	1.38249	1.37421	1.38129	1.37955	1.37397	1.37991	1.38198
1.45464	1.44870	1.44278	1.45947	1.44157	1.46186	1.45247	1.44399	1.45206	1.44269	1.44289	1.45857	1.43610	1.44128
1.39687	1.40754	1.38150	1.37561	1.39834	1.37483	1.38827	1.39117	1.39375	1.38478	1.39792	1.39282	1.38268	1.38204
1.42877	1.43141	1.41081	1.42562	1.42319	1.42581	1.41651	1.41752	1.42384	1.41158	1.41991	1.42426	1.40635	1.41307
1.37683	1.37688	1.38320	1.39442	1.37944	1.39143	1.37959	1.38288	1.37414	1.38140	1.37962	1.37431	1.37992	1.38207
1.45480	1.44864	1.44275	1.45939	1.44155	1.46184	1.45267	1.44371	1.45206	1.44259	1.44294	1.45844	1.43602	1.44118
1.39654	1.40758	1.38147	1.37584	1.39834	1.37492	1.38822	1.39160	1.39367	1.38481	1.39787	1.39299	1.38267	1.38208
1.42850	1.43158	1.41075	1.42520	1.42314	1.42556	1.41629	1.41761	1.42394	1.41151	1.41988	1.42435	1.40634	1.41306
1.37721	1.37676	1.38320	1.39407	1.37946	1.39113	1.37967	1.38234	1.37421	1.38147	1.37964	1.37419	1.37989	1.38208
1.45474	1.44874	1.44275	1.45955	1.44156	1.46192	1.45268	1.44415	1.45209	1.44257	1.44300	1.45842	1.43606	1.44118
1.39674	1.40746	1.38142	1.37548	1.39832	1.37469	1.38822	1.39099	1.39373	1.38484	1.39779	1.39293	1.38259	1.38207
1.42887	1.43151	1.41086	1.42566	1.42317	1.42593	1.41672	1.41775	1.42398	1.41151	1.41993	1.42426	1.40647	1.41307
1.37679	1.37679	1.38316	1.39441	1.37946	1.39131	1.37945	1.38275	1.37400	1.38148	1.37965	1.37421	1.37981	1.38204
1.45495	1.44884	1.44281	1.45940	1.44159	1.46184	1.45279	1.44411	1.45232	1.44260	1.44307	1.45848	1.43618	1.44128
1.39641	1.40743	1.38130	1.37591	1.39828	1.37472	1.38794	1.39125	1.39351	1.38481	1.39768	1.39287	1.38242	1.38198
1.42853	1.43175	1.41094	1.42544	1.42320	1.42571	1.41640	1.41792	1.42411	1.41151	1.42000	1.42458	1.40665	1.41327
1.37715	1.37668	1.38308	1.39402	1.37939	1.39130	1.37944	1.38225	1.37416	1.38141	1.37958	1.37398	1.37964	1.38192
1.45488	1.44901	1.44299	1.45954	1.44174	1.46192	1.45290	1.44454	1.45235	1.44277	1.44322	1.45860	1.43652	1.44157
1.39654	1.40718	1.38102	1.37532	1.39811	1.37465	1.38772	1.39071	1.39353	1.38466	1.39737	1.39268	1.38203	1.38174

1.42912	1.43213	1.41130	1.42575	1.42348	1.42592	1.41690	1.41825	1.42441	1.41183	1.42039	1.42460	1.40717	1.41364
1.37651	1.37636	1.38277	1.39468	1.37908	1.39115	1.37932	1.38206	1.37386	1.38109	1.37928	1.37392	1.37918	1.38151
1.45543	1.44964	1.44365	1.45951	1.44234	1.46205	1.45331	1.44560	1.45292	1.44343	1.44376	1.45895	1.43752	1.44251
1.39598	1.40683	1.38021	1.37608	1.39735	1.37464	1.38755	1.39024	1.39325	1.38393	1.39639	1.39241	1.38105	1.38101
1.42983	1.43303	1.41242	1.42547	1.42468	1.42620	1.41780	1.42017	1.42516	1.41284	1.42163	1.42557	1.40865	1.41505
1.37555	1.37503	1.38142	1.39318	1.37738	1.39060	1.37792	1.37957	1.37291	1.37943	1.37758	1.37279	1.37742	1.37985
1.45731	1.45198	1.44755	1.46158	1.44576	1.46336	1.45550	1.45020	1.45487	1.44703	1.44715	1.46022	1.44200	1.44677
1.39352	1.40384	1.38181	1.37262	1.39552	1.37239	1.38534	1.38707	1.39225	1.38293	1.39453	1.39062	1.37948	1.37936
1.43772	1.44262	1.41746	1.43078	1.43716	1.43009	1.42615	1.42998	1.43218	1.42434	1.43327	1.43218	1.41736	1.42285
1.36294	1.36057	1.36494	1.37176	1.36042	1.37101	1.36518	1.36540	1.36101	1.36310	1.36138	1.36141	1.36419	1.36770
*i*Pr	Li	*n*Bu	NH_2	NMe_2	NO_2	OH	OMe	Ph	SH	SiH_3	SMe	*t*Bu	
1.37115	1.36999	1.37047	1.37332	1.37796	1.37174	1.37063	1.36671	1.37661	1.36971	1.37585	1.36286	1.36510	
1.43143	1.44858	1.43091	1.43063	1.43336	1.41312	1.42749	1.42812	1.43271	1.42743	1.43258	1.43134	1.43734	
1.37629	1.37520	1.37683	1.37886	1.37760	1.38290	1.37460	1.38907	1.37595	1.37780	1.37797	1.38944	1.38763	
1.45779	1.45120	1.45732	1.44287	1.44657	1.45453	1.44503	1.43969	1.45721	1.45157	1.45418	1.45010	1.46928	
1.38509	1.38090	1.38505	1.39098	1.38571	1.39421	1.38592	1.38370	1.38842	1.38555	1.38959	1.37847	1.37332	
1.42699	1.43507	1.42374	1.42422	1.42275	1.41178	1.42101	1.41632	1.42811	1.42097	1.42800	1.42020	1.43327	
1.37748	1.37880	1.38056	1.38005	1.37924	1.37948	1.37630	1.39109	1.37716	1.38009	1.37982	1.39043	1.38842	
1.45556	1.44788	1.44956	1.43829	1.44213	1.45245	1.44129	1.43466	1.45466	1.44663	1.45083	1.44647	1.46842	
1.38627	1.38267	1.39079	1.39341	1.38668	1.39511	1.38784	1.38626	1.38924	1.38875	1.39115	1.38051	1.37418	
1.42590	1.43388	1.42059	1.42289	1.42146	1.41147	1.42001	1.41480	1.42693	1.41802	1.42724	1.41887	1.43297	
1.37804	1.37971	1.38254	1.38095	1.38009	1.37947	1.37687	1.39188	1.37802	1.38116	1.38022	1.39108	1.38794	
1.45473	1.44680	1.44742	1.43673	1.44129	1.45224	1.44022	1.43319	1.45339	1.44483	1.45016	1.44578	1.46885	
1.38686	1.38313	1.39204	1.39434	1.38777	1.39499	1.38840	1.38697	1.38957	1.38990	1.39149	1.38072	1.37379	
1.42592	1.43360	1.41986	1.42244	1.42099	1.41102	1.41958	1.41428	1.42680	1.41722	1.42716	1.41853	1.43288	
1.37815	1.37978	1.38300	1.38105	1.38053	1.37977	1.37710	1.39210	1.37753	1.38124	1.38030	1.39142	1.38836	
1.45452	1.44691	1.44655	1.43628	1.44107	1.45229	1.43981	1.43258	1.45407	1.44438	1.44995	1.44562	1.46885	
1.38701	1.38310	1.39267	1.39456	1.38758	1.39504	1.38861	1.38728	1.38954	1.39007	1.39155	1.38102	1.37388	
1.42556	1.43365	1.41912	1.42215	1.42098	1.41104	1.41948	1.41419	1.42703	1.41665	1.42698	1.41862	1.43327	
1.37815	1.37992	1.38316	1.38138	1.38036	1.37979	1.37719	1.39207	1.37787	1.38183	1.38040	1.39131	1.38809	
1.45445	1.44672	1.44632	1.43596	1.44122	1.45242	1.43967	1.43236	1.45364	1.44410	1.44984	1.44572	1.46867	
1.38698	1.38325	1.39280	1.39480	1.38671	1.39508	1.38866	1.38735	1.38987	1.39056	1.39163	1.38044	1.37389	
1.42567	1.43351	1.41966	1.42216	1.42079	1.41101	1.41923	1.41416	1.42721	1.41679	1.42697	1.41870	1.43301	
1.37818	1.37994	1.38269	1.38129	1.38033	1.37971	1.37732	1.39199	1.37737	1.38118	1.38037	1.39165	1.38796	
1.45449	1.44673	1.44700	1.43595	1.44116	1.45246	1.43944	1.43238	1.45406	1.44427	1.44988	1.44544	1.46879	
1.38710	1.38325	1.39222	1.39484	1.38733	1.39491	1.38877	1.38729	1.38937	1.39012	1.39164	1.38098	1.37369	

1.42554	1.43355	1.41992	1.42213	1.42101	1.41103	1.41935	1.41432	1.42676	1.41655	1.42710	1.41838	1.43319	
1.37815	1.38014	1.38256	1.38127	1.38026	1.37980	1.37728	1.39179	1.37800	1.38180	1.38025	1.39150	1.38807	
1.45452	1.44636	1.44724	1.43611	1.44134	1.45250	1.43942	1.43264	1.45360	1.44418	1.45009	1.44547	1.46885	
1.38698	1.38352	1.39188	1.39479	1.38677	1.39488	1.38880	1.38706	1.38964	1.39038	1.39152	1.38072	1.37385	
1.42567	1.43335	1.41987	1.42235	1.42093	1.41107	1.41918	1.41459	1.42681	1.41724	1.42720	1.41860	1.43296	
1.37804	1.38026	1.38299	1.38104	1.38003	1.37965	1.37734	1.39143	1.37778	1.38090	1.38027	1.39155	1.38803	
1.45471	1.44622	1.44703	1.43652	1.44161	1.45260	1.43932	1.43332	1.45386	1.44458	1.45016	1.44560	1.46861	
1.38676	1.38373	1.39242	1.39458	1.38654	1.39471	1.38873	1.38653	1.38961	1.38963	1.39132	1.38073	1.37389	
1.42582	1.43358	1.42036	1.42269	1.42136	1.41129	1.41951	1.41533	1.42723	1.41756	1.42753	1.41874	1.43323	
1.37801	1.38024	1.38152	1.38046	1.37963	1.37958	1.37703	1.39064	1.37740	1.38094	1.37990	1.39116	1.38844	
1.45501	1.44645	1.44911	1.43782	1.44229	1.45285	1.44007	1.43501	1.45438	1.44548	1.45079	1.44638	1.46846	
1.38664	1.38383	1.39010	1.39393	1.38599	1.39418	1.38820	1.38537	1.38922	1.38888	1.39068	1.38056	1.37429	
1.42680	1.43478	1.42442	1.42413	1.42261	1.41187	1.42016	1.41724	1.42816	1.41990	1.42841	1.41973	1.43297	
1.37638	1.37830	1.37782	1.37809	1.37771	1.37825	1.37569	1.38792	1.37621	1.37836	1.37874	1.38958	1.38767	
1.45749	1.45314	1.45646	1.44279	1.44585	1.45450	1.44381	1.44120	1.45654	1.44943	1.45376	1.45078	1.46973	
1.38477	1.38882	1.38490	1.39160	1.38512	1.39293	1.38706	1.37901	1.38777	1.38684	1.38884	1.37953	1.37279	
1.43367	1.43685	1.43300	1.43302	1.43330	1.42542	1.42647	1.42481	1.43463	1.42969	1.43657	1.42733	1.43531	
1.36488	1.37119	1.36476	1.36356	1.36321	1.35946	1.36434	1.37228	1.36445	1.36408	1.36601	1.37165	1.37140	

Table 4B-ESI. The subsequent CC distances (Å) in the studied screw structures calculated at the B3LYP/6-31G** level

BF_2	BH_2	Br	CBr_3	CCH	CCl_3	CF_3	CH_3	CHO	Cl	CN	COOH	F	H
1.36305	1.36076		1.37163		1.37101	1.36929		1.35884			1.35962		
1.43764	1.44248		1.4309		1.43045	1.42248		1.43442			1.43405		
1.39323	1.40332		1.37229		1.3717	1.37696		1.40144			1.39646		
1.4584	1.45305		1.46211		1.46406	1.45684		1.45066			1.45538		
1.37547	1.37476		1.39279		1.38988	1.38592		1.37933			1.37837		
1.42985	1.43331		1.42662		1.42653	1.41835		1.41866			1.42012		
1.39543	1.4061		1.37464		1.37407	1.37792		1.40467			1.39896		
1.45704	1.45104		1.46138		1.46344	1.45524		1.44819			1.45348		
1.37626	1.37587		1.3931		1.39059	1.38699		1.38119			1.37984		
1.42906	1.43211		1.42618		1.42614	1.41712		1.41739			1.41917		
1.39591	1.40663		1.37473		1.37414	1.37865		1.40552			1.39935		
1.45682	1.45062		1.46148		1.46354	1.45444		1.44778			1.45339		
1.37653	1.3762		1.39318		1.39039	1.38696		1.38149			1.37986		
1.42879	1.43188		1.42608		1.42627	1.41742		1.41717			1.41932		
1.39598	1.40668		1.37488		1.37411	1.3784		1.40557			1.39948		
1.45676	1.45047		1.46148		1.46349	1.4549		1.44763			1.45341		
1.37657	1.37615		1.39318		1.39038	1.38664		1.38118			1.37982		
1.42874	1.43182		1.42621		1.42619	1.41741		1.41727			1.41927		
1.39594	1.40685		1.37458		1.37396	1.37878		1.40611			1.39953		
1.4568	1.45033		1.46172		1.4637	1.45471		1.44754			1.45332		
1.37656	1.37626		1.39281		1.39021	1.38691		1.38153			1.37999		
1.42878	1.43165		1.42641		1.42643	1.41701		1.41687			1.4192		
1.3959	1.40688		1.37477		1.37394	1.37874		1.40593			1.39944		
1.45693	1.45032		1.46169		1.46372	1.45459		1.44744			1.45336		
1.37639	1.37634		1.39308		1.39028	1.38823		1.3816			1.37982		
1.42884	1.43166		1.42625		1.42642	1.41748		1.41698			1.41934		
1.396	1.40683		1.37474		1.37366	1.37889		1.40583			1.39958		
1.45668	1.45029		1.46177		1.46386	1.45437		1.44728			1.45331		
1.37649	1.37624		1.3929		1.39003	1.38646		1.38132			1.37987		
1.42876	1.4317		1.42636		1.42649	1.41677		1.41714			1.41926		
1.39604	1.4069		1.37473		1.37369	1.37888		1.40609			1.39964		
1.45666	1.45026		1.46173		1.46395	1.45457		1.44732			1.45331		
1.37652	1.37632		1.39301		1.39004	1.38732		1.38153			1.37982		

1.42877	1.43165		1.42639		1.4266	1.41752		1.4171			1.41941		
1.396	1.40672		1.37459		1.37356	1.37842		1.40574			1.39951		
1.45674	1.4506		1.46177		1.46385	1.45523		1.44758			1.45345		
1.37649	1.3762		1.3928		1.3898	1.38675		1.38124			1.37988		
1.42901	1.43211		1.42671		1.42673	1.41812		1.41782			1.41987		
1.39507	1.4057		1.37415		1.37345	1.37775		1.40453			1.39842		
1.45792	1.45255		1.46209		1.46384	1.45616		1.45072			1.45548		
1.37639	1.37602		1.39208		1.38957	1.3849		1.38543			1.38293		
1.4308	1.43534		1.43398		1.43285	1.42587		1.41937			1.42129		
1.38074	1.38878		1.36291		1.36359	1.36507		1.38108			1.37713		
*i*Pr	Li	*n*Bu	NH_2	NMe_2	NO_2	OH	OMe	Ph	SH	SiH_3	SMe	*t*Bu	
1.36495	1.37111	1.36484	1.36363	1.36321	1.35965	1.36435		1.37654	1.36395	1.36607	1.37162	1.36506	
1.43379	1.4372	1.43322	1.43316	1.43329	1.42538	1.42653		1.43309	1.42957	1.4366	1.42738	1.43744	
1.38419	1.38823	1.38401	1.39088	1.3851	1.39247	1.3868		1.37556	1.38706	1.38836	1.37953	1.38746	
1.4584	1.45419	1.45817	1.44404	1.44586	1.45514	1.44408		1.45737	1.44942	1.45487	1.45093	1.46924	
1.37614	1.3779	1.3761	1.37735	1.3777	1.37775	1.37554		1.38812	1.37871	1.37831	1.3894	1.3733	
1.42735	1.4357	1.42709	1.42609	1.4226	1.41259	1.42044		1.42777	1.41937	1.4289	1.42021	1.43354	
1.38519	1.38326	1.38463	1.39183	1.38592	1.3937	1.38784		1.37705	1.38849	1.39002	1.38	1.38733	
1.45686	1.44837	1.45643	1.44219	1.44231	1.45318	1.44037		1.45541	1.44575	1.45251	1.44683	1.46948	
1.37691	1.37964	1.37681	1.37691	1.37963	1.37891	1.37687		1.38901	1.38067	1.37931	1.39067	1.37331	
1.42692	1.43493	1.4267	1.42616	1.42136	1.41194	1.41974		1.42684	1.41774	1.42788	1.41929	1.43336	
1.38542	1.38274	1.38471	1.39197	1.38653	1.39433	1.38819		1.3774	1.38934	1.39048	1.38044	1.38761	
1.45676	1.44811	1.45649	1.43876	1.44164	1.45314	1.43993		1.45496	1.44507	1.45216	1.44636	1.46949	
1.37705	1.37958	1.37692	1.37943	1.37998	1.37901	1.377		1.38906	1.38115	1.37951	1.3909	1.37346	
1.4268	1.43504	1.42666	1.42472	1.42099	1.41183	1.41956		1.42676	1.41747	1.42788	1.41915	1.43327	
1.38559	1.38249	1.38425	1.39231	1.38666	1.39446	1.38825		1.37735	1.38939	1.3904	1.38062	1.38764	
1.45657	1.4483	1.45714	1.44107	1.44144	1.45309	1.43982		1.45511	1.4449	1.4522	1.44618	1.4695	
1.37708	1.3794	1.37642	1.3774	1.38009	1.37899	1.37706		1.38911	1.38103	1.37939	1.39093	1.37361	
1.42666	1.43495	1.42697	1.42584	1.42107	1.41178	1.41957		1.42697	1.41741	1.42785	1.41885	1.43347	
1.38584	1.38235	1.3843	1.39208	1.38658	1.39452	1.38822		1.3773	1.38968	1.39076	1.38076	1.3878	
1.45633	1.44849	1.45726	1.43838	1.44137	1.45301	1.43988		1.45533	1.44476	1.45193	1.44604	1.46933	
1.37719	1.37934	1.37618	1.37967	1.38014	1.37902	1.377		1.38907	1.38116	1.37963	1.39105	1.37376	
1.42666	1.43511	1.42693	1.42457	1.42092	1.41174	1.41962		1.42682	1.41733	1.4276	1.41908	1.43306	
1.38577	1.38223	1.38491	1.39226	1.38666	1.39457	1.38822		1.37733	1.38973	1.3906	1.38051	1.38831	
1.45628	1.44856	1.45676	1.44109	1.44123	1.45299	1.44002		1.45516	1.44481	1.45202	1.44605	1.46859	
1.3772	1.37923	1.37694	1.37733	1.38041	1.37906	1.37696		1.38901	1.38118	1.37953	1.39088	1.37415	

1.42652	1.43512	1.42658	1.42592	1.42092	1.4117	1.41975		1.42709	1.41725	1.4278	1.41886	1.43294	
1.38599	1.38224	1.3845	1.39196	1.38783	1.39455	1.38804		1.3772	1.38965	1.39058	1.38073	1.38792	
1.45608	1.4484	1.45698	1.43864	1.44104	1.45293	1.44031		1.45551	1.44498	1.45206	1.4461	1.46857	
1.37741	1.37927	1.37654	1.37958	1.38058	1.37896	1.37681		1.38898	1.38125	1.37948	1.39095	1.37394	
1.42653	1.43499	1.42694	1.42479	1.42098	1.41191	1.41992		1.42711	1.41771	1.42772	1.41907	1.43312	
1.38568	1.38225	1.38444	1.39174	1.38797	1.39466	1.3878		1.37721	1.38896	1.39068	1.38059	1.3878	
1.45634	1.44836	1.45699	1.4418	1.44127	1.45286	1.44073		1.45546	1.44563	1.45208	1.44626	1.4688	
1.37715	1.37927	1.37652	1.37696	1.38019	1.37875	1.37661		1.38891	1.38045	1.37947	1.39082	1.37382	
1.42665	1.43514	1.42681	1.42629	1.42145	1.41201	1.42021		1.42709	1.41871	1.42786	1.41932	1.4332	
1.38564	1.38208	1.38451	1.39111	1.38668	1.39479	1.38735		1.3772	1.38835	1.39045	1.38016	1.38753	
1.45651	1.44882	1.45702	1.44011	1.44209	1.45268	1.44164		1.45552	1.4469	1.45249	1.4469	1.46939	
1.37701	1.37857	1.37643	1.37909	1.37933	1.37883	1.37613		1.38871	1.37997	1.37929	1.38986	1.37359	
1.42714	1.43617	1.42737	1.42574	1.42264	1.41229	1.4213		1.42788	1.42111	1.42835	1.42045	1.43319	
1.38476	1.38037	1.38368	1.38876	1.38611	1.3942	1.38557		1.37612	1.38514	1.38909	1.37869	1.38761	
1.45823	1.45199	1.45861	1.44736	1.44653	1.45424	1.44543		1.45753	1.45157	1.45509	1.45047	1.46971	
1.37668	1.3751	1.37605	1.37524	1.3777	1.3828	1.37435		1.38746	1.37763	1.37749	1.38918	1.37264	
1.43145	1.44919	1.43114	1.43328	1.43336	1.4128	1.42754		1.43465	1.42774	1.43285	1.43122	1.43556	
1.37056	1.36955	1.3705	1.37264	1.37794	1.37184	1.37065		1.36453	1.36961	1.37586	1.36313	1.37131	

Table 4C-ESI. The subsequent CC distances (Å) in the studied spring structures calculated at the B3LYP/6-31G** level

BF_2	BH_2	Br	CBr_3	CCH	CCl_3	CF_3	CH_3	CHO	Cl	CN	COOH	F	H
1.36245	1.38724	1.36526	1.36237	1.36171	1.36322	1.36502	1.36553	1.35903	1.36455	1.36254	1.37567	1.36659	1.36817
1.43766	1.43708	1.42125	1.43467	1.43634	1.43312	1.42607	1.4307	1.43517	1.42449	1.43233	1.4259	1.4166	1.42277
1.39491	1.37427	1.37805	1.39226	1.3961	1.3894	1.3857	1.38503	1.40625	1.38159	1.39423	1.37723	1.38012	1.37939
1.4554	1.45061	1.44983	1.45944	1.44742	1.46309	1.45433	1.45314	1.44742	1.45019	1.44855	1.45322	1.44282	1.44972
1.37328	1.40408	1.38097	1.3698	1.37549	1.37116	1.37587	1.37758	1.37619	1.37632	1.37661	1.39807	1.38156	1.38027
1.42987	1.43373	1.41621	1.42744	1.4275	1.42792	1.41871	1.42466	1.42371	1.41761	1.42392	1.42483	1.40769	1.41491
1.39661	1.37429	1.37827	1.39412	1.39885	1.3901	1.38789	1.38644	1.40873	1.383	1.39655	1.3749	1.38175	1.38168
1.45327	1.44858	1.44713	1.45714	1.44371	1.463	1.45201	1.45084	1.44265	1.44756	1.44511	1.45046	1.4379	1.44614
1.37438	1.40491	1.38283	1.37006	1.37787	1.37121	1.37718	1.37884	1.37845	1.37772	1.37875	1.39887	1.38388	1.3822
1.42911	1.43339	1.41521	1.42702	1.42575	1.42772	1.41772	1.42366	1.42151	1.41662	1.42235	1.42424	1.40617	1.41329
1.39745	1.37437	1.37847	1.39628	1.39956	1.39053	1.38808	1.38701	1.41012	1.38367	1.39752	1.37571	1.38284	1.38249
1.45277	1.44842	1.44644	1.45483	1.44283	1.46262	1.45168	1.45027	1.44154	1.44696	1.44422	1.45074	1.4368	1.44521
1.37466	1.40503	1.38301	1.37044	1.37828	1.37127	1.37725	1.37918	1.37914	1.37798	1.37934	1.4003	1.38427	1.38267
1.42874	1.43331	1.41493	1.42616	1.42542	1.42768	1.41752	1.42342	1.42094	1.41631	1.42196	1.4247	1.40576	1.41285
1.39738	1.37441	1.37892	1.39567	1.39987	1.3904	1.38866	1.38715	1.41007	1.38397	1.39774	1.37563	1.383	1.38272
1.45262	1.44848	1.44631	1.45408	1.4427	1.46268	1.45128	1.45017	1.44125	1.44664	1.44396	1.45136	1.43652	1.44498
1.37475	1.40489	1.38315	1.37048	1.37836	1.37121	1.37737	1.37926	1.37913	1.37826	1.37928	1.4006	1.38447	1.38283
1.42875	1.43334	1.38315	1.42595	1.42531	1.42792	1.41723	1.42334	1.42096	1.41617	1.4218	1.42474	1.40566	1.4127
1.39762	1.37438	1.37902	1.39539	1.39985	1.39068	1.3891	1.38734	1.41067	1.38416	1.39791	1.37521	1.38313	1.38278
1.45254	1.4485	1.44605	1.4543	1.44259	1.46241	1.4511	1.44991	1.44123	1.44644	1.44377	1.45155	1.43643	1.44491
1.37475	1.40498	1.38325	1.37057	1.37828	1.37131	1.37777	1.37943	1.37942	1.37847	1.37928	1.40006	1.38444	1.3828
1.42862	1.43337	1.4147	1.42598	1.42526	1.42764	1.41724	1.42325	1.42076	1.41614	1.42168	1.42469	1.40565	1.41269
1.39752	1.37445	1.37924	1.39555	1.40016	1.39114	1.38903	1.3874	1.41011	1.3841	1.39829	1.37524	1.38309	1.38279
1.45248	1.44854	1.44606	1.45444	1.44246	1.46199	1.45102	1.44989	1.44118	1.44651	1.44354	1.45167	1.43647	1.44492
1.37474	1.40492	1.38348	1.37053	1.37844	1.37159	1.37774	1.37957	1.37911	1.3785	1.37952	1.40016	1.38443	1.38277
1.42861	1.43347	1.41469	1.42626	1.42518	1.42771	1.41713	1.42326	1.42092	1.41611	1.42153	1.42458	1.40572	1.41272
1.39746	1.37448	1.37901	1.39558	1.40018	1.39065	1.38884	1.3872	1.41062	1.38385	1.39843	1.37519	1.38296	1.38281
1.45249	1.44855	1.44611	1.45443	1.44234	1.46231	1.4512	1.44996	1.44124	1.44676	1.44329	1.45103	1.43662	1.445
1.37471	1.40501	1.38342	1.3705	1.37851	1.37139	1.37755	1.37938	1.37932	1.37818	1.37957	1.39921	1.38431	1.3827
1.42866	1.43361	1.41481	1.42599	1.42522	1.42778	1.41734	1.42337	1.42079	1.41629	1.42143	1.42436	1.40589	1.41287
1.39753	1.37437	1.3788	1.39483	1.40016	1.39036	1.38851	1.38713	1.41011	1.38368	1.39853	1.37488	1.3828	1.38265
1.45252	1.44873	1.44633	1.45508	1.44256	1.46264	1.45128	1.45028	1.44126	1.44703	1.44327	1.45119	1.43701	1.44523
1.37465	1.40482	1.38305	1.37068	1.37838	1.3712	1.37729	1.37917	1.37885	1.37784	1.37941	1.39953	1.38381	1.38248

1.42867	1.43388	1.41514	1.42612	1.42554	1.42771	1.41758	1.4237	1.42112	1.41671	1.42164	1.42491	1.40641	1.4133
1.39717	1.37417	1.37848	1.39481	1.39966	1.39068	1.38859	1.38672	1.41028	1.38329	1.39859	1.37512	1.38209	1.3822
1.4528	1.44917	1.44691	1.45565	1.44348	1.46249	1.45169	1.45078	1.4418	1.44764	1.44399	1.45167	1.43816	1.44614
1.37436	1.40444	1.38253	1.37012	1.37791	1.37115	1.377	1.37857	1.37857	1.37738	1.37943	1.39861	1.38276	1.38167
1.42929	1.43477	1.41622	1.42707	1.42665	1.42791	1.41823	1.42469	1.4221	1.41764	1.42267	1.42557	1.40834	1.41491
1.39637	1.373	1.37674	1.39431	1.39714	1.39056	1.38695	1.38533	1.40816	1.38191	1.39594	1.37324	1.37998	1.38027
1.45482	1.4513	1.44978	1.45579	1.44722	1.46277	1.45409	1.45316	1.44608	1.45022	1.44751	1.4538	1.44298	1.44972
1.37508	1.40291	1.38085	1.36906	1.37697	1.36997	1.37581	1.37727	1.38224	1.37703	1.37841	1.39647	1.38051	1.37939
1.43092	1.4421	1.42373	1.42968	1.4326	1.43101	1.42368	1.43092	1.42319	1.42252	1.42868	1.43425	1.41768	1.42277
1.37984	1.36092	1.36449	1.3719	1.37994	1.37108	1.36875	1.36989	1.379	1.36661	1.37898	1.36048	1.36456	1.36817
*i*Pr	Li	*n*Bu	NH_2	NMe_2	NO_2	OH	OMe	Ph	SH	SiH_3	SMe	*t*Bu	
1.37104	1.37103	1.3649	1.373	1.36504	1.37036	1.36644	1.36749	1.36442	1.3645	1.36614	1.36315	1.37145	
1.43298	1.43621	1.43342	1.43303	1.43378	1.41764	1.42507	1.42748	1.43436	1.43087	1.43593	1.43303	1.43632	
1.3753	1.38857	1.38374	1.37992	1.39452	1.37576	1.38716	1.3917	1.38833	1.3849	1.38805	1.38815	1.37208	
1.45782	1.46081	1.45782	1.44588	1.44827	1.45521	1.44197	1.44196	1.45627	1.4493	1.45502	1.45184	1.47002	
1.38457	1.3988	1.37532	1.38805	1.37946	1.39199	1.38206	1.3836	1.37483	1.37647	1.37751	1.37415	1.3876	
1.42866	1.43478	1.42856	1.42642	1.42496	1.41729	1.41608	1.41821	1.42773	1.42479	1.42986	1.42661	1.43426	
1.37605	1.38758	1.38436	1.38174	1.3967	1.37301	1.38861	1.39347	1.39004	1.38576	1.38927	1.38915	1.37279	
1.45658	1.46277	1.45709	1.4428	1.44426	1.45406	1.43633	1.43589	1.45433	1.44699	1.45327	1.44884	1.4703	
1.38532	1.39819	1.37563	1.38982	1.38167	1.39265	1.38455	1.38637	1.37583	1.37757	1.37838	1.37555	1.3877	
1.42821	1.43451	1.42806	1.42535	1.42356	1.41699	1.41447	1.41636	1.42708	1.42394	1.42913	1.42581	1.43402	
1.37624	1.38726	1.38501	1.38234	1.39743	1.37311	1.38971	1.39473	1.39036	1.38622	1.38958	1.38926	1.37268	
1.45637	1.46388	1.45656	1.44199	1.44341	1.45415	1.43483	1.4343	1.45404	1.44665	1.45305	1.44893	1.47032	
1.38541	1.3975	1.37597	1.39033	1.38203	1.39259	1.38524	1.38718	1.37599	1.37774	1.37844	1.3753	1.38754	
1.42801	1.43441	1.42803	1.42505	1.4232	1.41691	1.41389	1.41576	1.42693	1.42375	1.42898	1.4255	1.43422	
1.37621	1.38711	1.38534	1.38263	1.39755	1.37303	1.39	1.39499	1.39041	1.38633	1.3897	1.38983	1.3726	
1.45642	1.46418	1.45588	1.44154	1.44317	1.45428	1.43432	1.4337	1.45384	1.44658	1.45299	1.44854	1.47023	
1.38544	1.39732	1.37631	1.39049	1.38213	1.39248	1.38551	1.38742	1.37608	1.37774	1.37849	1.37571	1.38738	
1.42794	1.43436	1.42799	1.42499	1.42302	1.41689	1.41378	1.41565	1.42682	1.42374	1.42893	1.42561	1.43437	
1.37611	1.387	1.38488	1.38275	1.39764	1.3731	1.3901	1.39522	1.39051	1.38639	1.38974	1.38998	1.37255	
1.45653	1.46409	1.45656	1.44134	1.44289	1.45449	1.43409	1.43346	1.45378	1.44644	1.45298	1.44784	1.47052	
1.38539	1.39731	1.37592	1.39067	1.38233	1.39249	1.3856	1.38761	1.37615	1.37783	1.37848	1.37607	1.38745	
1.42799	1.43439	1.42817	1.425	1.42295	1.417	1.41366	1.41543	1.42677	1.42374	1.42894	1.42558	1.43425	
1.37622	1.38683	1.38409	1.38263	1.39755	1.37296	1.39007	1.39516	1.39058	1.38641	1.38977	1.38988	1.37271	
1.45641	1.46407	1.45764	1.44146	1.44282	1.45463	1.43417	1.43356	1.45367	1.44646	1.4529	1.44857	1.47059	
1.38533	1.39726	1.37538	1.39061	1.38243	1.39228	1.38552	1.38743	1.37614	1.37793	1.37849	1.37612	1.38747	

1.42809	1.43432	1.42834	1.42514	1.42312	1.4171	1.41367	1.41532	1.42673	1.42372	1.42889	1.42529	1.43434	
1.37642	1.3868	1.38437	1.38245	1.39774	1.37284	1.38987	1.39503	1.39064	1.38625	1.38982	1.38982	1.37267	
1.45608	1.46378	1.45749	1.44178	1.44292	1.45482	1.43445	1.43385	1.45372	1.44661	1.45294	1.44827	1.47047	
1.38557	1.39735	1.37541	1.39059	1.38233	1.39192	1.38533	1.3873	1.37613	1.37776	1.37848	1.37578	1.38745	
1.42801	1.4342	1.42837	1.42525	1.42323	1.41716	1.41384	1.41557	1.42676	1.42383	1.4289	1.42545	1.43417	
1.37642	1.38672	1.38442	1.38222	1.39728	1.37292	1.38958	1.3947	1.39056	1.38614	1.38977	1.38961	1.37263	
1.45621	1.46344	1.45728	1.44212	1.4433	1.455	1.43501	1.43447	1.45386	1.44682	1.45296	1.44854	1.47077	
1.38554	1.39741	1.37561	1.3904	1.38186	1.39172	1.38484	1.38678	1.37603	1.37755	1.37837	1.37561	1.3876	
1.42803	1.43411	1.42841	1.42551	1.42349	1.41724	1.41437	1.41599	1.42703	1.42405	1.429	1.42576	1.43419	
1.37613	1.38652	1.3845	1.38188	1.39669	1.37278	1.38871	1.39377	1.3903	1.38588	1.38962	1.3894	1.37268	
1.45663	1.46332	1.45731	1.44282	1.4443	1.45528	1.4365	1.4361	1.45417	1.44721	1.45331	1.44901	1.47026	
1.38511	1.39702	1.37546	1.38987	1.38126	1.39137	1.38354	1.38534	1.37557	1.3772	1.37817	1.37524	1.38734	
1.42853	1.43466	1.42878	1.42642	1.42516	1.41774	1.41643	1.41809	1.42769	1.42485	1.42954	1.42644	1.43436	
1.37557	1.38583	1.38375	1.38028	1.39472	1.37188	1.38622	1.39108	1.38952	1.38442	1.38853	1.38811	1.37127	
1.4578	1.46571	1.45839	1.44581	1.44883	1.45641	1.44182	1.44179	1.4561	1.44953	1.45527	1.45082	1.4716	
1.38422	1.394	1.37539	1.38906	1.37703	1.38967	1.38179	1.38387	1.37444	1.3763	1.37679	1.37541	1.3836	
1.43356	1.44673	1.43221	1.43189	1.43525	1.42526	1.42588	1.42732	1.43283	1.42928	1.43426	1.43076	1.44051	
1.365	1.3768	1.37055	1.36478	1.3786	1.36069	1.36915	1.37229	1.37676	1.37012	1.37552	1.37174	1.36254	

Table 5ESI. Population of the σ- and π- valence orbitals in 3-substituted thiophenes and substituent effect descriptors based on the thiophene ring C-atoms based on NBO populations calculated from B3LYP/6-31G** optimized structures.

Substituent	population of valence orbitals		$sEDA_T$	$pEDA_T$	$(s+p)EDA_T$
	sigma	pi			
BF_2	13.24416	4.29465	0.181	-0.051	0.130
BH_2	13.21942	4.23218	0.156	-0.114	0.043
Br	12.88661	4.40015	-0.176	0.054	-0.122
CBr_3	12.87874	4.32028	-0.184	-0.026	-0.210
CCH	12.86739	4.34119	-0.196	-0.005	-0.200
CCl_3	12.87137	4.32561	-0.192	-0.020	-0.212
CF_3	12.93227	4.33546	-0.131	-0.010	-0.141
CH_3	12.84201	4.34636	-0.221	0.000	-0.221
CHO-1	12.95171	4.28908	-0.111	-0.057	-0.168
CHO-2	12.95640	4.27443	-0.107	-0.071	-0.178
Cl	12.81577	4.40524	-0.247	0.059	-0.188
CN	12.90128	4.33436	-0.162	-0.012	-0.173
COOH-1	12.94267	4.29709	-0.120	-0.049	-0.169
COOH-2	12.94239	4.29653	-0.121	-0.049	-0.170
F	12.45335	4.40832	-0.610	0.062	-0.547
H	13.06300	4.34593	0.000	0.000	0.000
iPr	12.83910	4.34218	-0.224	-0.004	-0.228
Li	13.51715	4.30231	0.454	-0.044	0.411
nBu	12.83696	4.34519	-0.226	-0.001	-0.227
NH_2	12.64319	4.43925	-0.420	0.093	-0.326
NMe_2	12.63290	4.43857	-0.430	0.093	-0.337
NO2	12.73194	4.29979	-0.331	-0.046	-0.377
OH-1	12.52177	4.44049	-0.541	0.095	-0.447
OH-2	12.52809	4.43020	-0.535	0.084	-0.451
OMe-1	12.52083	4.44340	-0.542	0.097	-0.445
OMe-2	12.52789	4.43082	-0.535	0.085	-0.450
Ph	12.83674	4.34137	-0.226	-0.005	-0.231
SH-1	12.96271	4.37009	-0.100	0.024	-0.076
SH-2	12.93700	4.42259	-0.126	0.077	-0.049
SiH3	13.25088	4.33657	0.188	-0.009	0.179
SMe-1	12.95514	4.43078	-0.108	0.085	-0.023
SMe-2	12.98536	4.36267	-0.078	0.017	-0.061
tBu	12.83414	4.35164	-0.229	0.006	-0.223

Table 6ESI. Volume of 3-substituted thiophenes and pure substituents estimated based on cavity volume or surface calculated assuming the scrf=(iefpcm,solvent=water,read) key and PCMDoc PDens=1.0 as the "read" parameters using the single point at the 6-31G** calculations of the structures optimized in vacuum. Index "rel" means that unsubstituted and thiophene with abstracted hydrogen in position 3 was subtracted. L_{subst} denotes length of the substituent from the ipso C-atom to the most distanced atom of the substituent or the position of the face defined by atoms as in CF_3 or CBr_3 groups. W is the substituent width calculated from the cavity, and surface, assuming that the substituent volume is equal to the volume of a cylinder, or a cuboid or with one dimension equal to two radii of the bulkiest atom of the substituent.

Substituent	3-subst thiophene		sole substituent						
	C_{vol}	C_{surf}	$C_{vol/rel}$	$C_{surf/rel}$	L_{subst}	$W_{rel-cav}$	$W_{rel-surf}$	$W_{bulkiest-at}$	$W_{rel/at}$
	Å^3	Å^2	Å^3	Å^2	Å	Å^2	Å^2	Å	Å
bf2	159.6	165.8	42.2	39.9	2.23079	4.9	17.9	0.8141	22.0
bh2	149.5	157.1	32.1	31.2	2.13122	4.4	14.7	0.8141	18.0
br	150.9	157	33.5	31.1	1.89861	4.7	16.4	1.0305	15.9
cbr3	233.5	221.3	116.1	95.4	2.20429	8.2	43.3	1.0305	42.0
cch	156.3	163.8	38.9	37.9	3.69828	3.7	10.3	0.6513	15.8
ccl3	211.6	206.6	94.2	80.7	2.14666	7.5	37.6	0.7807	48.2
cf3	166.7	171.6	49.3	45.7	1.99203	5.6	23.0	0.6513	35.3
ch3	146.4	155.5	29.0	29.6	1.90155	4.4	15.6	0.6513	23.9
cho-1	151.6	160.1	34.2	34.2	2.04784	4.6	16.7	0.6513	25.7
cho-2	151.1	157.8	33.7	31.9	2.04784	4.6	15.6	0.6513	23.9
cl	143.6	151.5	26.2	25.6	1.74755	4.4	14.7	0.7807	18.8
cn	150.0	157.9	32.6	32.0	2.59015	4.0	12.4	0.6513	19.0
cooh-1	162.3	167	44.9	41.1	2.56628	4.7	16.0	0.6513	24.6
cooh-2	162.3	167.6	44.9	41.7	2.56628	4.7	16.3	0.6513	25.0
f	127.8	137	10.4	11.1	1.34421	3.1	8.3	0.4071	20.4
h	120.8	130.8	3.4	4.9	1.08390	2.0	4.6	0.5292	8.6
ipr	196.1	197.4	78.7	71.5	3.22870	5.6	22.2	0.6513	34.0
li	124.4	136.1	7.0	10.2	1.95828	2.1	5.2	1.6282	3.2
nbu	223.3	226	105.9	100.1	6.02258	4.7	16.6	0.6513	25.5
nh2	140.3	149.2	22.9	23.3	1.85288	4.0	12.6	0.5427	23.2
nme	187.5	187.1	70.1	61.2	3.13000	5.3	19.6	0.6513	30.0
no2	156.3	161.3	38.9	35.4	2.01781	5.0	17.6	0.5427	32.4
oh-1	133.6	142.1	16.2	16.2	1.91181	3.3	8.5	0.4652	18.3
oh-2	133.7	142.1	16.3	16.2	1.91181	3.3	8.5	0.4652	18.3
ome-1	159.4	164.5	42.0	38.6	3.25775	4.1	11.9	0.6513	18.2
ome-2	159.1	165.9	41.7	40.0	3.25775	4.0	12.3	0.6513	18.9
ph	223.4	215.6	106.0	89.7	5.38096	5.0	16.7	0.6513	25.6
sh-1	149.7	157.0	32.3	31.1	2.37628	4.2	13.1	0.8738	15.0
sh-2	149.8	157.0	32.4	31.1	2.37628	4.2	13.1	0.8738	15.0
sih3	164.3	170.0	46.9	44.1	2.38643	5.0	18.5	1.1476	16.1
sme-1	175.2	178.3	57.8	52.4	3.73153	4.4	14.1	0.8738	16.1
sme-2	176.0	180.4	58.6	54.5	3.73153	4.5	14.6	0.8738	16.7
tbu	217.3	210.9	99.9	85.0	3.15483	6.4	27.0	0.6513	41.4

Table 7ESI. Parameters of helix fitted using HELFIT program[1] based on coordinates of eleven S-atoms of each screw and spring conformers of the substituted undecathiophenes. The helix equation is expressed as follows: $x=a\cdot\cos(t)$; $y=a\cdot\sin(t)$; $z=b\cdot t$, where a is the helix radius and pitch is defined as $2\cdot\pi\cdot b$, a and pitch are in angstroms. Number of thiophene rings per turn is denoted as NTRT. Bond length alternation (BLA), defined as the difference between the average long and short C-C distances in the conjugated π system.

Substituent	belt	screw				spring			
	BLA	Pitch	NTRT	a	BLA	Pitch	NTRT	a	BLA
BF_2	0.057	9.77	2.51	0.92	0.058	22.25	7.92	2.23	0.057
BH_2	0.051	9.33	2.40	0.95	0.052	23.26	7.84	2.01	0.054
Br	0.047					21.48	9.32	3.49	0.050
CBr_3	0.059	10.48	2.71	0.87	0.062	22.26	7.10	1.31	0.060
CCH	0.047					17.37	10.63	4.85	0.048
CCl_3	0.062	10.85	2.81	0.83	0.064	20.08	6.08	0.56	0.065
CF_3	0.053	9.67	2.47	0.93	0.055	23.05	8.45	2.51	0.053
CH_3	0.048					24.08	8.40	2.32	0.056
CHO	0.056	9.72	2.50	0.95	0.042	6.07	10.27	5.16	0.040
Cl	0.047					22.76	9.05	3.09	0.053
CN	0.046					18.04	10.6	4.78	0.047
COOH	0.059	8.86	2.27	1.00	0.049	16.01	9.44	4.09	0.053
F	0.043					1.64	13.07	6.99	0.041
H	0.048					25.08	10.71	4.17	0.063
*i*Pr	0.060	9.88	2.54	0.94	0.062	22.21	6.87	1.24	0.059
Li	0.061	9.29	2.37	1.04	0.063	25.79	7.33	0.84	0.064
*n*Bu	0.051	10.01	2.57	0.92	0.063	21.92	6.63	1.05	0.050
NH_2	0.045	9.08	2.47	1.01	0.051	25.70	9.07	2.65	0.047
NMe_2	0.051					20.71	10.05	4.16	0.055
NO_2	0.047	9.01	2.30	0.98	0.048	19.59	6.94	1.79	0.041
OH	0.049	9.14	2.34	1.01	0.050	2.17	12.48	6.61	0.038
OMe	0.039					3.14	12.10	6.36	0.059
Ph	0.059	9.66	2.49	0.93	0.060	23.44	7.81	1.9	0.055
SH	0.049	8.61	2.22	1.01	0.050	24.09	8.44	2.38	0.059
SiH_3	0.055	9.47	2.43	0.96	0.057	23.77	7.77	1.81	0.056
SMe	0.049	8.46	2.15	1.02	0.050	23.25	8.50	2.51	0.073
*t*Bu	0.071	11.90	3.14	0.73	0.071	15.41	4.34	0.2	0.057

(1) P. Enkhbayar, S. Damdinsuren, M. Osaki and N. Matsushima, HELFIT: Helix fitting by a total least squares method, *Comp. Biol. Chem*., 2008, **32**, 307–310.

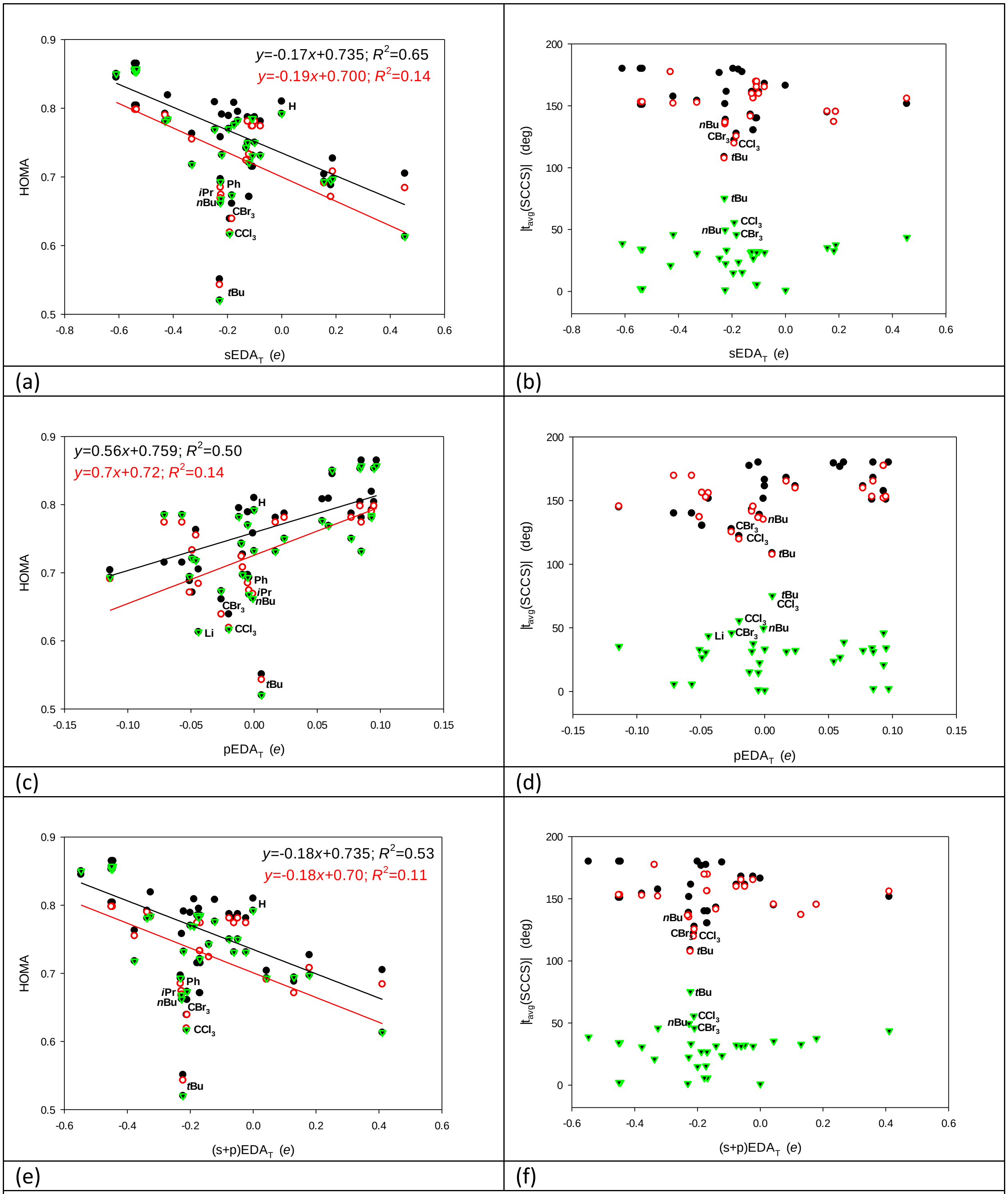

Fig. 1ESI. Correlations between substituent effect descriptors sEDAT (a, b), pEDAT (c,d) and (s+p)EDAT and HOMA index (a, c, e) and averaged module of the torsion SCCS angle (b, d, f) of the belt (black), screw (red) and spring (green) conformations of eleven substituted undecathiophenes

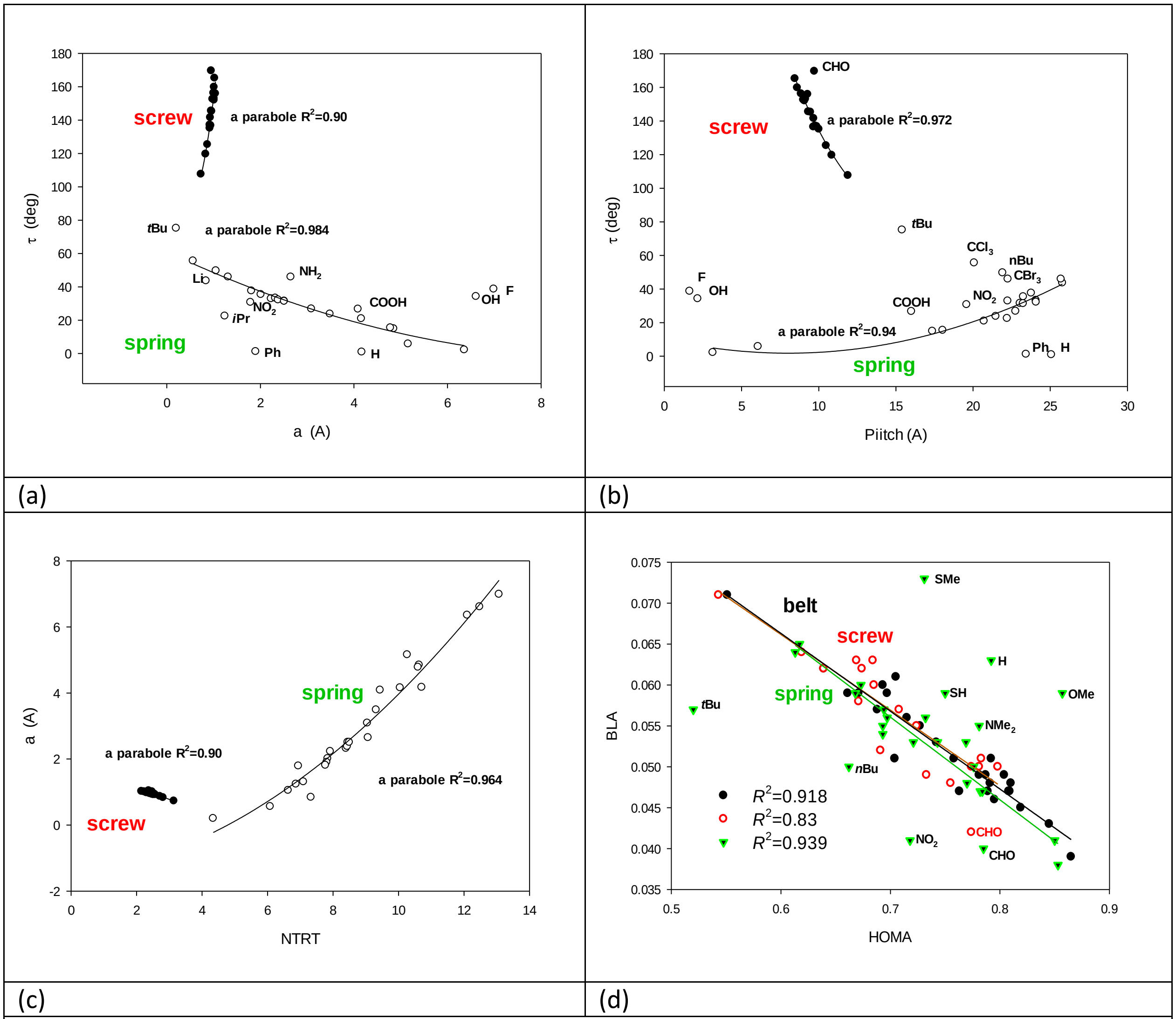

Fig. 2ESI. Correlations between the averaged module of the torsion SCCS angle and helix radius (a) and helix pitch (b) and between the helix radius and number of thiophene rings per turn NTRT (c) for screw and spring undecathiophenes. Linear correlations between HOMA index and the bond length alternation BLA (d). The spread of points have the same reasons as the spread of points in figures presented in the main text.

BELTS XYZ

BF_2			
C	16.37615100	7.70657000	-0.74432800
C	16.57137500	7.24850100	0.52439600
C	16.03269900	5.93739600	0.76498700
C	15.41234000	5.42167200	-0.37127000
C	14.72247600	4.16143000	-0.61548400
C	14.77050000	3.39016200	-1.75345400
C	13.93739700	2.22828500	-1.73393800
C	13.21944500	2.13413100	-0.54044300
C	12.25981600	1.15189600	-0.05815400
C	12.12194900	0.68375700	1.22894000
C	11.04558400	-0.23524100	1.42713000
C	10.33238500	-0.45061400	0.24591100
C	9.17666800	-1.28039700	-0.05814100
C	8.93403800	-1.97053200	-1.22492500
C	7.67871400	-2.65037300	-1.27673700
C	6.93867700	-2.44737400	-0.11007500
C	5.62573800	-2.91952300	0.30242600
C	5.23793400	-3.30590500	1.56570200
C	3.86086900	-3.66542700	1.69264700
C	3.18080600	-3.52275700	0.48101800
C	1.79384800	-3.75576900	0.10911600
C	1.32594100	-4.24709500	-1.08936700
C	-0.09656700	-4.31020600	-1.20388000
C	-0.72420600	-3.82749400	-0.05349300
C	-2.12794600	-3.68921500	0.30266700
C	-2.69607700	-3.88904700	1.54079300
C	-4.09727700	-3.61969700	1.61468400
C	-4.59906400	-3.17855700	0.38796400
C	-5.93457700	-2.78598000	-0.03423600
C	-6.51523700	-3.00622300	-1.26355600
C	-7.82214500	-2.45242500	-1.42399600
C	-8.23325500	-1.77063500	-0.27654500
C	-9.45856700	-1.05449900	0.04296200
C	-10.10714100	-1.01739000	1.25687800
C	-11.26338800	-0.17922500	1.30050800
C	-11.48072900	0.45696000	0.07620700
C	-12.50493500	1.38934300	-0.36887100
C	-13.07697900	1.46015000	-1.61960000
C	-14.01665600	2.52198300	-1.79568500
C	-14.14103800	3.29247300	-0.63873000
C	-14.95752700	4.45865300	-0.32747100
C	-15.59953000	4.73036900	0.85999600
C	-16.25403700	6.00198300	0.89642500
C	-16.07768200	6.68789200	-0.28874600

S	15.49574800	6.55887100	-1.69833300
S	13.57445800	3.47613000	0.51450100
S	10.99302100	0.48773700	-1.06771000
S	7.79629400	-1.42243600	1.01006000
S	4.24461800	-2.94230500	-0.77327800
S	0.44461200	-3.30094500	1.12841000
S	-3.33825900	-3.09548900	-0.81443500
S	-7.00337700	-1.81763600	0.95879300
S	-10.25705800	0.01879100	-1.08690600
S	-13.09751400	2.70233000	0.62645900
S	-15.14322500	5.81200200	-1.43415600
H	16.69221400	8.64496400	-1.17838000
H	17.10150700	7.81022200	1.28440500
H	15.42403700	3.62474700	-2.58577700
H	12.80755200	0.95869600	2.02226300
H	9.66180700	-2.02871500	-2.02616300
H	5.93706100	-3.37522100	2.39122700
H	1.98962200	-4.60133900	-1.86983400
H	-2.13009000	-4.26688600	2.38456100
H	-6.03386100	-3.59554900	-2.03559500
H	-9.78946600	-1.61622300	2.10275600
H	-12.86296500	0.73617900	-2.39755700
H	-15.62293100	4.02612700	1.68390200
H	-16.46641000	7.66446800	-0.54545500
B	-17.06459600	6.55299100	2.08469600
B	-14.82904500	2.62800500	-3.10432500
B	-12.15488600	-0.16683800	2.56073400
B	-8.63507700	-2.75211700	-2.70167700
B	-4.87389000	-3.94327700	2.90906200
B	-0.74294400	-4.97453300	-2.43845900
B	3.33817900	-4.25232700	3.02135500
B	7.34923300	-3.57253000	-2.47011000
B	10.87529900	-0.93689800	2.79132300
B	14.00861000	1.20726000	-2.88857400
B	16.27636200	5.23426000	2.11583900
F	15.94477500	3.97236400	2.36666400
F	16.87192400	5.89826300	3.10368800
F	14.74167000	1.48454600	-3.96378800
F	13.38989000	0.03210500	-2.87892000
F	8.17891500	-3.61938800	-3.50846100
F	6.27861300	-4.35583200	-2.52803500
F	0.02636800	-5.35632100	-3.45381700
F	-2.04362000	-5.21490300	-2.55405300
F	-8.05764700	-3.40604300	-3.70553300
F	-9.90758200	-2.41128300	-2.86702300
F	-14.55489800	1.80387800	-4.11179800
F	-15.81734600	3.49324000	-3.29457900
F	-17.64121200	7.74921900	2.03534700

F	-17.21311800	5.84972100	3.20179700
F	-13.32425800	0.45736600	2.63695000
F	-11.76106300	-0.82907700	3.64487800
F	-6.19498400	-3.86966900	3.01995300
F	-4.20681500	-4.33986000	3.98909300
F	2.11948000	-4.75463700	3.18149600
F	4.14070400	-4.28743600	4.08119700
F	10.00062400	-1.90930400	3.02169000
F	11.64978600	-0.57876200	3.81152300
BH_2			
C	17.83079900	6.13638700	-0.14301100
C	17.82970100	5.58885400	1.10252600
C	16.99375300	4.42208000	1.24735700
C	16.34535300	4.12271800	0.03875100
C	15.40590100	3.07178300	-0.30941100
C	15.24852400	2.44918600	-1.52527400
C	14.20535200	1.46895200	-1.59251100
C	13.53255400	1.37561100	-0.36051900
C	12.41932100	0.54744400	0.05936900
C	12.15572700	0.06582800	1.32147800
C	10.95038300	-0.69550200	1.45751100
C	10.26496200	-0.76325100	0.23042100
C	9.01686200	-1.40380200	-0.13230200
C	8.66127400	-1.92307000	-1.35670900
C	7.33799800	-2.46290500	-1.44290500
C	6.66064000	-2.31440100	-0.21816100
C	5.32082700	-2.69061600	0.18495200
C	4.88831200	-3.03917000	1.44471200
C	3.49068600	-3.32408900	1.56593000
C	2.84053000	-3.15410400	0.32926200
C	1.45121700	-3.31439000	-0.04886400
C	0.94979500	-3.68440900	-1.27651400
C	-0.47757100	-3.72222600	-1.37954300
C	-1.07337900	-3.33357100	-0.16494500
C	-2.46421600	-3.20755100	0.22006900
C	-3.00675400	-3.36668200	1.47550600
C	-4.41615000	-3.13846700	1.57769900
C	-4.94983400	-2.76393600	0.33027600
C	-6.30154600	-2.42765100	-0.06787100
C	-6.88533500	-2.60896000	-1.30153500
C	-8.23305600	-2.14274400	-1.42631600
C	-8.66919400	-1.55750300	-0.22282800
C	-9.93097900	-0.94439200	0.13861400
C	-10.51511100	-0.89178700	1.38427300
C	-11.75331700	-0.17745900	1.46067100
C	-12.09830800	0.35336500	0.20379700
C	-13.23565700	1.14554600	-0.21932100
C	-13.82656600	1.17171600	-1.46260100

C	-14.91753900	2.08705400	-1.61199300
C	-15.13259900	2.80083000	-0.42010300
C	-16.10004800	3.83061700	-0.08558100
C	-16.69449600	4.06621100	1.13322200
C	-17.56478100	5.20711500	1.16980800
C	-17.59207200	5.83042300	-0.07073000
S	16.76924500	5.26812700	-1.21067900
S	14.19002600	2.48178300	0.81362200
S	11.10915400	0.10189000	-1.02437400
S	7.65610100	-1.52408200	0.97382400
S	3.95125800	-2.65283300	-0.91631100
S	0.12279600	-2.93646700	1.03837500
S	-3.71921100	-2.70134000	-0.90186600
S	-7.42577300	-1.59608600	0.99726200
S	-10.90570100	-0.03343300	-1.00592900
S	-14.00621500	2.33342900	0.82192400
S	-16.60800900	5.05977900	-1.24468700
H	18.37748100	6.99506600	-0.50752900
H	18.42177300	5.97924300	1.92251300
H	15.89944900	2.65326200	-2.36827900
H	12.83864100	0.21180300	2.15116000
H	9.35340800	-1.96528600	-2.19055900
H	5.56850300	-3.13878000	2.28348800
H	1.59212800	-3.97566400	-2.10038200
H	-2.41538900	-3.69070800	2.32491600
H	-6.37644300	-3.11560100	-2.11425300
H	-10.09122500	-1.39845300	2.24438400
H	-13.51937800	0.50867800	-2.26397900
H	-16.53617000	3.42145900	1.99079000
H	-18.16316000	6.70855600	-0.34339700
B	-18.37304000	5.70464500	2.37156500
H	-19.06190400	6.67479800	2.26461100
H	-18.32156200	5.13210000	3.41872200
B	-15.77093000	2.13195900	-2.88359800
H	-16.76941200	2.77592100	-2.95958000
H	-15.43376500	1.47643000	-3.82390200
B	-12.59164500	-0.15074700	2.74212500
H	-12.13609000	-0.63468900	3.73498100
H	-13.68780600	0.31217700	2.78008600
B	-9.06200100	-2.40207300	-2.68768000
H	-10.22476200	-2.15457300	-2.75188000
H	-8.52487600	-2.89436100	-3.63460200
B	-5.18496700	-3.41337100	2.87341000
H	-4.56807700	-3.70528800	3.85411000
H	-6.37312200	-3.37086300	2.93461300
B	-1.18118000	-4.24982600	-2.63323000
H	-0.51958600	-4.51707900	-3.59157100
H	-2.35763600	-4.42609000	-2.68113000

B	2.88786600	-3.86124800	2.86724600
H	3.58255400	-3.92294900	3.83739000
H	1.76218200	-4.24137800	2.94292800
B	6.84817300	-3.20106300	-2.69190300
H	7.54719500	-3.21420000	-3.66082900
H	5.80932200	-3.78172300	-2.72653500
B	10.59419900	-1.41491900	2.76136600
H	9.67679400	-2.16911100	2.84675800
H	11.27837400	-1.22991500	3.72339200
B	13.99905800	0.59291200	-2.83053100
H	13.23772800	-0.32252500	-2.84802000
H	14.64476200	0.82203200	-3.80981600
B	16.96166000	3.60080000	2.53728000
H	17.55332900	4.00683300	3.49354900
H	16.39026900	2.55875600	2.61906500
Br			
C	-21.62267600	0.01189000	0.07543200
C	-21.16557100	-1.26929300	-0.01135000
C	-19.74315100	-1.33088600	-0.03164900
C	-19.10349200	-0.10832400	0.03834200
C	-17.70581800	0.26734200	0.04487300
C	-17.20553100	1.55162000	0.10078500
C	-15.79451000	1.60708000	0.09797100
C	-15.16136900	0.37959900	0.04040600
C	-13.76890500	-0.00068600	0.01947100
C	-13.27203600	-1.28854700	-0.02474700
C	-11.86221200	-1.34763300	-0.03825900
C	-11.22400900	-0.12110400	-0.00546500
C	-9.83098900	0.25519800	-0.00552400
C	-9.33137200	1.54296900	0.01162200
C	-7.92175800	1.59983200	0.01188300
C	-7.28546300	0.37178600	-0.00465600
C	-5.89294800	-0.00571500	-0.00888600
C	-5.39356100	-1.29378900	-0.01192000
C	-3.98391900	-1.35085400	-0.01761500
C	-3.34734000	-0.12285000	-0.01952400
C	-1.95500700	0.25516400	-0.02512800
C	-1.45685800	1.54358200	-0.04209800
C	-0.04733000	1.60217700	-0.03987800
C	0.59065500	0.37503900	-0.02100900
C	1.98354300	-0.00088400	-0.01193600
C	2.48417200	-1.28807300	0.01709700
C	3.89392900	-1.34390000	0.01823300
C	4.52958200	-0.11577400	-0.00992400
C	5.92165400	0.26337100	-0.01874900
C	6.41900300	1.55148100	-0.05615900
C	7.82868200	1.61107700	-0.05409900
C	8.46760500	0.38503600	-0.01523400

C	9.86085200	0.00972500	-0.00015000
C	10.36198800	-1.27647700	0.04168100
C	11.77220100	-1.33160900	0.04725900
C	12.40736400	-0.10391200	0.01020800
C	13.79995800	0.27655900	0.00233700
C	14.29681700	1.56374800	-0.03325600
C	15.70799800	1.62285800	-0.03345900
C	16.34631900	0.39825300	0.00133600
C	17.74341200	0.01955700	0.01194100
C	18.24096800	-1.26939100	0.03302600
C	19.65770300	-1.31368700	0.04206200
C	20.25440600	-0.08616200	0.02816900
S	-20.32659100	1.15890500	0.13435200
S	-16.38424100	-0.88985300	-0.01471900
S	-12.44183500	1.15329300	0.04643500
S	-8.50575100	-0.90146600	-0.02375800
S	-4.56730000	1.15073900	-0.01181000
S	-0.62810300	-0.89982900	-0.00752900
S	3.30838300	1.15643900	-0.03757000
S	7.24964200	-0.89015700	0.01927600
S	11.18575500	1.16719600	-0.03341300
S	15.12922700	-0.87590000	0.03739800
S	19.07144000	1.17870000	0.00273100
H	-22.64666800	0.35623800	0.10907500
H	-21.79717200	-2.14652600	-0.06006000
H	-17.82116600	2.44090800	0.14278100
H	-13.89061900	-2.17644100	-0.04655900
H	-9.94836200	2.43213600	0.02321600
H	-6.01078400	-2.18287400	-0.00937200
H	-2.07495000	2.43194200	-0.05652200
H	1.86778000	-2.17750100	0.03786600
H	5.80043300	2.43917500	-0.08504100
H	9.74618600	-2.16619100	0.06826500
H	13.67887600	2.45202400	-0.05891500
H	17.62344200	-2.15839800	0.04100900
H	21.30842400	0.14828700	0.02980600
Br	-18.86839300	-3.00759200	-0.14972100
Br	-14.91408000	3.28417600	0.17109100
Br	-10.98580500	-3.02741800	-0.09848100
Br	-7.04288400	3.27939000	0.03401000
Br	-3.10463300	-3.03038900	-0.02169200
Br	0.82955100	3.28291500	-0.06286400
Br	4.77427300	-3.02259100	0.05777800
Br	8.70409800	3.29226100	-0.10406500
Br	12.65245900	-3.01027200	0.10418700
Br	16.58135000	3.30635500	-0.08087600
Br	20.62425300	-2.94667000	0.06952900
CBr_3			

C	-14.66501900	9.30094200	1.63715900
C	-15.13361500	8.98830600	0.39631300
C	-14.94166700	7.61209400	0.04133700
C	-14.29676300	6.89483600	1.04555100
C	-13.87914600	5.50120200	1.18034400
C	-14.13113400	4.69612700	2.26501000
C	-13.54697100	3.39696200	2.19056400
C	-12.80260800	3.22641300	1.02412000
C	-12.04525500	2.11230700	0.46202900
C	-12.11588900	1.68920100	-0.84508900
C	-11.21777700	0.63385900	-1.17887400
C	-10.42111200	0.26534900	-0.09528200
C	-9.38962500	-0.74693700	0.10690600
C	-9.30830500	-1.58053700	1.19858700
C	-8.15969100	-2.42402200	1.21966200
C	-7.33061200	-2.20245600	0.12073200
C	-6.07855000	-2.79712200	-0.33618400
C	-5.82007100	-3.20547200	-1.62410100
C	-4.49242300	-3.67599100	-1.84397100
C	-3.71179000	-3.59124300	-0.69165200
C	-2.33293100	-3.94783900	-0.37320100
C	-1.93499700	-4.62387300	0.75709800
C	-0.52445900	-4.77319300	0.89600400
C	0.16908900	-4.16513900	-0.14931200
C	1.58410300	-4.04186700	-0.48522600
C	2.12650200	-4.27033400	-1.72843300
C	3.52231300	-3.99762500	-1.82770000
C	4.04625600	-3.51750500	-0.62796700
C	5.38060900	-3.11649500	-0.19377000
C	5.96758200	-3.48749400	0.99406100
C	7.23991600	-2.89379200	1.24017400
C	7.61397700	-2.02188400	0.21882300
C	8.79421400	-1.19731200	-0.02070400
C	9.48851700	-1.13188600	-1.20609700
C	10.55945100	-0.19074300	-1.21642400
C	10.65666700	0.50213100	-0.00993900
C	11.56598300	1.52239100	0.50228600
C	12.14177000	1.50594200	1.75200900
C	12.93070200	2.65303900	2.05815600
C	12.92742000	3.58006000	1.01816800
C	13.57567100	4.87474500	0.81870900
C	14.29323100	5.24222300	-0.29221700
C	14.72293100	6.60676600	-0.26964100
C	14.30337600	7.26224900	0.85997400
S	-13.94981100	7.93354500	2.40751200
S	-12.84444500	4.66928900	0.04723400
S	-10.79841900	1.22990200	1.30681100
S	-7.98680600	-0.96022300	-0.91073900

S	-4.63465100	-2.93767700	0.63482400
S	-0.93389800	-3.42748300	-1.27936000
S	2.80676200	-3.41780200	0.59337300
S	6.39179000	-1.95796500	-1.02164700
S	9.42861600	-0.02723300	1.10823300
S	11.95612300	3.01611600	-0.31324000
S	13.39518100	6.24377100	1.91003300
H	-14.70488000	10.25697800	2.13972400
H	-15.61914900	9.70141700	-0.25406500
H	-14.75782000	5.01286400	3.08624300
H	-12.82795500	2.09979100	-1.54659500
H	-10.07965200	-1.61710000	1.95463200
H	-6.58000200	-3.20732400	-2.39249700
H	-2.64231900	-5.04212600	1.45882000
H	1.54474800	-4.66636100	-2.54849000
H	5.51381200	-4.20517100	1.66271500
H	9.26332300	-1.77730900	-2.04311300
H	12.03626800	0.66706000	2.42514500
H	14.53111800	4.55506000	-1.09427500
H	14.49620900	8.28976000	1.12982900
C	15.54984700	7.19567200	-1.36459200
C	13.64992300	2.81331500	3.36760800
C	11.45282600	0.00401500	-2.40820200
C	8.04437500	-3.18939900	2.47379400
C	4.29839300	-4.20804400	-3.09628700
C	0.10005000	-5.49491500	2.05584000
C	-4.02713700	-4.18725500	-3.17738300
C	-7.90321400	-3.41177200	2.32194500
C	-11.16074900	0.02565200	-2.55099900
C	-15.37476100	7.05273400	-1.28336200
C	-13.73090300	2.36630700	3.26707300
Br	-16.66259500	8.22274500	-2.22073700
Br	-16.24982100	5.29201200	-1.12461200
Br	-13.80448600	6.86409600	-2.49528200
Br	-15.19092900	2.81664000	4.51923500
Br	-14.15754300	0.58055200	2.54337300
Br	-12.07690700	2.22041100	4.36739000
Br	-12.71456300	0.50646000	-3.66913300
Br	-9.54897000	0.68121300	-3.52061500
Br	-11.10216500	-1.94634600	-2.50558800
Br	-5.51785400	-4.56454600	-4.41582300
Br	-2.88599700	-2.82277700	-4.07301500
Br	-2.99556400	-5.86289700	-3.02989000
Br	-7.32740900	-5.17329400	1.64601000
Br	-6.49417700	-2.71664900	3.54636000
Br	-9.49634700	-3.74211800	3.44033200
Br	-1.19618700	-6.59478300	3.06049100
Br	0.84556300	-4.18090600	3.35367700

Br	1.54784800	-6.71513300	1.50297800
Br	3.32180500	-5.30105500	-4.41939400
Br	4.66115000	-2.45773700	-3.97426300
Br	6.02056200	-5.12052800	-2.78903500
Br	11.28221000	-1.44392100	-3.73990900
Br	10.97451600	1.69105300	-3.35210400
Br	13.36273300	0.08039100	-1.91986500
Br	9.95293700	-3.49833700	2.08320500
Br	7.90326900	-1.67468000	3.75981600
Br	7.40980800	-4.79430800	3.43339000
Br	13.80279000	1.11512700	4.36624800
Br	12.64656300	4.06783600	4.54550300
Br	15.48564000	3.49499800	3.15147900
Br	14.60961400	7.02015700	-3.10088500
Br	17.26999800	6.21819900	-1.51845000
Br	15.95788200	9.09551100	-1.08709000
CCH			
C	21.26922800	0.07059700	0.00001300
C	20.79331500	1.34505500	0.00000900
C	19.35787700	1.41543000	0.00000500
C	18.75645500	0.15615600	0.00000700
C	17.36124500	-0.22280000	0.00000500
C	16.84962800	-1.50164100	0.00000500
C	15.42663400	-1.57090200	0.00000300
C	14.82675600	-0.30887000	0.00000100
C	13.43464700	0.06846400	0.00000000
C	12.92196200	1.34870900	-0.00000100
C	11.50016600	1.41785500	-0.00000300
C	10.89922600	0.15547800	-0.00000400
C	9.50745300	-0.22080100	-0.00000500
C	8.99434400	-1.50120200	-0.00000500
C	7.57281600	-1.57018100	-0.00000700
C	6.97181200	-0.30765000	-0.00000800
C	5.58011800	0.06835900	-0.00001100
C	5.06658600	1.34866500	-0.00001800
C	3.64506800	1.41722800	-0.00001800
C	3.04438600	0.15449800	-0.00001000
C	1.65289100	-0.22210300	-0.00000800
C	1.14014800	-1.50272500	-0.00000800
C	-0.28129500	-1.57223600	-0.00000400
C	-0.88285000	-0.30989800	-0.00000100
C	-2.27464300	0.06557000	0.00000300
C	-2.78871100	1.34564000	0.00001200
C	-4.21027600	1.41369200	0.00001500
C	-4.81053500	0.15073900	0.00000700
C	-6.20187200	-0.22648700	0.00000800
C	-6.71406400	-1.50723300	0.00001200
C	-8.13556300	-1.57743500	0.00001100

C	-8.73773700	-0.31542700	0.00000600
C	-10.12979700	0.05957700	0.00000500
C	-10.64450900	1.33912500	0.00000500
C	-12.06642600	1.40641200	0.00000400
C	-12.66588000	0.14339100	0.00000200
C	-14.05754200	-0.23526200	0.00000100
C	-14.56897400	-1.51547100	0.00000200
C	-15.99167000	-1.58549400	0.00000000
C	-16.59334600	-0.32499000	-0.00000200
C	-17.98921900	0.05264100	-0.00000400
C	-18.50152500	1.33393700	0.00000400
C	-19.92987900	1.39121500	-0.00000200
C	-20.49179400	0.13085900	-0.00001400
S	19.98625500	-1.09649700	0.00001300
S	16.04923100	0.94846400	0.00000300
S	12.12087200	-1.10256200	-0.00000200
S	8.19352000	0.95037600	-0.00000600
S	4.26644400	-1.10317900	-0.00000200
S	0.33840400	0.94859700	-0.00000400
S	-3.58797700	-1.10646600	-0.00000400
S	-7.51705500	0.94359300	0.00000400
S	-11.44282700	-1.11303400	0.00000300
S	-15.37406600	0.93338700	-0.00000300
S	-19.30613000	-1.11968400	-0.00001800
H	22.29756100	-0.26201100	0.00001600
H	21.41902900	2.22881600	0.00000800
H	17.46521900	-2.39302500	0.00000600
H	13.53786700	2.23973600	0.00000000
H	9.61019300	-2.39225600	-0.00000400
H	5.68220600	2.23987700	-0.00002400
H	1.75632700	-2.39355200	-0.00001100
H	-2.17344400	2.23710500	0.00001800
H	-6.09752500	-2.39782500	0.00001600
H	-10.02982600	2.23101800	0.00000700
H	-13.95266200	-2.40625800	0.00000400
H	-17.88493100	2.22457200	0.00001400
H	-21.54379100	-0.11616500	-0.00002000
C	-16.67878400	-2.82954300	0.00000100
C	-17.21511600	-3.91525500	0.00000200
H	-17.70726800	-4.86021000	0.00001000
C	-20.67950500	2.60217600	0.00000400
C	-21.30833400	3.63580500	0.00000800
H	-21.86544900	4.54378300	0.00001100
C	-12.75668600	2.64843000	0.00000400
C	-13.29469300	3.73342800	0.00000400
H	-13.78807600	4.67780600	0.00000200
C	-8.82311900	-2.82088800	0.00001400
C	-9.35837500	-3.90726100	0.00001700

H	-9.84896700	-4.85309700	0.00001900
C	-4.89996400	2.65594200	0.00002400
C	-5.43735200	3.74126200	0.00003300
H	-5.92951600	4.68629400	0.00003900
C	-0.96960200	-2.81524700	-0.00000400
C	-1.50562900	-3.90123800	-0.00000400
H	-1.99649700	-4.84694900	-0.00000500
C	2.95578800	2.65969400	-0.00002500
C	2.41874700	3.74518300	-0.00003100
H	1.92713100	4.69051000	-0.00003800
C	6.88401400	-2.81290700	-0.00000700
C	6.34754700	-3.89867800	-0.00000700
H	5.85649700	-4.84430400	-0.00001000
C	10.81142100	2.66059000	-0.00000300
C	10.27488300	3.74633400	-0.00000400
H	9.78379800	4.69194200	-0.00000500
C	14.73822300	-2.81368800	0.00000200
C	14.20242700	-3.89981800	0.00000200
H	13.71247700	-4.84602400	0.00000100
C	18.66945600	2.65720500	0.00000100
C	18.13599500	3.74480100	-0.00000300
H	17.64693800	4.69143300	-0.00000700
CCl_3			
C	-12.89780000	9.51489100	0.98386400
C	-13.49671500	9.19721700	-0.19909100
C	-13.56222800	7.78670000	-0.44252000
C	-12.97920100	7.04503400	0.57822200
C	-12.77528200	5.61272100	0.79616900
C	-13.14824400	4.90700200	1.91413000
C	-12.75578300	3.53559300	1.90262600
C	-12.03636800	3.20308200	0.75941800
C	-11.42296300	1.96719800	0.27529700
C	-11.60959100	1.41323800	-0.96881100
C	-10.82945000	0.24555400	-1.21555700
C	-10.00314600	-0.07276500	-0.14262100
C	-9.03445900	-1.13900800	0.10662100
C	-9.00325500	-1.94918300	1.21672500
C	-7.90380300	-2.85639500	1.25190400
C	-7.06007000	-2.70443100	0.15609500
C	-5.82716300	-3.36538200	-0.26830900
C	-5.59271500	-3.90602200	-1.51044700
C	-4.27637100	-4.42474900	-1.68681300
C	-3.48181100	-4.24038000	-0.55953400
C	-2.09974500	-4.58168200	-0.22728800
C	-1.69177000	-5.24158200	0.90783600
C	-0.27821600	-5.37651500	1.03390100
C	0.40435800	-4.77188300	-0.01669300
C	1.82031600	-4.62446700	-0.34918100

C	2.39855500	-4.93947500	-1.55590900
C	3.78632500	-4.62354400	-1.64283900
C	4.26294100	-4.01946700	-0.48367500
C	5.57086600	-3.51356900	-0.07089400
C	6.21591300	-3.82617700	1.10215900
C	7.44647000	-3.13359500	1.29890400
C	7.72300600	-2.24587300	0.26392500
C	8.82264700	-1.32178900	-0.00821000
C	9.54027600	-1.25342300	-1.17867300
C	10.51189200	-0.21031900	-1.21233700
C	10.50399800	0.55498500	-0.05063900
C	11.27662800	1.70984900	0.40425000
C	11.93389100	1.80998200	1.60741600
C	12.55096400	3.07536500	1.83563900
C	12.32223000	3.96887000	0.79492100
C	12.71618100	5.35660700	0.54914300
C	13.34327000	5.82827300	-0.57680000
C	13.50130700	7.24957200	-0.58577500
C	12.97427500	7.83960700	0.53394500
S	-12.37339500	8.10587600	1.82791100
S	-11.86543900	4.58925500	-0.28401100
S	-10.21304200	1.07155600	1.15614900
S	-7.64644900	-1.44992100	-0.90321700
S	-4.37856300	-3.43571500	0.70042700
S	-0.71369400	-4.05639300	-1.14712400
S	2.98992600	-3.86292700	0.69708300
S	6.46165500	-2.29073800	-0.93898700
S	9.30358200	-0.03527900	1.06740400
S	11.36054300	3.22564200	-0.45436900
S	12.28109800	6.68778700	1.61089600
H	-12.74361800	10.49800200	1.40571000
H	-13.89666000	9.93710400	-0.87730500
H	-13.72495100	5.35492100	2.71076300
H	-12.32085000	1.81458100	-1.67650400
H	-9.77564300	-1.91950600	1.97207700
H	-6.36285000	-3.96518500	-2.26632900
H	-2.39515300	-5.65450000	1.61682100
H	1.84695600	-5.42396300	-2.34906700
H	5.83293200	-4.56695400	1.78952100
H	9.40033500	-1.96302000	-1.98170900
H	12.00605900	0.97914800	2.29491100
H	13.70040000	5.17832800	-1.36580800
H	12.96450500	8.88919300	0.78648900
C	14.19005400	7.96955000	-1.71123300
C	13.34010400	3.37335600	3.09168100
C	11.42081400	0.00914300	-2.40161000
C	8.31609400	-3.36032300	2.51586800
C	4.60719700	-4.91944700	-2.87874500

C	0.36381000	-6.09296200	2.20148200
C	-3.83223700	-5.08578500	-2.97299700
C	-7.70878200	-3.84455200	2.38066800
C	-10.91415000	-0.52297400	-2.51593000
C	-14.18030000	7.21176600	-1.69707700
C	-13.09312900	2.58906300	3.03285100
Cl	-15.25345100	8.40966200	-2.52666200
Cl	-15.19068800	5.75246500	-1.35727900
Cl	-12.87268900	6.75062300	-2.88337300
Cl	-14.41322600	3.23916300	4.08510800
Cl	-11.63202300	2.34235600	4.09749200
Cl	-13.64830000	0.97828600	2.43115300
Cl	-12.40564700	-0.10523500	-3.44907200
Cl	-9.48561500	-0.11499100	-3.57433600
Cl	-10.94505400	-2.30994700	-2.24933500
Cl	-9.22223300	-4.07506500	3.34253100
Cl	-6.43026800	-3.23201900	3.52884300
Cl	-7.21435600	-5.47573600	1.78097800
Cl	-5.23670200	-5.55017700	-4.01263800
Cl	-2.80401500	-3.93303700	-3.94317800
Cl	-2.88236100	-6.59274400	-2.66863800
Cl	-0.81946300	-7.13704500	3.08360000
Cl	1.00592600	-4.87665600	3.40024900
Cl	1.72261100	-7.16364000	1.67993700
Cl	3.78760600	-6.11339200	-3.96127200
Cl	4.84816100	-3.39248000	-3.84665800
Cl	6.22741400	-5.60735100	-2.46995500
Cl	7.88797400	-4.89437500	3.37300400
Cl	8.08741100	-1.99756000	3.70669400
Cl	10.06847600	-3.46911600	2.09002800
Cl	11.47607100	-1.44014400	-3.48227100
Cl	10.80884600	1.40424200	-3.40447200
Cl	13.12216300	0.35681500	-1.90310400
Cl	13.84065300	1.85695400	3.94398600
Cl	12.31434100	4.33121700	4.25690900
Cl	14.84752300	4.30370100	2.74762900
Cl	15.91077100	7.40397900	-1.85188200
Cl	13.35471300	7.60826100	-3.28383100
Cl	14.20138200	9.75572400	-1.47823500
CF_3			
C	19.29375700	5.47547700	-1.58385200
C	19.26338000	5.20539600	-0.24600100
C	18.26147800	4.25302500	0.10481500
C	17.52604900	3.80120900	-0.97877500
C	16.44276800	2.83514800	-1.08695000
C	16.23320400	1.95229200	-2.12392200
C	15.06638700	1.16032100	-1.97733200
C	14.35939600	1.43196000	-0.81472100

C	13.15785000	0.84477300	-0.24585500
C	12.88895500	0.67364000	1.09613800
C	11.61742800	0.10657500	1.35932800
C	10.88733900	-0.16510100	0.21088400
C	9.58731000	-0.77985000	0.00370900
C	9.22799900	-1.58400000	-1.05793300
C	7.87878600	-2.01359900	-1.02230900
C	7.17787600	-1.53639000	0.07650200
C	5.81458000	-1.75373000	0.52904900
C	5.39062600	-1.83307500	1.83933500
C	3.99330900	-2.01953300	1.98018800
C	3.32094200	-2.08289200	0.76731600
C	1.92520000	-2.31215700	0.43624500
C	1.45209300	-2.98063400	-0.67407400
C	0.03907300	-3.02545600	-0.75920100
C	-0.59661100	-2.38331100	0.29470400
C	-1.99992800	-2.22967200	0.63725100
C	-2.52845900	-2.19221400	1.91102900
C	-3.93193500	-2.00359900	1.94547500
C	-4.50349300	-1.89022600	0.68541300
C	-5.88120900	-1.74648400	0.24846900
C	-6.42685200	-2.26843900	-0.90617500
C	-7.79212000	-1.94247100	-1.09527600
C	-8.31674600	-1.15667000	-0.07800100
C	-9.65183000	-0.63833600	0.16424100
C	-10.23969900	-0.44745600	1.39765700
C	-11.54125600	0.10788700	1.33425900
C	-11.97276300	0.35333300	0.03807300
C	-13.22692300	0.85774200	-0.49473800
C	-13.81184100	0.48417100	-1.68685100
C	-15.01952500	1.16830600	-1.97264900
C	-15.37602100	2.08573200	-0.99648300
C	-16.53042000	2.96241200	-0.85992200
C	-17.19038400	3.26798300	0.30984700
C	-18.24205800	4.20903500	0.12780000
C	-18.37534600	4.61769900	-1.17204500
S	18.08431700	4.58567300	-2.44101000
S	15.14533400	2.70920500	0.07999800
S	11.78737800	0.32621800	-1.20275800
S	8.20977700	-0.51941100	1.05185000
S	4.44421000	-1.87185600	-0.55307200
S	0.58625200	-1.69114400	1.37732100
S	-3.26730200	-1.98429000	-0.54469600
S	-7.07932900	-0.79695100	1.10110900
S	-10.72395000	-0.08143200	-1.10264900
S	-14.17784100	2.11197800	0.27184700
S	-17.21635100	3.86696400	-2.20188000
H	19.95541000	6.14425500	-2.11590900

H	19.93521400	5.64918000	0.47666100
H	16.91803700	1.84260100	-2.95470900
H	13.60123100	0.90833900	1.87644500
H	9.92480500	-1.89629800	-1.82483800
H	6.06845500	-1.80319000	2.68259400
H	2.09993000	-3.46765300	-1.39134900
H	-1.93351800	-2.34087700	2.80278000
H	-5.87584100	-2.90764700	-1.58377400
H	-9.76896200	-0.73708700	2.32820300
H	-13.41115700	-0.29333000	-2.32430800
H	-16.94541400	2.81573900	1.26341600
H	-19.09935800	5.31271600	-1.57187900
C	-19.12524600	4.67237300	1.24284900
C	-15.82658100	0.86056600	-3.20032500
C	-12.35676700	0.33914300	2.57263500
C	-8.56755200	-2.46737700	-2.26815800
C	-4.69032600	-1.99521500	3.24058000
C	-0.65644500	-3.75613300	-1.87054100
C	3.35518200	-2.18522100	3.32841500
C	7.32201000	-2.93663600	-2.06677800
C	11.17026000	-0.20344300	2.75788800
C	18.07244800	3.79437900	1.52036800
C	14.70033200	0.10843100	-2.98266500
F	-18.40279300	5.19040700	2.26175600
F	-19.99315800	5.62006700	0.83122400
F	-19.84839100	3.65357100	1.76000000
F	-15.79837600	1.88352900	-4.09190500
F	-17.12424900	0.62718200	-2.91484800
F	-15.34688500	-0.22862900	-3.83591400
F	-12.56008000	1.66194100	2.80126200
F	-13.57618700	-0.23493700	2.49818800
F	-11.73876300	-0.15855600	3.66291000
F	-7.83420100	-3.34351700	-2.98404300
F	-8.93436200	-1.47178500	-3.11560700
F	-9.70145500	-3.09642400	-1.89295600
F	-5.21973700	-0.77351100	3.50685700
F	-5.71700000	-2.87184500	3.23809500
F	-3.88811900	-2.31132900	4.27692600
F	-1.31658900	-2.90449900	-2.69666100
F	-1.56903200	-4.63868000	-1.41138300
F	0.22327100	-4.43906600	-2.63034000
F	2.54824300	-1.13719100	3.63565800
F	2.59282500	-3.29722700	3.40053500
F	4.28451400	-2.26538100	4.30153100
F	6.40884000	-2.31256400	-2.85433300
F	6.70326700	-4.00806300	-1.52644300
F	8.29441700	-3.39578600	-2.87983200
F	10.13762800	0.58959100	3.14383400

F	10.75071800	-1.47996800	2.89048400
F	12.16850400	-0.00907300	3.64277200
F	13.56944900	0.43230500	-3.66162200
F	14.47854900	-1.09390700	-2.40991800
F	15.67633300	-0.05148000	-3.89948000
F	16.91570000	4.26527000	2.05462600
F	18.02728100	2.44851900	1.62306600
F	19.07485300	4.22835200	2.31179600
CH_3			
C	20.62487900	0.31582400	-2.57607900
C	20.32116200	1.46401800	-1.90249200
C	19.00807200	1.47854800	-1.33642900
C	18.31703100	0.30414700	-1.59568200
C	16.98752000	-0.12421800	-1.20581900
C	16.51825500	-1.41611000	-1.08742000
C	15.14873400	-1.53457000	-0.73066600
C	14.53982900	-0.29607200	-0.56297300
C	13.19532700	0.05876700	-0.16780500
C	12.74058500	1.28755300	0.27194500
C	11.35346400	1.35595600	0.55943700
C	10.70957100	0.14269800	0.34156400
C	9.33415700	-0.25366800	0.53606500
C	8.82664200	-1.53749300	0.60565100
C	7.42103200	-1.62837400	0.76842300
C	6.81488400	-0.37771500	0.83177100
C	5.43515800	0.00288500	1.02399300
C	4.93988100	1.25439400	1.34087800
C	3.52825900	1.34424400	1.43707300
C	2.90416200	0.12529700	1.19307500
C	1.51077200	-0.25335000	1.21862400
C	0.98338700	-1.52984700	1.27592800
C	-0.43195900	-1.60566900	1.25002600
C	-1.02740500	-0.35021100	1.17377600
C	-2.41597300	0.04450800	1.15426700
C	-2.93552700	1.31811600	1.29691700
C	-4.34704900	1.41404900	1.20873100
C	-4.94802400	0.17768800	0.99517500
C	-6.33711600	-0.19884200	0.87786700
C	-6.87630800	-1.46995500	0.94735700
C	-8.28015400	-1.54703400	0.76518000
C	-8.85446200	-0.29815300	0.55111200
C	-10.23023500	0.09607000	0.35663900
C	-10.76011200	1.37184000	0.41000300
C	-12.15104200	1.46512800	0.14874700
C	-12.72313400	0.22494800	-0.11101700
C	-14.09062100	-0.15592600	-0.38503100
C	-14.64696800	-1.41805200	-0.30584700
C	-16.01803700	-1.50166400	-0.66608700

C	-16.54128200	-0.26930100	-1.03165200
C	-17.88170600	0.11954700	-1.42645700
C	-18.45670300	1.37068600	-1.34230300
C	-19.78372200	1.45167300	-1.86419300
C	-20.21071500	0.24265300	-2.34721700
S	19.30002800	-0.79640300	-2.55671600
S	15.68292200	1.00235200	-0.88499100
S	11.84749000	-1.06299800	-0.24843800
S	8.01502700	0.89838400	0.65994700
S	4.09584800	-1.11429500	0.81855200
S	0.20000900	0.91054600	1.11298500
S	-3.73077300	-1.08837500	0.88288600
S	-7.62011700	0.95672300	0.55604900
S	-11.50395300	-1.04198400	-0.05261900
S	-15.30580600	0.97828400	-0.94995400
S	-19.01131500	-0.99901100	-2.18246200
H	21.54411700	0.05711300	-3.08253800
H	21.01469900	2.29100100	-1.79405300
H	17.15795700	-2.28028800	-1.23121700
H	13.40198500	2.13596000	0.41101900
H	9.46142800	-2.41599600	0.56314400
H	5.58655000	2.10534500	1.52613100
H	1.60764900	-2.41309200	1.35773400
H	-2.30959500	2.18426600	1.48304500
H	-6.27172900	-2.34751900	1.14977500
H	-10.15993900	2.24089900	0.65751200
H	-14.08442100	-2.28058800	0.03528300
H	-17.94418900	2.21604600	-0.89540700
H	-21.17267500	0.00350200	-2.77990900
C	-20.60750000	2.71118400	-1.86438200
H	-20.74868300	3.09444200	-0.84734900
H	-20.12086000	3.50423800	-2.44363100
H	-21.59617700	2.53943200	-2.29762400
C	-16.78142200	-2.79929300	-0.61204000
H	-17.78102100	-2.66746000	-0.18787600
H	-16.90761800	-3.23925900	-1.60854200
H	-16.24674600	-3.52960200	0.00143800
C	-12.88428500	2.78032800	0.18707000
H	-13.16451000	3.12077100	-0.81678200
H	-13.80472400	2.71788200	0.77591900
H	-12.25256100	3.55447000	0.63061100
C	-9.02139200	-2.85647800	0.83096800
H	-9.87782200	-2.80775300	1.51115200
H	-9.40406700	-3.15909000	-0.15079700
H	-8.35773100	-3.65051200	1.18299100
C	-5.06830500	2.72761100	1.35919200
H	-5.49336000	3.07092200	0.40868000
H	-5.89202600	2.66179600	2.07713200

H	-4.37910800	3.50095800	1.70822100
C	-1.15988900	-2.92207500	1.32298000
H	-1.91838600	-2.92721800	2.11260200
H	-1.66988200	-3.15956300	0.38210900
H	-0.45659900	-3.73310500	1.52881400
C	2.83246800	2.63194200	1.79219700
H	2.31176500	3.06530100	0.93009700
H	2.08818100	2.49001700	2.58204900
H	3.55773800	3.37132100	2.14184000
C	6.71605300	-2.95457300	0.88021800
H	6.04667700	-2.99056400	1.74568900
H	6.11011200	-3.17087100	-0.00749900
H	7.44401500	-3.76326400	0.98476500
C	10.70522700	2.61715900	1.06706000
H	10.07628800	3.08880100	0.30275500
H	10.06980500	2.42869700	1.93805700
H	11.46742200	3.34486000	1.35779700
C	18.49010500	2.64975500	-0.54302800
H	17.83290800	3.29135700	-1.14262600
H	17.91883700	2.32985900	0.33311500
H	19.32109100	3.27026600	-0.19596700
C	14.48725900	-2.87410000	-0.53956300
H	13.91988300	-2.92099800	0.39526900
H	13.79026500	-3.10513600	-1.35387800
H	15.23831500	-3.66804300	-0.51723800
CHO			
C	17.55777000	6.59456500	-0.51096500
C	17.62308100	6.09324600	0.75267000
C	16.85344900	4.89776900	0.92483600
C	16.19510800	4.50758100	-0.23822200
C	15.31319800	3.38374200	-0.51363900
C	15.23359000	2.64175900	-1.66603600
C	14.23163500	1.62860400	-1.63977100
C	13.51651300	1.60331100	-0.44431600
C	12.42967500	0.75286300	0.01012000
C	12.20021800	0.29498700	1.28494500
C	11.01920800	-0.49006200	1.41855800
C	10.31761200	-0.62384800	0.22198200
C	9.08654800	-1.32378200	-0.10034200
C	8.77174200	-1.96704900	-1.27312400
C	7.46259000	-2.52707900	-1.29736200
C	6.75073300	-2.29458400	-0.12217100
C	5.41023600	-2.67778400	0.28460200
C	4.98596000	-3.02454700	1.54461200
C	3.59428400	-3.31318100	1.63198900
C	2.93209400	-3.16831700	0.41420000
C	1.53984000	-3.35542500	0.04654000
C	1.04491200	-3.82382200	-1.14682100

C	-0.37700600	-3.85660300	-1.21526900
C	-0.98745300	-3.38734000	-0.05360200
C	-2.38577300	-3.24753900	0.31196700
C	-2.94461400	-3.41445300	1.55619100
C	-4.34630600	-3.16873300	1.60313900
C	-4.87251200	-2.78843300	0.36984300
C	-6.22420100	-2.44859300	-0.03749100
C	-6.82147300	-2.70219800	-1.24885100
C	-8.15291900	-2.20907300	-1.35648100
C	-8.58149200	-1.54733900	-0.20724000
C	-9.83975300	-0.90056400	0.11994900
C	-10.45831700	-0.84758300	1.34583200
C	-11.67098800	-0.10123000	1.35469700
C	-11.98124000	0.44524000	0.11094800
C	-13.09770400	1.26225000	-0.33143600
C	-13.71345800	1.24216700	-1.55967100
C	-14.76036500	2.19759500	-1.70080100
C	-14.93917100	2.97935100	-0.56254200
C	-15.87286200	4.05678100	-0.27376100
C	-16.50308600	4.32489800	0.91759100
C	-17.31368800	5.49931800	0.87807900
C	-17.28354600	6.12144300	-0.35002100
S	16.53253000	5.63365700	-1.53233900
S	14.08330100	2.85827800	0.62939200
S	11.12476400	0.24911400	-1.05738200
S	7.71148200	-1.35911400	0.99687400
S	4.04714000	-2.65235000	-0.82764500
S	0.21706900	-2.88722900	1.10815700
S	-3.61029300	-2.71930800	-0.83578500
S	-7.31222500	-1.52599000	0.99250200
S	-10.74705800	0.04157000	-1.05712700
S	-13.78881800	2.53192800	0.67166400
S	-16.26794400	5.29637800	-1.46653300
H	18.04588700	7.47128000	-0.91369900
H	18.20375300	6.52095600	1.56005300
H	15.89386500	2.76830200	-2.51517600
H	12.86841600	0.46347700	2.12063600
H	9.45853200	-2.09196900	-2.10144300
H	5.64131200	-3.12356300	2.40141000
H	1.66092400	-4.18716600	-1.96044600
H	-2.39742400	-3.74968000	2.42894600
H	-6.35906700	-3.26717600	-2.04915500
H	-10.10026300	-1.35996600	2.23057500
H	-13.47406100	0.53978700	-2.34889800
H	-16.42412100	3.70230100	1.80052800
H	-17.80971800	7.01806400	-0.65085900
C	14.09419800	0.67186700	-2.75477400
O	14.73381500	0.76528000	-3.78718700

H	13.38420200	-0.16315300	-2.60101800
C	16.87423900	4.15536200	2.19767200
O	17.44831000	4.56093700	3.19324000
H	16.34943000	3.18034200	2.20494900
C	10.69307100	-1.15053100	2.69811800
O	11.33859200	-0.97045500	3.71482900
H	9.83155300	-1.84500000	2.68768100
C	7.01381300	-3.34801100	-2.43979400
O	7.68042200	-3.49079200	-3.44870300
H	6.03558900	-3.85313200	-2.32839700
C	3.00683100	-3.81202300	2.89184900
O	3.64320500	-3.89077100	3.92692300
H	1.94972700	-4.13572900	2.84836200
C	-1.06185900	-4.44059000	-2.38620100
O	-0.46319800	-4.81081500	-3.37969300
H	-2.16065200	-4.54707700	-2.31155000
C	-5.11289800	-3.41614300	2.84111400
O	-4.58082600	-3.72563200	3.89161800
H	-6.21301800	-3.32589100	2.76573500
C	-8.97026800	-2.50050700	-2.55155600
O	-8.52132900	-3.06574200	-3.53206500
H	-10.03270200	-2.19480700	-2.50772200
C	-12.51286800	-0.04584100	2.56691600
O	-12.16565000	-0.52799400	3.62962000
H	-13.49907300	0.44408300	2.45946400
C	-15.59754500	2.23033000	-2.91708500
O	-15.36846400	1.53726500	-3.89156000
H	-16.46941000	2.91122300	-2.89413800
C	-18.10536000	5.98810200	2.02153100
O	-18.13432900	5.43592500	3.10427100
H	-18.68776800	6.91396400	1.82848100
Cl			
C	21.33830400	-0.05543100	0.18890300
C	20.87398800	-1.33701200	0.18816700
C	19.45082700	-1.39113000	0.16761900
C	18.81993900	-0.16057700	0.15293600
C	17.42311900	0.21672800	0.13219800
C	16.92196600	1.50190700	0.13109000
C	15.51031000	1.55403500	0.10613900
C	14.88028900	0.32195800	0.08790000
C	13.48728700	-0.05542500	0.06307900
C	12.98541800	-1.34207900	0.05591700
C	11.57479200	-1.39376800	0.02905600
C	10.94372300	-0.16136200	0.01496100
C	9.55128100	0.21550300	-0.01053300
C	9.04985100	1.50266500	-0.01957700
C	7.63954000	1.55512300	-0.04486000
C	7.00746200	0.32302700	-0.05612100

C	5.61476700	-0.05238600	-0.07996200
C	5.11169600	-1.33894900	-0.09247200
C	3.70124500	-1.38959100	-0.11310000
C	3.07055400	-0.15671600	-0.11754900
C	1.67835900	0.22073700	-0.13607100
C	1.17733700	1.50807100	-0.15046700
C	-0.23313100	1.56105200	-0.16235600
C	-0.86597700	0.32930600	-0.15840300
C	-2.25891400	-0.04581100	-0.16807500
C	-2.76218300	-1.33215700	-0.18515600
C	-4.17285100	-1.38286000	-0.18381500
C	-4.80365900	-0.15021100	-0.16721000
C	-6.19608800	0.22724100	-0.16345100
C	-6.69769600	1.51408500	-0.18273100
C	-8.10831900	1.56693900	-0.16267400
C	-8.74057100	0.33551800	-0.12984100
C	-10.13350200	-0.04046300	-0.10832300
C	-10.63674100	-1.32620600	-0.13611300
C	-12.04709800	-1.37814500	-0.08886600
C	-12.67697300	-0.14703500	-0.02766100
C	-14.06990300	0.23004900	0.02012800
C	-14.57437200	1.51446600	-0.02518500
C	-15.98412200	1.56684300	0.06088700
C	-16.60849700	0.33808800	0.16751200
C	-18.00320500	-0.04309300	0.24724100
C	-18.51877400	-1.30933100	0.05269400
C	-19.92233900	-1.36751300	0.25434200
C	-20.48675200	-0.17098700	0.59646700
S	20.04965100	1.10113100	0.16405000
S	16.10468100	-0.94365300	0.10142200
S	12.16726500	1.10500300	0.03566800
S	8.23025300	-0.94422500	-0.03411700
S	4.29491100	1.10897100	-0.09414800
S	0.35640600	-0.93839400	-0.13711100
S	-3.57905700	1.11536600	-0.14931100
S	-7.51766800	-0.93163900	-0.11782300
S	-11.45287200	1.11850200	-0.01834700
S	-15.38504700	-0.92477600	0.18525900
S	-19.29033400	1.07665200	0.68012000
H	22.36422000	0.28427100	0.20220900
H	21.49973600	-2.21994300	0.20157900
H	17.53558400	2.39368000	0.14829100
H	13.59898800	-2.23389400	0.07006700
H	9.66399500	2.39410600	-0.00817100
H	5.72484600	-2.23113400	-0.08710700
H	1.79199700	2.39922900	-0.15300200
H	-2.14903800	-2.22425700	-0.19943600
H	-6.08356500	2.40513800	-0.21255300

H	-10.02441300	-2.21723600	-0.19248300
H	-13.96485400	2.40351200	-0.12638900
H	-17.92556700	-2.16735600	-0.23712800
H	-21.52761400	0.04772800	0.78338700
Cl	18.62506300	-2.92709700	0.16305800
Cl	14.68547700	3.09089300	0.10147000
Cl	10.74910000	-2.93010000	0.01669700
Cl	6.81493600	3.09211800	-0.05959000
Cl	2.87459400	-2.92549700	-0.13165300
Cl	-1.05721700	3.09841200	-0.18234700
Cl	-4.99943100	-2.91895600	-0.20569600
Cl	-8.93305800	3.10421200	-0.18553200
Cl	-12.87382400	-2.91450800	-0.11962600
Cl	-16.81677500	3.10013800	0.00503600
Cl	-20.83460900	-2.84440800	0.06172500
CN			
C	21.25884700	-0.07988700	0.08888300
C	20.78013600	-1.35431400	0.08286300
C	19.34780400	-1.40533600	0.07350200
C	18.74694100	-0.14689900	0.07256300
C	17.34970900	0.22981500	0.06468100
C	16.83695800	1.50840600	0.06697400
C	15.41622200	1.55773800	0.05720500
C	14.81705200	0.29647300	0.04731400
C	13.42345200	-0.08063500	0.03637700
C	12.91163500	-1.36143000	0.03135700
C	11.49214800	-1.41091000	0.01998100
C	10.89128100	-0.14933400	0.01626100
C	9.49777200	0.22603400	0.00595900
C	8.98552800	1.50699300	0.00549200
C	7.56643900	1.55643300	-0.00624900
C	6.96575900	0.29444100	-0.01494700
C	5.57230700	-0.08049900	-0.02739800
C	5.05951400	-1.36128200	-0.03534000
C	3.64047000	-1.41003500	-0.04707200
C	3.04030600	-0.14765000	-0.04825300
C	1.64712600	0.22809500	-0.05869000
C	1.13539500	1.50930900	-0.06450300
C	-0.28356700	1.55938400	-0.07391200
C	-0.88487200	0.29745000	-0.07562300
C	-2.27839300	-0.07683000	-0.08514000
C	-2.79164000	-1.35733100	-0.10040200
C	-4.21071100	-1.40576900	-0.10521400
C	-4.81058400	-0.14315500	-0.09402700
C	-6.20358000	0.23300400	-0.09713700
C	-6.71492600	1.51413200	-0.11684500
C	-8.13395100	1.56494500	-0.11149600
C	-8.73573600	0.30337400	-0.08838600

C	-10.12937200	-0.07049200	-0.08203400
C	-10.64349900	-1.34996400	-0.11980600
C	-12.06266800	-1.39835200	-0.09618400
C	-12.66116300	-0.13644700	-0.04140000
C	-14.05414900	0.24083000	-0.01498900
C	-14.56661200	1.51927800	-0.08314000
C	-15.98546400	1.57147200	-0.02370200
C	-16.58290700	0.31439700	0.08939200
C	-17.97845700	-0.06210100	0.15775100
C	-18.49680800	-1.33580000	0.04116100
C	-19.91518300	-1.37976000	0.18050100
C	-20.46354500	-0.13304200	0.40118000
S	19.98224500	1.09246700	0.08318600
S	16.04403700	-0.94779000	0.04988000
S	12.11609500	1.09652500	0.02678200
S	8.19041900	-0.95137400	-0.00840300
S	4.26541400	1.09757100	-0.03419400
S	0.33915000	-0.94887400	-0.06416600
S	-3.58509400	1.10161700	-0.07464900
S	-7.51214400	-0.94320200	-0.06782800
S	-11.43512600	1.10697200	-0.00857300
S	-15.35950600	-0.92868900	0.13857800
S	-19.27054600	1.10071600	0.44048400
H	22.28816500	0.24945300	0.09627600
H	21.40116000	-2.24099500	0.08486700
H	17.44630500	2.40381300	0.07562700
H	13.52157500	-2.25672800	0.03589600
H	9.59526700	2.40241300	0.01347400
H	5.66884200	-2.25703900	-0.03301800
H	1.74547800	2.40456000	-0.06270200
H	-2.18260300	-2.25326400	-0.10947200
H	-6.10457500	2.40898200	-0.13773700
H	-10.03533900	-2.24519700	-0.16938300
H	-13.95874400	2.41028800	-0.18498200
H	-17.89735300	-2.21775700	-0.14964800
H	-21.50816700	0.11130300	0.53226500
C	-20.68927800	-2.57630300	0.09362200
N	-21.31733500	-3.55268800	0.02266300
C	-16.69614400	2.80473100	-0.09306700
N	-17.25814600	3.82294000	-0.14600600
C	-12.77442700	-2.63187000	-0.13575400
N	-13.34122300	-3.64853200	-0.16547100
C	-8.84280000	2.80048400	-0.13295600
N	-9.40833500	3.81819700	-0.14894700
C	-4.92191900	-2.64000500	-0.12229000
N	-5.49040600	-3.65611000	-0.13538900
C	-0.99340500	2.79450400	-0.08168200
N	-1.56105100	3.81115000	-0.08754700

C	2.92916600	-2.64430200	-0.05678100
N	2.35972400	-3.65992900	-0.06450100
C	6.85593900	2.79119700	-0.00849600
N	6.28746800	3.80740900	-0.01021900
C	10.78130100	-2.64545100	0.01355600
N	10.21179700	-3.66107900	0.00830100
C	14.70746800	2.79329600	0.05810400
N	14.14395800	3.81240100	0.05886400
C	18.63736500	-2.63905100	0.06616200
N	18.07229700	-3.65758200	0.06016900
COOH			
C	13.65519200	8.83195200	-0.46723900
C	14.10898500	8.40098600	0.74180300
C	14.00789100	6.98273700	0.91352700
C	13.45657800	6.34099600	-0.19012400
C	13.13314600	4.94916200	-0.49085600
C	13.37213200	4.29129400	-1.67180900
C	12.85425100	2.96401600	-1.72066400
C	12.18009800	2.60539000	-0.55631300
C	11.48379000	1.39934100	-0.12142000
C	11.54327400	0.83281600	1.12884600
C	10.67208300	-0.27922200	1.31294100
C	9.90617400	-0.55621600	0.18324600
C	8.88685800	-1.55239100	-0.12680800
C	8.75712700	-2.24419300	-1.30680300
C	7.60092800	-3.07333600	-1.37861100
C	6.81042400	-2.99575800	-0.23442200
C	5.55858700	-3.62170500	0.17577900
C	5.26545700	-4.10346800	1.42895300
C	3.92531300	-4.55946000	1.58576100
C	3.16359700	-4.40014400	0.43048200
C	1.77673200	-4.69071700	0.08527000
C	1.32856100	-5.21763000	-1.10212400
C	-0.08939100	-5.29035000	-1.21572200
C	-0.74605300	-4.78279400	-0.09696700
C	-2.14908400	-4.62513200	0.26865900
C	-2.69401100	-4.85273700	1.50961800
C	-4.07120400	-4.50683200	1.62013400
C	-4.58950500	-3.97689000	0.44094700
C	-5.90213800	-3.47617300	0.04889200
C	-6.53405000	-3.70467300	-1.14955300
C	-7.76191300	-3.00074300	-1.31075800
C	-8.06630500	-2.19132100	-0.21860400
C	-9.16857400	-1.29024500	0.09751400
C	-9.77974900	-1.15423700	1.32087200
C	-10.75461900	-0.11753900	1.37980100
C	-10.87685600	0.57580200	0.17789400
C	-11.70332300	1.69248000	-0.26695300

C	-12.32356800	1.80618900	-1.48796600
C	-12.97848500	3.05333600	-1.70034800
C	-12.83371100	3.92770400	-0.62702600
C	-13.29595000	5.28767100	-0.36409200
C	-13.83651700	5.75255700	0.81112500
C	-14.06842900	7.15955600	0.81109800
C	-13.68225300	7.75810200	-0.36600200
S	13.06388200	7.51601900	-1.42625900
S	12.19793600	3.92895700	0.58035700
S	10.28362200	0.57337400	-1.09189300
S	7.51996500	-1.88947400	0.91334200
S	4.13003400	-3.68442700	-0.83395500
S	0.41433300	-4.22209700	1.07940000
S	-3.35251900	-3.91817600	-0.78814500
S	-6.81670500	-2.31995500	0.99264000
S	-9.77382000	-0.07477100	-1.00727300
S	-11.88617100	3.18264700	0.63338800
S	-13.04796400	6.62031100	-1.48630600
H	13.63297400	9.84059000	-0.85544800
H	14.52715200	9.03947000	1.50906200
H	13.93514400	4.71342300	-2.49450100
H	12.21530300	1.16450800	1.90997800
H	9.48042800	-2.20616300	-2.11132200
H	5.98756500	-4.17433200	2.23232300
H	1.98100900	-5.58707400	-1.88297000
H	-2.15030900	-5.29218500	2.33617500
H	-6.16702200	-4.38753900	-1.90511000
H	-9.57996400	-1.79487800	2.17031200
H	-12.35730200	1.01339800	-2.22432200
H	-14.08306100	5.11959900	1.65423000
H	-13.75862900	8.80435200	-0.62526900
C	-14.66616700	7.85436700	1.96728600
O	-15.00235600	7.31039400	3.00001700
O	-14.80609700	9.18840000	1.76259200
H	-15.20173400	9.54250100	2.57621200
C	-13.76278200	3.22491900	-2.94379600
O	-13.77954200	2.40897300	-3.84481400
O	-14.48656900	4.36638100	-2.99144400
H	-14.95019900	4.35283900	-3.84568000
C	-11.53491200	0.03072700	2.62867300
O	-11.30992200	-0.60452600	3.64026900
O	-12.54476900	0.92705400	2.54987900
H	-12.97069600	0.92869800	3.42371000
C	-8.56106400	-3.27025400	-2.52699800
O	-8.17055700	-3.96144400	-3.44747200
O	-9.78577600	-2.69610100	-2.52571100
H	-10.20055200	-2.94688700	-3.36835800
C	-4.76514600	-4.81618400	2.89030500

O	-4.19832200	-5.23751300	3.87957500
O	-6.10141100	-4.60943900	2.85783600
H	-6.43098800	-4.84586700	3.74128700
C	-0.65687300	-5.94047300	-2.41800700
O	0.01367900	-6.30601400	-3.36361600
O	-1.99724600	-6.11677600	-2.37352300
H	-2.23938200	-6.54645100	-3.21122900
C	3.55345300	-5.19044100	2.87187200
O	4.29059100	-5.23151700	3.83748800
O	2.31924900	-5.74385600	2.88306200
H	2.20086100	-6.11749300	3.77267100
C	7.43751400	-3.93401800	-2.57132400
O	8.16781900	-3.88301900	-3.54166200
O	6.40929700	-4.80911200	-2.48826500
H	6.41155600	-5.30569500	-3.32396600
C	10.74728300	-1.01588800	2.59431000
O	11.41672700	-0.65086900	3.54095400
O	10.01890500	-2.15553800	2.62356900
H	10.15266700	-2.53629600	3.50794600
C	13.15388200	2.14388700	-2.91525000
O	13.70624600	2.57725800	-3.90749900
O	12.77800200	0.84826800	-2.80842400
H	13.02782900	0.42757100	-3.64836400
C	14.54443800	6.39044100	2.15908800
O	14.94745100	7.04919500	3.09796900
O	14.57517200	5.03738300	2.16449100
H	14.94457300	4.78315300	3.02691300
F			
C	-21.11797300	0.13226800	-0.00000200
C	-20.59896200	1.39603500	-0.00000200
C	-19.18204500	1.36657200	-0.00000100
C	-18.60429700	0.11571700	-0.00000100
C	-17.20803400	-0.24205600	0.00000000
C	-16.65639200	-1.50733300	0.00000100
C	-15.24899900	-1.47102900	0.00000100
C	-14.67246300	-0.21836700	0.00000000
C	-13.28077800	0.14070600	0.00000000
C	-12.73058600	1.40860900	0.00000000
C	-11.32426600	1.37479000	0.00000000
C	-10.74395300	0.12303400	0.00000000
C	-9.35233700	-0.23266400	0.00000000
C	-8.79995400	-1.50007700	0.00000100
C	-7.39405800	-1.46422200	0.00000100
C	-6.81507500	-0.21165300	0.00000000
C	-5.42396700	0.14499300	0.00000000
C	-4.87184500	1.41264900	0.00000000
C	-3.46593800	1.37724000	0.00000000
C	-2.88642000	0.12490900	0.00000000

C	-1.49542000	-0.23185800	0.00000000
C	-0.94380100	-1.49972700	0.00000100
C	0.46210600	-1.46501700	0.00000100
C	1.04237200	-0.21306400	0.00000100
C	2.43367000	0.14267600	0.00000100
C	2.98674900	1.40982700	0.00000100
C	4.39275700	1.37362900	0.00000100
C	4.97169200	0.12115100	0.00000100
C	6.36255400	-0.23678800	0.00000100
C	6.91348500	-1.50468300	0.00000200
C	8.31972900	-1.47103900	0.00000200
C	8.90091600	-0.21978600	0.00000100
C	10.29270600	0.13591000	0.00000000
C	10.84628700	1.40222700	0.00000000
C	12.25298300	1.36574000	-0.00000100
C	12.83138000	0.11370900	0.00000000
C	14.22305700	-0.24641600	-0.00000100
C	14.77275800	-1.51335700	-0.00000100
C	16.18102100	-1.48045200	-0.00000100
C	16.76177300	-0.23144700	-0.00000100
C	18.15842100	0.12736600	0.00000000
C	18.70964800	1.39192300	0.00000300
C	20.12655200	1.35589800	0.00000200
C	20.67332500	0.10607800	-0.00000300
S	-19.88352600	-1.08803400	-0.00000100
S	-15.94909900	0.98897600	-0.00000100
S	-12.01757900	-1.08796600	0.00000100
S	-8.09045700	0.99787300	0.00000000
S	-4.16142600	-1.08509600	0.00000100
S	-0.23215000	0.99761500	0.00000000
S	3.69582200	-1.08808900	0.00000100
S	7.62712000	0.99163800	0.00000000
S	11.55516900	-1.09483100	0.00000000
S	15.48971200	0.97976000	0.00000000
S	19.42416800	-1.09800300	-0.00000500
H	-22.15764200	-0.16214900	-0.00000200
H	-21.17826200	2.31021100	-0.00000200
H	-17.22362900	-2.42950300	0.00000200
H	-13.29980900	2.32950300	-0.00000100
H	-9.36799800	-2.42167100	0.00000100
H	-5.44025000	2.33401900	-0.00000100
H	-1.51264600	-2.42082500	0.00000100
H	2.41892200	2.33156400	0.00000000
H	6.34412700	-2.42549500	0.00000300
H	10.27898700	2.32434400	-0.00000100
H	14.20333600	-2.43422400	-0.00000200
H	18.13952900	2.31261100	0.00000600
H	21.71778700	-0.16569200	-0.00000400

F	20.85721300	2.48385900	0.00000500
F	12.99049100	2.49187600	-0.00000100
F	16.91406400	-2.61006700	-0.00000200
F	5.13089000	2.49883700	0.00000100
F	9.05551700	-2.59800300	0.00000300
F	1.19914700	-2.59080300	0.00000100
F	-6.65602200	-2.58913100	0.00000100
F	-2.72819700	2.50244100	-0.00000100
F	-14.51108600	-2.59584300	0.00000200
F	-10.58783600	2.50062100	-0.00000100
F	-18.44604400	2.49289200	-0.00000200
H-planar			
C	0.00000000	20.86104000	0.14696800
C	0.00000000	20.33662700	1.40990500
C	0.00000000	18.91410700	1.41888100
C	0.00000000	18.35297400	0.15832900
C	0.00000000	16.95703300	-0.21833100
C	0.00000000	16.39506500	-1.47900500
C	0.00000000	14.98097500	-1.47602100
C	0.00000000	14.42484700	-0.21168200
C	0.00000000	13.03511900	0.17088100
C	0.00000000	12.47816800	1.43562600
C	0.00000000	11.06525500	1.43893700
C	0.00000000	10.50236100	0.17673300
C	0.00000000	9.11160200	-0.19912500
C	0.00000000	8.54976000	-1.46208500
C	0.00000000	7.13725800	-1.46017200
C	0.00000000	6.57879000	-0.19574400
C	0.00000000	5.18924700	0.18385000
C	0.00000000	4.62997700	1.44803200
C	0.00000000	3.21747800	1.44901200
C	0.00000000	2.65642800	0.18564600
C	0.00000000	1.26639700	-0.19198800
C	0.00000000	0.70619800	-1.45576700
C	0.00000000	-0.70619800	-1.45576700
C	0.00000000	-1.26639700	-0.19198800
C	0.00000000	-2.65642800	0.18564600
C	0.00000000	-3.21747800	1.44901200
C	0.00000000	-4.62997700	1.44803200
C	0.00000000	-5.18924700	0.18385000
C	0.00000000	-6.57879000	-0.19574400
C	0.00000000	-7.13725800	-1.46017200
C	0.00000000	-8.54976000	-1.46208500
C	0.00000000	-9.11160200	-0.19912500
C	0.00000000	-10.50236100	0.17673300
C	0.00000000	-11.06525500	1.43893700
C	0.00000000	-12.47816800	1.43562600
C	0.00000000	-13.03511900	0.17088100

C	0.00000000	-14.42484700	-0.21168200
C	0.00000000	-14.98097500	-1.47602100
C	0.00000000	-16.39506500	-1.47900500
C	0.00000000	-16.95703300	-0.21833100
H	0.00000000	21.89984300	-0.15150300
S	0.00000000	19.61751800	-1.06301000
H	0.00000000	20.94378300	2.30762900
S	0.00000000	15.69514600	1.00463800
H	0.00000000	18.32149500	2.32683500
S	0.00000000	11.76619400	-1.04650500
H	0.00000000	16.98793900	-2.38669200
S	0.00000000	7.84692200	1.02304800
H	0.00000000	14.38419900	-2.38116700
S	0.00000000	3.92200700	-1.03570000
H	0.00000000	13.07522800	2.34054000
S	0.00000000	0.00000000	1.02851900
H	0.00000000	10.47251000	2.34672100
S	0.00000000	-3.92200700	-1.03570000
H	0.00000000	9.14348300	-2.36918600
S	0.00000000	-7.84692200	1.02304800
H	0.00000000	6.54117800	-2.36575600
S	0.00000000	-11.76619400	-1.04650500
H	0.00000000	5.22554600	2.35393500
S	0.00000000	-15.69514600	1.00463800
H	0.00000000	2.62320100	2.35577600
H	0.00000000	1.30110800	-2.36210400
H	0.00000000	-1.30110800	-2.36210400
H	0.00000000	-2.62320100	2.35577600
H	0.00000000	-5.22554600	2.35393500
H	0.00000000	-6.54117800	-2.36575600
H	0.00000000	-9.14348300	-2.36918600
H	0.00000000	-10.47251000	2.34672100
H	0.00000000	-13.07522800	2.34054000
H	0.00000000	-14.38419900	-2.38116700
H	0.00000000	-16.98793900	-2.38669200
C	0.00000000	-18.35297400	0.15832900
C	0.00000000	-18.91410700	1.41888100
S	0.00000000	-19.61751800	-1.06301000
C	0.00000000	-20.33662700	1.40990500
H	0.00000000	-18.32149500	2.32683500
C	0.00000000	-20.86104000	0.14696800
H	0.00000000	-20.94378300	2.30762900
H	0.00000000	-21.89984300	-0.15150300
H C-shaped			
C	-1.26574300	-0.22504200	-0.75589200
C	-0.70652900	-1.48593900	-0.84287100
C	0.70652800	-1.48593900	-0.84287100
C	1.26574300	-0.22504200	-0.75589200

S	0.00000000	0.99070700	-0.65077100
H	-1.30259000	-2.38768700	-0.92583700
H	1.30259000	-2.38768700	-0.92583700
C	2.65608100	0.15392700	-0.73760400
C	3.21856700	1.41131600	-0.85033400
S	3.91669500	-1.05596000	-0.54210200
C	4.63072000	1.41261700	-0.79942100
H	2.62627200	2.30886200	-0.98788800
C	5.18581300	0.15624100	-0.64637200
H	5.22998900	2.31101800	-0.89454400
C	6.57462600	-0.22033400	-0.56458900
C	7.14176600	-1.47925800	-0.62224100
S	7.82598000	0.99637200	-0.35284800
C	8.55117700	-1.47673700	-0.51794100
H	6.55576400	-2.38102900	-0.75916900
C	9.09899700	-0.21580500	-0.37781900
H	9.15455400	-2.37606400	-0.56735700
C	-2.65608100	0.15392700	-0.73760500
C	-3.21856700	1.41131600	-0.85033400
S	-3.91669500	-1.05596000	-0.54210300
C	-4.63072000	1.41261700	-0.79942100
H	-2.62627200	2.30886200	-0.98788800
C	-5.18581300	0.15624100	-0.64637200
H	-5.22998900	2.31101800	-0.89454400
C	-6.57462600	-0.22033400	-0.56459000
C	-7.14176600	-1.47925800	-0.62224200
S	-7.82598000	0.99637200	-0.35284800
C	-8.55117700	-1.47673700	-0.51794100
H	-6.55576400	-2.38102900	-0.75917000
C	-9.09899700	-0.21580500	-0.37781900
H	-9.15455400	-2.37606400	-0.56735800
C	10.48360900	0.16596700	-0.25447000
C	11.05205600	1.42264100	-0.33718600
S	11.72569700	-1.03940800	0.05415700
C	12.45663800	1.42638200	-0.17742000
H	10.47179400	2.31731900	-0.53264300
C	12.99827200	0.17279700	0.03170000
H	13.06206800	2.32354500	-0.23921600
C	14.37802000	-0.20211100	0.22292300
C	14.95496100	-1.45602200	0.17790400
S	15.59875600	1.01162100	0.57804300
C	16.35248200	-1.45004800	0.39984600
H	14.38872400	-2.35514900	-0.03731000
C	16.87637800	-0.19276500	0.62066300
H	16.96581700	-2.34353000	0.37133700
C	-10.48360900	0.16596700	-0.25447100
C	-11.05205600	1.42264100	-0.33718700
S	-11.72569700	-1.03940800	0.05415600

C	-12.45663800	1.42638200	-0.17742000
H	-10.47179400	2.31731900	-0.53264300
C	-12.99827200	0.17279700	0.03170000
H	-13.06206800	2.32354500	-0.23921600
C	-14.37802000	-0.20211100	0.22292200
C	-14.95496100	-1.45602200	0.17790400
S	-15.59875600	1.01162100	0.57804300
C	-16.35248200	-1.45004800	0.39984600
H	-14.38872400	-2.35514900	-0.03731000
C	-16.87637800	-0.19276500	0.62066300
H	-16.96581700	-2.34352900	0.37133700
C	18.24800400	0.19501000	0.86850400
C	18.82864800	1.44265100	0.77427600
S	19.44292900	-0.99052600	1.37271100
C	20.21546000	1.45018800	1.09237700
H	18.27820900	2.32401800	0.46509300
C	20.69181100	0.21255700	1.42697400
H	20.83418900	2.33958900	1.06485800
H	21.69792700	-0.06954500	1.70346400
C	-18.24800400	0.19501000	0.86850400
C	-18.82864800	1.44265100	0.77427600
S	-19.44292900	-0.99052600	1.37271100
C	-20.21545900	1.45018800	1.09237800
H	-18.27821000	2.32401800	0.46509300
C	-20.69181100	0.21255700	1.42697600
H	-20.83418800	2.33958900	1.06486000
H	-21.69792600	-0.06954400	1.70346700
H S-shaped			
C	1.26483000	-0.20397500	0.06051200
C	0.70544600	-1.46771000	0.03345300
C	-0.70543500	-1.46771100	-0.03597300
C	-1.26484800	-0.20397700	-0.06244300
S	-0.00002700	1.01641700	-0.00062900
H	1.29962900	-2.37396000	0.06546900
H	-1.29959300	-2.37395800	-0.06850900
C	-2.65344500	0.17414300	-0.12814300
C	-3.21060900	1.43603300	-0.21695500
S	-3.92171100	-1.04387000	-0.09789100
C	-4.62229900	1.43586300	-0.27105700
H	-2.61387000	2.34047600	-0.25271500
C	-5.18392000	0.17381600	-0.22427800
H	-5.21521500	2.33941200	-0.35682300
C	2.65342000	0.17414700	0.12634100
C	3.21058500	1.43605800	0.21485300
S	3.92167800	-1.04389200	0.09672900
C	4.62226400	1.43589200	0.26918300
H	2.61385400	2.34052500	0.25016100
C	5.18388300	0.17382500	0.22287800

H	5.21517400	2.33946700	0.35471900
C	6.57370200	-0.20467300	0.26032000
C	7.13200200	-1.46361100	0.37686200
S	7.84101900	1.00896000	0.14585000
C	8.54494800	-1.46368900	0.39069800
H	6.53538100	-2.36412500	0.46896400
C	9.10533200	-0.20480500	0.28491200
H	9.13991900	-2.36385500	0.49557500
C	-6.57374700	-0.20468600	-0.26141300
C	-7.13206900	-1.46362500	-0.37782800
S	-7.84103800	1.00894400	-0.14665200
C	-8.54501900	-1.46370500	-0.39135200
H	-6.53546400	-2.36413300	-0.47008300
C	-9.10538000	-0.20482100	-0.28544800
H	-9.14001300	-2.36386900	-0.49610800
C	10.49608900	0.17346700	0.27256000
C	11.05806400	1.43274800	0.36112000
S	11.75824600	-1.04125900	0.12118100
C	12.47115500	1.43202400	0.32721100
H	10.46502200	2.33377800	0.46984900
C	13.02661300	0.17229100	0.21176800
H	13.07028100	2.33185200	0.40861200
C	-10.49613600	0.17345100	-0.27279600
C	-11.05813200	1.43272500	-0.36133000
S	-11.75825400	-1.04126100	-0.12099700
C	-12.47121500	1.43200300	-0.32707900
H	-10.46511600	2.33374400	-0.47028900
C	-13.02664300	0.17227900	-0.21139000
H	-13.07036200	2.33182200	-0.40842000
C	14.41688400	-0.20773400	0.15604400
C	14.98337100	-1.46152700	0.27637900
S	15.66927100	0.99757100	-0.10577200
C	16.39524300	-1.46183300	0.18357500
H	14.39702500	-2.35615900	0.45305000
C	16.94144000	-0.20965200	-0.01086100
H	17.00016100	-2.35596200	0.28306500
C	-14.41690100	-0.20774200	-0.15529800
C	-14.98342200	-1.46153400	-0.27547600
S	-15.66921400	0.99756800	0.10684800
C	-16.39527100	-1.46183500	-0.18229800
H	-14.39712600	-2.35616700	-0.45230200
C	-16.94141100	-0.20965100	0.01227600
H	-17.00021900	-2.35596100	-0.28162700
C	18.33128700	0.17057200	-0.13992300
C	18.90457200	1.42016800	-0.02748200
S	19.56453300	-1.02939700	-0.49621100
C	20.31504900	1.41801600	-0.21476400
H	18.33052900	2.31055700	0.20291000

C	20.81670200	0.17113100	-0.46804200
H	20.93122700	2.30761200	-0.15512300
H	21.84331500	-0.11919800	-0.64138700
C	-18.33122300	0.17058000	0.14170000
C	-18.90453600	1.42017400	0.02937500
S	-19.56437600	-1.02937500	0.49836000
C	-20.31496200	1.41803100	0.21703700
H	-18.33055300	2.31055300	-0.20120100
C	-20.81654900	0.17115500	0.47048900
H	-20.93115400	2.30762700	0.15753300
H	-21.84311700	-0.11916600	0.64411900
*i*Pr			
C	17.75790700	6.72648800	-0.78882300
C	17.89027000	6.20334100	0.46485000
C	17.12045400	5.01438600	0.68664400
C	16.39504400	4.65487300	-0.43679200
C	15.49296500	3.53764400	-0.68642800
C	15.38392600	2.78894200	-1.83620200
C	14.36512900	1.79022500	-1.81719100
C	13.66844400	1.78478500	-0.61829000
C	12.58576300	0.94433200	-0.12994000
C	12.38125600	0.51008900	1.16177400
C	11.19719600	-0.25764100	1.36559200
C	10.46906800	-0.40652200	0.19479300
C	9.23752800	-1.12373600	-0.09689700
C	8.92367100	-1.81199000	-1.24876700
C	7.61428700	-2.37487300	-1.28369600
C	6.90098900	-2.09851800	-0.12673400
C	5.56648800	-2.48329900	0.30532100
C	5.15868200	-2.78807700	1.58598200
C	3.77020800	-3.07698300	1.73028500
C	3.09318600	-2.97926300	0.52358300
C	1.70120700	-3.21197600	0.17175800
C	1.21308800	-3.75735300	-0.99600500
C	-0.20808800	-3.81576500	-1.09291600
C	-0.82564100	-3.28746600	0.03102900
C	-2.22695600	-3.16248600	0.39996300
C	-2.77263800	-3.28322500	1.65970200
C	-4.17690600	-3.05111500	1.73950900
C	-4.71978500	-2.73179600	0.50374500
C	-6.08461500	-2.44594200	0.09005300
C	-6.68947600	-2.78987000	-1.09954400
C	-8.02727300	-2.32286400	-1.25907300
C	-8.45381000	-1.58941200	-0.16203200
C	-9.72407800	-0.94984400	0.14392900
C	-10.32971200	-0.84386200	1.37720700
C	-11.54880500	-0.10447700	1.39476200
C	-11.87875300	0.37891100	0.13809600

C	-13.01770400	1.15358200	-0.33236800
C	-13.68132200	1.02650900	-1.53274900
C	-14.72757600	1.97211000	-1.75069900
C	-14.85655600	2.85442800	-0.69081800
C	-15.78533900	3.95290200	-0.45446500
C	-16.39638800	4.29539400	0.73022600
C	-17.19676700	5.48066400	0.67078500
C	-17.17421300	6.03528200	-0.58298600
S	16.66645500	5.79277900	-1.75249200
S	14.26958300	3.03579000	0.46191600
S	11.24988200	0.43069400	-1.14022300
S	7.85647700	-1.12150600	0.98081400
S	4.18488300	-2.50548100	-0.77162300
S	0.36586900	-2.70222200	1.18473000
S	-3.47551100	-2.70065200	-0.73897000
S	-7.18022900	-1.46758700	1.04463300
S	-10.66084800	-0.07104800	-1.04781800
S	-13.66645000	2.51645300	0.55727700
S	-16.18300800	5.13880300	-1.68894400
H	18.23488800	7.60475800	-1.20120600
H	18.53177200	6.64422300	1.21959800
H	16.05293800	2.93580400	-2.67647900
H	13.09589200	0.71456300	1.95090600
H	9.64381300	-1.94001100	-2.04891900
H	5.86149200	-2.83658600	2.40987400
H	1.87464300	-4.14381000	-1.76300800
H	-2.17075700	-3.56920700	2.51489600
H	-6.18458500	-3.40205800	-1.83830600
H	-9.91533400	-1.32274600	2.25719600
H	-13.43875500	0.23872900	-2.23721400
H	-16.28391800	3.69849900	1.62932000
H	-17.68807700	6.91937400	-0.93246400
C	-17.98367700	5.99937800	1.86061900
C	-19.16419600	5.06246100	2.18872400
C	-18.46878200	7.44357900	1.68175100
H	-17.30226700	5.98189300	2.72358700
H	-18.82867400	4.03411000	2.35310400
H	-19.68503500	5.39752700	3.09224300
H	-19.88537000	5.04960300	1.36435400
H	-17.63860800	8.12281700	1.46511200
H	-19.19344400	7.52032600	0.86309700
H	-18.96610400	7.79452700	2.59126700
C	-15.60781500	1.94478600	-2.98712200
C	-16.27828700	0.57073200	-3.17544800
C	-14.81913000	2.35249700	-4.24669500
H	-16.40703500	2.67874100	-2.84353100
H	-16.85130300	0.28408600	-2.28863900
H	-16.96263100	0.59574400	-4.03021500

H	-15.54023100	-0.21567300	-3.36707400
H	-14.38175500	3.34942400	-4.13725600
H	-14.00213400	1.64999200	-4.44545500
H	-15.47383800	2.36197100	-5.12482400
C	-12.38077500	0.06499800	2.65353900
C	-11.70651900	1.03516100	3.64335800
C	-12.68091900	-1.28827900	3.32494600
H	-13.34119100	0.50243700	2.36282800
H	-11.54239800	2.01697500	3.18927200
H	-12.32939300	1.17120000	4.53413600
H	-10.73324900	0.65212300	3.96962900
H	-13.17408000	-1.97540900	2.63099700
H	-11.76664000	-1.77254600	3.68448500
H	-13.33872400	-1.14505200	4.18878200
C	-8.86563800	-2.65525000	-2.48034800
C	-8.89589600	-4.17003400	-2.75779700
C	-8.38307800	-1.87553400	-3.71926200
H	-9.89470300	-2.34450600	-2.27394500
H	-9.25020400	-4.72658900	-1.88507800
H	-9.56547600	-4.38856000	-3.59642400
H	-7.90412900	-4.55328400	-3.02075200
H	-8.41345800	-0.79548800	-3.54691400
H	-7.35227500	-2.14304800	-3.97681500
H	-9.01458700	-2.10025300	-4.58567800
C	-4.95309200	-3.19714400	3.03627600
C	-4.64999200	-2.03556900	4.00322000
C	-4.69086100	-4.55753500	3.70940500
H	-6.01983300	-3.15712000	2.79404500
H	-4.88841000	-1.06804500	3.55136400
H	-5.23714200	-2.13663900	4.92246300
H	-3.59040600	-2.02116300	4.28163400
H	-4.91388100	-5.38644000	3.03112800
H	-3.64806100	-4.65614900	4.02946600
H	-5.31940100	-4.66647800	4.59959700
C	-0.91801200	-4.43850800	-2.28166300
C	-0.39491900	-5.85604700	-2.58131100
C	-0.81710500	-3.53776400	-3.52838800
H	-1.97812200	-4.53352900	-2.02606200
H	-0.47134400	-6.50217300	-1.70176100
H	-0.97609700	-6.31121700	-3.39029800
H	0.65347500	-5.84182100	-2.89818300
H	-1.23812000	-2.54587800	-3.33855300
H	0.22642200	-3.40383800	-3.83419000
H	-1.35908500	-3.98273200	-4.36998800
C	3.15813300	-3.48728300	3.05772300
C	3.06152400	-2.28917000	4.02280200
C	3.92450300	-4.65611800	3.70503200
H	2.14001400	-3.83831800	2.86201400

H	2.47217200	-1.47510900	3.59025200
H	2.58826700	-2.58979000	4.96395000
H	4.05518900	-1.89234300	4.25879300
H	3.98748800	-5.51342000	3.02829800
H	4.94520500	-4.36859300	3.97927700
H	3.41757000	-4.98005600	4.62010400
C	7.12001500	-3.21298800	-2.44913300
C	8.09717100	-4.35477300	-2.78722300
C	6.84250400	-2.33869600	-3.68800600
H	6.17409800	-3.67599300	-2.15071900
H	8.28887600	-4.98510300	-1.91379000
H	7.68211800	-4.98695100	-3.57936400
H	9.05958100	-3.97169800	-3.14307100
H	6.11133400	-1.55515400	-3.46764100
H	7.75847000	-1.85017500	-4.03856500
H	6.45166000	-2.94806600	-4.51015300
C	10.84385100	-0.86493700	2.71143400
C	10.40939000	0.21834800	3.71801200
C	12.00023100	-1.71189100	3.27580400
H	9.99360200	-1.53793700	2.56233200
H	9.55460500	0.78981600	3.34395400
H	10.12400800	-0.23609700	4.67303900
H	11.22312000	0.92555900	3.91350600
H	12.29826400	-2.49523100	2.57260400
H	12.88275200	-1.09959800	3.49003600
H	11.69713000	-2.19149500	4.21257000
C	14.14030800	0.83743100	-2.97777400
C	15.44256200	0.12749900	-3.39398800
C	13.49753700	1.56059200	-4.17768900
H	13.44286200	0.06239200	-2.64461200
H	15.89254100	-0.40335300	-2.54977400
H	15.24049300	-0.60083000	-4.18652300
H	16.18412100	0.83551300	-3.77917700
H	12.54341900	2.01964700	-3.90185300
H	14.15179400	2.35381900	-4.55624500
H	13.31355700	0.85764700	-4.99747800
C	17.16028500	4.24791100	1.99619400
C	16.47045600	5.03169800	3.12964200
C	18.60167800	3.86822100	2.38534500
H	16.60630500	3.31443000	1.85425300
H	15.43032900	5.26131600	2.87949900
H	16.47742400	4.45161900	4.05892000
H	16.98338400	5.98029300	3.32407500
H	19.08717700	3.29101400	1.59285500
H	19.21446400	4.75560400	2.57749800
H	18.60300900	3.26307600	3.29827200
Li			
C	21.01590600	0.15966400	-0.04845200

C	20.45797800	1.36062300	0.30732100
C	19.02220300	1.39854000	0.34778800
C	18.52806300	0.14873800	-0.00240900
C	17.13568700	-0.26492900	-0.04459900
C	16.52187200	-1.46940500	0.22406400
C	15.08730300	-1.49200200	0.23449300
C	14.58634800	-0.23124800	-0.03852100
C	13.20555800	0.19835100	-0.07131500
C	12.60358200	1.41011500	0.20128300
C	11.17063800	1.44478100	0.22649600
C	10.65547900	0.18875600	-0.04128900
C	9.27256500	-0.23406400	-0.05910700
C	8.66884500	-1.43932900	0.23750100
C	7.23624200	-1.46908500	0.27283900
C	6.72331200	-0.21598100	-0.01134400
C	5.34113800	0.20967300	-0.03094200
C	4.74121400	1.42261700	0.24043000
C	3.30861700	1.45594000	0.28083400
C	2.79287800	0.19732300	0.02926600
C	1.40983700	-0.22717900	0.02473700
C	0.80985300	-1.42904700	0.34059400
C	-0.62272700	-1.46080500	0.38135700
C	-1.13788400	-0.21222000	0.08299000
C	-2.52143100	0.20984000	0.05702100
C	-3.12422800	1.42434300	0.31351200
C	-4.55713600	1.45535800	0.34761700
C	-5.06878900	0.19287500	0.10820200
C	-6.45155500	-0.23302400	0.09752200
C	-7.05318600	-1.43153200	0.42221100
C	-8.48619600	-1.46480400	0.44635000
C	-8.99884700	-0.22073200	0.12615200
C	-10.38308900	0.19674300	0.07305100
C	-10.99345700	1.41325800	0.29918200
C	-12.42707500	1.43999200	0.30451400
C	-12.92989400	0.17258400	0.07511100
C	-14.31234800	-0.25616000	0.03846200
C	-14.92089800	-1.44690400	0.37445600
C	-16.35530000	-1.48385500	0.35080800
C	-16.85807100	-0.25182200	-0.01825000
C	-18.24655200	0.15241900	-0.13949000
C	-18.88352700	1.35670800	0.04793400
C	-20.32960300	1.35836400	-0.03710900
C	-20.77602200	0.08692400	-0.28416300
S	19.80579700	-1.04055600	-0.37668400
S	15.89278500	0.95423000	-0.40964100
S	11.94998800	-1.00669300	-0.43075500
S	8.01867600	0.96964300	-0.42867600
S	4.08429600	-1.00002400	-0.36559700

S	0.15372000	0.97024800	-0.35330800
S	-3.77383800	-1.00641500	-0.26691800
S	-7.70425900	0.95827000	-0.30837900
S	-11.62528400	-1.02854000	-0.25411900
S	-15.55494800	0.92122200	-0.43653900
S	-19.45831600	-1.08528300	-0.51890500
H	22.06438200	-0.09441100	-0.14444500
H	21.08385100	2.21791200	0.54559900
H	17.12869100	-2.33605600	0.47460000
H	13.21789500	2.27461100	0.44332900
H	9.28236100	-2.30119500	0.49083000
H	5.35711600	2.28948300	0.46997800
H	1.42573900	-2.28673300	0.60241800
H	-2.51090300	2.29509300	0.53513600
H	-6.43926100	-2.28516300	0.70119100
H	-10.38712900	2.28957500	0.51768600
H	-14.31468400	-2.29172200	0.69356500
H	-18.30394200	2.24383600	0.29359200
H	-21.78414400	-0.28724800	-0.41980900
Li	-21.62739300	2.83840200	0.07562800
Li	-17.98349000	-2.53551600	0.83128000
Li	-14.06878200	2.49145000	0.71913000
Li	-10.10705000	-2.50865900	0.96508600
Li	-6.18558200	2.51168800	0.81163200
Li	-2.24291000	-2.49949600	0.91043800
Li	1.68357400	2.50487300	0.77059900
Li	5.61318200	-2.50579000	0.79292800
Li	9.54381300	2.48495700	0.72203600
Li	13.46222500	-2.53469600	0.72501200
Li	17.38518900	2.37010900	0.89823500
*n*Bu			
C	19.93315000	-0.87173400	4.84717300
C	19.83196900	-1.94821500	4.01439500
C	18.72002300	-1.88820800	3.11247500
C	17.97570400	-0.73347100	3.28715600
C	16.78425900	-0.23899700	2.61096200
C	16.49543500	1.06522100	2.27333200
C	15.21974900	1.26977100	1.67352400
C	14.50892900	0.08156100	1.54994100
C	13.21089400	-0.20386400	0.97239500
C	12.76997100	-1.40774900	0.45776700
C	11.42351500	-1.42017600	0.00575000
C	10.80510600	-0.18415100	0.17501800
C	9.47749800	0.27781400	-0.16841200
C	9.05461100	1.58727300	-0.30676000
C	7.67620400	1.75511300	-0.60316300
C	7.01237700	0.53574100	-0.70214200
C	5.63848500	0.21424100	-1.02396900

C	5.15431100	-0.97650400	-1.53306200
C	3.74626300	-1.03772700	-1.70574000
C	3.12167100	0.14509300	-1.31959700
C	1.73332000	0.55131800	-1.35539500
C	1.23616000	1.84149200	-1.36611900
C	-0.17927100	1.94946800	-1.34791200
C	-0.79813100	0.70189200	-1.32644700
C	-2.19367300	0.32260000	-1.34741900
C	-2.72063700	-0.91075600	-1.68545100
C	-4.13068600	-1.02378800	-1.57188700
C	-4.71629700	0.16285600	-1.13775000
C	-6.10017300	0.52597000	-0.92435000
C	-6.63444200	1.80044600	-0.87006200
C	-8.02728900	1.86702800	-0.60256700
C	-8.58988200	0.60313100	-0.44816000
C	-9.95453800	0.18180600	-0.21316000
C	-10.50565100	-1.05788700	-0.47932800
C	-11.86616000	-1.21398800	-0.10154100
C	-12.38296900	-0.05713700	0.47195600
C	-13.71052300	0.26127600	0.95923500
C	-14.28312500	1.51172800	1.07946500
C	-15.59036500	1.53094500	1.64318700
C	-16.03460200	0.26046000	1.97013600
C	-17.29598900	-0.20937000	2.52865100
C	-18.01760300	-1.32098400	2.15550200
C	-19.19107700	-1.55916800	2.93891000
C	-19.34170900	-0.61258100	3.91843400
S	18.65390300	0.26033100	4.57367300
S	15.42127900	-1.26466500	2.21943600
S	11.90621400	0.96464700	0.92403800
S	8.11264600	-0.79993600	-0.39267200
S	4.29970200	1.30382400	-0.71786400
S	0.39992400	-0.58537900	-1.29585300
S	-3.49154500	1.39090900	-0.84746600
S	-7.36453100	-0.64736500	-0.60865700
S	-11.15590200	1.19782900	0.56050500
S	-14.81530600	-0.94648200	1.59075600
S	-18.06330100	0.55991200	3.91124300
H	20.67712700	-0.68056200	5.60798100
H	20.53391600	-2.77516700	4.02972800
H	17.21234800	1.86159200	2.43025700
H	13.42456000	-2.26696200	0.38356900
H	9.74312600	2.41890800	-0.22174600
H	5.81741100	-1.78755100	-1.80729900
H	1.89151900	2.70209400	-1.41489000
H	-2.09258900	-1.71932900	-2.03808300
H	-6.02811500	2.67920400	-1.05131200
H	-9.93798500	-1.83820600	-0.97084200

H	-13.78019100	2.41192300	0.74212000
H	-17.72628600	-1.94150800	1.31462200
H	-20.14383300	-0.52791200	4.63934300
C	-20.13423000	-2.71479300	2.71256700
C	-19.56147200	-4.07417100	3.15757500
H	-20.39385800	-2.76919100	1.64650900
H	-21.07305800	-2.52673400	3.24721900
C	-20.52333800	-5.24173700	2.90807000
H	-18.61519900	-4.26024900	2.63201000
H	-19.30982700	-4.02316600	4.22502600
C	-19.96043400	-6.59280800	3.35881400
H	-21.47070100	-5.04746200	3.42934000
H	-20.77028200	-5.28534300	1.83844200
H	-20.66921100	-7.40515400	3.16835500
H	-19.03096100	-6.83170600	2.82972300
H	-19.73713800	-6.59076600	4.43160400
C	-16.38945300	2.80824500	1.75237000
C	-16.83523200	3.36013900	0.38314500
H	-15.78289900	3.56788700	2.26383500
H	-17.27423400	2.65195300	2.37463700
C	-17.62684700	4.66847800	0.49545800
H	-15.95484600	3.51812100	-0.25384800
H	-17.44541000	2.60153400	-0.12406300
C	-18.07611900	5.21706400	-0.86214900
H	-18.50528700	4.50572200	1.13425900
H	-17.01310000	5.42044500	1.01015700
H	-18.63777700	6.15018100	-0.75066600
H	-17.21703500	5.42136800	-1.51114600
H	-18.72058500	4.50120300	-1.38463500
C	-12.65845800	-2.48364600	-0.33288500
C	-12.06535900	-3.44915700	-1.36951800
H	-13.67738400	-2.21720700	-0.63955900
H	-12.77178600	-3.02160300	0.61961100
C	-12.96185700	-4.66793100	-1.62088800
H	-11.90024900	-2.91634500	-2.31539400
H	-11.07948000	-3.79736900	-1.03444400
C	-12.37175500	-5.64718700	-2.63968800
H	-13.13886400	-5.18984600	-0.67069300
H	-13.94672900	-4.32704700	-1.96734400
H	-13.03511400	-6.50278100	-2.80156300
H	-12.21180900	-5.16205400	-3.60919900
H	-11.40439100	-6.03597700	-2.30197100
C	-8.80470000	3.16429700	-0.52959300
C	-8.10851000	4.38746000	-1.14383400
H	-9.04170300	3.38826000	0.52079100
H	-9.77477500	3.02605900	-1.02310100
C	-8.99070100	5.64176800	-1.11446900
H	-7.17512100	4.59711600	-0.60492700

H	-7.82420700	4.16577800	-2.18105800
C	-8.30634400	6.87213100	-1.71679600
H	-9.92433500	5.43940700	-1.65624100
H	-9.28228200	5.85532700	-0.07723800
H	-8.96006300	7.74949800	-1.68223500
H	-7.38746800	7.12098900	-1.17385900
H	-8.03420500	6.70131600	-2.76449800
C	-4.90019300	-2.28218900	-1.91358700
C	-4.11541400	-3.34888100	-2.69038600
H	-5.79259000	-2.00908400	-2.49113700
H	-5.28081800	-2.73708700	-0.98758900
C	-4.97954100	-4.56123100	-3.05882500
H	-3.69890600	-2.90715700	-3.60544800
H	-3.25950000	-3.69046800	-2.09352900
C	-4.20460600	-5.64237100	-3.81771800
H	-5.40813200	-4.99175000	-2.14372200
H	-5.83225600	-4.22685400	-3.66480400
H	-4.84639700	-6.49346100	-4.06678400
H	-3.79261900	-5.25110200	-4.75481200
H	-3.36654200	-6.02120000	-3.22183500
C	-0.91849500	3.27051200	-1.38002200
C	-0.06764600	4.49717900	-1.73827300
H	-1.38841300	3.44711200	-0.40162300
H	-1.74997100	3.19717800	-2.09267500
C	-0.89737500	5.78462400	-1.81759600
H	0.72662100	4.63113100	-0.99207900
H	0.43558000	4.32828600	-2.69961500
C	-0.05897300	7.01900500	-2.16177100
H	-1.69001500	5.65831500	-2.56728400
H	-1.40952500	5.94480600	-0.85918700
H	-0.67789200	7.92043100	-2.21319700
H	0.71888800	7.19235800	-1.40955500
H	0.43941500	6.90210400	-3.13070200
C	3.02555100	-2.24024800	-2.27786200
C	3.90989800	-3.23684500	-3.04125900
H	2.22767300	-1.89258200	-2.94570900
H	2.51317400	-2.77833700	-1.46699500
C	3.10267200	-4.38037300	-3.66808400
H	4.46466500	-2.70764900	-3.82736100
H	4.66254900	-3.66345800	-2.36528100
C	3.97516200	-5.38898700	-4.42087400
H	2.53849000	-4.89914800	-2.88113100
H	2.35270200	-3.96120400	-4.35219600
H	3.37179600	-6.19133800	-4.85727300
H	4.52601400	-4.90611400	-5.23587400
H	4.71149900	-5.85193700	-3.75413900
C	7.02360200	3.10448400	-0.81730600
C	7.98622800	4.26332900	-1.11531200

H	6.42714100	3.36545700	0.06910900
H	6.30223000	3.02445800	-1.63986700
C	7.25157000	5.57702300	-1.40964400
H	8.66098000	4.41781500	-0.26306100
H	8.62282100	4.00089700	-1.97062900
C	8.20004000	6.74341300	-1.70144000
H	6.57728900	5.43068900	-2.26411600
H	6.60863200	5.83141600	-0.55618500
H	7.64719000	7.66485400	-1.91027000
H	8.86265100	6.93924200	-0.85074300
H	8.83303700	6.53115200	-2.57040200
C	10.76083500	-2.63265200	-0.61386200
C	11.70919400	-3.77160100	-1.01526300
H	10.19366000	-2.31685700	-1.49881600
H	10.01176500	-3.03387900	0.08408100
C	10.97639900	-4.93279500	-1.69852100
H	12.48568000	-3.38431400	-1.68829500
H	12.23061700	-4.15303200	-0.12750200
C	11.90855600	-6.08123900	-2.09504400
H	10.19316500	-5.31112500	-1.02774300
H	10.45641900	-4.55748900	-2.59016600
H	11.35716700	-6.89362800	-2.57907100
H	12.68134300	-5.74156200	-2.79375700
H	12.41676900	-6.50027600	-1.21928200
C	14.72449500	2.62043900	1.20054800
C	15.81558600	3.67890700	0.98032000
H	13.99609900	3.01615300	1.92357700
H	14.16502300	2.48889500	0.26637700
C	15.25773700	4.99269900	0.41911700
H	16.32940400	3.88812400	1.92770100
H	16.57774300	3.28338000	0.29593400
C	16.33519500	6.06096400	0.21133600
H	14.75042500	4.79256400	-0.53424100
H	14.48534800	5.37769700	1.09864300
H	15.90925200	6.98459900	-0.19337400
H	16.83366700	6.31032900	1.15483800
H	17.10555800	5.71575000	-0.48736900
C	18.48290300	-2.95598600	2.07082700
C	19.60276100	-3.03863000	1.01417100
H	17.53089700	-2.78518500	1.56180500
H	18.39277100	-3.93098100	2.56999200
C	19.36384600	-4.14497200	-0.02006800
H	19.68647100	-2.06908100	0.50623400
H	20.56792000	-3.20595500	1.51057100
C	20.47334000	-4.23533600	-1.07195400
H	19.26931900	-5.10960900	0.49726900
H	18.39994300	-3.97263200	-0.51763100
H	20.27454600	-5.03255800	-1.79546800

H	20.56834200	-3.29662400	-1.62923900
H	21.44425300	-4.44231000	-0.60766700
NH_2			
C	20.66927200	-0.39474200	2.45528400
C	20.30219800	-1.58101800	1.89199500
C	18.96491900	-1.57509300	1.37703200
C	18.32557500	-0.35371200	1.56684700
C	17.00252200	0.07010000	1.17755200
C	16.55151900	1.35702600	0.97868200
C	15.17626400	1.45544100	0.62213200
C	14.54677200	0.21462100	0.53754200
C	13.19931400	-0.12049500	0.16418600
C	12.71882100	-1.33570100	-0.28087200
C	11.32245900	-1.35867400	-0.55234500
C	10.70037500	-0.13312200	-0.31596500
C	9.33496100	0.26910200	-0.50946700
C	8.83586400	1.55139100	-0.62743900
C	7.42525100	1.62680400	-0.79342600
C	6.80907600	0.37558300	-0.80773300
C	5.43125800	0.01912200	-0.99996900
C	4.91571000	-1.20485400	-1.37945200
C	3.49694200	-1.25095800	-1.46561600
C	2.89008800	-0.03570900	-1.14865700
C	1.50528900	0.34384600	-1.16314200
C	0.97364300	1.61770700	-1.21417900
C	-0.44748000	1.66956200	-1.19846800
C	-1.04034900	0.40862300	-1.13519100
C	-2.42525600	0.02945900	-1.15086200
C	-2.96458500	-1.20569100	-1.45373400
C	-4.38223300	-1.27350100	-1.36326200
C	-4.96463300	-0.06442000	-0.98415000
C	-6.34687300	0.29561200	-0.83351800
C	-6.90105400	1.56058600	-0.83884100
C	-8.31033700	1.59285900	-0.64857200
C	-8.87054900	0.32561500	-0.49224300
C	-10.24171600	-0.07249800	-0.33222600
C	-10.79614200	-1.31874500	-0.54784600
C	-12.19215000	-1.40192200	-0.28542000
C	-12.74097700	-0.19436700	0.14141400
C	-14.10277700	0.15432500	0.44568800
C	-14.67278100	1.41092400	0.46993100
C	-16.05186800	1.43080000	0.82508900
C	-16.56789600	0.16440900	1.07962000
C	-17.91255300	-0.24638600	1.40366500
C	-18.49309000	-1.48319100	1.21780200
C	-19.84627800	-1.57153500	1.67362100
C	-20.29970500	-0.38742800	2.20115800
S	19.39738500	0.77578700	2.39658700

S	15.68153300	-1.07093100	0.95372800
S	11.87530900	1.03414900	0.29488400
S	8.01180300	-0.89353800	-0.56238200
S	4.11578200	1.15161900	-0.69456700
S	0.20498700	-0.84039600	-1.04684600
S	-3.71113900	1.14489000	-0.69346800
S	-7.60397100	-0.90410200	-0.53791600
S	-11.47860900	1.03363000	0.26239500
S	-15.29737200	-1.04949500	0.92473400
S	-19.05854800	0.83391100	2.20004700
H	21.61596600	-0.14298200	2.91222700
H	20.95236300	-2.44750500	1.83691700
H	17.19900400	2.22353500	1.06016000
H	13.35903700	-2.19729600	-0.43682100
H	9.47176400	2.43010300	-0.62182000
H	5.54407000	-2.05576200	-1.61954300
H	1.59024100	2.50696700	-1.28886000
H	-2.35742800	-2.04974700	-1.76277900
H	-6.31272200	2.45690600	-1.00336200
H	-10.21918200	-2.16134000	-0.91369500
H	-14.12163700	2.30904400	0.21222300
H	-17.97067400	-2.31273900	0.75357500
H	-21.27916200	-0.16279900	2.59864200
N	18.39443900	-2.70419900	0.79586300
H	17.64677800	-2.52094200	0.13731000
H	19.06576500	-3.36383300	0.42600600
N	14.55828800	2.68212200	0.40827700
H	15.19492800	3.43851300	0.19679100
H	13.76465600	2.66748700	-0.22085000
N	10.67644700	-2.51006000	-0.98810500
H	9.84335900	-2.35913200	-1.54412600
H	11.28831500	-3.19952500	-1.40325300
N	6.76187400	2.84344800	-0.90691700
H	7.35115500	3.61399800	-1.19166200
H	5.89252200	2.81968400	-1.42615900
N	2.81838000	-2.41332100	-1.81516300
H	1.91722600	-2.27571800	-2.25675000
H	3.38198200	-3.09117300	-2.31016500
N	-1.13979200	2.87540400	-1.22533200
H	-0.60322500	3.65463200	-1.58180000
H	-2.06645800	2.83678700	-1.63256200
N	-5.08041000	-2.44962900	-1.61662100
H	-6.03189300	-2.32860000	-1.94248300
H	-4.57268500	-3.12148100	-2.17614700
N	-9.01986800	2.78912300	-0.60921600
H	-8.54552900	3.56647600	-1.04860400
H	-9.98937700	2.72811700	-0.89663000
N	-12.89874300	-2.59215400	-0.43355400

H	-13.88650900	-2.48576300	-0.63221400
H	-12.46026100	-3.25825400	-1.05529200
N	-16.77064200	2.62123800	0.91868300
H	-16.38430900	3.37798100	0.36999200
H	-17.76978000	2.52927100	0.77618900
N	-20.57514400	-2.76847600	1.61440200
H	-21.57545300	-2.63810300	1.69636400
H	-20.36207800	-3.32821100	0.79795100
NMe_2			
C	21.33796800	-0.03096100	1.02590600
C	20.84433100	-1.29799900	0.92957900
C	19.42100800	-1.33392100	0.76460900
C	18.84267900	-0.07558900	0.73843900
C	17.45042200	0.28767900	0.59656100
C	16.91119600	1.55502500	0.56255400
C	15.49630400	1.58248400	0.41717500
C	14.93396400	0.31812400	0.33887500
C	13.54533200	-0.04658900	0.20143100
C	13.01306000	-1.31623000	0.11154800
C	11.59700300	-1.34771200	-0.00697900
C	11.02250200	-0.08579400	-0.00857700
C	9.62966300	0.27324300	-0.10517400
C	9.08575400	1.54157000	-0.10287300
C	7.66902800	1.56864600	-0.20873600
C	7.10460900	0.30480600	-0.29407100
C	5.71352500	-0.05744000	-0.39955300
C	5.17969500	-1.32587100	-0.50547200
C	3.76127100	-1.35743200	-0.58510700
C	3.18532600	-0.09617100	-0.53840500
C	1.78998100	0.26336400	-0.56455700
C	1.24763200	1.53262200	-0.55244600
C	-0.17289200	1.55998600	-0.55391200
C	-0.74333900	0.29616300	-0.57125500
C	-2.13853300	-0.06511200	-0.57634600
C	-2.67938000	-1.33488100	-0.60007300
C	-4.10004700	-1.36467300	-0.59612600
C	-4.67082600	-0.10027400	-0.56661100
C	-6.06456900	0.26338600	-0.52285700
C	-6.60360500	1.53413000	-0.54528900
C	-8.02159500	1.56939700	-0.46003600
C	-8.59321900	0.30786300	-0.37234700
C	-9.98338100	-0.05145400	-0.24721500
C	-10.52725200	-1.31974700	-0.23002300
C	-11.93812100	-1.34823300	-0.05921900
C	-12.49977700	-0.08542700	0.05364700
C	-13.88245300	0.27910900	0.24087700
C	-14.41471900	1.54903000	0.32025700
C	-15.82323300	1.58139300	0.51836200

C	-16.39145700	0.31963000	0.59092200
C	-17.77667800	-0.04425700	0.79415300
C	-18.30837400	-1.31508000	0.80336100
C	-19.71737200	-1.36538900	1.06162500
C	-20.23820400	-0.10196600	1.23846300
S	20.07770900	1.16082500	0.91701600
S	16.17829100	-0.92284100	0.44179500
S	12.25852000	1.15938800	0.13610300
S	8.35225900	-0.93666900	-0.24538000
S	4.42449200	1.14768000	-0.40490900
S	0.50409200	-0.94636600	-0.58509800
S	-3.42417800	1.14342900	-0.55539700
S	-7.35068000	-0.93905800	-0.40442700
S	-11.25691700	1.15631700	-0.05668800
S	-15.16120200	-0.92501000	0.40889800
S	-19.03066000	1.14598700	1.11886500
H	22.36336900	0.28623100	1.15336700
H	21.46395600	-2.18634800	0.97366200
H	17.51352000	2.45353000	0.64022600
H	13.62411500	-2.21183400	0.13133900
H	9.68964600	2.43875800	-0.02476000
H	5.79310000	-2.21958100	-0.52721300
H	1.85601900	2.43014100	-0.54023200
H	-2.06918800	-2.23081100	-0.61949000
H	-5.99362500	2.42693300	-0.62316500
H	-9.92691800	-2.21608000	-0.33882400
H	-13.80943000	2.44457800	0.23616200
H	-17.69670100	-2.19543700	0.64965900
H	-21.26408800	0.17644400	1.42549000
N	18.62824400	-2.51297000	0.63179300
N	14.69299900	2.76051300	0.34603700
N	10.80050500	-2.52808200	-0.11886700
N	6.86167000	2.74644100	-0.23437200
N	2.96473100	-2.53441300	-0.71719500
N	-0.97936100	2.73893800	-0.54079100
N	-4.90449100	-2.54282200	-0.63026000
N	-8.82278800	2.74980500	-0.47717300
N	-12.74286900	-2.52740300	-0.01485200
N	-16.61472500	2.76509700	0.62083600
N	-20.43581400	-2.56987100	1.16843000
C	18.85654000	-3.23205600	-0.62253800
H	18.10793700	-4.02455000	-0.72354600
H	18.74370200	-2.54420500	-1.46364700
H	19.85662000	-3.69593200	-0.68317300
C	18.67887300	-3.39079900	1.80109900
H	19.66061300	-3.87596000	1.94250200
H	18.44560200	-2.81562100	2.70005200
H	17.92610200	-4.17811700	1.69122200

C	10.86091400	-3.39147100	1.06054400
H	11.84349800	-3.87589500	1.19903000
H	10.63728400	-2.80453100	1.95435300
H	10.10562100	-4.17878400	0.96801200
C	11.01641700	-3.26260100	-1.36571200
H	10.26431000	-4.05375500	-1.45151600
H	10.89921500	-2.58427400	-2.21400700
H	12.01437400	-3.73080500	-1.42977900
C	6.97711900	3.56771300	0.97017800
H	6.80690800	2.94703600	1.85291900
H	7.96057300	4.05896000	1.07432300
H	6.21051700	4.34917000	0.94620000
C	7.00779700	3.52673200	-1.46389400
H	6.84943200	2.87818100	-2.32861700
H	6.24827900	4.31527300	-1.48214900
H	7.99920600	4.00268700	-1.56242900
C	14.78266500	3.60989700	1.53336400
H	14.59154900	3.01050000	2.42647000
H	15.76407700	4.10279600	1.64790900
H	14.01817800	4.39136000	1.47338100
C	14.86863600	3.51149000	-0.89816200
H	14.72675000	2.84355600	-1.75088100
H	14.11253300	4.30162300	-0.95108800
H	15.86353100	3.98207200	-0.98572900
C	3.23543400	-3.55794400	0.29006100
H	4.21670800	-4.04955300	0.16751100
H	3.19580900	-3.11097900	1.28603800
H	2.46368000	-4.33226100	0.22889400
C	2.95213800	-3.07624700	-2.07778100
H	2.20125500	-3.87054900	-2.14274700
H	2.67872400	-2.28809000	-2.78290700
H	3.92636000	-3.49574100	-2.38390300
C	-0.84399200	3.52780800	0.68377000
H	-1.00720700	2.88491500	1.55178800
H	0.14473600	4.00833900	0.78756300
H	-1.60572700	4.31441400	0.69051000
C	-0.84983400	3.55097400	-1.75067600
H	-1.01850200	2.92497500	-2.63000100
H	-1.61070600	4.33835500	-1.73819600
H	0.13883600	4.03250200	-1.85073000
C	-4.55542200	-3.53897200	0.37972000
H	-3.58879100	-4.03888100	0.19155300
H	-4.51261400	-3.06459700	1.36283000
H	-5.33140200	-4.31116900	0.40327200
C	-5.03148300	-3.12265200	-1.96907500
H	-5.79299700	-3.90930800	-1.95079400
H	-5.35317700	-2.35157900	-2.67261800
H	-4.08897600	-3.56234000	-2.33965000

C	-8.39491700	3.78260900	0.46278200
H	-8.27104500	3.34554500	1.45622700
H	-7.44811200	4.27464100	0.17770000
H	-9.16818000	4.55578200	0.52050600
C	-9.05323700	3.27826800	-1.82351100
H	-9.43402600	2.48345000	-2.46861900
H	-9.80698300	4.07111500	-1.77601100
H	-8.14061500	3.69526600	-2.28414500
C	-12.35485400	-3.47108500	1.03151200
H	-11.38470300	-3.96402200	0.84264700
H	-12.29507300	-2.94962500	1.98958400
H	-13.11845000	-4.25192700	1.11174700
C	-12.90666700	-3.16954700	-1.31959300
H	-13.66110700	-3.95936500	-1.24068400
H	-13.25666300	-2.43326600	-2.04676600
H	-11.97430500	-3.62127600	-1.70188400
C	-20.14022900	-3.60289400	0.18186200
H	-20.50543500	-3.35297600	-0.82921900
H	-19.06544800	-3.77968000	0.11904800
H	-20.61116100	-4.53977300	0.49444200
C	-21.85262500	-2.43619200	1.45651300
H	-22.27649100	-3.42747500	1.63937100
H	-21.99129700	-1.83549100	2.35999300
H	-22.42399700	-1.96364500	0.63721200
C	-16.13624200	3.72058200	1.61616600
H	-15.98495500	3.20991300	2.56999500
H	-15.19104900	4.21797800	1.33393500
H	-16.89349500	4.49874300	1.75862100
C	-16.89314400	3.39154000	-0.67219300
H	-17.31412600	2.64921000	-1.35398800
H	-17.63139700	4.18872100	-0.53571800
H	-15.99510400	3.82924100	-1.14311700
NO_2			
C	19.77569200	4.16709600	-2.22683100
C	19.59406800	4.39805400	-0.89949700
C	18.43911100	3.73236400	-0.39475500
C	17.73901900	2.96843100	-1.32562600
C	16.58873100	2.07914800	-1.28568000
C	16.33307100	1.10959600	-2.23129000
C	15.09506100	0.45594300	-2.04838400
C	14.36692100	0.88284200	-0.93873900
C	13.13016000	0.47021100	-0.29769900
C	12.82815100	0.71535200	1.02590700
C	11.51485500	0.33990500	1.38091400
C	10.76876500	-0.22987200	0.34944800
C	9.45484200	-0.84059500	0.24608500
C	9.09893700	-1.74281000	-0.73513200
C	7.73247100	-2.09362100	-0.70648400

C	6.99412600	-1.49301200	0.31321900
C	5.62987500	-1.60592100	0.79884300
C	5.23761900	-1.27757500	2.08031400
C	3.84194700	-1.34784600	2.27570800
C	3.11338000	-1.75716100	1.15883100
C	1.71873200	-2.06706100	0.89696600
C	1.29038900	-2.88386900	-0.12916300
C	-0.11361300	-2.92960400	-0.26197200
C	-0.81631500	-2.16958000	0.67334500
C	-2.21892900	-1.97795900	0.99811000
C	-2.67415800	-1.55206000	2.22904200
C	-4.06546100	-1.31947600	2.26393900
C	-4.73410800	-1.58240400	1.06815100
C	-6.12367200	-1.59019600	0.64603200
C	-6.59777600	-2.31205100	-0.43003700
C	-7.95420000	-2.05746900	-0.72376100
C	-8.57871200	-1.14954600	0.13161700
C	-9.93410300	-0.65382800	0.29343300
C	-10.43041800	-0.13554200	1.47156100
C	-11.72865400	0.40376600	1.34510800
C	-12.28572300	0.29867000	0.07037100
C	-13.57687600	0.60912300	-0.51797300
C	-14.06801300	0.00895000	-1.65883000
C	-15.28077200	0.57202400	-2.11192500
C	-15.77527600	1.60969000	-1.32291400
C	-16.98714600	2.41404700	-1.31784300
C	-17.52870800	2.99058000	-0.18349700
C	-18.62333100	3.83290900	-0.48215300
C	-18.92533700	3.91668600	-1.81760900
S	18.54285900	3.13841100	-2.87580700
S	15.26112300	2.15489800	-0.13659800
S	11.74860900	-0.26329800	-1.09900300
S	8.05349600	-0.44663700	1.23112700
S	4.22282200	-2.02912100	-0.16578300
S	0.33251900	-1.36508100	1.71890100
S	-3.56619400	-2.10605700	-0.12404000
S	-7.41132600	-0.59360100	1.30927300
S	-11.12712100	-0.47670800	-0.98560900
S	-14.66820500	1.88679600	-0.00004500
S	-17.88396600	2.92887200	-2.75032600
H	20.55424100	4.54677300	-2.87328000
H	20.22929500	5.01096600	-0.27604600
H	17.00069400	0.84184100	-3.03848300
H	13.51296500	1.13729100	1.74874900
H	9.77967800	-2.17074800	-1.45826000
H	5.91009600	-1.00672600	2.88276400
H	1.94032800	-3.45938800	-0.77405600
H	-2.05478600	-1.41782200	3.10530000

H	-6.01757000	-3.02381200	-1.00120700
H	-9.90880400	-0.14326700	2.41888000
H	-13.60435900	-0.82479900	-2.16825500
H	-17.18208200	2.81570700	0.82599200
H	-19.73074600	4.48048500	-2.26503000
N	-19.38067300	4.53740500	0.53662500
O	-20.31488300	5.24591300	0.16149000
O	-19.03149500	4.37689300	1.70634200
N	-15.91775600	0.02079500	-3.28740100
O	-16.82455400	0.67219500	-3.81641000
O	-15.50651100	-1.06198500	-3.70120100
N	-12.37266800	0.96505300	2.51135100
O	-13.39213300	1.64350900	2.34220600
O	-11.85785900	0.74706700	3.60614100
N	-8.57922900	-2.76043500	-1.82175400
O	-9.67307800	-2.35029900	-2.22643500
O	-7.97626300	-3.71799100	-2.30147900
N	-4.67351800	-0.88064100	3.50009900
O	-5.82987100	-0.44503700	3.45558500
O	-3.99733600	-0.95056800	4.52385700
N	-0.69440000	-3.75960900	-1.29349800
O	-1.88969500	-3.59686200	-1.56515100
O	0.04039700	-4.56943500	-1.85407500
N	3.29857500	-1.03946200	3.57963100
O	2.07896800	-0.85762900	3.67190900
O	4.08524000	-0.95895100	4.52011600
N	7.23045200	-3.04176700	-1.67576400
O	6.00511400	-3.14415300	-1.80693000
O	8.05363100	-3.68033200	-2.32728500
N	11.07319600	0.53178700	2.74420800
O	9.86262900	0.44224800	2.98015500
O	11.92741500	0.79019300	3.58874900
N	14.71146400	-0.58998100	-2.96894600
O	13.54007700	-0.98505900	-2.94550600
O	15.57142400	-1.01662900	-3.73712600
N	18.09952100	3.87633000	1.00117900
O	16.97919900	3.50455200	1.37216700
O	18.94267400	4.37531900	1.74344300
OH			
C	20.46813700	2.28792300	-1.93812400
C	20.12705500	2.76022700	-0.70442600
C	18.82905000	2.33147900	-0.29679700
C	18.18649400	1.53415600	-1.23239000
C	16.88878700	0.90613600	-1.15399600
C	16.45789200	-0.25634200	-1.75022400
C	15.10843800	-0.58245400	-1.45110700
C	14.47568300	0.32587800	-0.61345200
C	13.14375500	0.28946200	-0.06715000

C	12.67684500	0.83183100	1.10930800
C	11.29644000	0.59191500	1.33719900
C	10.67199800	-0.14133800	0.33670200
C	9.31298100	-0.60868700	0.25727500
C	8.81515800	-1.70530400	-0.41106000
C	7.41343600	-1.87201600	-0.26457600
C	6.80271700	-0.90390000	0.52189500
C	5.42510700	-0.78836500	0.92286600
C	4.89988700	-0.20244600	2.05323400
C	3.48502300	-0.28444900	2.13069000
C	2.89147600	-0.93571800	1.05726500
C	1.50438900	-1.24412300	0.82734900
C	0.95984700	-2.27302300	0.09125000
C	-0.45936200	-2.27973300	0.08713900
C	-1.03680700	-1.25286500	0.82230900
C	-2.42737300	-0.98223800	1.07871000
C	-2.99878800	-0.34672600	2.15864400
C	-4.41389200	-0.26290400	2.08522500
C	-4.96053000	-0.83370600	0.94347300
C	-6.34202400	-0.97571000	0.56352900
C	-6.92363100	-1.93537000	-0.23471900
C	-8.32502400	-1.77137300	-0.39100000
C	-8.85026600	-0.67911400	0.28631700
C	-10.21839700	-0.24226200	0.39402200
C	-10.82475000	0.45663800	1.41365500
C	-12.20352400	0.71002900	1.18739100
C	-12.68540300	0.20895900	-0.01378300
C	-14.02634200	0.23173900	-0.54168100
C	-14.63387400	-0.66203800	-1.39389000
C	-15.98161100	-0.32997900	-1.69823100
C	-16.43538300	0.82660300	-1.08407600
C	-17.74966000	1.42586700	-1.12481800
C	-18.39771200	2.15669700	-0.15763800
C	-19.69908300	2.58871400	-0.55452700
C	-20.05293700	2.18459200	-1.81551800
S	19.21889000	1.31376000	-2.64031100
S	15.57886800	1.64385100	-0.22979200
S	11.82828400	-0.49697700	-0.94359800
S	8.00353800	0.27231900	1.04883400
S	4.12937900	-1.41319400	-0.10115900
S	0.22597700	-0.22413200	1.49278400
S	-3.66987500	-1.44492300	-0.08725300
S	-7.55273800	0.19368800	1.09618000
S	-11.37186600	-0.55130900	-0.90640200
S	-15.13342100	1.54835200	-0.14535700
S	-18.75672500	1.30169400	-2.57119200
H	21.38611000	2.45662100	-2.48326300
H	20.75765500	3.38418100	-0.08366000

H	17.08819600	-0.89596700	-2.35539400
H	13.29974000	1.35265400	1.82577300
H	9.42685900	-2.40978800	-0.96069400
H	5.49754000	0.23920100	2.84084000
H	1.54313500	-3.03945600	-0.40368700
H	-2.44117400	0.01703700	3.01268900
H	-6.38637500	-2.76871200	-0.67011700
H	-10.32489600	0.75036900	2.32829800
H	-14.16023500	-1.56142500	-1.76736700
H	-17.98730600	2.36243200	0.82325300
H	-20.97952800	2.35373900	-2.34535900
O	-20.45275000	3.31333500	0.31891600
H	-21.29979800	3.52949300	-0.09217000
O	-12.92260300	1.36262500	2.13619900
H	-13.87118700	1.28609800	1.94479600
O	-5.09776000	0.31172800	3.10725300
H	-6.04464800	0.11370300	3.02790200
O	2.85010000	0.22608900	3.21634600
H	1.93228200	-0.08830600	3.24482500
O	10.72476700	1.04722600	2.48100700
H	9.85549000	0.63435100	2.60726900
O	18.35227600	2.70731600	0.91957600
H	17.56186400	2.18969500	1.14033700
O	14.57483300	-1.72387000	-1.95613300
H	13.73699700	-1.92625000	-1.50993300
O	6.80838900	-2.93700500	-0.84920800
H	5.90947400	-3.04400100	-0.49902000
O	-1.11523400	-3.26545900	-0.57646500
H	-2.05243000	-3.27144400	-0.32401700
O	-9.02003200	-2.67414400	-1.12893100
H	-9.97288000	-2.56143000	-0.98185900
O	-16.70253300	-1.15140500	-2.50426800
H	-17.64079700	-0.90434100	-2.47110600
OMe			
C	-21.20394600	-0.04721100	0.00004800
C	-20.70645100	-1.32016300	0.00004500
C	-19.27851500	-1.34290700	0.00003600
C	-18.69411700	-0.08275200	0.00003300
C	-17.29938900	0.27422000	0.00002500
C	-16.76008900	1.54850100	0.00002300
C	-15.34380200	1.55878800	0.00001500
C	-14.76603000	0.29335400	0.00001200
C	-13.37766300	-0.06814200	0.00000500
C	-12.84212800	-1.34678100	0.00000100
C	-11.42742000	-1.36305000	-0.00000500
C	-10.84160700	-0.10044800	-0.00000700
C	-9.45280800	0.25349000	-0.00001200
C	-8.91115800	1.53031800	-0.00001300

C	-7.49691100	1.54048200	-0.00001700
C	-6.91544100	0.27563900	-0.00002000
C	-5.52848800	-0.08304100	-0.00002300
C	-4.99070300	-1.36183900	-0.00002500
C	-3.57659000	-1.37655700	-0.00002800
C	-2.99050800	-0.11387200	-0.00002700
C	-1.60269000	0.24057100	-0.00002800
C	-1.06123300	1.51789300	-0.00002700
C	0.35288600	1.52874900	-0.00002700
C	0.93587500	0.26472800	-0.00002900
C	2.32279300	-0.09327400	-0.00002800
C	2.86141100	-1.37173600	-0.00003000
C	4.27566200	-1.38586800	-0.00002800
C	4.86168800	-0.12346900	-0.00002500
C	6.24956500	0.23184300	-0.00002100
C	6.79070100	1.50899000	-0.00001600
C	8.20524900	1.52038900	-0.00001200
C	8.78884800	0.25726700	-0.00001300
C	10.17655800	-0.10144700	-0.00000900
C	10.71500100	-1.37915600	-0.00001000
C	12.13025700	-1.39335000	-0.00000500
C	12.71581800	-0.13199800	0.00000100
C	14.10587100	0.22437700	0.00000900
C	14.64651000	1.49989900	0.00001500
C	16.06371600	1.50944700	0.00002300
C	16.64353600	0.24843900	0.00002300
C	18.03760300	-0.11710300	0.00003100
C	18.57494500	-1.38711800	0.00003200
C	19.99969600	-1.39967900	0.00004100
C	20.55719600	-0.14574600	0.00004700
S	-19.95055900	1.14605900	0.00004100
S	-16.02490100	-0.93415900	0.00001800
S	-12.09471800	1.13435500	0.00000000
S	-8.17408300	-0.95456400	-0.00001700
S	-4.24529400	1.12076600	-0.00002400
S	-0.32243500	-0.96668000	-0.00002900
S	3.60618600	1.11086200	-0.00002500
S	7.53095500	-0.97450600	-0.00002000
S	11.46076500	1.10195300	-0.00000100
S	15.38701000	-0.98102200	0.00001300
S	19.32625200	1.08770000	0.00004200
H	-22.23977300	0.26055100	0.00005400
H	-21.33176200	-2.20313400	0.00004800
H	-17.37036500	2.44219000	0.00002700
H	-13.45698400	-2.23713200	0.00000400
H	-9.52258200	2.42295700	-0.00001000
H	-5.60524100	-2.25232400	-0.00002500
H	-1.67343000	2.40997600	-0.00002600

H	2.24715100	-2.26241800	-0.00003200
H	6.17819600	2.40090100	-0.00001600
H	10.10104900	-2.27012700	-0.00001500
H	14.03510500	2.39266500	0.00001400
H	17.98747500	-2.29719000	0.00002600
H	21.59857900	0.13589600	0.00005400
O	20.64423300	-2.60209600	0.00004200
O	16.86075100	2.61442200	0.00003100
O	12.92543500	-2.49739000	-0.00000500
O	9.00254500	2.62281500	-0.00000700
O	5.07110200	-2.48904600	-0.00002800
O	1.15091000	2.62973100	-0.00002600
O	-2.78136800	-2.47925800	-0.00002900
O	-6.69808300	2.64025500	-0.00001900
O	-10.63304800	-2.46581300	-0.00000900
O	-14.54491100	2.65768000	0.00001100
O	-18.48772400	-2.44783500	0.00003100
C	22.06032700	-2.54905100	0.00005200
H	22.44173500	-2.03595400	0.89314800
H	22.40966400	-3.58283000	0.00005200
H	22.44174700	-2.03595000	-0.89303600
C	16.22797000	3.88506900	0.00003400
H	15.60780700	4.02457900	-0.89431800
H	15.60779800	4.02457000	0.89438000
H	17.03135100	4.62285800	0.00004100
C	12.29321300	-3.76944800	-0.00000500
H	11.67362400	-3.90803900	0.89462400
H	11.67362900	-3.90804100	-0.89463900
H	13.09741100	-4.50603900	-0.00000300
C	8.37171900	3.89588400	-0.00000800
H	7.75243900	4.03501100	-0.89466100
H	7.75243300	4.03501000	0.89464100
H	9.17665400	4.63169700	-0.00000400
C	4.43870300	-3.76174600	-0.00002900
H	3.81944000	-3.89982400	0.89470200
H	3.81944100	-3.89982400	-0.89476100
H	5.24286400	-4.49832400	-0.00002800
C	0.52177100	3.90429100	-0.00002600
H	-0.09701100	4.04382900	-0.89480700
H	-0.09701100	4.04382900	0.89475600
H	1.32790800	4.63866400	-0.00002500
C	-3.41341800	-3.75258600	-0.00003500
H	-4.03239100	-3.89061200	0.89480800
H	-4.03239100	-3.89060400	-0.89487800
H	-2.60889800	-4.48867700	-0.00003800
C	-7.32549900	3.91611300	-0.00001300
H	-7.94381300	4.05633800	-0.89493000
H	-7.94380600	4.05633300	0.89490900

H	-6.51819900	4.64909500	-0.00001400
C	-11.26517500	-3.73949500	-0.00000600
H	-11.88382900	-3.87745800	0.89501500
H	-11.88383700	-3.87745800	-0.89502200
H	-10.46054100	-4.47534100	-0.00001000
C	-15.17070500	3.93511000	0.00001500
H	-15.78826500	4.07631800	-0.89516600
H	-15.78825500	4.07631800	0.89520200
H	-14.36212100	4.66646700	0.00001000
C	-19.12344000	-3.72070200	0.00003300
H	-19.74163700	-3.85774100	0.89539500
H	-19.74164700	-3.85773900	-0.89532200
H	-18.32029400	-4.45791000	0.00002800
Ph			
C	15.90641700	8.55832200	-1.34700800
C	16.22097800	8.11135500	-0.09681300
C	15.83477400	6.75166200	0.14865300
C	15.20554800	6.18458800	-0.95062800
C	14.59194700	4.87698600	-1.13816000
C	14.62153200	4.08917600	-2.26618000
C	13.88285200	2.87089700	-2.16728600
C	13.25912400	2.74095600	-0.93278000
C	12.37889300	1.71101800	-0.40396000
C	12.31554300	1.24671000	0.89127700
C	11.31080900	0.26091400	1.12728600
C	10.57543200	-0.01627400	-0.01875800
C	9.44353800	-0.90016700	-0.24514300
C	9.16801400	-1.63534200	-1.37734200
C	7.95211800	-2.38030900	-1.32829900
C	7.27283300	-2.18939600	-0.13112100
C	5.99570600	-2.69625000	0.34322900
C	5.65501400	-3.02221000	1.63805100
C	4.30471200	-3.44785600	1.81458300
C	3.59308300	-3.42616200	0.62149300
C	2.20021000	-3.72466100	0.32978800
C	1.69086100	-4.34937100	-0.78710500
C	0.26839900	-4.46223000	-0.81507500
C	-0.32117300	-3.88531400	0.30355000
C	-1.71585600	-3.71567300	0.67659200
C	-2.25196300	-3.74033600	1.94564500
C	-3.65508300	-3.48829400	2.01004300
C	-4.19730100	-3.24604200	0.75380800
C	-5.54899900	-2.90722100	0.33854300
C	-6.20493600	-3.32286600	-0.79924700
C	-7.51685100	-2.78693400	-0.96485900
C	-7.85961000	-1.92009800	0.06568300
C	-9.04893300	-1.11503000	0.28865600
C	-9.61818100	-0.77814800	1.49754600

C	-10.75868700	0.07463400	1.40733600
C	-11.05344600	0.41033700	0.09188300
C	-12.07750500	1.27462900	-0.47410600
C	-12.77896700	1.08700500	-1.64428000
C	-13.69248900	2.13517900	-1.97034600
C	-13.66624500	3.15719400	-1.03092100
C	-14.37714000	4.42807400	-0.97645300
C	-14.89726800	5.05161800	0.13435500
C	-15.44108600	6.35287800	-0.11790600
C	-15.31412300	6.69684000	-1.44478800
S	15.09866600	7.33844600	-2.27045000
S	13.58332500	4.13317800	0.08302300
S	11.13012400	0.95501000	-1.36986500
S	8.14282000	-1.07972500	0.91256000
S	4.60086600	-2.86876400	-0.70099700
S	0.89280800	-3.20300200	1.37002200
S	-2.96156000	-3.32095300	-0.48840400
S	-6.54777500	-1.77230600	1.22133200
S	-9.90846200	-0.32342900	-1.01500000
S	-12.50704300	2.81443600	0.23841200
S	-14.54142100	5.46778500	-2.38258600
H	16.11545500	9.52449500	-1.78462200
H	16.75228600	8.70866600	0.63507000
H	15.21074200	4.34530100	-3.13943200
H	13.01647000	1.56049800	1.65644000
H	9.85346600	-1.69228700	-2.21529700
H	6.37543800	-3.01536400	2.44813000
H	2.32622600	-4.77763800	-1.55401800
H	-1.66398800	-3.99392700	2.82044800
H	-5.78082000	-4.04935200	-1.48306600
H	-9.26387400	-1.18055400	2.43982100
H	-12.68908700	0.18361300	-2.23672800
H	-14.87709400	4.59246700	1.11609900
H	-15.68143400	7.58939300	-1.93192300
C	16.14772200	6.06964000	1.42772300
C	16.74506500	4.79835100	1.45331300
C	15.89725300	6.71577300	2.64963800
C	17.06958000	4.18922100	2.66374500
H	16.96479800	4.29520500	0.51719600
C	16.22292800	6.10550500	3.86021900
H	15.42946700	7.69609100	2.64687700
C	16.80934400	4.83921600	3.87187100
H	17.53448600	3.20754900	2.66292000
H	16.01509100	6.61895600	4.79459000
H	17.06448200	4.36358600	4.81427100
C	13.87876200	1.87125900	-3.26328100
C	14.16407400	0.51732500	-3.02082000
C	13.63924900	2.27884800	-4.58594700

C	14.19749100	-0.40089800	-4.06834300
H	14.37123500	0.19101000	-2.00670300
C	13.67315900	1.35908200	-5.63333900
H	13.41092300	3.32136200	-4.78806800
C	13.95127000	0.01555200	-5.37823400
H	14.42428100	-1.44289400	-3.86187400
H	13.47898100	1.69263000	-6.64865700
H	13.97905200	-0.70119300	-6.19368000
C	11.16846400	-0.40312800	2.44622700
C	11.11500300	-1.80224200	2.55947500
C	11.14107100	0.36583400	3.62131200
C	11.02496100	-2.41132600	3.80944500
H	11.15649300	-2.41016200	1.66140200
C	11.05122000	-0.24492300	4.87156500
H	11.17617300	1.44898800	3.54872200
C	10.99126300	-1.63569100	4.97008200
H	10.98910300	-3.49479500	3.87749800
H	11.02492200	0.36711000	5.76853500
H	10.92210500	-2.11211800	5.94359600
C	7.55235700	-3.28913100	-2.43078200
C	7.20448600	-4.62769800	-2.18562800
C	7.57126500	-2.83376600	-3.75927500
C	6.87381500	-5.48017900	-3.23712000
H	7.20664000	-4.99886800	-1.16574100
C	7.24076100	-3.68799200	-4.81063000
H	7.83351100	-1.79964700	-3.96307100
C	6.88940000	-5.01380300	-4.55322800
H	6.61125800	-6.51335200	-3.02868800
H	7.25505200	-3.31576600	-5.83095400
H	6.63282200	-5.67985900	-5.37178800
C	3.80025400	-3.91738600	3.12810900
C	3.11851500	-5.13910700	3.25479000
C	4.04734100	-3.16424000	4.28760100
C	2.68727900	-5.58644600	4.50194200
H	2.93845500	-5.74146600	2.37014200
C	3.61559300	-3.61329800	5.53506700
H	4.56848300	-2.21496100	4.20404800
C	2.93286500	-4.82537800	5.64661200
H	2.16482400	-6.53546200	4.58096200
H	3.81033300	-3.01362300	6.41954100
H	2.59696600	-5.17597300	6.61808500
C	-0.43309400	-5.18599100	-1.90363700
C	-1.38277100	-6.18308500	-1.62572400
C	-0.11374400	-4.92021600	-3.24539100
C	-2.00182400	-6.88337600	-2.65907400
H	-1.62521100	-6.41309800	-0.59318700
C	-0.73368400	-5.62204600	-4.27857400
H	0.61465300	-4.14814600	-3.47578900

C	-1.68102000	-6.60510500	-3.98930900
H	-2.73080000	-7.65367900	-2.42442200
H	-0.47808100	-5.39766500	-5.31015800
H	-2.16353000	-7.15256000	-4.79365500
C	-4.40149500	-3.56322100	3.28963600
C	-5.58089600	-4.31734400	3.40631800
C	-3.90304600	-2.91795500	4.43341900
C	-6.24557700	-4.41326000	4.62721400
H	-5.96715500	-4.83862800	2.53633500
C	-4.56895000	-3.01513100	5.65460000
H	-2.99557400	-2.32600700	4.35709400
C	-5.74330400	-3.76220100	5.75577900
H	-7.15389900	-5.00455900	4.69903700
H	-4.17090300	-2.50382900	6.52635900
H	-6.26190200	-3.83912700	6.70689900
C	-8.38978600	-3.20287400	-2.09040200
C	-9.69225600	-3.67894300	-1.86714400
C	-7.90048500	-3.18066800	-3.40664400
C	-10.48474100	-4.10700900	-2.93033800
H	-10.07543400	-3.72234800	-0.85257400
C	-8.69443800	-3.60963800	-4.46974300
H	-6.89648600	-2.81117600	-3.59435800
C	-9.98985300	-4.07286600	-4.23562700
H	-11.48830500	-4.47534400	-2.73793500
H	-8.30080600	-3.57875000	-5.48164100
H	-10.60828200	-4.40776300	-5.06321100
C	-11.53827700	0.46301900	2.60818400
C	-12.93421200	0.31341100	2.65380300
C	-10.87775100	0.94047800	3.75205800
C	-13.64717600	0.64230900	3.80492500
H	-13.45572600	-0.07405400	1.78445400
C	-11.59242000	1.26926500	4.90327700
H	-9.79926900	1.06713900	3.72938400
C	-12.97988100	1.12246400	4.93363600
H	-14.72582000	0.51527300	3.82307400
H	-11.06438300	1.64349900	5.77558300
H	-13.53681000	1.37747500	5.83047400
C	-14.58678900	2.05109500	-3.15050200
C	-15.96167900	2.32090300	-3.04799300
C	-14.07562300	1.64251900	-4.39349300
C	-16.79559600	2.19533100	-4.15710800
H	-16.37489300	2.61953500	-2.09005800
C	-14.91139600	1.51655700	-5.50234500
H	-13.01343100	1.43758000	-4.49061300
C	-16.27447200	1.79334300	-5.38857400
H	-17.85666100	2.40505200	-4.05728900
H	-14.49580500	1.20530400	-6.45643000
H	-16.92612600	1.69479300	-6.25178800

C	-16.07280000	7.20596500	0.91081900
C	-16.00047300	8.60716800	0.83263700
C	-16.76587400	6.63618400	1.99208400
C	-16.60874100	9.40996700	1.79510200
H	-15.44134000	9.06678200	0.02304900
C	-17.36882700	7.43965100	2.95831800
H	-16.85056200	5.55601600	2.06359900
C	-17.29538000	8.83011300	2.86359400
H	-16.53618200	10.49097500	1.71679600
H	-17.90312500	6.97758900	3.78356300
H	-17.76527200	9.45578800	3.61658200
SH			
C	-9.78135900	9.67837900	0.47794600
C	-10.65464600	9.51128800	-0.55788500
C	-11.14314100	8.16909700	-0.71861400
C	-10.59579600	7.32441600	0.23659900
C	-10.74772900	5.89003900	0.51632800
C	-11.32708300	5.31643300	1.62146300
C	-11.27258200	3.88548700	1.68223600
C	-10.59866000	3.36882400	0.58378600
C	-10.26608300	2.00467400	0.15395900
C	-10.69071600	1.37060100	-0.98884500
C	-10.14994700	0.06274500	-1.21354300
C	-9.26210000	-0.28582200	-0.20528000
C	-8.46093300	-1.48701100	0.06453200
C	-8.59900400	-2.34736400	1.12661500
C	-7.63038000	-3.40029800	1.20560900
C	-6.71140600	-3.30574400	0.16922900
C	-5.53901600	-4.10418200	-0.21222400
C	-5.37783000	-4.85324400	-1.35260100
C	-4.09625600	-5.47456300	-1.51318800
C	-3.25419900	-5.15230100	-0.45778400
C	-1.86957200	-5.51078800	-0.12418500
C	-1.44177100	-6.23738500	0.96053600
C	-0.02168200	-6.36710500	1.10205900
C	0.64078300	-5.68449600	0.09130300
C	2.06063600	-5.49043100	-0.23070800
C	2.72926400	-5.94185100	-1.34254900
C	4.09986600	-5.53737900	-1.45157600
C	4.46287300	-4.72616100	-0.38533000
C	5.71302500	-4.05301200	-0.00914300
C	6.48833500	-4.30151700	1.09747900
C	7.61550500	-3.43475900	1.27525200
C	7.66935000	-2.47972700	0.26944700
C	8.59089700	-1.37377300	-0.02113300
C	9.41006800	-1.24090800	-1.11606600
C	10.15037300	-0.01622100	-1.19506900
C	9.84935600	0.81408000	-0.12373900

C	10.31130800	2.14685300	0.28455100
C	10.98427800	2.46900100	1.43868200
C	11.24095300	3.86422800	1.64080400
C	10.71614300	4.62010100	0.60211700
C	10.70803500	6.06030300	0.30911500
C	11.36431900	6.70604200	-0.70915100
C	11.08246800	8.10944600	-0.81461600
C	10.18230400	8.50110900	0.14300800
S	-9.50531300	8.19349300	1.30783400
S	-10.05067600	4.64623300	-0.48852100
S	-9.10931100	0.99643900	0.98368900
S	-7.05782800	-1.93360000	-0.87017400
S	-4.05091700	-4.09842100	0.69759800
S	-0.48964800	-4.90413500	-1.00168600
S	3.11935400	-4.47931200	0.71722700
S	6.34117400	-2.66739100	-0.86279700
S	8.67049000	0.07513200	0.94638500
S	9.91874500	3.60524900	-0.58779900
S	9.66966700	7.19370700	1.15897700
H	-9.29699700	10.58994600	0.79974100
H	-10.95783700	10.33527300	-1.18999400
H	-11.81322900	5.92785100	2.37001800
H	-11.41250200	1.83824700	-1.64518900
H	-9.41388500	-2.23071300	1.82865700
H	-6.19259900	-4.97564900	-2.05379700
H	-2.15343100	-6.69580000	1.63425900
H	2.23796000	-6.58537900	-2.06018900
H	6.25786300	-5.12360500	1.76191900
H	9.49517100	-2.03807300	-1.84272200
H	11.31353100	1.69695200	2.12125700
H	12.04764500	6.17751800	-1.36417500
H	9.78578400	9.49017600	0.32072000
C	11.72240700	9.01767000	-1.86357700
C	13.25578400	9.02798200	-1.66465800
C	11.39369900	8.47952400	-3.27542100
C	11.19801200	10.46031100	-1.74960100
H	13.51961600	9.41651400	-0.67584700
H	13.68091500	8.02352800	-1.75429000
H	13.73452100	9.66221900	-2.41923300
H	10.31289900	8.46397700	-3.44778700
H	11.85265000	9.11420600	-4.04173600
H	11.76958700	7.46166200	-3.41801300
H	11.66480400	11.09091900	-2.51299700
H	10.11401600	10.50601300	-1.89740300
H	11.42998200	10.89619900	-0.77225100
C	12.01063300	4.39008500	2.86681000
C	13.06736600	5.43941600	2.45600800
C	11.01915300	5.01335800	3.87790000

C	12.75398800	3.23953600	3.58042400
H	13.78976400	5.01086500	1.75326700
H	12.62049500	6.31595400	1.98488600
H	13.61782200	5.77690400	3.34124600
H	10.25478300	4.28789400	4.17499300
H	11.55210400	5.33308000	4.78085100
H	10.51378000	5.88689900	3.46051000
H	13.34271300	3.64648600	4.40858500
H	12.06455300	2.50307700	4.00464400
H	13.44079800	2.71952600	2.90475900
C	11.14513500	0.26857200	-2.33508700
C	10.49978800	1.22457700	-3.36682500
C	12.45644300	0.88295600	-1.79656300
C	11.51897700	-1.03672800	-3.07260000
H	9.56474100	0.80749300	-3.75477100
H	10.27954400	2.20162200	-2.93134400
H	11.17836500	1.37995200	-4.21362000
H	12.94019100	0.20546500	-1.08517500
H	13.15169200	1.05380400	-2.62583100
H	12.29520600	1.83743300	-1.29386000
H	12.28186200	-0.82037300	-3.82716100
H	11.92755300	-1.78708300	-2.38808100
H	10.66367100	-1.47625100	-3.59497400
C	8.60437300	-3.60509900	2.44368300
C	10.06687000	-3.45883300	1.96840100
C	8.30070900	-2.55863200	3.54259100
C	8.46375000	-5.00764500	3.07679700
H	10.30306400	-4.20247800	1.20010100
H	10.27403500	-2.47134200	1.55392000
H	10.74670100	-3.61797700	2.81268300
H	7.26540200	-2.64408200	3.88849100
H	8.95951000	-2.71437100	4.40481200
H	8.45441800	-1.53848900	3.18385400
H	9.22364400	-5.13236300	3.85477700
H	7.48853300	-5.15026200	3.55233600
H	8.60640700	-5.80304000	2.33814400
C	4.99870900	-5.98988300	-2.61709800
C	5.13564100	-4.84304600	-3.64719400
C	6.39726100	-6.41404800	-2.11698400
C	4.37983300	-7.20577300	-3.34205900
H	4.15318300	-4.52143600	-4.00784500
H	5.64058400	-3.97409000	-3.21931200
H	5.71997700	-5.17952400	-4.51158200
H	6.32183000	-7.23485000	-1.39609100
H	7.00151400	-6.76176400	-2.96208000
H	6.93369500	-5.59453300	-1.63696600
H	5.06714000	-7.55418900	-4.11954800
H	4.20064100	-8.03907100	-2.65489500

H	3.43475800	-6.95659300	-3.83425500
C	0.61816300	-7.18040100	2.24248000
C	1.79039800	-8.04584800	1.72962400
C	1.11931100	-6.22210300	3.34931800
C	-0.41686400	-8.13631900	2.87704600
H	1.45379400	-8.73841100	0.95109300
H	2.60182200	-7.44548400	1.31621100
H	2.19900700	-8.63889200	2.55512300
H	0.30332300	-5.59511200	3.72304000
H	1.51924600	-6.79631300	4.19314200
H	1.91207400	-5.56432300	2.98592500
H	0.07577000	-8.75258800	3.63579700
H	-1.22754100	-7.59638300	3.37566200
H	-0.85774700	-8.80795300	2.13323400
C	-3.76880700	-6.38267100	-2.71248400
C	-2.90854200	-5.60670400	-3.73854500
C	-3.02397300	-7.65959100	-2.26305500
C	-5.06333800	-6.83213300	-3.42608600
H	-3.41751500	-4.69384000	-4.06459800
H	-1.93917700	-5.32367300	-3.32229100
H	-2.72545600	-6.22667400	-4.62387300
H	-3.62480900	-8.22995400	-1.54698600
H	-2.83457600	-8.30070400	-3.13099100
H	-2.06373300	-7.43925100	-1.79461300
H	-4.81089500	-7.53250400	-4.22845100
H	-5.74893500	-7.34087200	-2.74066000
H	-5.59558900	-5.99316800	-3.88503500
C	-7.67070200	-4.47030700	2.31230300
C	-7.41400300	-5.88164700	1.73961400
C	-6.61519500	-4.14317900	3.39576200
C	-9.05610000	-4.49541700	2.99604200
H	-8.16322000	-6.13751000	0.98312200
H	-6.42893700	-5.97160500	1.28004900
H	-7.47855300	-6.62341700	2.54325400
H	-6.77712400	-3.14318200	3.81112800
H	-6.68149100	-4.86579900	4.21737400
H	-5.59975900	-4.18316700	2.99569100
H	-9.08405200	-5.30707700	3.73002000
H	-9.26824700	-3.56619100	3.53368500
H	-9.86188800	-4.66725700	2.27507000
C	-10.55561900	-0.79683300	-2.42478900
C	-9.43644500	-0.75675500	-3.49270500
C	-10.83118200	-2.25875500	-2.00831800
C	-11.84717100	-0.25083400	-3.07350100
H	-9.21904700	0.27331300	-3.79340300
H	-8.51030200	-1.20414500	-3.12510200
H	-9.74636300	-1.31187900	-4.38576800
H	-11.63494900	-2.30728500	-1.26626300

H	-11.14359000	-2.83925500	-2.88343900
H	-9.95163200	-2.74300900	-1.58182500
H	-12.15457600	-0.91583100	-3.88687500
H	-12.67110500	-0.19775200	-2.35452600
H	-11.70332700	0.74409000	-3.50595000
C	-11.91234900	3.08971700	2.83497200
C	-12.68034900	1.85654800	2.30990200
C	-10.81755900	2.64368000	3.83347400
C	-12.92429800	3.96636800	3.60675900
H	-13.47139700	2.15707200	1.61494000
H	-12.03113100	1.14932200	1.79214500
H	-13.14934900	1.32946100	3.14819800
H	-10.26079600	3.50618600	4.21397400
H	-11.27174900	2.12979000	4.68876200
H	-10.10389200	1.95774300	3.37196000
H	-13.41856100	3.36064600	4.37289700
H	-12.43989200	4.80318800	4.11916800
H	-13.69892300	4.36959400	2.94641300
C	-12.14542600	7.78642600	-1.82381700
C	-11.40061700	7.08647400	-2.98563600
C	-13.25564800	6.85968100	-1.28161600
C	-12.83374600	9.04655200	-2.39383400
H	-10.60152300	7.72379800	-3.37823500
H	-10.95431300	6.14178400	-2.66687700
H	-12.09566700	6.87093900	-3.80560600
H	-13.80504400	7.34384500	-0.46750500
H	-13.96890800	6.62989300	-2.08104200
H	-12.86079400	5.91563900	-0.90411700
H	-13.59245300	8.74795000	-3.12431400
H	-13.33187900	9.62569200	-1.60961500
H	-12.12783600	9.70381400	-2.91097000
SiH_3			
C	19.39878400	4.99445500	-0.95907300
C	19.26545900	4.66101300	0.35888400
C	18.18803000	3.75082800	0.63174100
C	17.51022000	3.40736200	-0.53079400
C	16.37987500	2.51878300	-0.74567400
C	16.12894100	1.71358200	-1.83636400
C	14.92086800	0.95440200	-1.76887400
C	14.23409500	1.19589000	-0.58396400
C	12.99671300	0.63861000	-0.07100700
C	12.65470900	0.39797600	1.24400200
C	11.35295500	-0.15265600	1.44422700
C	10.68530000	-0.33277500	0.23693000
C	9.37398500	-0.88010400	-0.05264000
C	8.97384200	-1.57827000	-1.17406800
C	7.60646500	-1.98705700	-1.18188800
C	6.94761800	-1.58216800	-0.02503100

C	5.58270100	-1.79359600	0.41661800
C	5.12165200	-1.93375000	1.71001400
C	3.71318700	-2.12478900	1.83780600
C	3.08507200	-2.12723900	0.59598400
C	1.69424700	-2.31683300	0.23294400
C	1.18814900	-2.88422200	-0.91916300
C	-0.23568900	-2.92994000	-1.00183800
C	-0.82927100	-2.37544700	0.12813000
C	-2.22372900	-2.23248600	0.49838000
C	-2.76923800	-2.24053900	1.76639000
C	-4.18470600	-2.06845900	1.82227800
C	-4.73042900	-1.92356800	0.55043100
C	-6.10419200	-1.75644200	0.11775000
C	-6.68329000	-2.19299400	-1.05668100
C	-8.06515500	-1.87147500	-1.21116300
C	-8.54877400	-1.16440900	-0.11461400
C	-9.87473500	-0.65838200	0.18325200
C	-10.46868400	-0.51386200	1.42072300
C	-11.78917800	0.02744300	1.40337700
C	-12.20874800	0.30347600	0.10604800
C	-13.46511000	0.82551300	-0.39786200
C	-14.08217200	0.53708500	-1.59783700
C	-15.31763300	1.21617000	-1.82516000
C	-15.64346400	2.04869500	-0.76135100
C	-16.79952000	2.90245000	-0.53936300
C	-17.43410900	3.17635300	0.65272100
C	-18.53420500	4.08769500	0.54539300
C	-18.71211700	4.49583400	-0.75642100
S	18.19523400	4.22026600	-1.92993000
S	15.06786200	2.38365100	0.40335000
S	11.65813700	0.21821800	-1.11655000
S	8.01453000	-0.66978800	1.02884800
S	4.23678900	-1.85165100	-0.70008900
S	0.37869900	-1.77057700	1.24931100
S	-3.48374200	-1.95996800	-0.68502500
S	-7.28149100	-0.87354700	1.06459300
S	-10.95345800	-0.06969500	-1.06246600
S	-14.40583800	2.01038900	0.48105300
S	-17.55453700	3.80455800	-1.84265200
H	20.13033100	5.64656900	-1.41578800
H	19.92901200	5.04004300	1.12823800
H	16.82919200	1.63600700	-2.66092300
H	13.35207400	0.57444600	2.05564800
H	9.67045900	-1.83382500	-1.96505400
H	5.79761500	-1.94478900	2.55805300
H	1.83638600	-3.30555300	-1.67975100
H	-2.16142600	-2.41398900	2.64773900
H	-6.12962300	-2.77962500	-1.78170200

H	-9.97519300	-0.83655400	2.33104100
H	-13.67298600	-0.19117400	-2.28964900
H	-17.12940700	2.70781100	1.58259500
H	-19.46355700	5.16909700	-1.14714500
Si	17.90472400	3.09858600	2.37176700
H	16.69987100	3.68545900	3.01528600
H	17.77137600	1.62000200	2.38847300
H	19.09205900	3.48980700	3.17670900
Si	14.49693100	-0.28040300	-3.12404700
H	14.16300400	-1.61324900	-2.56232500
H	13.36310700	0.17207800	-3.97170000
H	15.70165200	-0.40002200	-3.98606200
Si	10.78759200	-0.64829100	3.16978700
H	9.73648200	0.25579300	3.70458100
H	10.27111900	-2.03964800	3.19405300
H	11.97836700	-0.55108400	4.05349200
Si	6.94015900	-3.04950500	-2.58563100
H	6.24389800	-4.25523700	-2.07149100
H	6.00572900	-2.30285500	-3.46721900
H	8.11237100	-3.46806200	-3.39739200
Si	2.95000700	-2.45054100	3.52753200
H	2.14277500	-1.30245400	4.01546600
H	2.09104100	-3.66077900	3.51240900
H	4.07852600	-2.66100100	4.47118300
Si	-1.07262400	-3.78067000	-2.45763400
H	-2.06985600	-4.78198800	-2.00388600
H	-1.74780300	-2.81754300	-3.36552200
H	0.00014500	-4.46826400	-3.22215800
Si	-5.08888700	-2.17491600	3.46999900
H	-5.60116000	-0.85531600	3.92153100
H	-6.22496600	-3.12788900	3.40663900
H	-4.09900600	-2.65644800	4.46823500
Si	-9.02538400	-2.49990000	-2.70345200
H	-10.27128800	-3.19520200	-2.29458900
H	-9.37933100	-1.41037100	-3.64970200
H	-8.13631200	-3.45933800	-3.40871500
Si	-12.77724200	0.17685400	2.99851500
H	-12.93952600	1.58816100	3.43408900
H	-14.12275100	-0.43493300	2.86404900
H	-12.01025500	-0.54820500	4.04462200
Si	-16.34619500	0.84639800	-3.35814100
H	-17.76021600	0.56597800	-3.00653000
H	-16.31238700	1.95780100	-4.34370100
H	-15.74822900	-0.35678400	-3.99339700
Si	-19.62613100	4.66388000	1.95528000
H	-20.30201100	3.51449900	2.61342100
H	-18.83316900	5.38078900	2.98887700
H	-20.65473400	5.58428400	1.40610300

SMe			
C	-21.19523400	0.23882500	-2.43238500
C	-20.81217400	1.46233800	-1.97014500
C	-19.44516300	1.50663600	-1.54817500
C	-18.78655200	0.29251700	-1.69883800
C	-17.42921100	-0.11883300	-1.39684000
C	-16.94690800	-1.41011300	-1.38371700
C	-15.56296000	-1.52476600	-1.08620200
C	-14.95111800	-0.29615900	-0.86375400
C	-13.58998300	0.05483400	-0.52259600
C	-13.13000100	1.30349100	-0.15505300
C	-11.73679300	1.37086600	0.10500700
C	-11.09149600	0.15013900	-0.06387200
C	-9.70972200	-0.24047500	0.10464600
C	-9.21487200	-1.52946500	0.10805500
C	-7.80820800	-1.62560200	0.26392400
C	-7.18696800	-0.38621500	0.38237300
C	-5.80090600	-0.01835700	0.56494600
C	-5.31505000	1.24128400	0.85555600
C	-3.90220300	1.32550200	0.95180600
C	-3.26791500	0.10695400	0.73144700
C	-1.87185400	-0.26842200	0.74571700
C	-1.36574500	-1.55273100	0.74819800
C	0.05046500	-1.63340700	0.72489300
C	0.66784700	-0.38649700	0.69754800
C	2.06127400	-0.00275000	0.67769500
C	2.56320100	1.27974400	0.77963500
C	3.97723700	1.37179800	0.71766200
C	4.59759700	0.13615400	0.56074600
C	5.99100600	-0.23648100	0.46621100
C	6.50408300	-1.51776000	0.50446900
C	7.91365400	-1.59828400	0.36640900
C	8.51855000	-0.35446500	0.21332500
C	9.90344500	0.03038900	0.05935900
C	10.40772900	1.31541500	0.08779300
C	11.80942100	1.40877900	-0.11059300
C	12.41656900	0.17136600	-0.29901100
C	13.79764800	-0.20166600	-0.51226900
C	14.32097300	-1.47836500	-0.46640400
C	15.71145700	-1.56054300	-0.74104100
C	16.28634600	-0.32360400	-1.00642300
C	17.65148900	0.06255500	-1.30979000
C	18.17421000	1.33696100	-1.23389300
C	19.53691300	1.43243800	-1.64765400
C	20.04516300	0.21911000	-2.03615700
S	-19.88898700	-0.89943500	-2.37890900
S	-16.12639700	1.00586100	-1.03435000
S	-12.24721500	-1.08288700	-0.56211600

S	-8.38441200	0.90321100	0.29207700
S	-4.45935000	-1.14528300	0.39268600
S	-0.54702300	0.89037200	0.70409000
S	3.38876300	-1.14329100	0.48185700
S	7.30050400	0.91858800	0.23845900
S	11.21087200	-1.11130400	-0.23792100
S	15.06935800	0.94362800	-0.92248700
S	18.87293800	-1.05684900	-1.90257300
H	-22.15985200	-0.06728900	-2.81222800
H	-21.46371800	2.32653700	-1.92530600
H	-17.56386800	-2.27924800	-1.57940200
H	-13.77103800	2.17250600	-0.06554300
H	-9.84041400	-2.40812900	0.00469000
H	-5.95145600	2.10540500	1.00516500
H	-1.98928100	-2.43862400	0.76954100
H	1.93869200	2.15728400	0.89821800
H	5.89090300	-2.40183800	0.63303900
H	9.79404400	2.19337500	0.25134700
H	13.73138600	-2.35640600	-0.23076700
H	17.60368000	2.18809200	-0.87976400
H	21.04102100	-0.01584300	-2.38130500
S	16.55860900	-3.13137900	-0.75406600
S	20.37039900	2.99960400	-1.60239600
S	12.63761800	2.98866800	-0.13916300
S	8.75483500	-3.17141700	0.36257300
S	4.80705400	2.94891700	0.79949500
S	0.88745800	-3.20836100	0.69780600
S	-3.08741500	2.87620200	1.29018600
S	-6.98298300	-3.20729100	0.26323700
S	-10.95238600	2.90595700	0.56418700
S	-14.74977500	-3.11192900	-1.05657800
S	-18.70505600	3.01130000	-0.94251600
C	22.02577600	2.55516700	-2.22038100
H	22.60442100	3.48056500	-2.23115500
H	22.51428700	1.83653000	-1.55801600
H	21.97198000	2.15626600	-3.23611900
C	17.36278200	-3.11865100	0.89711000
H	18.10004500	-2.31664000	0.96392000
H	16.61796000	-3.01780100	1.68848200
H	17.86915300	-4.08148500	0.99774200
C	13.21355000	3.12184900	1.60023600
H	12.36994300	3.07312900	2.29105400
H	13.93864000	2.33930500	1.83101700
H	13.69703500	4.09755800	1.68864800
C	9.23958200	-3.32126800	2.12841300
H	9.94891900	-2.53905900	2.40496600
H	8.36080800	-3.28236100	2.77454500
H	9.72107000	-4.29652000	2.23164200

C	5.20978900	3.04743800	2.58937800
H	4.30283400	2.98293100	3.19313300
H	5.91201000	2.26178300	2.87426400
H	5.67808800	4.02229400	2.74415100
C	1.17013700	-3.49165900	2.49115800
H	1.84398200	-2.73814700	2.90298800
H	0.22412500	-3.49251300	3.03556500
H	1.63605800	-4.47622700	2.57567100
C	-2.82796600	2.75570900	3.10499000
H	-3.77901400	2.61770700	3.62235400
H	-2.14220500	1.94176800	3.34748000
H	-2.38344300	3.70472800	3.41379700
C	-6.87517300	-3.55403200	2.06425500
H	-6.23860500	-2.82173200	2.56433100
H	-7.86897500	-3.56391700	2.51530400
H	-6.42592900	-4.54543600	2.15857000
C	-10.97114900	2.78036100	2.39705700
H	-11.99253800	2.67061400	2.76585200
H	-10.35368400	1.94667000	2.73645700
H	-10.55044000	3.71619100	2.77232100
C	-14.86577900	-3.54013500	0.72652100
H	-14.29771900	-2.83302500	1.33376200
H	-15.90761900	-3.56710300	1.05068400
H	-14.42933400	-4.53625100	0.83065200
C	-18.94734200	2.82608700	0.86960800
H	-20.00635800	2.71333800	1.10826200
H	-18.38018500	1.97571600	1.25256100
H	-18.57061200	3.74520800	1.32444500
*t*Bu			
C	-9.78135900	9.67837900	0.47794600
C	-10.65464600	9.51128800	-0.55788500
C	-11.14314100	8.16909700	-0.71861400
C	-10.59579600	7.32441600	0.23659900
C	-10.74772900	5.89003900	0.51632800
C	-11.32708300	5.31643300	1.62146300
C	-11.27258200	3.88548700	1.68223600
C	-10.59866000	3.36882400	0.58378600
C	-10.26608300	2.00467400	0.15395900
C	-10.69071600	1.37060100	-0.98884500
C	-10.14994700	0.06274500	-1.21354300
C	-9.26210000	-0.28582200	-0.20528000
C	-8.46093300	-1.48701100	0.06453200
C	-8.59900400	-2.34736400	1.12661500
C	-7.63038000	-3.40029800	1.20560900
C	-6.71140600	-3.30574400	0.16922900
C	-5.53901600	-4.10418200	-0.21222400
C	-5.37783000	-4.85324400	-1.35260100
C	-4.09625600	-5.47456300	-1.51318800

C	-3.25419900	-5.15230100	-0.45778400
C	-1.86957200	-5.51078800	-0.12418500
C	-1.44177100	-6.23738500	0.96053600
C	-0.02168200	-6.36710500	1.10205900
C	0.64078300	-5.68449600	0.09130300
C	2.06063600	-5.49043100	-0.23070800
C	2.72926400	-5.94185100	-1.34254900
C	4.09986600	-5.53737900	-1.45157600
C	4.46287300	-4.72616100	-0.38533000
C	5.71302500	-4.05301200	-0.00914300
C	6.48833500	-4.30151700	1.09747900
C	7.61550500	-3.43475900	1.27525200
C	7.66935000	-2.47972700	0.26944700
C	8.59089700	-1.37377300	-0.02113300
C	9.41006800	-1.24090800	-1.11606600
C	10.15037300	-0.01622100	-1.19506900
C	9.84935600	0.81408000	-0.12373900
C	10.31130800	2.14685300	0.28455100
C	10.98427800	2.46900100	1.43868200
C	11.24095300	3.86422800	1.64080400
C	10.71614300	4.62010100	0.60211700
C	10.70803500	6.06030300	0.30911500
C	11.36431900	6.70604200	-0.70915100
C	11.08246800	8.10944600	-0.81461600
C	10.18230400	8.50110900	0.14300800
S	-9.50531300	8.19349300	1.30783400
S	-10.05067600	4.64623300	-0.48852100
S	-9.10931100	0.99643900	0.98368900
S	-7.05782800	-1.93360000	-0.87017400
S	-4.05091700	-4.09842100	0.69759800
S	-0.48964800	-4.90413500	-1.00168600
S	3.11935400	-4.47931200	0.71722700
S	6.34117400	-2.66739100	-0.86279700
S	8.67049000	0.07513200	0.94638500
S	9.91874500	3.60524900	-0.58779900
S	9.66966700	7.19370700	1.15897700
H	-9.29699700	10.58994600	0.79974100
H	-10.95783700	10.33527300	-1.18999400
H	-11.81322900	5.92785100	2.37001800
H	-11.41250200	1.83824700	-1.64518900
H	-9.41388500	-2.23071300	1.82865700
H	-6.19259900	-4.97564900	-2.05379700
H	-2.15343100	-6.69580000	1.63425900
H	2.23796000	-6.58537900	-2.06018900
H	6.25786300	-5.12360500	1.76191900
H	9.49517100	-2.03807300	-1.84272200
H	11.31353100	1.69695200	2.12125700
H	12.04764500	6.17751800	-1.36417500

H	9.78578400	9.49017600	0.32072000
C	11.72240700	9.01767000	-1.86357700
C	13.25578400	9.02798200	-1.66465800
C	11.39369900	8.47952400	-3.27542100
C	11.19801200	10.46031100	-1.74960100
H	13.51961600	9.41651400	-0.67584700
H	13.68091500	8.02352800	-1.75429000
H	13.73452100	9.66221900	-2.41923300
H	10.31289900	8.46397700	-3.44778700
H	11.85265000	9.11420600	-4.04173600
H	11.76958700	7.46166200	-3.41801300
H	11.66480400	11.09091900	-2.51299700
H	10.11401600	10.50601300	-1.89740300
H	11.42998200	10.89619900	-0.77225100
C	12.01063300	4.39008500	2.86681000
C	13.06736600	5.43941600	2.45600800
C	11.01915300	5.01335800	3.87790000
C	12.75398800	3.23953600	3.58042400
H	13.78976400	5.01086500	1.75326700
H	12.62049500	6.31595400	1.98488600
H	13.61782200	5.77690400	3.34124600
H	10.25478300	4.28789400	4.17499300
H	11.55210400	5.33308000	4.78085100
H	10.51378000	5.88689900	3.46051000
H	13.34271300	3.64648600	4.40858500
H	12.06455300	2.50307700	4.00464400
H	13.44079800	2.71952600	2.90475900
C	11.14513500	0.26857200	-2.33508700
C	10.49978800	1.22457700	-3.36682500
C	12.45644300	0.88295600	-1.79656300
C	11.51897700	-1.03672800	-3.07260000
H	9.56474100	0.80749300	-3.75477100
H	10.27954400	2.20162200	-2.93134400
H	11.17836500	1.37995200	-4.21362000
H	12.94019100	0.20546500	-1.08517500
H	13.15169200	1.05380400	-2.62583100
H	12.29520600	1.83743300	-1.29386000
H	12.28186200	-0.82037300	-3.82716100
H	11.92755300	-1.78708300	-2.38808100
H	10.66367100	-1.47625100	-3.59497400
C	8.60437300	-3.60509900	2.44368300
C	10.06687000	-3.45883300	1.96840100
C	8.30070900	-2.55863200	3.54259100
C	8.46375000	-5.00764500	3.07679700
H	10.30306400	-4.20247800	1.20010100
H	10.27403500	-2.47134200	1.55392000
H	10.74670100	-3.61797700	2.81268300
H	7.26540200	-2.64408200	3.88849100

H	8.95951000	-2.71437100	4.40481200
H	8.45441800	-1.53848900	3.18385400
H	9.22364400	-5.13236300	3.85477700
H	7.48853300	-5.15026200	3.55233600
H	8.60640700	-5.80304000	2.33814400
C	4.99870900	-5.98988300	-2.61709800
C	5.13564100	-4.84304600	-3.64719400
C	6.39726100	-6.41404800	-2.11698400
C	4.37983300	-7.20577300	-3.34205900
H	4.15318300	-4.52143600	-4.00784500
H	5.64058400	-3.97409000	-3.21931200
H	5.71997700	-5.17952400	-4.51158200
H	6.32183000	-7.23485000	-1.39609100
H	7.00151400	-6.76176400	-2.96208000
H	6.93369500	-5.59453300	-1.63696600
H	5.06714000	-7.55418900	-4.11954800
H	4.20064100	-8.03907100	-2.65489500
H	3.43475800	-6.95659300	-3.83425500
C	0.61816300	-7.18040100	2.24248000
C	1.79039800	-8.04584800	1.72962400
C	1.11931100	-6.22210300	3.34931800
C	-0.41686400	-8.13631900	2.87704600
H	1.45379400	-8.73841100	0.95109300
H	2.60182200	-7.44548400	1.31621100
H	2.19900700	-8.63889200	2.55512300
H	0.30332300	-5.59511200	3.72304000
H	1.51924600	-6.79631300	4.19314200
H	1.91207400	-5.56432300	2.98592500
H	0.07577000	-8.75258800	3.63579700
H	-1.22754100	-7.59638300	3.37566200
H	-0.85774700	-8.80795300	2.13323400
C	-3.76880700	-6.38267100	-2.71248400
C	-2.90854200	-5.60670400	-3.73854500
C	-3.02397300	-7.65959100	-2.26305500
C	-5.06333800	-6.83213300	-3.42608600
H	-3.41751500	-4.69384000	-4.06459800
H	-1.93917700	-5.32367300	-3.32229100
H	-2.72545600	-6.22667400	-4.62387300
H	-3.62480900	-8.22995400	-1.54698600
H	-2.83457600	-8.30070400	-3.13099100
H	-2.06373300	-7.43925100	-1.79461300
H	-4.81089500	-7.53250400	-4.22845100
H	-5.74893500	-7.34087200	-2.74066000
H	-5.59558900	-5.99316800	-3.88503500
C	-7.67070200	-4.47030700	2.31230300
C	-7.41400300	-5.88164700	1.73961400
C	-6.61519500	-4.14317900	3.39576200
C	-9.05610000	-4.49541700	2.99604200

H	-8.16322000	-6.13751000	0.98312200
H	-6.42893700	-5.97160500	1.28004900
H	-7.47855300	-6.62341700	2.54325400
H	-6.77712400	-3.14318200	3.81112800
H	-6.68149100	-4.86579900	4.21737400
H	-5.59975900	-4.18316700	2.99569100
H	-9.08405200	-5.30707700	3.73002000
H	-9.26824700	-3.56619100	3.53368500
H	-9.86188800	-4.66725700	2.27507000
C	-10.55561900	-0.79683300	-2.42478900
C	-9.43644500	-0.75675500	-3.49270500
C	-10.83118200	-2.25875500	-2.00831800
C	-11.84717100	-0.25083400	-3.07350100
H	-9.21904700	0.27331300	-3.79340300
H	-8.51030200	-1.20414500	-3.12510200
H	-9.74636300	-1.31187900	-4.38576800
H	-11.63494900	-2.30728500	-1.26626300
H	-11.14359000	-2.83925500	-2.88343900
H	-9.95163200	-2.74300900	-1.58182500
H	-12.15457600	-0.91583100	-3.88687500
H	-12.67110500	-0.19775200	-2.35452600
H	-11.70332700	0.74409000	-3.50595000
C	-11.91234900	3.08971700	2.83497200
C	-12.68034900	1.85654800	2.30990200
C	-10.81755900	2.64368000	3.83347400
C	-12.92429800	3.96636800	3.60675900
H	-13.47139700	2.15707200	1.61494000
H	-12.03113100	1.14932200	1.79214500
H	-13.14934900	1.32946100	3.14819800
H	-10.26079600	3.50618600	4.21397400
H	-11.27174900	2.12979000	4.68876200
H	-10.10389200	1.95774300	3.37196000
H	-13.41856100	3.36064600	4.37289700
H	-12.43989200	4.80318800	4.11916800
H	-13.69892300	4.36959400	2.94641300
C	-12.14542600	7.78642600	-1.82381700
C	-11.40061700	7.08647400	-2.98563600
C	-13.25564800	6.85968100	-1.28161600
C	-12.83374600	9.04655200	-2.39383400
H	-10.60152300	7.72379800	-3.37823500
H	-10.95431300	6.14178400	-2.66687700
H	-12.09566700	6.87093900	-3.80560600
H	-13.80504400	7.34384500	-0.46750500
H	-13.96890800	6.62989300	-2.08104200
H	-12.86079400	5.91563900	-0.90411700
H	-13.59245300	8.74795000	-3.12431400
H	-13.33187900	9.62569200	-1.60961500
H	-12.12783600	9.70381400	-2.91097000

SCREWS XYZ

BF_2			
C	-21.19146100	-0.13468000	-0.36060900
C	-20.73917700	-1.41715900	-0.26798500
C	-19.32184500	-1.52574300	-0.05305100
C	-18.71411700	-0.27343700	0.00598200
C	-17.32507300	0.12922100	0.19399700
C	-16.86259600	1.20110200	0.92139100
C	-15.45285700	1.42647500	0.84190800
C	-14.82969000	0.48755300	0.01892300
C	-13.43496200	0.29167400	-0.35433000
C	-12.95751900	-0.12970500	-1.57440300
C	-11.54646400	-0.34819200	-1.63259700
C	-10.93661200	-0.08426800	-0.40500600
C	-9.54690600	-0.14734500	0.02750100
C	-9.08574400	-0.53006800	1.26672600
C	-7.67691400	-0.39181300	1.46041100
C	-7.05151700	0.11925900	0.32179700
C	-5.65672000	0.41899900	0.02708500
C	-5.18087400	1.45571300	-0.74346000
C	-3.76924700	1.45102200	-0.96390900
C	-3.15695100	0.36442700	-0.33696900
C	-1.76515400	-0.05856500	-0.25793200
C	-1.29579300	-1.35257300	-0.24527300
C	0.11242100	-1.48613100	-0.04402300
C	0.72823400	-0.24234000	0.10527700
C	2.11884300	0.13778500	0.31585700
C	2.58131000	1.17867200	1.08858900
C	3.99168600	1.39973200	1.02903000
C	4.61658700	0.49071100	0.17346700
C	6.01218800	0.30380200	-0.19972600
C	6.49205000	-0.08410400	-1.43016300
C	7.90259600	-0.30324000	-1.49099400
C	8.50981000	-0.07487400	-0.25484200
C	9.89814600	-0.15310800	0.17906400
C	10.35520800	-0.57112900	1.40836000
C	11.76382200	-0.44142300	1.60925200
C	12.39326700	0.09996600	0.48697100
C	13.78953500	0.40448100	0.20449900
C	14.27007500	1.46288500	-0.53277700
C	15.68208800	1.45791700	-0.75244200
C	16.28812800	0.35044300	-0.15877700
C	17.68085300	-0.07579200	-0.09410600
C	18.16171500	-1.36179700	-0.19114100
C	19.57555500	-1.47668500	-0.00396500

C	20.14632900	-0.24537900	0.25011100
S	-19.89226400	1.00150700	-0.21464800
S	-15.98622400	-0.65346300	-0.61843100
S	-12.10662500	0.44476400	0.77661300
S	-8.20475500	0.39390400	-0.95874700
S	-4.32550500	-0.60562900	0.52222600
S	-0.43205600	1.05383200	-0.02867800
S	3.46098600	-0.62149300	-0.51407900
S	7.33817400	0.42453600	0.93804600
S	11.24411000	0.41209200	-0.78862500
S	15.11651900	-0.64115800	0.66837500
S	18.99883800	1.03299600	0.25825200
H	-22.20452600	0.21378400	-0.50615700
H	-21.38791500	-2.28276000	-0.33142500
H	-17.52031800	1.82853200	1.51177300
H	-13.60540700	-0.28453200	-2.42948400
H	-9.74424300	-0.90976500	2.03949600
H	-5.83024200	2.22101600	-1.15284000
H	-1.94785600	-2.20818800	-0.37867300
H	1.92297000	1.78615100	1.69894100
H	5.84644500	-0.21369600	-2.29118600
H	9.69404300	-0.97068500	2.16878100
H	13.62427200	2.24313200	-0.91907800
H	17.52144900	-2.20800200	-0.41336400
H	21.19295400	-0.03070100	0.42103800
B	20.38066400	-2.78732700	-0.08116100
B	16.33088300	2.53609100	-1.64668800
B	12.37916100	-0.79266300	2.98074600
B	8.54160900	-0.82905700	-2.79421300
B	4.61322500	2.59538800	1.78222300
B	0.74252800	-2.88979900	0.08173400
B	-3.13248200	2.51134600	-1.88766100
B	-7.05796800	-0.70371700	2.83980000
B	-10.90932100	-0.90792300	-2.92226600
B	-14.82952500	2.65330300	1.54028000
B	-18.67741300	-2.90679400	0.18535300
F	19.78473600	-3.94731200	-0.33512600
F	21.69645000	-2.80532000	0.10378800
F	17.60508900	2.52255200	-2.01750600
F	15.58632300	3.53854800	-2.10598600
F	11.60713100	-1.32230200	3.92577400
F	13.65034400	-0.58234100	3.29976000
F	9.81651700	-1.18120500	-2.90609000
F	7.78786900	-0.96520900	-3.88174600
F	5.88597900	2.95757500	1.67762200
F	3.84567400	3.33661700	2.57642200
F	2.01429100	-3.10532900	0.39509500
F	-0.01473700	-3.96605700	-0.11114800

F	-1.85531800	2.50832300	-2.24946900
F	-3.88936400	3.48790200	-2.38027700
F	-5.78575400	-0.48408000	3.14906400
F	-7.82678200	-1.20668100	3.80163200
F	-9.63349400	-1.25966700	-3.02767400
F	-11.66458200	-1.07543700	-4.00442400
F	-13.55849700	3.01493700	1.41040000
F	-15.59218300	3.42571700	2.30960700
F	-17.40565600	-3.08329500	0.52540900
F	-19.41860800	-4.00599200	0.06934600
BH_2			
C	-20.92025600	-0.24148200	0.03334300
C	-20.45420500	-0.95275100	1.09567400
C	-19.02741200	-0.87506100	1.29308300
C	-18.43205100	-0.07411400	0.30650200
C	-17.04385900	0.28643000	0.07349700
C	-16.55604900	1.45425700	-0.46334100
C	-15.13796500	1.50247100	-0.66605600
C	-14.53544000	0.30211200	-0.24984400
C	-13.14586500	-0.11558300	-0.23827200
C	-12.65481800	-1.39497600	-0.36098100
C	-11.23585100	-1.53412000	-0.22637800
C	-10.63370900	-0.28406100	0.00465900
C	-9.24303100	0.08657100	0.18615500
C	-8.74605600	1.14420300	0.91303200
C	-7.32793900	1.33212700	0.85059600
C	-6.73261800	0.35317600	0.03450900
C	-5.34475800	0.11760900	-0.31508400
C	-4.85612600	-0.44861400	-1.47025600
C	-3.43865800	-0.64539900	-1.51692800
C	-2.83505300	-0.19450800	-0.32882200
C	-1.44541300	-0.16748000	0.08545800
C	-0.95344100	-0.23904800	1.36878600
C	0.46539000	-0.10142200	1.50142500
C	1.06698700	0.07809200	0.24239000
C	2.45719700	0.24335500	-0.13635100
C	2.95292900	0.92255400	-1.22595600
C	4.37073500	0.85285900	-1.41198900
C	4.96740200	0.08146900	-0.39802200
C	6.35528800	-0.25774100	-0.14891700
C	6.84356400	-1.38529900	0.47094600
C	8.26125900	-1.42473200	0.66675200
C	8.86564400	-0.26053000	0.15810000
C	10.25575100	0.14983800	0.10846800
C	10.74946100	1.43454800	0.11472800
C	12.16824700	1.55796100	-0.03164400
C	12.76767900	0.29070400	-0.14826400
C	14.15772400	-0.09725500	-0.29488500

C	14.65325300	-1.21502000	-0.92653800
C	16.07096500	-1.39972800	-0.84340900
C	16.66514900	-0.35546200	-0.11371700
C	18.05529500	-0.09621200	0.21824200
C	18.55240000	0.50588000	1.35128900
C	19.97702400	0.68022200	1.36760600
C	20.53344600	0.19477300	0.19140400
S	-19.62173300	0.54041500	-0.81574600
S	-15.71233900	-0.81807800	0.38243700
S	-11.81329200	0.99987200	0.02774000
S	-7.91675300	-0.75758000	-0.60090200
S	-4.01111500	0.46501900	0.77659400
S	-0.11314300	0.04960200	-1.04104800
S	3.78472600	-0.50357500	0.74172100
S	7.68979200	0.81371600	-0.55166000
S	11.58630800	-0.98800900	-0.05363400
S	15.48463400	0.80853600	0.41991300
S	19.37722400	-0.45834000	-0.89284100
H	-21.93784600	-0.12696600	-0.31378900
H	-21.09820000	-1.51966300	1.75840200
H	-17.20114900	2.28784200	-0.71831800
H	-13.29874300	-2.24717200	-0.54869400
H	-9.38486200	1.79655500	1.49829100
H	-5.50074100	-0.72922700	-2.29603600
H	-1.59661100	-0.39114600	2.22865600
H	2.31343600	1.47774200	-1.90345500
H	6.19856100	-2.19588900	0.79205900
H	10.10773600	2.30196800	0.22418800
H	14.01421400	-1.91411100	-1.45485600
H	17.91615200	0.80294500	2.17787000
H	21.58260500	0.18607400	-0.07473800
B	20.81122700	1.29922000	2.49259300
H	21.99700800	1.38550800	2.37587700
H	20.28755800	1.69907400	3.48909100
B	16.75902400	-2.65275600	-1.39391700
H	17.90704500	-2.89218800	-1.18890800
H	16.11151600	-3.43089000	-2.02873700
B	12.84890200	2.92452500	-0.15388800
H	14.00059100	3.04398500	-0.43038100
H	12.19079000	3.90846100	0.00839100
B	8.93445000	-2.56269900	1.43976500
H	10.08444000	-2.53693600	1.74631900
H	8.27171900	-3.50021300	1.77065100
B	5.05208800	1.46331700	-2.64027300
H	4.39679200	2.12467900	-3.38915200
H	6.20154300	1.28185600	-2.89128500
B	1.14290600	-0.05136700	2.87401100
H	0.48146400	-0.26146800	3.84661500

H	2.29522300	0.21414300	3.01110400
B	-2.76472300	-1.36398100	-2.68945600
H	-3.42605200	-1.64822700	-3.64314700
H	-1.61523600	-1.67342100	-2.67623800
B	-6.64477600	2.53158300	1.51379300
H	-7.29847800	3.25165000	2.20798300
H	-5.49515800	2.78916300	1.34228200
B	-10.55857200	-2.90740800	-0.23085900
H	-9.40636400	-3.05521300	0.02998000
H	-11.21942400	-3.87113400	-0.48064700
B	-14.46157500	2.68989700	-1.35573400
H	-13.31235100	2.68022500	-1.66739400
H	-15.11914900	3.65347800	-1.61549000
B	-18.33851500	-1.50485100	2.50528700
H	-17.18831000	-1.32111500	2.75457500
H	-18.98412600	-2.18671400	3.24523700
CBr_3			
C	21.61122200	0.25069600	-0.96477300
C	21.28003400	0.95445100	0.15440900
C	19.89334000	0.85284200	0.50520000
C	19.17977400	0.06305700	-0.39198800
C	17.77933900	-0.34711600	-0.48278700
C	17.34158000	-1.63434700	-0.68202400
C	15.93332700	-1.76132800	-0.87230600
C	15.28610500	-0.52946300	-0.81316100
C	13.88950300	-0.11792100	-0.94313500
C	13.44281900	0.91602300	-1.73111900
C	12.05512600	1.21472500	-1.59090200
C	11.43298800	0.37877800	-0.66645100
C	10.06275100	0.26830500	-0.16957400
C	9.70986900	0.17267900	1.15564600
C	8.32275800	-0.06656600	1.38627500
C	7.60419700	-0.15412500	0.19624500
C	6.19498200	-0.38564300	-0.11565000
C	5.73271200	-1.28255700	-1.04932800
C	4.32576400	-1.23220600	-1.27768100
C	3.70455800	-0.26553400	-0.49007100
C	2.31971000	0.17983000	-0.34730000
C	1.90987100	1.49209000	-0.34776100
C	0.52644900	1.68359600	-0.05771200
C	-0.13027000	0.47642800	0.16898900
C	-1.51198600	0.12504400	0.49151700
C	-1.89228500	-0.74492300	1.48549500
C	-3.28715500	-1.04069500	1.51602200
C	-3.98409000	-0.37151000	0.51231100
C	-5.38962900	-0.33330700	0.11367600
C	-5.84395800	-0.45733800	-1.17802400
C	-7.24587800	-0.25130500	-1.33891000

C	-7.87254200	0.03654800	-0.12837900
C	-9.25538600	0.32171600	0.24889700
C	-9.65113100	1.35462300	1.06521000
C	-11.03661300	1.34902500	1.40344600
C	-11.71103600	0.27801100	0.82137200
C	-13.10175900	-0.17037600	0.84322900
C	-13.50856800	-1.46597800	1.05665500
C	-14.90768400	-1.68609500	0.88560600
C	-15.57992900	-0.51768200	0.53521500
C	-16.97991900	-0.20502500	0.25229300
C	-17.43213300	0.49695500	-0.83670000
C	-18.83859100	0.75844500	-0.80553600
C	-19.43191400	0.25916000	0.32585300
S	20.23882100	-0.54226500	-1.64371900
S	16.42768200	0.75708400	-0.52851500
S	12.57211000	-0.75415900	0.01012200
S	8.65357200	0.05955100	-1.17931400
S	4.86793200	0.55977700	0.51220800
S	0.97150000	-0.86645800	0.02216700
S	-2.90410200	0.60612000	-0.44597600
S	-6.71902200	0.04978300	1.17903700
S	-10.62116700	-0.69992700	-0.12531500
S	-14.47360700	0.82401000	0.42023400
S	-18.30705800	-0.53260400	1.36135200
H	22.58567000	0.15267900	-1.42201000
H	21.99355600	1.52172700	0.73439100
H	18.01544900	-2.47912100	-0.68847600
H	14.09534400	1.45154600	-2.40593400
H	10.43042600	0.27643900	1.95445400
H	6.38790100	-1.97262000	-1.56158700
H	2.58596000	2.30855700	-0.55839700
H	-1.18628200	-1.16445400	2.18789200
H	-5.18647100	-0.69823600	-2.00116200
H	-8.96328800	2.11116300	1.41499800
H	-12.81904700	-2.25018000	1.33532200
H	-16.78825300	0.79798300	-1.65331300
H	-20.47688300	0.30980100	0.59273200
C	-19.53251000	1.49364300	-1.90432000
C	-15.54540100	-3.02837400	1.10604100
C	-11.66164600	2.41284000	2.26097000
C	-7.93199500	-0.37668500	-2.66979400
C	-3.89765400	-1.94793200	2.54652300
C	-0.11580500	3.04188300	-0.04740900
C	3.62955900	-2.15420200	-2.23848600
C	7.73929200	-0.17155600	2.76682100
C	11.37535600	2.28895300	-2.39146600
C	15.26153600	-3.08976500	-1.07425100
C	19.30819900	1.55771900	1.69592900

Br	-21.46135000	1.69364500	-1.60171500
Br	-19.27907900	0.54657800	-3.62827600
Br	-18.75548800	3.30798300	-2.10362900
Br	-16.87652700	-3.47708300	-0.27542800
Br	-16.44230100	-3.07547000	2.88390100
Br	-14.22982800	-4.50371200	1.10638600
Br	-12.90209800	1.67664900	3.60323700
Br	-12.65842400	3.70776600	1.12438300
Br	-10.32348400	3.46570700	3.26436000
Br	-9.23036100	1.06961500	-2.99610900
Br	-6.66694800	-0.33618700	-4.18667700
Br	-8.88877000	-2.11920400	-2.78180800
Br	-4.82861600	-0.87117700	3.93809400
Br	-5.19060400	-3.21673300	1.76978500
Br	-2.55268700	-3.04087900	3.49470500
Br	-1.14158800	3.32309000	-1.73042600
Br	-1.33949000	3.29414500	1.47715300
Br	1.20415500	4.50783200	0.05486700
Br	2.31023400	-1.22010700	-3.36657100
Br	2.69381000	-3.60006400	-1.24019700
Br	4.88009500	-3.05130000	-3.47663300
Br	6.46665400	-1.66688200	2.94178600
Br	6.79537800	1.51690200	3.23876700
Br	9.11363300	-0.44942200	4.15683100
Br	10.15918900	3.39383600	-1.30301900
Br	10.32642900	1.47464300	-3.87551600
Br	12.65337300	3.53788000	-3.23288200
Br	13.91099200	-3.04863100	-2.51061800
Br	14.36931700	-3.66767400	0.60945300
Br	16.53359200	-4.52377900	-1.55185900
Br	18.07411800	0.42005400	2.72989600
Br	18.32039200	3.18432900	1.10843400
Br	20.68678800	2.17419200	2.97068400
CCl_3			
C	21.33381700	-0.37916700	-0.36542300
C	20.95636300	0.92563700	-0.48544500
C	19.56084200	1.13830300	-0.23977400
C	18.88755200	-0.04229200	0.04966300
C	17.48963000	-0.35840700	0.34750100
C	17.03858500	-1.05489300	1.44195300
C	15.64086800	-1.34049300	1.42256800
C	15.01633700	-0.84066000	0.28605600
C	13.62880800	-0.84071400	-0.18044200
C	13.19269500	-1.28678000	-1.40416100
C	11.80868600	-1.05034400	-1.65673500
C	11.18050000	-0.41175400	-0.59382200
C	9.80298700	0.02095600	-0.35222600
C	9.41306600	1.28601100	0.01469600

C	8.02423400	1.40851500	0.31640000
C	7.34509500	0.20452900	0.17024500
C	5.94371600	-0.18578600	0.33357400
C	5.48531800	-1.21795800	1.11551700
C	4.08629400	-1.46923100	0.99611200
C	3.46929500	-0.60117400	0.10243300
C	2.08563800	-0.43237400	-0.34418000
C	1.66430600	-0.39910900	-1.65150100
C	0.28240500	-0.08618800	-1.81624000
C	-0.36008400	0.11844200	-0.60050400
C	-1.74091000	0.43284800	-0.23051600
C	-2.13412800	1.46270900	0.58956100
C	-3.52560600	1.46697100	0.90228700
C	-4.20473600	0.41436800	0.29899000
C	-5.60730200	-0.00329400	0.28585500
C	-6.06935800	-1.26406500	0.57762000
C	-7.46724800	-1.44360600	0.35874100
C	-8.08083200	-0.28596600	-0.10663100
C	-9.46165500	0.05472400	-0.45188200
C	-9.87168900	0.63458900	-1.62827000
C	-11.25107200	0.99561600	-1.65702700
C	-11.90462000	0.67596700	-0.47193100
C	-13.28650200	0.81920300	-0.01197800
C	-13.67765100	1.39960000	1.17048600
C	-15.07260000	1.28794600	1.44730600
C	-15.75640800	0.61095600	0.44438700
C	-17.16481100	0.26608800	0.24203400
C	-17.66366300	-0.99057100	0.01071000
C	-19.06902100	-1.00358200	-0.25572300
C	-19.61438600	0.25416400	-0.23774100
S	19.99650400	-1.39278300	0.02934700
S	16.17069100	-0.04343000	-0.74999400
S	12.31396500	-0.09954900	0.69382200
S	8.43657700	-1.06270700	-0.32292500
S	4.63124800	0.50135700	-0.58692200
S	0.75770900	-0.06442300	0.72529300
S	-3.10895300	-0.57148500	-0.63315500
S	-6.91661600	1.00064700	-0.28028800
S	-10.79935000	-0.05365300	0.66257100
S	-14.66206100	0.10807300	-0.81612700
S	-18.43939500	1.46730900	0.09995900
H	22.32468000	-0.79540800	-0.48049600
H	21.64620600	1.72147300	-0.72675600
H	17.69638500	-1.35109200	2.24662600
H	13.85101800	-1.77936700	-2.10561500
H	10.10808300	2.11237000	0.06118600
H	6.13818200	-1.78052600	1.76764900
H	2.33211800	-0.60037500	-2.47712200

H	-1.43896300	2.20454900	0.95615500
H	-5.41935600	-2.04444000	0.94715100
H	-9.19730400	0.79497500	-2.45743400
H	-12.97901300	1.90065800	1.82554200
H	-17.05111600	-1.88258700	0.05031000
H	-20.64560500	0.53024000	-0.39849800
C	-19.82236600	-2.27953100	-0.50700800
C	-15.69796700	1.89021900	2.68637500
C	-11.89575700	1.62655000	-2.87187200
C	-8.16779400	-2.75376000	0.64553400
C	-4.15455100	2.53466600	1.77084300
C	-0.37524400	-0.03000000	-3.17787100
C	3.38874800	-2.54661400	1.79741600
C	7.39763700	2.72856300	0.70967700
C	11.13442700	-1.49662800	-2.93564500
C	14.95248300	-2.07477600	2.55223300
C	18.92619400	2.50837700	-0.32952700
Cl	-19.70105200	-3.37010300	0.94165900
Cl	-21.56704400	-1.99095300	-0.84963000
Cl	-19.10330900	-3.15766300	-1.92596700
Cl	-16.46341300	3.49453100	2.28041400
Cl	-14.46228800	2.19705200	3.97472900
Cl	-16.95378200	0.81685100	3.41162200
Cl	-13.00854400	2.97716600	-2.42583400
Cl	-12.83882600	0.37367700	-3.80038700
Cl	-10.66230400	2.31433600	-4.00408900
Cl	-9.04065800	-2.65700700	2.24274100
Cl	-9.35942800	-3.18379200	-0.64119000
Cl	-6.99658100	-4.12868700	0.75834200
Cl	-5.34817500	1.84559500	2.93846000
Cl	-5.00040800	3.76783700	0.72904400
Cl	-2.91531000	3.41924600	2.74838200
Cl	-1.28975700	-1.57526000	-3.49109500
Cl	-1.51849500	1.35987000	-3.32652600
Cl	0.84400600	0.14972300	-4.50286600
Cl	2.18680300	-3.46163600	0.80787700
Cl	2.52899400	-1.80425600	3.22296900
Cl	4.56160100	-3.75943400	2.45212500
Cl	6.56078200	3.47178400	-0.72899200
Cl	8.63944600	3.91426900	1.28054200
Cl	6.19699500	2.54374500	2.04605100
Cl	9.98255100	-0.25847600	-3.56884000
Cl	10.22375800	-3.04956300	-2.64898800
Cl	12.33692400	-1.81469900	-4.25013900
Cl	13.73340600	-3.26459600	1.95157500
Cl	14.11477900	-0.88524200	3.65017300
Cl	16.13294100	-3.00072400	3.56481300
Cl	17.72515800	2.79832100	0.98815300

Cl	18.08480100	2.69985000	-1.93688000
Cl	20.15985000	3.82759200	-0.21120200
CF_3			
C	-21.40156000	0.31523300	0.36654700
C	-20.91469000	1.59062300	0.38371400
C	-19.49963800	1.64575300	0.21506600
C	-18.91164200	0.40007700	0.07190400
C	-17.52913600	-0.00689600	-0.13833100
C	-17.09655400	-1.05563300	-0.91994600
C	-15.69559500	-1.26987900	-0.87026700
C	-15.03084600	-0.37746200	-0.04327900
C	-13.62545300	-0.23186700	0.30332500
C	-13.12672100	0.17449100	1.52256500
C	-11.71630700	0.31059200	1.55075000
C	-11.10913800	0.00540900	0.34148500
C	-9.71743200	0.02086700	-0.08140400
C	-9.25526700	0.33534400	-1.34195500
C	-7.85329600	0.19423800	-1.49229700
C	-7.21573400	-0.23203400	-0.33674400
C	-5.81836400	-0.50646000	-0.03994700
C	-5.34424500	-1.47547500	0.81857700
C	-3.93693300	-1.45730000	0.98491800
C	-3.30803100	-0.46527800	0.24735000
C	-1.91098700	-0.07769500	0.12819300
C	-1.43719000	1.20586900	-0.04138400
C	-0.03176800	1.28267400	-0.20616000
C	0.59596400	0.04676300	-0.16257500
C	1.99314600	-0.33829900	-0.28967300
C	2.47104400	-1.48778100	-0.88190500
C	3.87727000	-1.63962000	-0.78923100
C	4.50064200	-0.59804400	-0.11851000
C	5.89550600	-0.34711300	0.21016500
C	6.36274600	0.26449300	1.35371500
C	7.76663100	0.45799200	1.36951100
C	8.39975600	-0.00883100	0.22710100
C	9.79577900	-0.00381200	-0.18188500
C	10.26485700	0.14685000	-1.46954300
C	11.67314000	0.03601400	-1.58462100
C	12.30745000	-0.20219500	-0.37471600
C	13.70955000	-0.38515700	-0.03126700
C	14.20690000	-1.23045800	0.93685500
C	15.61070000	-1.13697800	1.11443600
C	16.21083000	-0.21054400	0.27628000
C	17.59650500	0.21270000	0.12595700
C	18.03381900	1.49287300	-0.13123000
C	19.44471500	1.57949200	-0.29431400
C	20.07301100	0.37001300	-0.16421800
S	-20.13772500	-0.84639300	0.16341400

S	-16.16893800	0.73974000	0.67061500
S	-12.31427600	-0.46303500	-0.83346500
S	-8.38093000	-0.45153400	0.94591200
S	-4.48754700	0.44274200	-0.66658100
S	-0.58231000	-1.21706500	0.09285400
S	3.31780600	0.58175100	0.39072800
S	7.22862300	-0.70315100	-0.86738800
S	11.13233200	-0.28029500	0.91474700
S	15.01228700	0.54029200	-0.74680400
S	18.95549100	-0.90171000	0.15339800
H	-22.42819600	-0.00937900	0.46048400
H	-21.53107600	2.47267600	0.49582700
H	-17.76348800	-1.65915500	-1.52173600
H	-13.75313100	0.36861400	2.38350100
H	-9.90196400	0.66637900	-2.14419600
H	-5.98618300	-2.18882600	1.31906700
H	-2.07777800	2.07826000	-0.04312300
H	1.83312800	-2.20758600	-1.37827700
H	5.71826600	0.56682900	2.16893800
H	9.61875700	0.33830000	-2.31655800
H	13.58525400	-1.90769900	1.50813000
H	17.36945200	2.34730400	-0.18001000
H	21.13228400	0.16842800	-0.23162300
C	20.15730000	2.86817300	-0.55747200
C	16.33225400	-1.97447200	2.12943900
C	12.36666100	0.16134500	-2.90966500
C	8.45404300	1.12467900	2.52525400
C	4.57905400	-2.83446300	-1.36554600
C	0.66355900	2.59618400	-0.41626100
C	-3.24095900	-2.42560600	1.89642200
C	-7.17105700	0.47855900	-2.79859100
C	-10.99615800	0.75924600	2.78877500
C	-15.04260900	-2.37555000	-1.64654600
C	-18.76049700	2.95059900	0.17682100
F	21.48218500	2.68052900	-0.72908800
F	19.68115800	3.47666900	-1.66729400
F	19.99699400	3.74259600	0.46169200
F	17.15319400	-1.23988300	2.90857500
F	17.09750400	-2.93038100	1.54391200
F	15.46161100	-2.61033900	2.94104700
F	13.08120600	1.31212100	-2.99823100
F	13.22988800	-0.85180100	-3.13174300
F	11.47856800	0.17010100	-3.92481600
F	9.26477500	2.12728500	2.12679900
F	9.22314100	0.25321600	3.22679300
F	7.55855900	1.64376200	3.38979900
F	5.41038900	-3.41629400	-0.47587100
F	5.33094600	-2.50451200	-2.44668400

F	3.69562000	-3.76946500	-1.77031700
F	1.49818300	2.56806200	-1.47642000
F	1.40976500	2.95010600	0.66136300
F	-0.22507000	3.58910400	-0.62313500
F	-2.40769900	-1.80550000	2.75810000
F	-2.49237100	-3.32196300	1.20424200
F	-4.12869800	-3.13250300	2.62481400
F	-6.40362900	1.59691300	-2.73448300
F	-8.06972900	0.67351900	-3.78472400
F	-6.35911000	-0.52986300	-3.17979400
F	-11.86442900	1.15834700	3.74008300
F	-10.15608000	1.78745100	2.54639000
F	-10.24909600	-0.23858200	3.32734600
F	-14.23107400	-3.13069100	-0.87626200
F	-14.28245400	-1.89727900	-2.66495800
F	-15.96300700	-3.19560600	-2.19391200
F	-17.99016800	3.12724200	1.28171900
F	-17.93697200	3.04321500	-0.88900800
F	-19.61437700	3.99341400	0.12260400
CHO			
C	-21.01272500	0.12221700	-0.31460600
C	-20.54627700	1.40074200	-0.28787500
C	-19.12935400	1.47850100	-0.09543600
C	-18.52977300	0.22741600	0.01459400
C	-17.13802800	-0.15716300	0.20105000
C	-16.64824600	-1.19449000	0.95492500
C	-15.23565100	-1.36333200	0.86706300
C	-14.62800600	-0.43194000	0.02896800
C	-13.23257300	-0.22397200	-0.32450500
C	-12.72892700	0.17828300	-1.53723900
C	-11.31749700	0.37164700	-1.54720700
C	-10.72414600	0.10945700	-0.31487300
C	-9.33437600	0.15501600	0.11080900
C	-8.84841800	0.53765800	1.33706300
C	-7.43747100	0.39715400	1.47444300
C	-6.82666100	-0.10252300	0.32681500
C	-5.43162400	-0.37773000	0.02275200
C	-4.93208600	-1.39330700	-0.75542100
C	-3.51935100	-1.35120900	-0.93223300
C	-2.92071900	-0.28038300	-0.27282000
C	-1.52879100	0.12610000	-0.16686500
C	-1.03605600	1.40685100	-0.10804300
C	0.37449700	1.48060800	0.07539600
C	0.97833400	0.22814400	0.15655500
C	2.37090100	-0.15843600	0.31550500
C	2.86349400	-1.23867800	1.00608400
C	4.27563300	-1.39766000	0.90903700
C	4.88073300	-0.41563800	0.12823500

C	6.27415000	-0.18495400	-0.21663000
C	6.76932000	0.30880500	-1.39886800
C	8.18112500	0.49650900	-1.40609000
C	8.78340300	0.13647900	-0.20300900
C	10.17649100	0.14421900	0.21329600
C	10.67305400	0.42480300	1.46269800
C	12.08456200	0.27053100	1.57654500
C	12.68467300	-0.13566800	0.38717000
C	14.07623300	-0.39091200	0.05123700
C	14.56532300	-1.34631700	-0.80550800
C	15.97689400	-1.29401800	-0.99098900
C	16.58380800	-0.27774800	-0.25848800
C	17.98029800	0.11302100	-0.13586800
C	18.49054300	1.38511500	-0.04283800
C	19.90780800	1.41629500	0.12730500
C	20.46273000	0.15672500	0.17057200
S	-19.73078200	-1.03458500	-0.12785200
S	-15.82355200	0.64543600	-0.64953900
S	-11.93155700	-0.37357300	0.85108800
S	-8.01713400	-0.39725900	-0.91695700
S	-4.12495000	0.66981500	0.56288200
S	-0.21833200	-1.03548900	0.00588600
S	3.68333500	0.70022400	-0.48214600
S	7.58395700	-0.43275800	0.93210700
S	11.48390000	-0.32432700	-0.86720400
S	15.39141200	0.60949000	0.65753400
S	19.27509200	-1.07726800	0.01437000
H	-22.03144700	-0.22028400	-0.43297900
H	-21.16112600	2.28693800	-0.38068800
H	-17.26283500	-1.83946800	1.57085300
H	-13.33171300	0.33848600	-2.42291800
H	-9.46333200	0.91901500	2.14328400
H	-5.53794900	-2.17125800	-1.20410900
H	-1.64519800	2.29855200	-0.19437700
H	2.25305500	-1.92266000	1.58331200
H	6.16085200	0.54010500	-2.26484100
H	10.06600100	0.74064300	2.30258900
H	13.95295900	-2.08665100	-1.30609500
H	17.89365300	2.28626800	-0.11519800
H	21.50759500	-0.10225100	0.28238400
C	20.69410100	2.65849500	0.23429900
O	20.20431300	3.77045300	0.19142800
H	21.78809000	2.51280500	0.35962800
C	16.65113100	-2.20119000	-1.94144300
O	16.06596900	-3.09591800	-2.52424100
H	17.72721800	-2.01394300	-2.11860000
C	12.76337300	0.45771400	2.87476400
O	12.18105600	0.84704100	3.87065000

H	13.83975800	0.20316100	2.90787800
C	8.85787300	1.09457000	-2.57486400
O	8.27297100	1.35402400	-3.61068800
H	9.93471200	1.32040700	-2.45736200
C	4.94938400	-2.55639600	1.52936200
O	4.36188500	-3.35990900	2.23053800
H	6.02665600	-2.67631100	1.30680300
C	1.04827600	2.78490500	0.23763500
O	0.46323200	3.84598700	0.11846700
H	2.12321200	2.75409000	0.49852600
C	-2.83806100	-2.32674800	-1.80707000
O	-3.41950600	-3.26183200	-2.32671400
H	-1.76043700	-2.15441500	-1.99023000
C	-6.76948500	0.69197100	2.75804000
O	-7.35951700	1.16151300	3.71403400
H	-5.69315600	0.44246000	2.82072300
C	-10.63098300	0.87850900	-2.75215000
O	-11.20356600	1.05138700	-3.81282200
H	-9.55656700	1.11934300	-2.64041000
C	-14.56079500	-2.48167300	1.55377100
O	-15.14377000	-3.24565000	2.30211800
H	-13.48305000	-2.61137800	1.33717900
C	-18.44900700	2.77722700	0.05541300
O	-19.01962300	3.84547700	-0.07948200
H	-17.37505500	2.73883800	0.32283200
COOH			
C	-21.30264300	0.20884100	-0.24722100
C	-20.93211300	-1.06887000	0.03334700
C	-19.51401100	-1.23200800	0.17068900
C	-18.80335300	-0.04127500	0.00572700
C	-17.39923900	0.32981900	0.10002100
C	-16.94425300	1.61620300	0.29521700
C	-15.53261000	1.76504900	0.25205700
C	-14.86976300	0.56007400	-0.00437600
C	-13.47610300	0.19431900	-0.19553800
C	-13.05478600	-0.96494400	-0.81406200
C	-11.65027000	-1.16837900	-0.81156800
C	-10.95662200	-0.14577800	-0.15482600
C	-9.55593100	0.08653900	0.15571900
C	-9.10922800	0.94556700	1.13885100
C	-7.69974100	1.09902000	1.20408900
C	-7.02838500	0.35223500	0.22934000
C	-5.63251000	0.20874400	-0.14925700
C	-5.20382300	-0.26558900	-1.37201200
C	-3.79803500	-0.41609400	-1.49625800
C	-3.11113300	-0.06726200	-0.32785300
C	-1.71274500	-0.09279400	0.06711300
C	-1.27415100	-0.07536300	1.37543400

C	0.13462400	-0.00917400	1.53382700
C	0.81434600	0.04652400	0.31181500
C	2.21354300	0.17195700	-0.06070700
C	2.65388500	0.59890800	-1.29671600
C	4.06059200	0.55247700	-1.47986700
C	4.73623600	0.05547800	-0.35945300
C	6.13012400	-0.23260000	-0.06585100
C	6.55425100	-1.06832100	0.94693100
C	7.96146200	-1.13452600	1.11920300
C	8.65549000	-0.31079800	0.22544800
C	10.05942600	0.00899400	0.02842600
C	10.51594000	1.12193500	-0.64749700
C	11.92452500	1.18848800	-0.80930000
C	12.58529800	0.08430000	-0.25904900
C	13.97508300	-0.33785200	-0.20606400
C	14.38527000	-1.63221700	0.03975400
C	15.79056200	-1.80510500	0.14608500
C	16.49435800	-0.60574000	-0.00146700
C	17.90137500	-0.24201900	0.07874100
C	18.36499400	1.03016300	0.36001700
C	19.77741600	1.15912600	0.26786800
C	20.38469200	-0.02415500	-0.08926100
S	-19.92846300	1.25657200	-0.36124200
S	-16.02830200	-0.74990400	-0.10858600
S	-12.07644700	1.08908900	0.38183900
S	-8.17605100	-0.58948600	-0.69952000
S	-4.23878600	0.51727100	0.87794500
S	-0.32528700	-0.06347600	-1.01331700
S	3.59716100	-0.26999600	0.93058100
S	7.52991500	0.47220400	-0.86433300
S	11.43045900	-0.97770200	0.51902000
S	15.38636900	0.70256500	-0.34952600
S	19.26450700	-1.30520800	-0.28546800
H	-22.29701600	0.60249400	-0.40393900
H	-21.62504900	-1.89215800	0.14258300
H	-17.59974700	2.45693400	0.47971100
H	-13.73104700	-1.66910000	-1.28005500
H	-9.77067000	1.46142800	1.82206700
H	-5.87594200	-0.49820700	-2.18714200
H	-1.94138900	-0.11970100	2.22583500
H	1.98977900	0.95517500	-2.07281500
H	5.87755400	-1.64459700	1.56361100
H	9.86240000	1.89838200	-1.02205600
H	13.70006300	-2.46391300	0.13591900
H	17.71697700	1.85112400	0.63872000
H	21.44756700	-0.18810400	-0.20224700
C	20.56085000	2.38180200	0.52813400
O	21.76978200	2.46995100	0.44352800

C	16.38552700	-3.12819100	0.39032000
O	17.56772300	-3.41316200	0.32209500
C	12.56499400	2.32224100	-1.49207300
O	13.76079500	2.54898000	-1.54859500
C	8.56766800	-1.99634700	2.14474400
O	9.75467300	-2.23846300	2.27479000
C	4.68442400	0.99469400	-2.73579700
O	5.87740100	1.13882600	-2.93742000
C	0.75687000	-0.00347100	2.86624400
O	1.94683900	-0.10530400	3.10737300
C	-3.18583000	-0.89148600	-2.74590500
O	-1.99554300	-0.90735500	-3.00584700
C	-7.06861000	1.96550300	2.21075500
O	-5.87632000	2.04466900	2.44879900
C	-11.04549900	-2.34875600	-1.44706600
O	-9.85659700	-2.53682100	-1.63528700
C	-14.89339600	3.07248700	0.46121100
O	-13.70112200	3.27857900	0.60265500
C	-18.91768800	-2.54268900	0.46672700
O	-17.73165700	-2.82027100	0.43806900
O	19.77668500	3.43305700	0.88093700
H	20.38345500	4.17791900	1.02549400
O	15.45155800	-4.06250900	0.70433700
H	15.94149100	-4.89315100	0.82147000
O	11.66269800	3.14935800	-2.07830800
H	12.18394200	3.86496700	-2.47826700
O	7.64249600	-2.54058400	2.97509000
H	8.14109200	-3.09981600	3.59349900
O	3.76957500	1.25565800	-3.70330200
H	4.27975200	1.55510500	-4.47391900
O	-0.15626800	0.11943400	3.86219900
H	0.35161100	0.09263300	4.68994100
O	-4.10882800	-1.32442700	-3.64110000
H	-3.60658300	-1.59444200	-4.42766500
O	-7.97592700	2.70264500	2.89926800
H	-7.46271100	3.21859100	3.54289700
O	-11.97425000	-3.25702300	-1.83844400
H	-11.47688500	-3.97901200	-2.25705400
O	-15.79361300	4.08779100	0.49730700
H	-15.27497300	4.89332100	0.65879500
O	-19.85284600	-3.47508700	0.77961000
H	-19.36104200	-4.29841600	0.93548900
*i*Pr			
C	-21.15198600	-0.02791400	-0.36674800
C	-20.71303700	-1.29206600	-0.09779200
C	-19.29837800	-1.38637500	0.11576400
C	-18.67708500	-0.15545400	-0.00594600
C	-17.27893800	0.23634500	0.13046300

C	-16.78275900	1.36741600	0.73729500
C	-15.36871300	1.53731800	0.64270500
C	-14.76910300	0.50278900	-0.05655000
C	-13.37482200	0.24635100	-0.39218000
C	-12.88045900	-0.26501800	-1.57116100
C	-11.47145300	-0.48931900	-1.59314500
C	-10.87280000	-0.13720900	-0.39438300
C	-9.48209900	-0.17474300	0.03770500
C	-9.00386300	-0.51850200	1.28244600
C	-7.59424500	-0.37777500	1.45257200
C	-6.97814800	0.08604300	0.30141700
C	-5.58137000	0.36999300	0.00147100
C	-5.08557500	1.40797200	-0.75559100
C	-3.67328300	1.39363600	-0.95705300
C	-3.07265600	0.31176300	-0.33306500
C	-1.67924000	-0.10317900	-0.24873600
C	-1.18957800	-1.39023700	-0.26809100
C	0.21921600	-1.50852300	-0.07657300
C	0.82394300	-0.27314800	0.09228700
C	2.21525400	0.10201900	0.30268500
C	2.69275300	1.11357300	1.10606600
C	4.10346500	1.31862800	1.05316700
C	4.72270700	0.43422800	0.18406800
C	6.12111000	0.25568200	-0.18031200
C	6.62306900	-0.08407600	-1.41718200
C	8.03488400	-0.28203300	-1.46785600
C	8.62999400	-0.08371600	-0.23228600
C	10.02069900	-0.15718400	0.19370500
C	10.50129400	-0.63530700	1.39244400
C	11.90940900	-0.50331600	1.57982500
C	12.52304900	0.09218900	0.48949200
C	13.91782900	0.41722800	0.22425200
C	14.40609200	1.53018900	-0.42310200
C	15.81919400	1.54678600	-0.62205700
C	16.42694100	0.41345800	-0.10845700
C	17.82587800	0.00429200	-0.06396800
C	18.33693400	-1.25635600	-0.27574000
C	19.75179400	-1.36398200	-0.08692900
C	20.30069400	-0.16204600	0.27705400
S	-19.84543800	1.10497600	-0.38814400
S	-15.96639200	-0.66721600	-0.59714800
S	-12.06505100	0.46136400	0.75191600
S	-8.15469400	0.34958100	-0.97913400
S	-4.26784200	-0.68117500	0.49175000
S	-0.36326400	1.02287500	0.01813900
S	3.54582200	-0.63805100	-0.56479800
S	7.42984000	0.33851600	0.98252400
S	11.34498100	0.48915600	-0.75515000

S	15.24022600	-0.66668900	0.60953900
S	19.11798000	1.09928600	0.40707600
H	-22.16413200	0.30282200	-0.55431300
H	-21.38058500	-2.14438500	-0.04018100
H	-17.43068000	2.06773000	1.25249700
H	-13.52606400	-0.46888000	-2.41813500
H	-9.66239500	-0.87651900	2.06596100
H	-5.73284800	2.18021800	-1.15605100
H	-1.83868000	-2.24408800	-0.42762100
H	2.03287300	1.69945600	1.73612700
H	5.98060300	-0.18340900	-2.28504500
H	9.84625000	-1.08586200	2.12976500
H	13.75333400	2.33389000	-0.74527200
H	17.70608500	-2.08400700	-0.58100800
H	21.34311700	0.05820400	0.46496400
C	20.54896400	-2.63939500	-0.27792000
C	20.47536300	-3.14284800	-1.73144400
C	20.10822700	-3.73484800	0.71077800
H	21.59817000	-2.39881400	-0.06203500
H	20.82037500	-2.37790100	-2.43348800
H	21.09773200	-4.03453600	-1.86318700
H	19.44917800	-3.41045500	-2.00628800
H	20.19730100	-3.39284600	1.74619100
H	19.06462500	-4.02275900	0.54263200
H	20.72398100	-4.63291200	0.59214300
C	16.53007800	2.68036100	-1.33890500
C	16.51502000	3.97419700	-0.50196500
C	15.93717000	2.92257800	-2.74000100
H	17.57537000	2.38508700	-1.47475400
H	16.98255600	3.82190600	0.47551800
H	17.05820500	4.77282600	-1.01866800
H	15.49039000	4.32263400	-0.33083000
H	15.96265700	2.01114400	-3.34458900
H	14.89639700	3.25983000	-2.68473500
H	16.50694300	3.69663200	-3.26507700
C	12.60702300	-0.95338700	2.85097200
C	12.62482600	-2.48984600	2.96903700
C	11.97537600	-0.31067000	4.10036400
H	13.64595300	-0.61250200	2.80155200
H	13.11659200	-2.95002700	2.10667000
H	13.16112400	-2.79994700	3.87238500
H	11.60779800	-2.89332800	3.02783800
H	11.97673600	0.78098600	4.02849800
H	10.93934000	-0.63679100	4.24194800
H	12.53582900	-0.59404000	4.99754900
C	8.75536800	-0.68861700	-2.74093300
C	8.78657200	0.46413600	-3.76329900
C	8.13909000	-1.95815700	-3.35842600

H	9.79083900	-0.92664100	-2.47746500
H	9.27211800	1.35230400	-3.34776700
H	9.33543200	0.16548400	-4.66296900
H	7.77359300	0.74969400	-4.06791900
H	8.13094300	-2.78436500	-2.64137500
H	7.10805500	-1.78794000	-3.68662100
H	8.71628800	-2.27219600	-4.23451500
C	4.79900900	2.40257300	1.85710400
C	4.81980000	2.06042900	3.35974100
C	4.16416500	3.78498600	1.61502100
H	5.83720600	2.45919400	1.51495600
H	5.31463800	1.10200600	3.54355100
H	5.35396600	2.83319500	3.92302500
H	3.80313800	1.99343200	3.76293300
H	4.16116000	4.03982300	0.55110000
H	3.12960500	3.82252600	1.97271600
H	4.72562900	4.55860000	2.14944400
C	0.92640400	-2.85162800	-0.04589700
C	0.96020700	-3.50000900	-1.44357500
C	0.29318200	-3.80130100	0.98857600
H	1.96161200	-2.67833700	0.26483300
H	1.45708800	-2.85167200	-2.17163100
H	1.49906800	-4.45316400	-1.41292400
H	-0.05276000	-3.69878400	-1.81080300
H	0.28029800	-3.35040800	1.98525500
H	-0.73787200	-4.05819900	0.72276900
H	0.86194900	-4.73550100	1.04415700
C	-2.95607400	2.44844000	-1.78048400
C	-2.91648500	3.80304000	-1.04605000
C	-3.58041400	2.59757400	-3.18046300
H	-1.92228400	2.11683900	-1.92040800
H	-2.42741500	3.71376100	-0.07131500
H	-2.36683400	4.54454900	-1.63586000
H	-3.92738800	4.19061200	-0.87763500
H	-3.59874600	1.64100900	-3.71103600
H	-4.60836300	2.97204600	-3.12654300
H	-3.00215100	3.30884100	-3.77960600
C	-6.89526900	-0.68787100	2.76407900
C	-6.86776700	-2.20349900	3.04230100
C	-7.53052400	0.07805700	3.93980600
H	-5.85827900	-0.34777100	2.67837800
H	-6.37231600	-2.74827300	2.23295800
H	-6.33024200	-2.41393300	3.97323100
H	-7.88213400	-2.60544000	3.14279100
H	-7.53576600	1.15624400	3.75443600
H	-8.56449200	-0.23758300	4.11599300
H	-6.96808500	-0.10687700	4.86111000
C	-10.75558600	-1.06507100	-2.80179400

C	-10.70111200	-0.04801500	-3.95848300
C	-11.39309100	-2.39016000	-3.26055000
H	-9.72516100	-1.28676700	-2.50587400
H	-10.20429300	0.87747900	-3.65206900
H	-10.15193700	-0.46282800	-4.81063600
H	-11.70822600	0.21239400	-4.30260200
H	-11.41880500	-3.12061100	-2.44651000
H	-12.41968600	-2.24380300	-3.61318300
H	-10.81890500	-2.82133000	-4.08741600
C	-14.65165600	2.74017500	1.22882700
C	-14.65979200	2.70481700	2.76951700
C	-15.24362700	4.06176200	0.70333100
H	-13.60783100	2.69994400	0.90171800
H	-14.19488300	1.79000300	3.14938400
H	-14.11024600	3.56034000	3.17682800
H	-15.68251400	2.74659500	3.16042800
H	-15.22194900	4.09890300	-0.38982200
H	-16.28289300	4.19389000	1.02307700
H	-14.67009900	4.91309200	1.08513400
C	-18.60996400	-2.69129600	0.47280100
C	-18.59874100	-3.66667200	-0.72022400
C	-19.24548000	-3.34480700	1.71455700
H	-17.56913500	-2.46253700	0.72345300
H	-18.10096800	-3.22637500	-1.58947600
H	-18.07178200	-4.59045900	-0.45778100
H	-19.61748800	-3.93626000	-1.02060100
H	-19.23798100	-2.66207800	2.56937000
H	-20.28429200	-3.63882100	1.52877800
H	-18.69248800	-4.24786500	1.99421500
Li			
C	-21.01130000	0.15172900	0.08909600
C	-20.44726900	0.96232200	1.04028700
C	-19.01089800	0.96863700	1.08876800
C	-18.52367500	0.11154400	0.11142900
C	-17.13125300	-0.21455800	-0.15218900
C	-16.51497300	-1.37661400	-0.56259400
C	-15.07948500	-1.39234700	-0.58130000
C	-14.58304100	-0.17659700	-0.14665400
C	-13.19906800	0.23469000	-0.03161200
C	-12.57641400	1.45726200	-0.17662700
C	-11.14206900	1.47471800	-0.13953700
C	-10.65114900	0.19368900	0.03346700
C	-9.27056900	-0.23171400	0.13368900
C	-8.65950500	-1.25628100	0.82658900
C	-7.22517400	-1.29853000	0.81049000
C	-6.72278000	-0.24159400	0.07444400
C	-5.33879000	0.08051200	-0.20552200
C	-4.72257300	0.66762900	-1.29102000

C	-3.28804600	0.70238800	-1.29459800
C	-2.79156200	0.09735700	-0.15515800
C	-1.40867300	-0.06817100	0.24276800
C	-0.79257100	-0.09291900	1.47662000
C	0.64204900	-0.12551600	1.49547400
C	1.13794800	-0.11335500	0.20531900
C	2.52064800	-0.14601100	-0.22527000
C	3.13702300	-0.68859600	-1.33340900
C	4.57134300	-0.65765600	-1.36984900
C	5.06644500	-0.07094800	-0.22039900
C	6.44816600	0.17288900	0.13912100
C	7.05501500	1.17001600	0.87387100
C	8.48955700	1.18181100	0.90771600
C	8.99444400	0.14374300	0.14736800
C	10.38043200	-0.21262500	-0.07576600
C	11.00756500	-1.42335200	-0.28366300
C	12.44207600	-1.42194900	-0.32608900
C	12.92506900	-0.14238900	-0.12715200
C	14.30389300	0.30216700	-0.10984600
C	14.90616500	1.48106100	-0.49445900
C	16.34150700	1.52405000	-0.47164600
C	16.84877300	0.30873400	-0.05798000
C	18.23913200	-0.07114000	0.11772400
C	18.86538600	-0.91926900	1.00055400
C	20.31404500	-0.94675900	0.97260400
C	20.77222600	-0.06437900	0.03072500
S	-19.80710700	-0.67834200	-0.84570500
S	-15.89205500	1.01710500	0.18327100
S	-11.96951700	-1.01802600	0.25552900
S	-8.02981900	0.74385800	-0.68471800
S	-4.10354500	-0.42100200	0.97019000
S	-0.17421100	-0.16709300	-1.03192900
S	3.75331800	0.53544900	0.85810600
S	7.69202700	-0.90807100	-0.52493200
S	11.60164000	1.07517500	0.00880100
S	15.55230000	-0.85699100	0.39767700
S	19.46623300	0.72122500	-0.88906600
H	-22.06116700	-0.00689100	-0.12477400
H	-21.06885100	1.55189100	1.71072900
H	-17.11949400	-2.24491200	-0.81303900
H	-13.17271500	2.34747300	-0.36280000
H	-9.26409600	-1.95976900	1.39447700
H	-5.32358200	1.03595500	-2.11931500
H	-1.39306200	-0.03723100	2.38177200
H	2.53720700	-1.15809300	-2.10980500
H	6.44737500	1.93233900	1.35610700
H	10.41619800	-2.33295700	-0.35930600
H	14.29641300	2.31035900	-0.84523400

H	18.27496000	-1.48927500	1.71429200
H	21.78530600	0.17239600	-0.27289300
Li	21.56949700	-2.00838300	2.06230100
Li	17.96459900	2.67310600	-0.63974800
Li	14.09673000	-2.45197900	-0.76566200
Li	10.12782800	1.95265300	1.75472600
Li	6.21602400	-0.92002100	-2.47568000
Li	2.29321000	-0.37677200	2.59332200
Li	-1.63811000	1.43553900	-2.15195000
Li	-5.57898700	-2.35141800	1.22641000
Li	-9.48916600	2.58979900	-0.03373500
Li	-13.43066900	-2.33553700	-1.19049000
Li	-17.36133400	1.45435800	2.08033500
*n*Bu			
C	-21.31514800	0.32874900	0.56537600
C	-20.84106600	1.51989900	0.09719100
C	-19.42490900	1.53993300	-0.12241700
C	-18.83626900	0.32868700	0.19676900
C	-17.45023200	-0.11935600	0.13039200
C	-16.98920900	-1.35369300	-0.26649100
C	-15.57931200	-1.54244200	-0.15176100
C	-14.94422700	-0.41782100	0.34733700
C	-13.54047800	-0.13742900	0.61579900
C	-13.02098600	0.59184300	1.66170000
C	-11.60748500	0.78260700	1.62857900
C	-11.02837900	0.18012100	0.52446100
C	-9.64326700	0.09577600	0.08205700
C	-9.17828300	0.15002000	-1.21283800
C	-7.77333900	-0.04686200	-1.36363700
C	-7.14596300	-0.25705000	-0.14775600
C	-5.74626300	-0.48318600	0.18834600
C	-5.24098600	-1.36643300	1.11522100
C	-3.82602100	-1.31712900	1.29319700
C	-3.23412100	-0.36564500	0.48040400
C	-1.84148900	0.02988000	0.31386500
C	-1.34877300	1.30698600	0.17207000
C	0.05710400	1.39483600	-0.05580100
C	0.65566300	0.14646300	-0.09135600
C	2.04188400	-0.25588600	-0.28799500
C	2.50125100	-1.34450700	-0.99499300
C	3.91376600	-1.54113200	-0.95952800
C	4.55313700	-0.57474000	-0.20182100
C	5.96204900	-0.35887400	0.10010900
C	6.50665200	0.04649600	1.29758000
C	7.91695800	0.26321900	1.28216700
C	8.46586000	0.01699000	0.03526800
C	9.83769700	0.09440500	-0.44937700
C	10.26468700	0.56372700	-1.67094100

C	11.66626100	0.44728600	-1.91140200
C	12.32971200	-0.12996800	-0.84206100
C	13.73892700	-0.43284400	-0.62923600
C	14.27035400	-1.54615400	-0.01874700
C	15.69015400	-1.53915300	0.12790000
C	16.25852200	-0.38696800	-0.38591100
C	17.64846700	0.04675300	-0.47245400
C	18.15133400	1.30374400	-0.22625000
C	19.55326600	1.44359200	-0.47761500
C	20.10203500	0.26991400	-0.92439700
S	-20.03921800	-0.82046500	0.77389700
S	-16.10596500	0.86207200	0.67824900
S	-12.24677300	-0.61255400	-0.46756500
S	-8.30723400	-0.21696100	1.17320200
S	-4.43955400	0.46401100	-0.49561700
S	-0.53412100	-1.12657300	0.15445900
S	3.39328700	0.56619900	0.46727800
S	7.22480500	-0.47642000	-1.10998900
S	11.20598000	-0.52905300	0.45170800
S	15.02679500	0.68333200	-1.04057200
S	18.93195400	-1.00305200	-1.05768600
H	-22.33486900	0.06141400	0.80546400
H	-21.47896100	2.37569100	-0.09635800
H	-17.65503300	-2.11927500	-0.65059900
H	-13.64629000	0.97906000	2.45936300
H	-9.83747300	0.34267600	-2.05280400
H	-5.87754100	-2.05797800	1.65723400
H	-1.98790700	2.18056800	0.24597500
H	1.83286400	-1.99311400	-1.55161400
H	5.90266400	0.17665700	2.18948700
H	9.58045800	1.00377600	-2.38878700
H	13.64913400	-2.37206800	0.31158200
H	17.52773200	2.11146500	0.14237700
H	21.13643800	0.07730700	-1.17612600
C	20.32754700	2.71637200	-0.23976500
C	20.51547200	3.05379600	1.25190700
H	21.04188100	2.22413700	1.74189600
H	19.53223600	3.11726100	1.73704600
C	16.42595900	-2.65853000	0.82590800
C	16.09525100	-2.75441100	2.32888900
H	16.35685500	-1.80406300	2.81210000
H	15.01178000	-2.87453900	2.46113200
C	12.28276300	0.86639000	-3.22523700
C	11.84501400	-0.02001500	-4.40870500
H	12.13924600	-1.05766600	-4.20444500
H	10.74928500	-0.02301500	-4.48110000
C	8.65414400	0.77048000	2.49919900
C	8.28552400	2.22292600	2.86290400

H	8.52272500	2.87387200	2.01141900
H	7.20024700	2.30014200	3.01148900
C	4.56693700	-2.72546000	-1.63195100
C	4.25734400	-4.06233700	-0.92803600
H	4.60658800	-4.00579000	0.11108200
H	3.16999700	-4.20723400	-0.87625500
C	0.74114500	2.72238000	-0.28226500
C	0.32431000	3.40397000	-1.60140200
H	0.56803700	2.73769700	-2.43901900
H	-0.76600600	3.53270400	-1.62196400
C	-3.12413500	-2.17887200	2.31604100
C	-3.44124200	-1.76784800	3.76830000
H	-3.12611900	-0.72735000	3.92017200
H	-4.52800000	-1.78250300	3.92512400
C	-7.11728100	-0.08267400	-2.72385400
C	-7.54510500	-1.30268100	-3.56426800
H	-7.28691100	-2.21903900	-3.01771600
H	-8.63791900	-1.31040000	-3.67143600
C	-10.89723300	1.61352400	2.67119000
C	-11.25655200	3.11134700	2.59419700
H	-10.98380700	3.49203700	1.60137400
H	-12.34458600	3.23328200	2.67835100
C	-14.92661400	-2.86185900	-0.49104700
C	-15.33527200	-4.00069600	0.46496800
H	-15.06174600	-3.71988400	1.49030400
H	-16.42784100	-4.11055500	0.46114600
C	-18.72660400	2.74729100	-0.70204800
C	-19.15070900	3.05846500	-2.15148800
H	-18.92647600	2.18900300	-2.78295400
H	-20.23955400	3.19421700	-2.19650400
C	16.82481900	-3.90720200	3.02860300
H	17.90802400	-3.78343800	2.89580400
H	16.56515100	-4.85344200	2.53445500
C	16.50012900	-4.00462200	4.52218500
H	17.03440500	-4.83540200	4.99434900
H	16.78107600	-3.08598300	5.04942300
H	15.42808500	-4.16320200	4.68522900
C	12.44331400	0.43301800	-5.74581800
H	13.53896400	0.43484000	-5.66982400
H	12.15106300	1.47439300	-5.93863500
C	12.01393100	-0.44539800	-6.92461300
H	12.32323300	-1.48608700	-6.77630200
H	10.92501000	-0.43965000	-7.04797000
H	12.45642600	-0.09801900	-7.86380900
C	9.00973000	2.72826600	4.11628000
H	10.09454800	2.64881200	3.96421400
H	8.77306700	2.06840400	4.96221900
C	8.64854400	4.17215500	4.47728800

H	8.90756100	4.86020700	3.66472900
H	7.57438700	4.27675700	4.66769700
H	9.17917200	4.50404400	5.37550500
C	4.90193500	-5.26662900	-1.62441600
H	5.98832200	-5.11438600	-1.67736100
H	4.55142100	-5.31434900	-2.66452900
C	4.60540200	-6.59535900	-0.92278400
H	4.97547900	-6.58918700	0.10857800
H	3.52802900	-6.79249400	-0.88519100
H	5.07901300	-7.43546200	-1.44090700
C	1.00331200	4.76293400	-1.80756000
H	2.09316600	4.62948200	-1.78400200
H	0.76015500	5.42100600	-0.96209900
C	0.59962000	5.44411400	-3.11861700
H	0.86149300	4.82455700	-3.98369100
H	-0.48089800	5.62322400	-3.15665400
H	1.10074500	6.41013400	-3.23789500
C	-2.76314800	-2.66988600	4.80619500
H	-1.67716300	-2.65673300	4.64292200
H	-3.08133400	-3.70911900	4.64569400
C	-3.06828100	-2.25826400	6.24956800
H	-2.72693000	-1.23654100	6.45015800
H	-4.14469100	-2.29188600	6.45265500
H	-2.57379900	-2.92074900	6.96729900
C	-6.89581900	-1.33032700	-4.95300200
H	-5.80322900	-1.32413300	-4.84166800
H	-7.15099000	-0.40704300	-5.49111400
C	-7.31842800	-2.54238300	-5.78837300
H	-6.84122000	-2.53473700	-6.77363900
H	-7.04340800	-3.48032600	-5.29300500
H	-8.40308400	-2.55784600	-5.94422300
C	-10.56360100	3.94751800	3.67650500
H	-9.47593800	3.82195200	3.58997100
H	-10.83618600	3.55609200	4.66620900
C	-10.91465300	5.43641700	3.60060600
H	-10.40727700	6.00742400	4.38487200
H	-10.62086900	5.86346600	2.63517600
H	-11.99255800	5.59595900	3.71735700
C	-14.68636900	-5.34274800	0.10656500
H	-13.59388300	-5.23016500	0.11493000
H	-14.95538500	-5.61139500	-0.92422900
C	-15.09069300	-6.47697900	1.05295200
H	-14.61242700	-7.42103400	0.77261500
H	-14.80275300	-6.25224000	2.08608300
H	-16.17480400	-6.63683800	1.04045900
C	-18.45838100	4.30255300	-2.72053900
H	-17.36994000	4.16280000	-2.67668900
H	-18.67910400	5.16556500	-2.07744100

C	-18.87568800	4.61750900	-4.16008400
H	-18.36652200	5.51005200	-4.53775400
H	-18.63418300	3.78719400	-4.83315000
H	-19.95482800	4.79532500	-4.23053600
C	21.28328200	4.36090800	1.48088700
H	22.26450500	4.29522100	0.99119900
H	20.75214200	5.18447400	0.98436200
C	21.47326800	4.69499100	2.96346100
H	22.03240900	3.90595000	3.47883000
H	22.02330600	5.63233100	3.09502700
H	20.50861700	4.80107000	3.47237900
H	21.31073500	2.63869600	-0.71903600
H	19.81176400	3.55245500	-0.73153200
H	16.17256500	-3.61245000	0.34318300
H	17.50549900	-2.53240300	0.70866200
H	13.37346200	0.85032400	-3.15734200
H	12.00268900	1.90748700	-3.43675500
H	8.42650600	0.11901000	3.35397900
H	9.73435800	0.70813000	2.34491000
H	5.65094500	-2.59194500	-1.67313400
H	4.22272400	-2.78155600	-2.67351000
H	0.50629800	3.39334800	0.55542200
H	1.82684900	2.59758800	-0.27925900
H	-2.04179200	-2.14377600	2.16728600
H	-3.42246900	-3.22561100	2.16790600
H	-7.37180900	0.83538100	-3.27097500
H	-6.02900800	-0.08769600	-2.62183300
H	-9.81394100	1.50605000	2.57441400
H	-11.15671000	1.23537000	3.66945300
H	-15.19715600	-3.14344100	-1.51806100
H	-13.83819400	-2.76392000	-0.47702300
H	-18.94121900	3.62238800	-0.07290800
H	-17.64262700	2.60986900	-0.67452900
NH_2			
C	-21.00706800	-0.88425600	0.25180200
C	-20.38533900	-1.96414400	0.80566900
C	-18.95604600	-1.85891400	0.80285500
C	-18.50939400	-0.67325700	0.22903500
C	-17.16367200	-0.17843000	0.05746400
C	-16.75655300	1.12895600	-0.09118800
C	-15.35348800	1.29599600	-0.28416900
C	-14.65808100	0.09053400	-0.26265300
C	-13.24447000	-0.17057500	-0.37861100
C	-12.62807200	-1.29399100	-0.88246200
C	-11.20403300	-1.27613700	-0.80679800
C	-10.70181000	-0.10635700	-0.24379800
C	-9.34207300	0.30393200	-0.01402000
C	-8.88501000	1.26677600	0.86167400

C	-7.47599600	1.47581900	0.83325900
C	-6.82234400	0.64127600	-0.06940600
C	-5.41821300	0.50739700	-0.36466400
C	-4.83389800	0.10891700	-1.54662100
C	-3.41151100	0.01802700	-1.50685500
C	-2.87565100	0.36684100	-0.27029700
C	-1.50586300	0.42548600	0.16463600
C	-1.02659900	0.41070700	1.45830200
C	0.38944300	0.51968000	1.56949300
C	1.02588000	0.59973500	0.33380400
C	2.42856300	0.67521400	0.01204000
C	3.01652300	1.25802800	-1.08872400
C	4.43372900	1.11442500	-1.15309700
C	4.96236400	0.41595400	-0.07132000
C	6.32664400	0.08472400	0.24288200
C	6.78524800	-0.89758700	1.09610700
C	8.20257700	-0.95513800	1.22987300
C	8.85998000	-0.01071400	0.44704600
C	10.26982600	0.22882000	0.26336200
C	10.89508700	1.41550800	-0.04781400
C	12.30652700	1.31610700	-0.22738800
C	12.79214900	0.02634200	-0.03804400
C	14.13848000	-0.47958000	-0.11124800
C	14.54476400	-1.78338500	-0.30336900
C	15.95769300	-1.97365000	-0.29047900
C	16.66137600	-0.79117500	-0.10276600
C	18.08816200	-0.55030700	-0.06950700
C	18.77772600	0.37281800	0.68124200
C	20.18929300	0.39803600	0.43397600
C	20.57074700	-0.52069100	-0.51184100
S	-19.87868000	0.29910100	-0.31121000
S	-15.77817900	-1.25819300	-0.06370000
S	-12.03203300	0.95333200	0.23439500
S	-7.99181500	-0.36148900	-0.93154600
S	-4.17462300	0.76436000	0.85968500
S	-0.16652900	0.60494300	-0.96802000
S	3.66106300	-0.10284900	1.00566000
S	7.68873800	0.99560900	-0.40804000
S	11.45782300	-1.07379000	0.32369700
S	15.54650600	0.55634700	0.11835700
S	19.20618800	-1.40851700	-1.13285000
H	-22.06892400	-0.71878700	0.13536000
H	-20.91396100	-2.82053600	1.21029600
H	-17.44671500	1.96526400	-0.06709200
H	-13.18144100	-2.11112500	-1.33280000
H	-9.54372900	1.82227100	1.52032600
H	-5.40887800	-0.08539900	-2.44571200
H	-1.67411900	0.32296700	2.32395800

H	2.44905600	1.81259300	-1.82844300
H	6.12178800	-1.57317800	1.62505300
H	10.35984800	2.35657800	-0.11653800
H	13.84709400	-2.60027000	-0.45334500
H	18.29591700	0.99450600	1.42825300
H	21.56854900	-0.74810700	-0.85912600
N	21.05605300	1.21948700	1.16834700
H	21.95608400	1.35949800	0.72747600
H	20.64725300	2.11053300	1.42079400
N	16.52185100	-3.24433000	-0.38866200
H	16.05975000	-3.86671700	-1.03745200
H	17.52768400	-3.26590700	-0.48830400
N	13.09242100	2.43292000	-0.49785600
H	13.95711600	2.24347000	-0.99068200
H	12.58606200	3.20391200	-0.91210500
N	8.81086000	-1.83357600	2.11860700
H	8.37643200	-2.74161600	2.20212500
H	9.81951800	-1.87980900	2.08689900
N	5.18381900	1.68739300	-2.17507200
H	6.06185400	1.22617900	-2.38124400
H	4.65718400	1.88692800	-3.01471600
N	1.01303400	0.60495900	2.80765800
H	0.60437800	0.04791200	3.54427600
H	2.02287600	0.58621100	2.80904900
N	-2.66612900	-0.32334600	-2.63038600
H	-1.76831100	-0.75344600	-2.44327500
H	-3.18549400	-0.82676700	-3.33672200
N	-6.87747300	2.47100800	1.59435500
H	-7.27920700	2.62907200	2.50723300
H	-5.86797000	2.50019000	1.59258000
N	-10.42871500	-2.31239500	-1.31579300
H	-9.51912900	-2.42801100	-0.88533400
H	-10.91786700	-3.19244400	-1.40685900
N	-14.80204000	2.54412900	-0.53609200
H	-15.23215400	3.32288900	-0.05869000
H	-13.79431600	2.60037500	-0.56789900
N	-18.14052800	-2.87602200	1.29287800
H	-17.22633100	-2.57905900	1.61345000
H	-18.59290700	-3.48001000	1.96619300
NO_2			
C	-21.30067100	0.16184100	-0.16947800
C	-20.93928700	-1.00053100	0.43627200
C	-19.52592000	-1.10626400	0.58770900
C	-18.79488400	-0.01847900	0.11729400
C	-17.39084500	0.36355700	0.10496400
C	-16.94899100	1.66389900	-0.00489700
C	-15.54495800	1.77483100	-0.11353800
C	-14.86079700	0.56061100	-0.11316600

C	-13.47161700	0.15845900	-0.25525900
C	-13.07473600	-1.09618600	-0.66726800
C	-11.67670900	-1.28805000	-0.61950200
C	-10.95172600	-0.19530000	-0.14572600
C	-9.55085200	0.07161000	0.13344600
C	-9.11564500	1.05247500	0.99956500
C	-7.71197300	1.20259900	1.02022000
C	-7.02037800	0.35831400	0.15222900
C	-5.62753400	0.17783200	-0.22045700
C	-5.22024900	-0.39856900	-1.40514300
C	-3.82198800	-0.57128000	-1.49545900
C	-3.10731900	-0.15593700	-0.37232700
C	-1.71080600	-0.17745600	0.02833600
C	-1.28954000	-0.12135400	1.34023500
C	0.11301000	-0.03853100	1.47807700
C	0.81813000	-0.00282400	0.27543100
C	2.21545900	0.13579000	-0.09797300
C	2.63724000	0.58388900	-1.33211300
C	4.03747100	0.52818400	-1.50285800
C	4.73964000	0.01187200	-0.41421100
C	6.13293400	-0.29075300	-0.13464700
C	6.54352400	-1.19658500	0.82056300
C	7.94419600	-1.23550000	0.99407000
C	8.65779300	-0.34214300	0.19546300
C	10.05628500	0.02512000	0.05357000
C	10.48268700	1.23001400	-0.46351100
C	11.88415500	1.31371000	-0.61399700
C	12.58240700	0.16499600	-0.24207200
C	13.97423900	-0.25020300	-0.26816000
C	14.37978900	-1.56801100	-0.27724100
C	15.78005400	-1.72721100	-0.18522500
C	16.49755100	-0.53701600	-0.07375000
C	17.89659000	-0.18633500	0.11208900
C	18.32182900	0.99418700	0.69317900
C	19.72517900	1.14608000	0.63363700
C	20.37998100	0.11108500	0.01559500
S	-19.92485400	1.13633900	-0.56491500
S	-16.01831200	-0.73425700	0.10671800
S	-12.06207900	1.12082000	0.16652500
S	-8.17088900	-0.69608300	-0.63950600
S	-4.22697200	0.53854200	0.77932100
S	-0.31872700	-0.16050700	-1.04570300
S	3.60502600	-0.33073200	0.87326000
S	7.53285900	0.49783000	-0.84899700
S	11.44262400	-1.00414000	0.38742900
S	15.37955000	0.80378600	-0.17473400
S	19.28347200	-1.10335100	-0.48672200
H	-22.29385500	0.50665600	-0.42030400

H	-21.61844500	-1.77119200	0.77193900
H	-17.58745900	2.53631200	-0.00132300
H	-13.74255200	-1.87459500	-1.00925800
H	-9.75972900	1.65692300	1.62301600
H	-5.88071300	-0.69037700	-2.20980600
H	-1.94292700	-0.14712100	2.20123500
H	1.98599700	0.95781900	-2.10989000
H	5.88377300	-1.83618700	1.39028500
H	9.83396700	2.05358200	-0.72765100
H	13.71697700	-2.41848600	-0.35702300
H	17.67350100	1.72293900	1.16017200
H	21.44680800	0.00935600	-0.11997600
N	20.42164200	2.28856300	1.19665400
O	21.64709300	2.31866200	1.08455600
O	19.73380200	3.14937400	1.74644800
N	16.33768600	-3.06113900	-0.19923500
O	17.55860500	-3.18128200	-0.34516200
O	15.55740900	-4.00400300	-0.07421700
N	12.46259600	2.53212000	-1.13365200
O	13.69028700	2.66486900	-1.07772100
O	11.69523100	3.37418700	-1.59601800
N	8.50708400	-2.15416100	1.95789300
O	9.73279600	-2.31450800	1.96328000
O	7.72955000	-2.73186400	2.71474000
N	4.61101200	0.98270500	-2.74965500
O	5.83883300	1.11215900	-2.81336200
O	3.84020300	1.22342400	-3.67632000
N	0.68581200	0.01115700	2.80453600
O	1.91099900	-0.11229000	2.91515300
O	-0.08296400	0.16504400	3.75104600
N	-3.26249300	-1.15224100	-2.69520600
O	-2.03517600	-1.11918400	-2.84077800
O	-4.04348200	-1.63999200	-3.50920200
N	-7.12434600	2.18970900	1.89790700
O	-5.89735400	2.17772800	2.04954300
O	-7.88282100	2.98230000	2.45191700
N	-11.12757400	-2.55731300	-1.04065700
O	-9.90205200	-2.64461300	-1.17960100
O	-11.91495300	-3.47832200	-1.24593400
N	-14.95180900	3.08783400	-0.22616700
O	-13.72407600	3.18488300	-0.11729500
O	-15.70702300	4.03918900	-0.41701300
N	-18.97113900	-2.28084600	1.21852400
O	-17.74941200	-2.46172600	1.14645300
O	-19.75026200	-3.04415000	1.78515700
OH			
C	-21.09672300	-0.03832200	-0.00229700
C	-20.59935200	-1.03892500	0.78056600

C	-19.17510200	-1.01348800	0.85700600
C	-18.59588000	0.00986800	0.12185300
C	-17.19914100	0.34927600	-0.01706100
C	-16.63146600	1.57882600	-0.25803100
C	-15.21415600	1.54314200	-0.34525600
C	-14.66283600	0.28405300	-0.15335100
C	-13.27925400	-0.11634400	-0.16019200
C	-12.73880000	-1.34732000	-0.45746100
C	-11.32302700	-1.38516400	-0.35841200
C	-10.74319000	-0.17602700	0.00055100
C	-9.35224500	0.15386300	0.17329600
C	-8.78978300	1.13693500	0.95645000
C	-7.37332000	1.18602500	0.87660400
C	-6.81463200	0.22717600	0.04251700
C	-5.42963800	-0.02552000	-0.25917800
C	-4.88401600	-0.60928900	-1.38070100
C	-3.46851700	-0.70799700	-1.33841000
C	-2.89348900	-0.18351800	-0.18888100
C	-1.50435000	-0.09792000	0.18021200
C	-0.94901200	-0.03670500	1.43878000
C	0.46786900	0.05114000	1.43110400
C	1.03374600	0.04020500	0.16350000
C	2.42131100	0.09920500	-0.21709600
C	2.97513300	0.56103200	-1.39013100
C	4.39111100	0.46425300	-1.42672300
C	4.95769800	-0.05819100	-0.27230000
C	6.34536100	-0.27813600	0.04474400
C	6.89726100	-1.19113200	0.91505700
C	8.31353900	-1.12141900	0.98910600
C	8.88181000	-0.16346300	0.16123800
C	10.27032100	0.16951000	-0.03076200
C	10.82303500	1.36652200	-0.42666000
C	12.23973700	1.33469800	-0.52119200
C	12.80679100	0.11487200	-0.18176200
C	14.19619800	-0.26864600	-0.15381100
C	14.75061100	-1.51579500	-0.32979800
C	16.16848500	-1.52602500	-0.23174700
C	16.73147800	-0.28265700	0.00677400
C	18.12441700	0.07543800	0.15082800
C	18.68595100	1.07201800	0.91263900
C	20.10785400	1.13346500	0.80178600
C	20.63674100	0.18278700	-0.03198700
S	-19.84762200	0.95819400	-0.67245900
S	-15.95069800	-0.89517200	0.07734700
S	-12.00376800	1.02054500	0.28787200
S	-8.09626800	-0.71569500	-0.71382400
S	-4.15873900	0.38517800	0.89695200
S	-0.24087900	-0.01289300	-1.05134800

S	3.68451800	-0.50301200	0.86082300
S	7.61077900	0.71740800	-0.68186200
S	11.53520100	-1.04098000	0.20371300
S	15.45939800	0.92197200	0.17177100
S	19.37903000	-0.78400600	-0.74904200
H	-22.13187900	0.17552600	-0.22827900
H	-21.19924800	-1.77415200	1.30197300
H	-17.19565400	2.49815200	-0.35399400
H	-13.32092700	-2.20743500	-0.76430500
H	-9.35666800	1.80154600	1.59659500
H	-5.46207700	-0.94461700	-2.23292900
H	-1.52116600	-0.07486600	2.35752100
H	2.40281300	0.98415900	-2.20641400
H	6.32323400	-1.91607400	1.47887600
H	10.24947200	2.26301000	-0.62746500
H	14.17829700	-2.40876100	-0.54914500
H	18.12138500	1.72781900	1.56404300
H	21.67372100	-0.00599700	-0.27079300
O	20.78946600	2.07217900	1.51614500
H	21.73535400	1.98243300	1.34166100
O	16.83747100	-2.69812100	-0.38451700
H	17.76870800	-2.57952400	-0.13773000
O	12.90457900	2.45395200	-0.90694700
H	13.82708800	2.23738100	-1.11744100
O	8.97751300	-1.96819500	1.81693400
H	9.89713000	-1.67702200	1.92452500
O	5.05669800	0.88492300	-2.53262300
H	5.97519400	0.57215500	-2.50921500
O	1.13449800	0.12195500	2.61152400
H	2.05494800	0.39017500	2.46040600
O	-2.81107700	-1.26946200	-2.38506100
H	-1.89439500	-1.46909300	-2.13653200
O	-6.69994300	2.11572800	1.60132100
H	-5.78232000	2.18138900	1.29215100
O	-10.66980300	-2.54496700	-0.62499100
H	-9.75024100	-2.48870700	-0.31982800
O	-14.53495800	2.69378300	-0.58613800
H	-13.61680800	2.49514500	-0.83001000
O	-18.52921600	-1.93980500	1.61381400
H	-17.60419800	-1.67688400	1.74310600
Ph			
C	21.63992800	0.09868800	-0.54134700
C	21.17140100	1.38026000	-0.53981600
C	19.76336400	1.48350500	-0.28490100
C	19.17562200	0.23957400	-0.10536800
C	17.78612200	-0.14379400	0.11074100
C	17.31483700	-1.15135800	0.92093300
C	15.90869200	-1.38429900	0.83528900

C	15.29211200	-0.52077100	-0.06063100
C	13.88940000	-0.33942500	-0.40420800
C	13.37314800	0.02367000	-1.62826700
C	11.96532800	0.25692400	-1.64299300
C	11.39160000	0.05477500	-0.39437400
C	10.00213500	0.09298700	0.03725300
C	9.52173000	0.49774800	1.26284500
C	8.12063200	0.30519100	1.45385900
C	7.51527200	-0.25454900	0.33605600
C	6.11806200	-0.54304800	0.04781600
C	5.62156700	-1.58170200	-0.70807900
C	4.21347600	-1.54103600	-0.93659100
C	3.61897400	-0.44323400	-0.32769400
C	2.22360400	-0.04341300	-0.22485200
C	1.72954000	1.24138100	-0.17758400
C	0.32566000	1.33880300	0.05786300
C	-0.26881500	0.08967300	0.18352700
C	-1.66324800	-0.29489000	0.34370400
C	-2.15317200	-1.37692100	1.04094200
C	-3.55967200	-1.58500700	0.91967800
C	-4.15997600	-0.62628300	0.11338500
C	-5.55699100	-0.39336300	-0.22043900
C	-6.05595700	0.11494900	-1.39931200
C	-7.46056700	0.36535200	-1.40063100
C	-8.05109200	0.02735000	-0.18962900
C	-9.44391900	0.03303000	0.23098400
C	-9.92825200	0.26657300	1.49909200
C	-11.33403700	0.07595600	1.65179300
C	-11.94021100	-0.30338700	0.46099800
C	-13.33939100	-0.52338900	0.12614400
C	-13.84396500	-1.42240300	-0.78679400
C	-15.25081400	-1.32821900	-1.01139400
C	-15.83393500	-0.33421700	-0.23757200
C	-17.22718400	0.06084100	-0.07417300
C	-17.72631300	1.33654800	0.05076800
C	-19.14139000	1.40023900	0.26816300
C	-19.69444600	0.14048400	0.31287100
S	20.36993500	-1.03997600	-0.25537800
S	16.46274500	0.56460400	-0.79065000
S	12.60423200	-0.41594900	0.78398200
S	8.69025800	-0.54036100	-0.93597800
S	4.81294800	0.52741700	0.51702000
S	0.92061600	-1.19010900	0.01335600
S	-2.97296800	0.51845400	-0.48814900
S	-6.85655500	-0.59288100	0.93763000
S	-10.75949000	-0.42361700	-0.83229700
S	-14.63532400	0.47479900	0.75452300
S	-18.52238900	-1.11186000	0.10399300

H	22.65719200	-0.24131100	-0.67683700
H	21.80576200	2.24855600	-0.67505200
H	17.96657700	-1.74110900	1.55567200
H	13.99376900	0.14901800	-2.50841200
H	10.16431600	0.90779300	2.03384800
H	6.25665400	-2.35724200	-1.12129000
H	2.36409300	2.11392400	-0.28475700
H	-1.51416700	-2.03637200	1.61741000
H	-5.42446100	0.33411200	-2.25295200
H	-9.28521800	0.54724100	2.32569200
H	-13.21477200	-2.12879100	-1.31651400
H	-17.09935500	2.21373600	-0.05954300
H	-20.72995900	-0.11937000	0.48310500
C	-19.90475200	2.65744500	0.41601400
C	-19.31465000	3.79065300	1.00086100
C	-21.23799200	2.75231600	-0.01779700
C	-20.03392600	4.97516900	1.14983400
H	-18.29200200	3.73535400	1.36166400
C	-21.95867300	3.93443300	0.13747200
H	-21.70264200	1.89824700	-0.50156500
C	-21.35980300	5.05253300	0.72100600
H	-19.55881800	5.83739200	1.60894800
H	-22.98700800	3.98599900	-0.20877500
H	-21.92006900	5.97546400	0.83739000
C	-15.94397800	-2.19045200	-1.99914300
C	-16.83348700	-1.65010800	-2.94266500
C	-15.67761600	-3.56891900	-2.04285800
C	-17.44349700	-2.46589300	-3.89327300
H	-17.03415200	-0.58369700	-2.93379900
C	-16.28853400	-4.38410800	-2.99480200
H	-14.99785600	-4.00307300	-1.31537000
C	-17.17448000	-3.83572300	-3.92314100
H	-18.12485600	-2.02896600	-4.61759700
H	-16.07393700	-5.44883400	-3.00871600
H	-17.64964500	-4.46988000	-4.66580400
C	-12.00432300	0.24131700	2.96482100
C	-12.86222500	-0.74656000	3.47577600
C	-11.74434100	1.37536900	3.75182700
C	-13.45062700	-0.59779100	4.73001300
H	-13.05582500	-1.63787600	2.88774400
C	-12.33313600	1.52270200	5.00709900
H	-11.08720500	2.15031400	3.36783400
C	-13.18961000	0.53730400	5.49999900
H	-14.10916700	-1.37362900	5.10947700
H	-12.12470400	2.40962600	5.59853300
H	-13.64824000	0.65128400	6.47780700
C	-8.14504500	0.95583600	-2.57699300
C	-8.98368300	2.07502700	-2.44666900

C	-7.91806300	0.43398200	-3.86106600
C	-9.58491000	2.64672000	-3.56605200
H	-9.15184300	2.50116600	-1.46283700
C	-8.52015900	1.00711700	-4.98046200
H	-7.27623400	-0.43469800	-3.97625800
C	-9.35681900	2.11474200	-4.83665200
H	-10.22785400	3.51383500	-3.44626200
H	-8.33778700	0.58553700	-5.96474700
H	-9.82586300	2.56185700	-5.70814000
C	-4.23705200	-2.73189900	1.57266400
C	-5.09759400	-3.57721600	0.85305000
C	-3.98287200	-3.02596200	2.92244600
C	-5.69429800	-4.67533200	1.46905000
H	-5.28759100	-3.37373700	-0.19585700
C	-4.58007300	-4.12552600	3.53751500
H	-3.32422800	-2.37770300	3.49319300
C	-5.43919600	-4.95320200	2.81335700
H	-6.35485300	-5.31919300	0.89571200
H	-4.37647500	-4.33305600	4.58407900
H	-5.90463600	-5.80978100	3.29195300
C	-0.35434300	2.65131700	0.18172000
C	-1.20377800	2.93405400	1.26403700
C	-0.11393400	3.66228800	-0.76327400
C	-1.80295300	4.18572100	1.38930800
H	-1.38300000	2.16926400	2.01283600
C	-0.71354300	4.91462900	-0.63638100
H	0.53602000	3.45655400	-1.60891000
C	-1.56144700	5.18036900	0.43968800
H	-2.45472300	4.38657100	2.23457800
H	-0.52033700	5.68154600	-1.38091300
H	-2.02868100	6.15565500	0.53921900
C	3.52934000	-2.56033000	-1.76944900
C	2.66441700	-2.18953000	-2.81219800
C	3.78128700	-3.92645000	-1.56137600
C	2.06156500	-3.15672100	-3.61369600
H	2.47626500	-1.13694400	-2.99748700
C	3.17777500	-4.89332200	-2.36445800
H	4.44338500	-4.22847900	-0.75501500
C	2.31460500	-4.51191700	-3.39255000
H	1.39803000	-2.85072700	-4.41725200
H	3.37976000	-5.94521100	-2.18405500
H	1.84447200	-5.26460600	-4.01851800
C	7.45293500	0.64832800	2.73318900
C	6.60924800	-0.26687700	3.38409500
C	7.69819300	1.88764800	3.34711900
C	6.02013700	0.05388500	4.60527700
H	6.42636600	-1.23595600	2.93122200
C	7.10851700	2.20716400	4.56945800

H	8.34346900	2.60786700	2.85244200
C	6.26607800	1.29196300	5.20211100
H	5.37256500	-0.66758400	5.09490900
H	7.30487000	3.17326500	5.02542000
H	5.80646900	1.54029500	6.15421300
C	11.26097500	0.70052700	-2.87086100
C	10.39233500	1.80425300	-2.85496000
C	11.49615600	0.05023700	-4.09345100
C	9.77042200	2.23624300	-4.02449500
H	10.21676200	2.32892600	-1.92139600
C	10.87343800	0.48374000	-5.26319000
H	12.16045000	-0.80870300	-4.12001000
C	10.00715700	1.57740900	-5.23253700
H	9.10447900	3.09366300	-3.99328200
H	11.06285600	-0.03592200	-6.19799500
H	9.52205400	1.91570400	-6.14338300
C	15.24950200	-2.46350300	1.61056600
C	14.38843900	-3.38547300	0.99275000
C	15.52312800	-2.61563600	2.97990000
C	13.81071700	-4.41999600	1.72574900
H	14.18313400	-3.29207000	-0.06877300
C	14.94474500	-3.65155800	3.71213400
H	16.18219800	-1.90667700	3.47268800
C	14.08539600	-4.55675900	3.08786700
H	13.14961000	-5.12491000	1.23013300
H	15.16325700	-3.74893200	4.77165400
H	13.63479000	-5.36406400	3.65760100
C	19.08186700	2.79663500	-0.18406600
C	18.22262800	3.09537000	0.88632600
C	19.32843700	3.79352900	-1.14259300
C	17.62104900	4.34809000	0.98717200
H	18.03721700	2.34219200	1.64521600
C	18.72628200	5.04682000	-1.04030200
H	19.98403800	3.57542100	-1.98075000
C	17.86929500	5.32840700	0.02452100
H	16.96182600	4.56097700	1.82368200
H	18.92424300	5.80204400	-1.79548500
H	17.40004300	6.30447500	0.10495300
SH			
C	-21.28717900	-0.16914300	-0.11673700
C	-20.83696500	-0.68794000	1.06161800
C	-19.41548200	-0.62094600	1.19786200
C	-18.78534200	-0.04452100	0.10488400
C	-17.38911300	0.22207200	-0.17847000
C	-16.89199000	1.11285000	-1.10595800
C	-15.47834400	1.10211700	-1.23280100
C	-14.85526400	0.19570500	-0.38539000
C	-13.46255700	-0.12705900	-0.17003200

C	-12.96751100	-1.22183000	0.51015600
C	-11.55527900	-1.24966500	0.63186800
C	-10.92803500	-0.16606700	0.02965600
C	-9.53456300	0.19474100	-0.09791500
C	-9.03448100	1.41813000	-0.49893700
C	-7.62162200	1.47180500	-0.59981400
C	-6.99858900	0.27602300	-0.26464900
C	-5.60642200	-0.10753200	-0.21468100
C	-5.10985400	-1.39320900	-0.12691900
C	-3.69879800	-1.47299000	-0.01922800
C	-3.07260200	-0.23245600	-0.03255200
C	-1.68043700	0.14736400	0.03786800
C	-1.18310600	1.41722100	0.25635000
C	0.23032800	1.51161900	0.21032000
C	0.85725200	0.29648700	-0.03819400
C	2.25115900	-0.06612400	-0.15220400
C	2.75369400	-1.26120000	-0.62856300
C	4.16508000	-1.37330200	-0.56516100
C	4.78530700	-0.24595900	-0.04023400
C	6.17600400	0.07072200	0.19140300
C	6.66701700	1.12217300	0.94054900
C	8.08035800	1.23336200	0.94325500
C	8.71173100	0.25086600	0.19140700
C	10.10778900	-0.01829800	-0.07015800
C	10.61659100	-0.82169100	-1.07081800
C	12.02688700	-0.97356900	-1.04373900
C	12.63645100	-0.27402100	-0.01097900
C	14.02330000	-0.13818000	0.37854700
C	14.49242200	0.30521600	1.59823900
C	15.90677600	0.41676900	1.68010100
C	16.55064400	0.04954900	0.50997100
C	17.95531300	0.00672800	0.14649300
C	18.49087600	0.23577700	-1.10193200
C	19.90262400	0.03335000	-1.16842700
C	20.43872700	-0.34710300	0.03310900
S	-19.98754300	0.41835300	-1.09589500
S	-16.06400900	-0.65908300	0.56827600
S	-12.13247600	0.90298500	-0.68344000
S	-8.20769600	-0.92894000	0.16865200
S	-4.27773000	1.04475700	-0.16756000
S	-0.34956500	-0.97169600	-0.22963400
S	3.57443400	0.95881600	0.38927600
S	7.51167500	-0.81524800	-0.53269700
S	11.42030200	0.57464300	0.93907000
S	15.37409600	-0.43952800	-0.70441900
S	19.22417500	-0.48318800	1.26546500
H	-22.30710500	-0.08340900	-0.46431000
H	-21.48201900	-1.09559700	1.83030600

H	-17.52106200	1.76771700	-1.69685300
H	-13.59759600	-1.99954800	0.92480500
H	-9.66187000	2.27219900	-0.72417500
H	-5.73817200	-2.27575300	-0.13623600
H	-1.81232900	2.27859900	0.44578300
H	2.12932100	-2.05688700	-1.01691600
H	6.03230300	1.81452000	1.48068500
H	9.99642200	-1.30408100	-1.81698200
H	13.83854000	0.55303100	2.42649700
H	17.89236500	0.56113300	-1.94504000
H	21.47382900	-0.53962500	0.27735600
S	20.77815800	0.30106800	-2.69685800
H	21.99967000	-0.03223300	-2.23747000
S	16.63078600	0.97752900	3.20775300
H	17.82305400	1.34978900	2.70112500
S	12.82272900	-1.93924000	-2.31661200
H	13.67888100	-2.61273400	-1.52057900
S	8.85845200	2.51235700	1.91712000
H	9.70929900	2.96302100	0.97158200
S	4.96449900	-2.83843400	-1.20342000
H	5.68477000	-3.14413400	-0.10323100
S	1.02600900	3.07762900	0.54041100
H	1.74009300	3.16228400	-0.60235200
S	-2.90416100	-3.07291900	0.03594400
H	-2.21043300	-2.89078700	1.17973400
S	-6.82546000	3.00779300	-1.04677300
H	-6.07031000	2.53085700	-2.05885600
S	-10.76421500	-2.63795700	1.43183900
H	-10.03517900	-1.93848100	2.32716600
S	-14.68211900	2.24953800	-2.34738000
H	-13.95653700	1.35006300	-3.04522000
S	-18.63982500	-1.29165800	2.65914100
H	-17.81095000	-0.25820500	2.91605700
SiH_3			
C	-21.18434300	0.39219500	-0.00373100
C	-20.69303300	1.12212100	1.04124500
C	-19.26800500	1.03703400	1.20211400
C	-18.69878000	0.21519400	0.23872800
C	-17.31397100	-0.17171400	0.01681900
C	-16.83454700	-1.37914800	-0.44360300
C	-15.41685200	-1.45023000	-0.60746100
C	-14.80338300	-0.25287500	-0.25794000
C	-13.40428200	0.13728900	-0.26593200
C	-12.87636900	1.38804500	-0.50967700
C	-11.45530900	1.48152400	-0.40634000
C	-10.88720000	0.25697900	-0.07289700
C	-9.50115600	-0.12164000	0.13756800
C	-9.00582800	-1.08265400	0.99439400

C	-7.58996700	-1.26340300	0.95548700
C	-6.99243400	-0.40833000	0.03623400
C	-5.59976100	-0.23606800	-0.33748700
C	-5.09155900	0.11928600	-1.56962300
C	-3.67194100	0.26139200	-1.62649000
C	-3.08461400	0.00421500	-0.39234800
C	-1.69404000	0.03395800	0.02420900
C	-1.19056400	0.35387600	1.26821500
C	0.22832000	0.26045900	1.39499500
C	0.82052400	-0.14392000	0.20354500
C	2.21394300	-0.36874900	-0.13734500
C	2.72998900	-1.27446500	-1.04094200
C	4.14805900	-1.23477100	-1.20250900
C	4.72603000	-0.26410700	-0.39166800
C	6.11236200	0.13410000	-0.22433100
C	6.60160100	1.38892700	0.07401900
C	8.01916700	1.47202200	0.22228300
C	8.62471300	0.23515500	0.02875700
C	10.02137400	-0.15854100	0.08237800
C	10.54412100	-1.37446000	0.47124200
C	11.96478400	-1.48502300	0.38032600
C	12.53772900	-0.31019500	-0.09387400
C	13.92516500	0.03768900	-0.34629000
C	14.42176700	0.89122300	-1.30926500
C	15.83776600	1.07727500	-1.28687700
C	16.43180000	0.33822700	-0.27173500
C	17.82629100	0.21766900	0.12592500
C	18.33534900	0.08388600	1.39889500
C	19.76120700	-0.04380200	1.45948500
C	20.31417900	-0.00459400	0.20024600
S	-19.91846800	-0.42309900	-0.85348300
S	-15.98404300	0.93766900	0.26324400
S	-12.11502700	-0.98487900	0.10998200
S	-8.18636300	0.59957300	-0.76458800
S	-4.29271000	-0.40752900	0.81353500
S	-0.38266700	-0.40492800	-1.04837100
S	3.51142500	0.58136200	0.55309600
S	7.43588300	-1.00596900	-0.33070900
S	11.31535200	0.90679600	-0.42099800
S	15.23867600	-0.57086600	0.63765600
S	19.12903600	0.16576900	-1.05011300
H	-22.21190400	0.28448500	-0.32247100
H	-21.32966400	1.70118400	1.70100400
H	-17.49658800	-2.21150200	-0.65617400
H	-13.50548600	2.23227200	-0.77017700
H	-9.65451500	-1.64865000	1.65415100
H	-5.73399200	0.26923900	-2.43054300
H	-1.83578600	0.66392100	2.08306000

H	2.09465200	-1.96936800	-1.57937200
H	5.94685400	2.24744100	0.17723800
H	9.91204000	-2.18002700	0.82896800
H	13.77454700	1.37533400	-2.03264200
H	17.69566100	0.10477500	2.27500100
H	21.35998900	-0.06539300	-0.07034400
Si	-18.41283200	1.89162200	2.64224300
H	-17.49328100	0.97072800	3.35603900
H	-17.64373900	3.09145900	2.21933200
H	-19.48175400	2.33580200	3.57537200
Si	-14.61518400	-3.00387100	-1.30451000
H	-13.68598200	-2.68236900	-2.41607100
H	-13.87120900	-3.77170500	-0.27222300
H	-15.71646600	-3.86070600	-1.81633000
Si	-10.59349500	3.14018700	-0.63168900
H	-9.65917800	3.42149400	0.48629400
H	-9.84114700	3.21972300	-1.91068300
H	-11.65987900	4.17499000	-0.65439900
Si	-6.77038400	-2.59252300	2.00744800
H	-5.85661100	-3.43593800	1.19783400
H	-6.00555700	-2.02222000	3.14676700
H	-7.86360900	-3.43610700	2.55653600
Si	-2.83754700	0.81874500	-3.21977300
H	-1.92243900	1.96238300	-2.98154100
H	-2.07153800	-0.27545900	-3.87057800
H	-3.92299500	1.24086000	-4.14271700
Si	1.05813100	0.58792100	3.05321100
H	1.99203300	-0.50661400	3.41595400
H	1.80272800	1.87370400	3.07021400
H	-0.02776100	0.66527800	4.06472500
Si	4.99465400	-2.35186400	-2.45977400
H	5.90764100	-1.58064800	-3.33920700
H	5.76670200	-3.44645900	-1.81655600
H	3.91717000	-2.96240100	-3.28112400
Si	8.82972600	3.10071900	0.70794400
H	9.74879900	2.92610400	1.85978400
H	9.58509200	3.71451700	-0.41467500
H	7.73088300	4.02509500	1.09062300
Si	12.82202600	-3.10817300	0.79942700
H	13.75580500	-3.52057000	-0.27751500
H	13.57362000	-3.03761900	2.07929300
H	11.75258700	-4.12993900	0.94299900
Si	16.66958600	2.27323200	-2.47935900
H	17.60238600	3.18149700	-1.76749500
H	17.41626700	1.57368800	-3.55681900
H	15.58683700	3.07328600	-3.10912300
Si	20.78424800	-0.22293000	3.01919900
H	20.40559400	-1.44870800	3.77097700

H	22.21600500	-0.31192000	2.63307800
H	20.59488300	0.94347000	3.92192300
SMe			
C	21.49496600	0.00157100	0.35388300
C	21.06090900	-0.83507000	-0.63086900
C	19.63651100	-0.85776700	-0.76856500
C	18.98698500	-0.02365800	0.13265900
C	17.58216800	0.27033600	0.34224100
C	17.06288200	1.31158800	1.08177900
C	15.64483600	1.33416000	1.16127300
C	15.04539200	0.29179100	0.46424600
C	13.65675500	-0.06855000	0.27616500
C	13.17861700	-1.26334800	-0.22252300
C	11.76617600	-1.33322200	-0.34332400
C	11.12586100	-0.17165300	0.07515300
C	9.72691100	0.19209900	0.12294400
C	9.21311700	1.45604600	0.33388400
C	7.79602100	1.52285600	0.36775000
C	7.18770600	0.28722100	0.17304900
C	5.79643500	-0.10486700	0.13033900
C	5.30579100	-1.39520400	0.15724600
C	3.89364400	-1.49569800	0.06282000
C	3.26500200	-0.25884400	-0.03488200
C	1.87033700	0.10532600	-0.15047100
C	1.37067200	1.34763500	-0.48632700
C	-0.04547700	1.43878300	-0.48242800
C	-0.66756700	0.24187500	-0.14265000
C	-2.06281600	-0.11423700	-0.01038500
C	-2.56490100	-1.27343800	0.54699700
C	-3.97935000	-1.38162500	0.51945700
C	-4.59818500	-0.28210500	-0.06602900
C	-5.99163600	0.02583300	-0.30034600
C	-6.48714200	1.03111100	-1.10657800
C	-7.90217900	1.13828800	-1.11993000
C	-8.52736300	0.19476900	-0.31152900
C	-9.92295300	-0.05604900	-0.02624800
C	-10.42416400	-0.85198200	0.98414800
C	-11.83926900	-0.95776000	1.01028700
C	-12.45843700	-0.22565400	0.00301100
C	-13.85272400	-0.04850900	-0.34034100
C	-14.34885500	0.49243800	-1.50894400
C	-15.76476700	0.58785700	-1.56460200
C	-16.38700800	0.10873900	-0.41843200
C	-17.78572900	0.01928000	-0.04322900
C	-18.27553500	-0.18272000	1.23050300
C	-19.69781200	-0.28268000	1.29789000
C	-20.28388100	-0.15171600	0.06471600
S	20.17108500	0.78133700	1.15585000

S	16.27351100	-0.71353300	-0.29986500
S	12.31019700	1.00876900	0.62928700
S	8.40893900	-0.96109700	-0.05786700
S	4.46049700	1.03460100	0.00031700
S	0.53983900	-1.00386500	0.16454100
S	-3.39033800	0.89322100	-0.57858400
S	-7.32536100	-0.83583400	0.46016300
S	-11.25125700	0.60453300	-0.97451700
S	-15.18585500	-0.47524000	0.72691500
S	-19.11362100	0.09741900	-1.19565000
H	22.50932700	0.20702300	0.66592600
H	21.71586200	-1.43142700	-1.25432100
H	17.67562500	2.06611600	1.56080300
H	13.81901300	-2.09499800	-0.49157600
H	9.83103000	2.33838000	0.45136900
H	5.93687500	-2.27057200	0.25526200
H	1.99895000	2.19084200	-0.74797800
H	-1.94037900	-2.04220200	0.98640100
H	-5.85713100	1.68540400	-1.69744300
H	-9.79871700	-1.35136200	1.71460900
H	-13.72000200	0.81071400	-2.33198700
H	-17.63756700	-0.24535800	2.10487600
H	-21.33355600	-0.16914300	-0.18830000
S	-20.49699100	-0.53309400	2.86363700
S	-16.59308400	1.21916400	-3.01382300
S	-12.67398300	-1.90633500	2.26984600
S	-8.73148300	2.35023000	-2.13313300
S	-4.81592800	-2.78894400	1.22820100
S	-0.87940600	2.95147200	-0.92844200
S	3.07038300	-3.07749100	0.12007100
S	6.94516200	3.07532800	0.58706100
S	10.95760500	-2.81123900	-0.93001300
S	14.78553600	2.62627400	2.04137500
S	18.83250700	-1.90311200	-1.96885200
C	-22.25081400	-0.60367300	2.37520100
H	-22.43516900	-1.43504200	1.69061300
H	-22.81568100	-0.76697300	3.29475300
H	-22.57242100	0.33688800	1.92163400
C	-16.91363300	2.95662200	-2.51146800
H	-17.59903700	2.99532600	-1.66300900
H	-15.97946600	3.46614500	-2.26897500
H	-17.37813300	3.44322300	-3.37228600
C	-12.98929500	-3.48368100	1.38198300
H	-13.67435900	-3.32981600	0.54642300
H	-12.05316500	-3.92140300	1.03126600
H	-13.45305800	-4.15510000	2.10831900
C	-9.02647600	3.69269600	-0.91395300
H	-9.70138800	3.35890800	-0.12393000

H	-8.08371100	4.03924100	-0.48723900
H	-9.49562700	4.50979300	-1.46688400
C	-1.16284000	3.70180000	0.72480600
H	-1.83365000	3.08281700	1.32312900
H	-0.21622800	3.85049800	1.24706000
H	-1.63254600	4.67139900	0.54393300
C	6.62566500	3.05451900	2.39646900
H	5.95304200	2.23760900	2.66367700
H	7.56155000	2.97298400	2.95186500
H	6.14682300	4.00758500	2.63282000
C	14.46768000	1.80805500	3.65565300
H	13.79558800	0.95672400	3.53436900
H	15.40426800	1.49035400	4.11689800
H	13.98862100	2.55800600	4.28931900
C	18.57647700	-0.72682300	-3.35707100
H	17.89529200	0.07469100	-3.06634000
H	19.52889600	-0.31275900	-3.69240600
H	18.12750300	-1.30468100	-4.16825100
C	10.72214600	-2.40757500	-2.70705200
H	10.04159500	-1.56286400	-2.82732300
H	11.68055300	-2.19439400	-3.18342800
H	10.27980600	-3.29495700	-3.16568000
C	2.84882900	-3.42823900	-1.66984100
H	2.18245400	-2.69668900	-2.13020800
H	3.81263600	-3.43939200	-2.18161900
H	2.39425800	-4.41962300	-1.73303800
C	-5.09235300	-3.82796400	-0.26169900
H	-5.76066100	-3.32902700	-0.96566500
H	-4.14377100	-4.06956000	-0.74418800
H	-5.56338700	-4.74824700	0.09143700
*t*Bu			
C	-20.93728100	0.59005700	0.38469400
C	-20.50258500	1.63761300	-0.37494200
C	-19.11570700	1.57264400	-0.74720700
C	-18.51631600	0.43325400	-0.22992600
C	-17.15143300	-0.10516600	-0.30655400
C	-16.75027500	-1.26120900	-0.92991900
C	-15.37112600	-1.61412300	-0.76131200
C	-14.71552100	-0.68408800	0.03235600
C	-13.32624600	-0.56116200	0.49514100
C	-12.86989800	-0.67090400	1.78575900
C	-11.47745900	-0.39180300	1.98003400
C	-10.86956800	-0.04703000	0.78125600
C	-9.49229000	0.32144700	0.42527700
C	-9.05260800	1.54412100	-0.01985700
C	-7.67168700	1.60943100	-0.39809700
C	-7.05599500	0.37601800	-0.23946600
C	-5.68115500	-0.09477400	-0.45765700

C	-5.25437700	-1.00763800	-1.39111900
C	-3.87156300	-1.37735300	-1.31382500
C	-3.24031600	-0.71848200	-0.26816500
C	-1.86116000	-0.72179500	0.23864500
C	-1.43508400	-1.16584300	1.46685200
C	-0.05241000	-0.93889000	1.76748500
C	0.58182600	-0.28771200	0.71815000
C	1.95868600	0.17411600	0.49969700
C	2.36476700	1.47021900	0.29109400
C	3.75087400	1.64720700	-0.02622700
C	4.41017300	0.42639200	-0.06163500
C	5.80409200	0.03901200	-0.31389300
C	6.26569200	-0.75513700	-1.33564100
C	7.65871300	-1.08816200	-1.28630700
C	8.26370900	-0.52263700	-0.17269200
C	9.63973500	-0.53564100	0.34087300
C	10.07117300	-1.07446300	1.52869300
C	11.44970000	-0.85047700	1.85048300
C	12.07354600	-0.10255200	0.86221500
C	13.44850700	0.38580200	0.68870600
C	13.86740300	1.69395800	0.68585800
C	15.25116700	1.90376900	0.37730100
C	15.89156000	0.69832000	0.12782000
C	17.27686900	0.35057200	-0.21862700
C	17.73600800	-0.15549200	-1.40910100
C	19.13189800	-0.49030600	-1.42414600
C	19.70843000	-0.24095500	-0.20515600
S	-19.66411100	-0.53061600	0.69015100
S	-15.80584200	0.58567800	0.56333000
S	-12.01834300	-0.06152700	-0.54644200
S	-8.18438000	-0.83113500	0.35386400
S	-4.35703700	0.32231200	0.59894000
S	-0.53344500	0.03988000	-0.59833100
S	3.31258700	-0.90323700	0.27343400
S	7.10956500	0.38511800	0.79085700
S	10.95451200	0.31985900	-0.42326900
S	14.78273200	-0.65568700	0.26537000
S	18.58358100	0.38641200	0.95486900
H	-21.93239800	0.41502200	0.76969700
H	-21.16387600	2.43851700	-0.67767600
H	-17.45165000	-1.85008400	-1.50602200
H	-13.54029300	-0.95033000	2.58765800
H	-9.72656100	2.38886500	-0.07086200
H	-5.93919800	-1.40843100	-2.12665500
H	-2.12241300	-1.64880100	2.14862900
H	1.66054600	2.28824600	0.36477900
H	5.59758100	-1.09611400	-2.11533600
H	9.39221100	-1.62826400	2.16344700

H	13.17625900	2.49709100	0.90450400
H	17.07914600	-0.27158200	-2.26381300
H	20.73878700	-0.38263800	0.08688400
C	19.84999600	-1.04131600	-2.65504600
C	19.74477300	-0.01762700	-3.80923900
C	21.33755300	-1.30732900	-2.36241800
C	19.18231300	-2.36775800	-3.08554400
H	18.70253400	0.18571600	-4.07389800
H	20.21201700	0.93316900	-3.53370900
H	20.24851600	-0.39827500	-4.70486800
H	21.46430700	-2.04293600	-1.56126200
H	21.83140400	-1.70110700	-3.25648200
H	21.86024800	-0.39013200	-2.07133000
H	19.67786100	-2.77407600	-3.97440800
H	19.24551600	-3.11508700	-2.28827200
H	18.12470000	-2.22694400	-3.32877500
C	15.88258600	3.30799900	0.34740500
C	16.70385800	3.53919200	1.63849700
C	16.78979200	3.48856200	-0.88947200
C	14.78887600	4.39716800	0.28166800
H	16.07958200	3.40650700	2.52828800
H	17.54759500	2.84932000	1.71022400
H	17.10281900	4.56009500	1.65466900
H	16.22051200	3.34759100	-1.81423400
H	17.20332900	4.50301400	-0.89904300
H	17.62422600	2.78600800	-0.89907100
H	15.26123000	5.38052000	0.19189800
H	14.13172600	4.26097800	-0.58330400
H	14.16993500	4.41710600	1.18401800
C	12.09152700	-1.39613700	3.13963100
C	12.92401200	-2.65925600	2.81481200
C	12.99048300	-0.33507600	3.81178600
C	11.00401100	-1.79360600	4.16250900
H	12.30565100	-3.42208400	2.33033000
H	13.76221300	-2.43114500	2.15247000
H	13.33252800	-3.08996900	3.73618200
H	12.41479700	0.56256400	4.06044500
H	13.40535200	-0.73881600	4.74183000
H	13.82488000	-0.03340600	3.17733900
H	11.48086600	-2.10785300	5.09629300
H	10.33786400	-0.95572400	4.39236200
H	10.39342500	-2.63204700	3.81345100
C	8.33059100	-1.96623900	-2.35839200
C	9.12086000	-1.07454600	-3.34570100
C	9.27607800	-3.00786300	-1.72040900
C	7.26897000	-2.74368400	-3.16864000
H	8.46839700	-0.32359200	-3.80315600
H	9.94294600	-0.55295500	-2.85059400

H	9.54746600	-1.68662400	-4.14878500
H	8.72999400	-3.65896400	-1.02989200
H	9.71309600	-3.63660100	-2.50372500
H	10.09522900	-2.54531400	-1.16828400
H	7.77035300	-3.41629200	-3.87178800
H	6.62950100	-3.35157200	-2.52056900
H	6.62925900	-2.07913600	-3.75731600
C	4.35942800	3.03725100	-0.28914400
C	5.11389100	3.52622600	0.97010200
C	5.31912000	3.01042600	-1.49953800
C	3.24973300	4.06513500	-0.60506400
H	4.44970100	3.54801700	1.84035800
H	5.96387700	2.88274400	1.20824000
H	5.49645100	4.54105000	0.81107600
H	4.79652200	2.68114100	-2.40364500
H	5.70998700	4.01728200	-1.68271900
H	6.16979700	2.34614100	-1.34315300
H	3.70756400	5.02446700	-0.86597500
H	2.63110700	3.74772700	-1.45070900
H	2.59380200	4.24285200	0.25266000
C	0.57784300	-1.37465600	3.10351100
C	1.40256700	-2.66795600	2.89894900
C	1.47812500	-0.26255700	3.68522000
C	-0.51795900	-1.67592200	4.15024100
H	0.78310900	-3.46378500	2.47246800
H	2.25260600	-2.50587800	2.23271900
H	1.79414400	-3.02210100	3.85958200
H	0.90237100	0.65227000	3.85975800
H	1.89498000	-0.58804300	4.64458700
H	2.31085400	-0.01476300	3.02583800
H	-0.04727400	-1.91431100	5.10923900
H	-1.17837500	-0.81667400	4.30536000
H	-1.13353600	-2.53617100	3.86938400
C	-3.23259400	-2.38129800	-2.29128300
C	-2.40646800	-1.62196600	-3.35644400
C	-2.32801300	-3.38811100	-1.54690000
C	-4.32131900	-3.19420900	-3.02694000
H	-3.02780300	-0.89423200	-3.88866200
H	-1.56527800	-1.08743600	-2.90959100
H	-2.00248000	-2.32548400	-4.09357600
H	-2.89981000	-3.94685100	-0.79862900
H	-1.91105100	-4.10778900	-2.25986000
H	-1.49509100	-2.90132900	-1.03783800
H	-3.84502000	-3.94759300	-3.66229700
H	-4.98143300	-3.71592400	-2.32645800
H	-4.93786800	-2.56553300	-3.67669300
C	-7.01805600	2.91038800	-0.89985300
C	-6.19596100	3.55479200	0.24206100

C	-6.10670400	2.64900500	-2.11884700
C	-8.09544300	3.92731900	-1.33860000
H	-6.82501900	3.74433800	1.11809900
H	-5.36529900	2.91718700	0.55289500
H	-5.77774400	4.51284700	-0.08781200
H	-6.67710500	2.21252200	-2.94542800
H	-5.67714800	3.59460900	-2.46713000
H	-5.28286200	1.97309000	-1.88582900
H	-7.60909500	4.81377800	-1.75814600
H	-8.75619300	3.51232000	-2.10656200
H	-8.71230100	4.26401200	-0.49972300
C	-10.80749900	-0.48463900	3.36355000
C	-10.01070500	-1.80676200	3.46940500
C	-9.86759900	0.71457500	3.61648500
C	-11.87079900	-0.48152000	4.48463300
H	-10.65940500	-2.67001900	3.28781100
H	-9.18937400	-1.84211500	2.75009900
H	-9.58252300	-1.91047000	4.47310400
H	-10.41663400	1.66007200	3.55652400
H	-9.43501800	0.63835000	4.62005600
H	-9.04528900	0.75771700	2.90109600
H	-11.37144100	-0.47442200	5.45868300
H	-12.51426700	0.40265000	4.43151000
H	-12.50668200	-1.37167700	4.45422700
C	-14.76008900	-2.87000500	-1.40921000
C	-13.96886400	-2.46928000	-2.67714900
C	-13.82862500	-3.61594800	-0.42826100
C	-15.86970700	-3.85850400	-1.83225600
H	-14.60967700	-1.93615900	-3.38719800
H	-13.12064600	-1.82450800	-2.43553800
H	-13.57901700	-3.36310200	-3.17786300
H	-14.37040100	-3.91239900	0.47595200
H	-13.44404900	-4.52440100	-0.90471200
H	-12.97324000	-3.01136700	-0.12403400
H	-15.41243600	-4.77468100	-2.21911800
H	-16.50992900	-4.13432500	-0.98805700
H	-16.50433900	-3.45385000	-2.62655800
C	-18.44585800	2.65691600	-1.61153100
C	-17.61572700	3.60332600	-0.71156400
C	-17.53712000	2.03591100	-2.69528500
C	-19.51070800	3.50881100	-2.33829200
H	-18.24263300	4.05320700	0.06529600
H	-16.79420100	3.07649100	-0.22100800
H	-17.18469500	4.41381500	-1.31078800
H	-18.10862700	1.36832900	-3.34850400
H	-17.10807300	2.83031700	-3.31575100
H	-16.71229900	1.46311900	-2.26946400
H	-19.01307600	4.22493300	-2.99996300

H	-20.17466000	2.89061800	-2.95129000
H	-20.12537200	4.08665900	-1.64117100

SPRINGS XYZ

BF_2			
C	-15.34053000	-0.52510200	-3.52776100
C	-14.94060000	-1.57839700	-4.29385200
C	-13.88846100	-2.36550100	-3.71046700
C	-13.51270600	-1.87338500	-2.46051100
S	-14.45904700	-0.46190900	-2.03721100
C	-12.51955900	-2.36394900	-1.51648000
C	-12.16670300	-3.65634300	-1.21461700
C	-11.14999900	-3.79126400	-0.21830800
C	-10.73230100	-2.55067000	0.26850200
S	-11.59227700	-1.25034800	-0.52339800
C	-9.75466200	-2.22340000	1.29276400
C	-9.43887400	-2.90050200	2.44633800
C	-8.43115100	-2.28797500	3.25359000
C	-7.97953100	-1.08754600	2.69872500
S	-8.79995200	-0.75146100	1.19181400
C	-6.99292800	-0.13807800	3.18418700
C	-6.68255200	0.21484100	4.47600800
C	-5.66020700	1.20440600	4.60594100
C	-5.19139800	1.63400200	3.36161900
S	-6.01436600	0.79879500	2.06459200
C	-4.18704300	2.62858300	3.02668600
C	-3.86658000	3.79837900	3.67384300
C	-2.82930100	4.56647700	3.06113900
C	-2.35855100	3.96425900	1.89106800
S	-3.19836400	2.46211300	1.58312300
C	-1.34058100	4.40077400	0.95135800
C	-0.99897300	5.67502100	0.56464800
C	0.04701500	5.75501200	-0.40512900
C	0.50255000	4.48929800	-0.78395200
S	-0.36218100	3.23444300	0.07307200
C	1.52230300	4.10492200	-1.74419400
C	1.87927800	4.70931400	-2.92622100
C	2.92140500	4.05485400	-3.65187500
C	3.35773100	2.89626500	-3.00365600
S	2.48063500	2.65000400	-1.51130500
C	4.36582600	1.92476300	-3.39049700
C	4.71972500	1.48851900	-4.64519400
C	5.74777200	0.49691200	-4.67529500
C	6.17622400	0.15334300	-3.39019300
S	5.30890200	1.06890200	-2.17927300
C	7.17053200	-0.81268700	-2.95666300
C	7.51137000	-2.02221500	-3.51392700
C	8.52854900	-2.74534300	-2.81855500
C	8.96157100	-2.06569500	-1.67721100

S	8.11229200	-0.54814000	-1.49630600
C	9.94783800	-2.43845100	-0.67772500
C	10.26198100	-3.68247200	-0.18519600
C	11.27771800	-3.69712000	0.82026100
C	11.73784300	-2.41161200	1.11286100
S	10.91476600	-1.21784500	0.13756700
C	12.73642500	-1.96437500	2.07166500
C	13.01974200	-2.45499600	3.32461400
C	14.05293300	-1.74331500	4.01277700
C	14.54096700	-0.70011100	3.25284500
S	13.77170100	-0.58043100	1.71916900
H	-16.09673700	0.21730200	-3.74135300
H	-15.36266400	-1.79958100	-5.26698900
H	-12.61677600	-4.51064000	-1.70066800
H	-9.91075200	-3.83295500	2.72245200
H	-7.16798400	-0.22924800	5.33333800
H	-4.35622000	4.11384600	4.58429100
H	-1.47855900	6.55481200	0.96951900
H	1.41269200	5.61762500	-3.28036000
H	4.26126200	1.87131800	-5.54598100
H	7.05078300	-2.39827000	-4.41645300
H	9.78254200	-4.58612600	-0.53434700
H	12.49691800	-3.29832100	3.75422300
H	15.32199700	-0.00100400	3.52033400
B	14.56736600	-2.07053200	5.42869600
B	11.77389100	-5.04755600	1.38321300
B	9.05829500	-4.07025400	-3.41067600
B	6.28948700	0.00888300	-6.03726000
B	3.46807100	4.70268700	-4.94339900
B	0.57725600	7.14106500	-0.83419000
B	-2.32359600	5.84860600	3.75884300
B	-5.16334300	1.60005300	6.01399200
B	-7.93282400	-3.01436400	4.52241900
B	-10.60500300	-5.19422500	0.12683100
B	-13.26909800	-3.53213200	-4.50678000
F	15.53772100	-1.35928700	5.99249100
F	14.05557500	-3.07241300	6.13360000
F	12.75760900	-5.17590000	2.26596900
F	11.19672300	-6.17264200	0.97101500
F	10.06239100	-4.77197700	-2.89789700
F	8.49387600	-4.55988800	-4.51075600
F	7.30425400	-0.83561400	-6.18240800
F	5.72457600	0.45389500	-7.15587300
F	4.49211500	4.22952100	-5.64444900
F	2.89823800	5.81282300	-5.40293300
F	1.60827900	7.33040000	-1.64980000
F	-0.01490900	8.23508100	-0.36427200
F	-1.31253900	6.59154000	3.32247200

F	-2.91812500	6.25616600	4.87625200
F	-4.15475200	2.43243900	6.24698600
F	-5.76219200	1.08184600	7.08223900
F	-6.94988400	-2.57654300	5.30175300
F	-8.50474000	-4.16038500	4.88021000
F	-9.60001500	-5.42314600	0.96508800
F	-11.15386000	-6.26288500	-0.44394800
F	-12.25833300	-4.28206600	-4.07989900
F	-13.75505600	-3.82902700	-5.71025100
BH_2			
C	15.81502000	-1.57924800	3.01542000
C	15.43209500	-2.83373500	3.37838200
C	14.34316600	-3.37723100	2.60475800
C	13.94259900	-2.47358800	1.60920500
S	14.88499700	-1.00379800	1.66407000
C	12.92965100	-2.64136700	0.58350200
C	12.59862200	-3.76893900	-0.12652800
C	11.52632200	-3.61485200	-1.06726400
C	11.06531800	-2.28839400	-1.08791000
S	11.92961400	-1.29167600	0.05412700
C	10.04585200	-1.69415300	-1.92913200
C	9.73371200	-1.97550300	-3.23747500
C	8.65052000	-1.21646800	-3.79116100
C	8.16023800	-0.28848800	-2.85735300
S	9.01202200	-0.39553200	-1.33844200
C	7.12097000	0.70659400	-3.02628700
C	6.80177000	1.43937200	-4.14433800
C	5.70042400	2.34499800	-3.99560400
C	5.20219000	2.31643800	-2.68220600
S	6.07018600	1.17226100	-1.69118300
C	4.14444700	3.11301500	-2.09489100
C	3.80229900	4.41846600	-2.35552000
C	2.68919600	4.92586500	-1.60827600
C	2.20575300	3.95909600	-0.71081100
S	3.10072700	2.46621700	-0.83141900
C	1.14364000	4.08022300	0.26668500
C	0.78596400	5.17432400	1.01775900
C	-0.32649100	4.97999300	1.90050400
C	-0.79333000	3.65649200	1.83425400
S	0.11686400	2.71105900	0.68423700
C	-1.85083500	3.02935800	2.60011000
C	-2.21286500	3.25033900	3.90740900
C	-3.32088800	2.47392200	4.38063000
C	-3.77928300	1.59598300	3.38423200
S	-2.86660600	1.76791000	1.90692700
C	-4.83188300	0.60428200	3.46574000
C	-5.19445700	-0.17934900	4.53507200
C	-6.29917000	-1.06333700	4.30583900

C	-6.75445100	-0.96301600	2.98041300
S	-5.84206800	0.21753300	2.07542100
C	-7.80508200	-1.71360300	2.32411500
C	-8.16970900	-3.02578200	2.50883900
C	-9.27406100	-3.47723200	1.71435900
C	-9.72643400	-2.45785600	0.85995700
S	-8.81194400	-0.98719000	1.07433400
C	-10.77614000	-2.50977200	-0.13694200
C	-11.14615800	-3.55822700	-0.94471500
C	-12.24711400	-3.29892000	-1.82575500
C	-12.69144200	-1.97335500	-1.69578100
S	-11.77397700	-1.10277300	-0.49488500
C	-13.73703400	-1.28938100	-2.43279200
C	-14.08713300	-1.42248600	-3.75503500
C	-15.17384700	-0.58195800	-4.17667100
C	-15.62291000	0.19671800	-3.12005900
S	-14.76630500	-0.07956000	-1.65785200
H	16.58395100	-0.95200000	3.44468300
H	15.88605500	-3.37774400	4.19899100
H	13.11562200	-4.70991100	0.01219100
H	10.27328700	-2.71629500	-3.81342000
H	7.34962700	1.34467400	-5.07305600
H	4.34160000	5.02528900	-3.07143300
H	1.31366200	6.11721100	0.95320000
H	-1.69171500	3.95779900	4.53954500
H	-4.67666400	-0.13662700	5.48469100
H	-7.65377200	-3.67665600	3.20299800
H	-10.63664100	-4.51299200	-0.92245000
H	-13.56018500	-2.08466500	-4.43015000
H	-16.42772500	0.92001300	-3.14099600
B	-15.77859600	-0.50360000	-5.58228000
H	-16.68699500	0.24177500	-5.79718400
H	-15.34976200	-1.18043400	-6.46796200
B	-12.88605000	-4.39375800	-2.68735800
H	-12.33547600	-5.45006100	-2.78018000
H	-13.92790600	-4.23059000	-3.24061400
B	-9.90946400	-4.85851900	1.90688100
H	-10.95882200	-5.14278600	1.42059500
H	-9.34844500	-5.66062200	2.59194000
B	-6.93230000	-1.89098000	5.42967100
H	-7.98113300	-2.43868500	5.29441300
H	-6.37002600	-1.96054600	6.48155800
B	-3.95361800	2.70426300	5.75731400
H	-5.00426400	2.22890600	6.05496200
H	-3.38917100	3.40765500	6.54097600
B	-0.95391000	6.13099500	2.69437800
H	-0.38393700	7.18018000	2.73728000
H	-2.00554600	6.01843200	3.24198300

B	2.07703700	6.30360200	-1.88302600
H	2.65974300	7.06167800	-2.59948400
H	1.02442500	6.62620400	-1.42877200
B	5.11364000	3.12297500	-5.17822500
H	5.70780800	3.13221100	-6.21476800
H	4.06959100	3.69122500	-5.10249600
B	8.07001200	-1.50813800	-5.17881700
H	8.65692900	-2.25314700	-5.90558600
H	7.03673600	-1.03859900	-5.54010600
B	10.92682800	-4.79991000	-1.83069900
H	11.48799000	-5.85432500	-1.79455600
H	9.90319100	-4.70832800	-2.43319100
B	13.66645400	-4.70638500	2.94688500
H	14.16359900	-5.41407100	3.77264300
H	12.64373400	-5.04352800	2.43672900
Br			
C	11.12882100	3.32304800	-6.52073000
C	10.68801100	4.61397500	-6.48857500
C	10.20596900	4.98582600	-5.20157700
C	10.27444000	3.98891700	-4.24856500
S	10.97839000	2.55096500	-4.97872500
C	9.88318400	3.98787200	-2.85257600
C	9.82327400	5.04012800	-1.96681100
C	9.39975300	4.65197500	-0.67233900
C	9.12633600	3.30502900	-0.52277600
S	9.40888200	2.49615400	-2.05878100
C	8.68085900	2.54836700	0.62725500
C	8.81855700	2.83738100	1.96804600
C	8.26145600	1.84541300	2.80962100
C	7.68497800	0.77272600	2.15399600
S	7.84756100	1.01630200	0.41983600
C	7.02736300	-0.41012100	2.66417700
C	7.15851200	-1.01968600	3.89394600
C	6.35428600	-2.17584700	4.02884100
C	5.58727200	-2.49301900	2.92206700
S	5.88703700	-1.29855100	1.66630500
C	4.65696800	-3.57527900	2.68865500
C	4.57144900	-4.80357200	3.30967100
C	3.52762900	-5.61164400	2.80094900
C	2.78340200	-5.04227300	1.78313300
S	3.41768400	-3.43544100	1.45159400
C	1.65728300	-5.54775000	1.02985300
C	1.25661200	-6.85138200	0.82412800
C	0.10173900	-6.95594900	0.01374500
C	-0.41946900	-5.75339800	-0.42852200
S	0.57566200	-4.44382400	0.19411000
C	-1.56727300	-5.46227200	-1.25849400
C	-2.20975800	-6.26918800	-2.17380800

C	-3.29670800	-5.62740800	-2.81276300
C	-3.52502700	-4.32157700	-2.41807300
S	-2.33290100	-3.88212000	-1.20207800
C	-4.52319800	-3.36272900	-2.83767900
C	-5.23707200	-3.30569600	-4.01605000
C	-6.12404700	-2.20482700	-4.07428800
C	-6.12217500	-1.38667000	-2.95923900
S	-4.96623500	-2.01979200	-1.79532200
C	-6.87815700	-0.19074100	-2.65851900
C	-7.45701700	0.71386800	-3.52267800
C	-8.11272100	1.77614500	-2.85600900
C	-8.06020300	1.72722900	-1.47504000
S	-7.15428500	0.29868800	-0.99427500
C	-8.60569600	2.61507400	-0.47087100
C	-8.93459900	3.94962600	-0.57299700
C	-9.44026200	4.48919600	0.63482500
C	-9.51435900	3.60193700	1.69046000
S	-8.92671700	2.03445100	1.15598900
C	-9.97328500	3.78257900	3.05382900
C	-9.97249200	4.92453700	3.82516700
C	-10.49447800	4.69742300	5.12743300
C	-10.88899900	3.41195300	5.36374500
S	-10.64198300	2.42943800	3.95953500
H	11.53988800	2.77801900	-7.35868900
H	10.69144200	5.28580600	-7.33697400
H	10.07658000	6.05769500	-2.22544200
H	9.30595700	3.72418500	2.34463800
H	7.81133000	-0.66156900	4.67586300
H	5.23344000	-5.12608200	4.09921100
H	1.76978300	-7.71010800	1.23006200
H	-1.91492600	-7.28473100	-2.39169400
H	-5.13073800	-4.01938900	-4.81933000
H	-7.41277400	0.63100200	-4.59842000
H	-8.81690400	4.53202000	-1.47468400
H	-9.60357800	5.88154600	3.48642600
H	-11.30990100	2.99274300	6.26515500
Br	9.49736200	6.72038800	-4.90918000
Br	9.21708400	5.95689900	0.69216900
Br	8.31683100	2.06657800	4.69257500
Br	6.36531900	-3.14507000	5.65923900
Br	3.22235300	-7.32989200	3.54447500
Br	-0.63426800	-8.66363300	-0.36099800
Br	-4.33506100	-6.57581800	-4.08561000
Br	-7.22817500	-1.96072400	-5.59729400
Br	-9.00453000	3.11426800	-3.86220100
Br	-9.98702800	6.30422900	0.70824400
Br	-10.61663200	6.07458700	6.42674000
CBr_3			

C	-18.03543100	0.82443700	1.53000600
C	-17.63945600	2.12425400	1.43136400
C	-16.42316200	2.29865000	0.69076200
C	-15.92728700	1.09055500	0.20804900
S	-16.95565900	-0.23470400	0.70013800
C	-14.76418200	0.78163800	-0.61761300
C	-14.40289800	1.32202300	-1.82335000
C	-13.18210500	0.81068900	-2.35794500
C	-12.63093600	-0.18478600	-1.55246400
S	-13.60685000	-0.42889700	-0.12481300
C	-11.45151400	-1.02361000	-1.72156800
C	-11.06740000	-1.74557600	-2.82079400
C	-9.83724700	-2.45228500	-2.66695000
C	-9.30139300	-2.30156100	-1.38642600
S	-10.30519600	-1.24403800	-0.42294100
C	-8.12245600	-2.87170300	-0.75272800
C	-7.64925800	-4.15579800	-0.82547900
C	-6.43746600	-4.38428100	-0.10904600
C	-6.01752600	-3.25098500	0.58894400
S	-7.09189200	-1.90718500	0.27906100
C	-4.91159300	-3.03746000	1.50853900
C	-4.46567300	-3.86585900	2.50509500
C	-3.32054900	-3.38700000	3.20703300
C	-2.93276600	-2.11766600	2.77631200
S	-3.94668200	-1.58083300	1.45695200
C	-1.90388700	-1.20884200	3.25636000
C	-1.57949000	-0.90708400	4.55334000
C	-0.48945300	0.00106800	4.69652300
C	-0.01854700	0.45523000	3.46382000
S	-0.88860300	-0.30963100	2.15445200
C	1.00086200	1.43449300	3.12139400
C	1.22562800	2.66022700	3.69184200
C	2.33900200	3.37034200	3.15299500
C	2.93359800	2.68741900	2.09105400
S	2.14570500	1.14873600	1.83229200
C	4.02288600	3.05070400	1.19839700
C	4.25861100	4.25856200	0.59525100
C	5.44852400	4.30687700	-0.18911000
C	6.09581400	3.07238400	-0.24004000
S	5.25799100	1.89877800	0.74821900
C	7.26774400	2.62650800	-0.97830100
C	7.58428300	2.86406500	-2.29059800
C	8.82674500	2.30359000	-2.71009400
C	9.44022900	1.56558400	-1.69793400
S	8.49717900	1.63720800	-0.22843300
C	10.65566100	0.76464200	-1.68668600
C	11.07306300	-0.15737600	-2.61020000
C	12.33494500	-0.75677000	-2.31882400

C	12.85847600	-0.31580700	-1.10409900
S	11.80959200	0.87844400	-0.37933900
C	14.06652200	-0.69125800	-0.38368400
C	14.55585400	-1.94524000	-0.13384800
C	15.80307600	-1.93624800	0.56496800
C	16.23105400	-0.66761200	0.86415600
S	15.14217400	0.53374500	0.27957000
H	-18.89928800	0.43119800	2.04689500
H	-18.17588300	2.94581300	1.88345100
H	-14.98509100	2.09158300	-2.30589400
H	-11.64397100	-1.75333000	-3.73269400
H	-8.14965900	-4.92191300	-1.39702600
H	-4.93616300	-4.81289100	2.72028700
H	-2.09866200	-1.34439300	5.39228400
H	0.61764600	3.03887400	4.49901600
H	3.60375700	5.10600400	0.72752500
H	6.95001500	3.44742900	-2.94021400
H	10.49860400	-0.38736300	-3.49442500
H	14.05358800	-2.85014500	-0.44792400
H	17.13410400	-0.38472900	1.38353000
C	16.55521000	-3.19818600	0.83433000
C	12.94947400	-1.76776300	-3.25018800
C	9.34988400	2.51799100	-4.10544700
C	5.89040600	5.58335200	-0.85397400
C	2.75762900	4.70165400	3.71873200
C	0.03078700	0.38046000	6.05768600
C	-2.66487100	-4.21859700	4.27739400
C	-5.75713700	-5.72749400	-0.14830900
C	-9.25907800	-3.26355400	-3.79613700
C	-12.61564400	1.31836900	-3.65684900
C	-15.80397500	3.65097000	0.46802300
Br	15.41441500	-4.49176500	1.80386200
Br	17.06965700	-4.02520200	-0.89593700
Br	18.18647600	-2.91590400	1.88793500
Br	12.49030000	-3.61445900	-2.65330300
Br	12.24863600	-1.57799500	-5.09136000
Br	14.90814000	-1.64492200	-3.40465400
Br	8.90089300	0.96184900	-5.26860400
Br	11.29646800	2.80617200	-4.18441700
Br	8.53291000	4.10743400	-4.95429000
Br	7.83959600	5.86192700	-0.81866700
Br	5.10372700	7.16910700	0.03001800
Br	5.28636600	5.63059500	-2.75287800
Br	4.70852600	4.95696800	3.80379600
Br	1.97954000	6.19207800	2.64586000
Br	2.10424700	4.93940300	5.57101400
Br	1.99016100	0.55506500	6.14411500
Br	-0.44922900	-0.97179800	7.41987700

Br	-0.77668900	2.10279800	6.65785400
Br	-0.69960600	-4.10117200	4.28865500
Br	-3.08687200	-6.13949600	4.05860000
Br	-3.33307300	-3.69061000	6.08078900
Br	-3.79106400	-5.63020100	-0.20608900
Br	-6.28961600	-6.76112400	-1.74848100
Br	-6.28127500	-6.82033400	1.43531100
Br	-7.30109400	-3.12627700	-3.95647300
Br	-9.98343700	-2.70385300	-5.54976000
Br	-9.73475400	-5.19012200	-3.58305800
Br	-10.65738600	1.53092000	-3.62617700
Br	-13.34782700	3.09033400	-4.13917500
Br	-13.08800900	0.08893700	-5.15506200
Br	-13.84212400	3.65071200	0.65991800
Br	-16.47574000	5.00314300	1.74632400
Br	-16.25239800	4.34093300	-1.34952900
CCH			
C	6.44680500	-5.77734600	6.32514500
C	6.58217300	-6.96816700	5.67871500
C	6.96145100	-6.82852000	4.30041600
C	7.11393900	-5.49374000	3.92068700
S	6.79504800	-4.43782800	5.28138700
C	7.48478400	-4.95064500	2.63129200
C	8.13583700	-5.56914400	1.58939200
C	8.34726300	-4.73328800	0.45167900
C	7.85080100	-3.43738700	0.62758000
S	7.11825800	-3.28421400	2.20949900
C	7.87688100	-2.31721800	-0.28282500
C	8.61959300	-2.15173300	-1.43153000
C	8.40317000	-0.90877600	-2.09560300
C	7.47398600	-0.09321800	-1.43967100
S	6.88010000	-0.89534400	-0.00220900
C	6.99803200	1.22074500	-1.79847200
C	7.52006100	2.11170200	-2.71134500
C	6.79593400	3.33497700	-2.81656600
C	5.69042500	3.39126600	-1.95964200
S	5.57030700	1.90981500	-1.03609100
C	4.72660800	4.44967200	-1.78016800
C	4.79426000	5.76223000	-2.19549300
C	3.65743500	6.54719400	-1.84483000
C	2.69083000	5.82335700	-1.13679300
S	3.21687100	4.16834100	-0.92208700
C	1.41937500	6.26423500	-0.61708100
C	0.95714800	7.54983200	-0.43473900
C	-0.35782600	7.62831800	0.10936700
C	-0.92273700	6.37148500	0.35775600
S	0.19938600	5.10819000	-0.09779500
C	-2.21984400	6.04346400	0.89684800

C	-3.12658600	6.86403600	1.53291600
C	-4.32293100	6.20805700	1.94474100
C	-4.33820100	4.84546100	1.62284800
S	-2.85434700	4.40514300	0.80615400
C	-5.36151900	3.85923300	1.86885800
C	-6.46812300	3.94287100	2.68661200
C	-7.27908100	2.77091900	2.69773900
C	-6.78358900	1.75490200	1.87152200
S	-5.30998000	2.28296300	1.08927600
C	-7.31767700	0.43876100	1.61950900
C	-8.30922100	-0.23907500	2.29574800
C	-8.59184100	-1.53621200	1.77638900
C	-7.79505500	-1.86991700	0.67511200
S	-6.70398800	-0.55369800	0.30285000
C	-7.78466300	-3.07858200	-0.11397400
C	-8.36384800	-4.29787700	0.16262700
C	-8.15629200	-5.28613000	-0.84514300
C	-7.39509900	-4.81809200	-1.91916200
S	-6.94489800	-3.14899700	-1.65836800
C	-6.97966100	-5.51618500	-3.11687800
C	-6.87016500	-6.87259700	-3.32709600
C	-6.41952900	-7.21450300	-4.64328700
C	-6.18783300	-6.09853100	-5.42121300
S	-6.52759900	-4.63617500	-4.57479400
H	6.16785900	-5.59982000	7.35402500
H	6.41535800	-7.93296400	6.14148800
H	8.46606700	-6.59764400	1.62641600
H	9.31177500	-2.89539400	-1.79941400
H	8.40510300	1.90699900	-3.29636300
H	5.63705400	6.16731200	-2.73678700
H	1.54042000	8.42659700	-0.67683600
H	-2.94834200	7.91476200	1.71053500
H	-6.70300100	4.81826900	3.27469900
H	-8.82869900	0.17159200	3.14945100
H	-8.92582000	-4.49406300	1.06436000
H	-7.08874200	-7.60875700	-2.56665500
H	-5.84243600	-6.07174600	-6.44449500
C	-6.22314200	-8.55128000	-5.09393500
C	-6.05932200	-9.68980900	-5.46885300
H	-5.91255700	-10.69110000	-5.80161700
C	-8.70327100	-6.59443100	-0.73604100
C	-9.18761200	-7.69585300	-0.60093100
H	-9.61609400	-8.66557900	-0.49478700
C	-9.60084800	-2.36054300	2.34539900
C	-10.47048200	-3.02368100	2.86551800
H	-11.23858800	-3.61130100	3.31278300
C	-8.46756600	2.69791700	3.47448000
C	-9.47071500	2.68641300	4.15286000

H	-10.35838400	2.67029900	4.74203400
C	-5.36932100	6.91419800	2.59852300
C	-6.23102600	7.56096100	3.15151800
H	-6.99679400	8.12415500	3.63294200
C	-0.99136200	8.87934400	0.34318200
C	-1.48682100	9.96963500	0.52321300
H	-1.93252800	10.92492600	0.67851300
C	3.55585300	7.91633700	-2.21401600
C	3.51617600	9.07856900	-2.55193400
H	3.47029800	10.09999700	-2.85182400
C	7.18806800	4.35535000	-3.72553600
C	7.56192900	5.19374100	-4.51541300
H	7.88023700	5.93350200	-5.21311800
C	9.07576200	-0.58532200	-3.30560900
C	9.67303300	-0.35717600	-4.33412000
H	10.18910700	-0.15176400	-5.24341600
C	8.99034900	-5.21939400	-0.71910400
C	9.54317700	-5.68101800	-1.69276700
H	10.02238700	-6.08152600	-2.55605900
C	7.12509900	-7.95246200	3.44672000
C	7.25339000	-8.94129300	2.75917800
H	7.35889200	-9.80412400	2.14303200
CCl_3			
C	-18.87648400	-1.36434500	-1.81915900
C	-18.59912400	-2.44193400	-1.03160800
C	-17.39233500	-2.29537400	-0.27264500
C	-16.77909400	-1.06698600	-0.48577000
S	-17.69033900	-0.12531100	-1.64379100
C	-15.58036700	-0.45584300	0.08885700
C	-15.29349200	-0.18481200	1.40199600
C	-14.02867800	0.44630400	1.60421300
C	-13.35909800	0.69334200	0.41131200
S	-14.28860700	0.10344000	-0.94269900
C	-12.08416700	1.34927500	0.12034200
C	-11.68253000	2.62635600	0.41693700
C	-10.36860000	2.94554800	-0.04140700
C	-9.77578400	1.89557300	-0.73401400
S	-10.84148900	0.51437800	-0.77635700
C	-8.48590600	1.77028800	-1.41207500
C	-7.96419300	2.53180200	-2.42612600
C	-6.67587300	2.10659500	-2.87076700
C	-6.22822600	0.97273200	-2.20205100
S	-7.39377800	0.47126400	-1.00406300
C	-5.01657100	0.16513700	-2.34031100
C	-4.53647000	-0.49267500	-3.44349000
C	-3.32515100	-1.21234500	-3.21165800
C	-2.89857400	-1.12709100	-1.89077000
S	-3.98501200	-0.12501700	-0.96279700

C	-1.76441800	-1.71972100	-1.18286400
C	-1.41909300	-3.04035200	-1.05184600
C	-0.25208500	-3.26266900	-0.26012200
C	0.27795200	-2.08281600	0.25200800
S	-0.65747500	-0.71105200	-0.28664500
C	1.41348700	-1.81181700	1.13209200
C	1.68592000	-2.31576500	2.37831200
C	2.88057900	-1.80145600	2.96707700
C	3.50726900	-0.85535300	2.16329800
S	2.62926400	-0.64717300	0.66951000
C	4.70098100	-0.03367000	2.35881600
C	4.98685600	0.84982900	3.36798700
C	6.24391100	1.51143000	3.22432600
C	6.90808700	1.14782000	2.05824300
S	5.98410300	-0.04022300	1.17520300
C	8.17169400	1.59078600	1.46968200
C	8.55748000	2.85626200	1.10920700
C	9.86203600	2.92591300	0.53332000
C	10.46322400	1.67690400	0.42135200
S	9.41711100	0.43707600	1.06519500
C	11.74826400	1.25029600	-0.13140600
C	12.24827200	1.43056700	-1.39534200
C	13.53923500	0.85517700	-1.59845700
C	14.01148300	0.19110000	-0.47167100
S	12.86422900	0.32014100	0.83665300
C	15.23390800	-0.57201000	-0.22061100
C	15.71385900	-1.67783500	-0.87146500
C	16.92650300	-2.18357500	-0.30444600
C	17.34686000	-1.45609400	0.77903600
S	16.28483900	-0.14401700	1.12266600
H	-19.70976700	-1.23377400	-2.49498900
H	-19.22093400	-3.32478000	-0.99632000
H	-15.96845000	-0.43890800	2.20522500
H	-12.30912800	3.31806300	0.95923300
H	-8.48665100	3.38204300	-2.83749000
H	-5.03342900	-0.45503500	-4.40114500
H	-1.98208100	-3.83414700	-1.51890500
H	1.04968400	-3.04309100	2.85948700
H	4.31692800	1.01725800	4.19776100
H	7.92590800	3.71836300	1.26166000
H	11.70907400	1.96998800	-2.15918500
H	15.21698500	-2.12421900	-1.72190200
H	18.22251800	-1.62019100	1.38835600
C	17.62420800	-3.39243100	-0.86564400
C	14.26074900	0.94715100	-2.92740500
C	10.46847600	4.23587500	0.07332300
C	6.73784000	2.52488900	4.23656300
C	3.35126700	-2.23974000	4.33901700

C	0.28772800	-4.65481900	-0.00224500
C	-2.63977400	-2.00297900	-4.30743100
C	-5.93481700	2.82007900	-3.98321400
C	-9.74562900	4.30717200	0.19016100
C	-13.52922400	0.82452000	2.98360800
C	-16.89424900	-3.37113300	0.66818900
Cl	16.50807000	-4.82616800	-0.82621600
Cl	18.09163700	-3.09476300	-2.59310100
Cl	19.11294400	-3.81541900	0.05730300
Cl	16.02172900	1.28715300	-2.73044900
Cl	14.06500400	-0.61142000	-3.85617500
Cl	13.58465100	2.26834200	-3.96197700
Cl	10.29905400	4.40403800	-1.73566700
Cl	9.63107900	5.65521700	0.81832000
Cl	12.21529500	4.37351300	0.50677400
Cl	8.50339100	2.35762300	4.57231700
Cl	6.41415100	4.21681800	3.63540900
Cl	5.88505800	2.35092500	5.82159400
Cl	5.13963000	-2.47349500	4.41022400
Cl	2.87255500	-0.99990700	5.58937900
Cl	2.59876800	-3.80830500	4.83010200
Cl	2.08902000	-4.72163400	-0.09334900
Cl	-0.23798900	-5.23336900	1.64698000
Cl	-0.34169700	-5.84712700	-1.20707600
Cl	-0.84431700	-1.82496400	-4.26483700
Cl	-3.17897000	-1.47107800	-5.94896000
Cl	-3.05684900	-3.77300700	-4.15329800
Cl	-4.16939000	2.97872000	-3.64244300
Cl	-6.57022400	4.49325400	-4.23940300
Cl	-6.14896900	1.91762400	-5.55448300
Cl	-8.00394300	4.19527900	0.65190300
Cl	-10.58272500	5.20648900	1.51617000
Cl	-9.88661400	5.32482900	-1.31830700
Cl	-11.77094800	0.47203800	3.19665300
Cl	-14.40541200	-0.08005300	4.28047100
Cl	-13.80923700	2.60199800	3.28618600
Cl	-15.10574300	-3.60114800	0.57015700
Cl	-17.33484700	-2.95584200	2.38949100
Cl	-17.64680700	-4.97711100	0.30234900
CF_3			
C	-14.96936500	-0.94338500	-4.24617500
C	-14.46271100	-1.98310800	-4.97112200
C	-13.43773600	-2.69431600	-4.28025500
C	-13.17531600	-2.19246400	-3.01556200
S	-14.21939300	-0.82413600	-2.69267600
C	-12.22692000	-2.63834800	-2.00719600
C	-11.82814200	-3.91868000	-1.69939900
C	-10.87473200	-3.98248700	-0.65074600

C	-10.54069100	-2.74338700	-0.12228000
S	-11.41966600	-1.48616300	-0.96276500
C	-9.63199400	-2.39205600	0.95436900
C	-9.31399900	-3.10422700	2.08940800
C	-8.38053500	-2.44824400	2.93099500
C	-7.98013400	-1.20478900	2.46167200
S	-8.76977200	-0.86534200	0.93866000
C	-7.06568400	-0.23293300	3.03319800
C	-6.81937800	0.05236700	4.35786600
C	-5.85500300	1.07309800	4.55141400
C	-5.35663100	1.60454900	3.36923300
S	-6.09535900	0.80152700	2.00255100
C	-4.37870000	2.65369100	3.14750500
C	-4.10926700	3.76496800	3.91536200
C	-3.07561200	4.58217900	3.39357400
C	-2.54354600	4.11798900	2.19731100
S	-3.34188800	2.63219000	1.73386400
C	-1.49223700	4.66143100	1.35760800
C	-1.11825200	5.97404900	1.16940800
C	-0.03548600	6.13553700	0.26934500
C	0.43012000	4.94234500	-0.26814800
S	-0.49202400	3.60453500	0.37889600
C	1.49484600	4.68719900	-1.22036900
C	1.95512300	5.48450400	-2.24536600
C	3.01516500	4.90538600	-2.98644200
C	3.37543600	3.63629400	-2.55228900
S	2.38387800	3.17581500	-1.18736400
C	4.38428100	2.72004600	-3.05099300
C	4.84745300	2.55574900	-4.33789400
C	5.84149300	1.55315000	-4.46246700
C	6.14499200	0.91307900	-3.26824800
S	5.18437900	1.59141200	-1.97350200
C	7.07808300	-0.16212200	-2.98629400
C	7.46298800	-1.21000300	-3.79296300
C	8.39524100	-2.08634000	-3.18265700
C	8.72865300	-1.73269400	-1.88190100
S	7.87477900	-0.27548400	-1.42846100
C	9.62000700	-2.37053500	-0.93003100
C	9.91253700	-3.70751400	-0.77821200
C	10.83284500	-3.97339800	0.26759200
C	11.24755200	-2.83973100	0.95055600
S	10.48865200	-1.42316000	0.26319700
C	12.15631300	-2.69798200	2.07680000
C	12.34185800	-3.54116500	3.14800000
C	13.30962600	-3.05582900	4.07252700
C	13.85023300	-1.85113100	3.71208200
S	13.19959200	-1.28728400	2.21857700
H	-15.73782800	-0.23940900	-4.53223800

H	-14.78582900	-2.23603700	-5.97225300
H	-12.20083800	-4.79377200	-2.21131200
H	-9.73254600	-4.07290300	2.31877000
H	-7.31144400	-0.45346400	5.17539300
H	-4.63260900	3.99391200	4.83169100
H	-1.60083100	6.80553200	1.66088300
H	1.54896000	6.45991200	-2.46760200
H	4.49010700	3.13486500	-5.17630500
H	7.09201800	-1.35371100	-4.79692200
H	9.48511700	-4.48310700	-1.39612500
H	11.79393700	-4.46391800	3.27942600
H	14.59440200	-1.27661100	4.24375300
C	13.68778200	-3.79457500	5.31839800
C	11.28687400	-5.37340700	0.56856700
C	8.94094500	-3.27675600	-3.91886700
C	6.48061800	1.25378700	-5.78874300
C	3.65813900	5.64168500	-4.12728600
C	0.52621800	7.49578600	-0.03290900
C	-2.62943900	5.82289300	4.11345400
C	-5.44333300	1.50113200	5.93154000
C	-7.89716000	-3.08494400	4.20281500
C	-10.31019000	-5.29665000	-0.19162300
C	-12.72940700	-3.85896700	-4.90776400
F	-11.38943700	-3.78059200	-4.78053000
F	-13.00835400	-3.94227500	-6.22534100
F	-13.10624300	-5.04253300	-4.35327400
F	-10.63554100	-6.28736600	-1.04497900
F	-8.96618600	-5.27261700	-0.08629200
F	-10.79174900	-5.65590500	1.02927100
F	-8.26146700	-4.38072000	4.26446500
F	-8.41842900	-2.47796500	5.30286600
F	-6.55599600	-3.03044200	4.32780400
F	-5.93117500	2.73148600	6.24653600
F	-5.91099100	0.64675800	6.86247100
F	-4.10424500	1.56691400	6.07265200
F	-3.17030200	5.88856600	5.34556900
F	-1.28963500	5.88422400	4.25113000
F	-3.00816300	6.95300200	3.45782500
F	0.02777100	8.42268300	0.80830600
F	1.87001300	7.53098900	0.06962700
F	0.21902400	7.90383500	-1.29384000
F	3.26566700	6.93050900	-4.15401800
F	3.32207100	5.10395600	-5.33072300
F	5.00427300	5.62514300	-4.05664900
F	6.16401600	2.19455900	-6.70011800
F	6.06490900	0.06170600	-6.29487100
F	7.82508500	1.19545300	-5.71076400
F	8.62396000	-3.22338200	-5.22772100

F	10.28241500	-3.36959300	-3.82642300
F	8.43340700	-4.44408200	-3.43883500
F	10.91636500	-6.22178100	-0.41161300
F	10.74219600	-5.84562000	1.72180700
F	12.62436200	-5.46384100	0.70370000
F	14.27084400	-4.98029300	5.03617500
F	12.60252400	-4.06548900	6.07766800
F	14.55163200	-3.08508400	6.07465300
CH_3			
C	-15.44447700	-0.97990500	-3.72559400
C	-14.93573100	-2.01272300	-4.45986200
C	-13.86494100	-2.71035900	-3.81673900
C	-13.57758000	-2.18216300	-2.56904200
S	-14.63894000	-0.82853000	-2.20167000
C	-12.57079300	-2.60478700	-1.61020100
C	-12.13652800	-3.87932100	-1.31915700
C	-11.11547400	-3.95406700	-0.32843400
C	-10.76918300	-2.70431900	0.16190800
S	-11.70474200	-1.44171500	-0.62495500
C	-9.78926200	-2.36326900	1.17599900
C	-9.42037400	-3.07553300	2.29751200
C	-8.41014000	-2.46314800	3.09200400
C	-8.00264700	-1.24295300	2.57347000
S	-8.86976100	-0.87213400	1.09086200
C	-7.00877900	-0.32095500	3.08865800
C	-6.66619600	-0.06039200	4.39896000
C	-5.62975200	0.90040100	4.56864500
C	-5.17390600	1.39997700	3.35752500
S	-6.02923900	0.65895600	2.01332000
C	-4.14194200	2.38855100	3.11101300
C	-3.78266600	3.47598900	3.87962600
C	-2.70887200	4.25114200	3.35808000
C	-2.24013900	3.75340800	2.15091300
S	-3.13083700	2.31398700	1.67978700
C	-1.16986900	4.25676400	1.31222200
C	-0.75496500	5.55879600	1.12409800
C	0.34591200	5.71560600	0.23578100
C	0.77985900	4.50369000	-0.28174900
S	-0.17813800	3.17421900	0.35257700
C	1.86099200	4.24419800	-1.21233500
C	2.33857600	5.04059100	-2.23257200
C	3.43008700	4.49111200	-2.96220100
C	3.79176000	3.23446600	-2.49920100
S	2.77924900	2.75102800	-1.14702800
C	4.83847200	2.35270700	-2.97803600
C	5.32386900	2.19919800	-4.26002900
C	6.37009000	1.24360400	-4.39514200
C	6.68779500	0.63861900	-3.18800800

S	5.69138900	1.27516900	-1.88811400
C	7.67958000	-0.38346000	-2.91408800
C	8.11463400	-1.40838700	-3.72794400
C	9.11402700	-2.24571900	-3.15608500
C	9.44515000	-1.86120200	-1.86554300
S	8.52132400	-0.44899200	-1.37655500
C	10.40074300	-2.46000900	-0.95284200
C	10.74886600	-3.78657800	-0.81327800
C	11.73576300	-4.04602600	0.18093800
C	12.14439400	-2.89106000	0.82763500
S	11.31039700	-1.48441700	0.18663300
C	13.11556100	-2.74008700	1.89801100
C	13.36962100	-3.58270400	2.95741400
C	14.40438200	-3.13051800	3.83623300
C	14.92419500	-1.93047100	3.42846600
S	14.18459200	-1.34538800	1.97304300
H	-16.24112900	-0.29817900	-3.98848400
H	-15.29760700	-2.27340400	-5.44879400
H	-12.55600400	-4.75965100	-1.79197600
H	-9.88164900	-4.01770300	2.56846100
H	-7.16439700	-0.53186600	5.23755000
H	-4.29428100	3.73691600	4.79824000
H	-1.24474500	6.40194500	1.59611200
H	1.90108400	6.00059100	-2.47844200
H	4.92211600	2.74366000	-5.10599500
H	7.70866300	-1.58096800	-4.71741100
H	10.28816800	-4.57550100	-1.39573500
H	12.80136400	-4.48928500	3.12999600
H	15.70490500	-1.35230400	3.90398800
C	14.84555800	-3.88070100	5.06401900
H	15.23399600	-4.87295200	4.80695300
H	15.63284800	-3.34162600	5.59721900
H	14.01194900	-4.03066800	5.75963400
C	12.25865800	-5.42966500	0.45998200
H	13.28042500	-5.39986400	0.84697200
H	11.64222500	-5.95572400	1.19982000
H	12.25357500	-6.03453500	-0.45187200
C	9.72792800	-3.39823900	-3.90493100
H	10.76312500	-3.56869600	-3.59732600
H	9.17753100	-4.33274700	-3.73770700
H	9.71715300	-3.20610200	-4.98190900
C	7.04013600	0.96005700	-5.71287200
H	8.08956800	0.68521400	-5.57659800
H	6.55239300	0.13653300	-6.24968400
H	6.99756400	1.83883000	-6.36301700
C	4.09969300	5.22883100	-4.09060300
H	5.16467400	4.98820700	-4.14929500
H	3.65320100	4.98130600	-5.06198600

H	3.99961300	6.30976200	-3.95548600
C	0.95257100	7.05910600	-0.06856900
H	2.02843000	6.98075500	-0.24788400
H	0.50416500	7.51708500	-0.95931300
H	0.79334900	7.75014400	0.76432100
C	-2.16102900	5.45654800	4.07408200
H	-1.08863500	5.57414600	3.89599100
H	-2.65130800	6.38268200	3.74835000
H	-2.32176300	5.36849000	5.15253200
C	-5.10408700	1.28990700	5.92426800
H	-4.04243000	1.54798400	5.88252600
H	-5.63685500	2.15691500	6.33493300
H	-5.22924400	0.46808300	6.63536700
C	-7.86125800	-3.10656700	4.33726000
H	-6.81154200	-2.84383400	4.49378900
H	-8.41401100	-2.79776600	5.23357500
H	-7.93591700	-4.19603800	4.27190000
C	-10.49657600	-5.25847900	0.09732800
H	-9.45669600	-5.12521800	0.40715700
H	-11.03544300	-5.70986400	0.94005600
H	-10.51993700	-5.98082000	-0.72404300
C	-13.13736300	-3.85721000	-4.46520700
H	-12.10897600	-3.93465700	-4.10313600
H	-13.62981300	-4.81729600	-4.26464400
H	-13.11047400	-3.73068600	-5.55185000
CHO			
C	-5.90299400	0.08706700	-3.05086200
C	-7.08454800	0.75825900	-3.07098500
C	-6.98134000	2.14177900	-2.70365300
C	-5.66210200	2.51448100	-2.39021400
S	-4.60242400	1.12973600	-2.57808700
C	-5.08358500	3.76939600	-1.95948500
C	-5.64570700	5.01951900	-1.83656500
C	-4.75918800	6.03963700	-1.38889600
C	-3.45317300	5.56689100	-1.15360600
S	-3.38450300	3.85150500	-1.49438100
C	-2.24836500	6.22203800	-0.70587400
C	-2.02284500	7.53571300	-0.35442000
C	-0.69161100	7.83383200	0.04510400
C	0.15962000	6.71045500	0.00191200
S	-0.74630600	5.31235600	-0.53441800
C	1.55998700	6.54203000	0.29966200
C	2.48649300	7.44344700	0.78034000
C	3.79755100	6.92462200	0.95647900
C	3.90095300	5.56232000	0.60757300
S	2.34669200	4.98234000	0.05041400
C	5.00351300	4.63460600	0.63793300
C	6.34396000	4.84568600	0.88420300

C	7.16140800	3.68607400	0.80543300
C	6.43221000	2.51994500	0.49175600
S	4.73955700	2.92259900	0.30385000
C	6.82766300	1.14426900	0.32362000
C	8.07983000	0.56568600	0.33426200
C	8.09681800	-0.83785400	0.11441500
C	6.80816700	-1.38180300	-0.06420400
S	5.61581500	-0.10480800	0.03284100
C	6.34670800	-2.72923200	-0.28438600
C	7.05081100	-3.88179000	-0.56329700
C	6.25500800	-5.04431300	-0.74838800
C	4.87470600	-4.79461000	-0.59917800
S	4.62281300	-3.10106300	-0.23809200
C	3.71994700	-5.65363200	-0.67518900
C	3.61371000	-6.97926400	-1.04117200
C	2.29465300	-7.50671000	-1.01779100
C	1.33224400	-6.56179600	-0.60629800
S	2.11671400	-5.03243000	-0.27871800
C	-0.09222700	-6.65323900	-0.40695300
C	-0.97823300	-7.65799100	-0.73359200
C	-2.33465300	-7.39744100	-0.39919900
C	-2.51814500	-6.13926400	0.21089200
S	-0.97316000	-5.33129400	0.36057700
C	-3.69167200	-5.45995000	0.70095400
C	-4.99418800	-5.89593500	0.81852500
C	-5.89920200	-4.94626000	1.36756500
C	-5.28044700	-3.72643200	1.70233800
S	-3.58302900	-3.79845300	1.28506800
C	-5.78520400	-2.51211400	2.30384100
C	-7.08559300	-2.10863600	2.54210500
C	-7.17802200	-0.82207800	3.14351200
C	-5.94575000	-0.24423400	3.36544300
S	-4.66189800	-1.25672000	2.85529600
H	-5.70618800	-0.94935100	-3.28498600
H	-8.02840900	0.29781200	-3.34113700
H	-6.68931700	5.19232300	-2.06530300
H	-2.81736000	8.27012500	-0.38977900
H	2.21229400	8.46773000	0.99856900
H	6.72326400	5.83096700	1.12296000
H	8.97070000	1.15709800	0.50284700
H	8.13132000	-3.87862200	-0.62604600
H	4.48584000	-7.55735600	-1.31867000
H	-0.64173200	-8.57227900	-1.20506600
H	-5.28497000	-6.89057900	0.50582300
H	-7.93099200	-2.73596100	2.29215200
H	-5.75728700	0.72217600	3.81242200
C	-8.44877900	-0.15938700	3.50039600
O	-8.52108500	0.94370300	4.00755800

H	-9.36112700	-0.74889600	3.26973900
C	-7.30162300	-5.36828800	1.52205800
O	-8.25515200	-4.69945700	1.88448800
H	-7.45010900	-6.43419700	1.25390900
C	-3.31955600	-8.43630600	-0.73985200
O	-4.51905000	-8.44328900	-0.51451900
H	-2.85643300	-9.29686000	-1.26369500
C	2.12921300	-8.91926300	-1.39635600
O	1.09216300	-9.55158100	-1.51572300
H	3.10025100	-9.41805000	-1.59042600
C	6.96293700	-6.29933700	-1.04743000
O	6.48640000	-7.39754800	-1.28519200
H	8.06407400	-6.16972000	-1.04557500
C	9.40961400	-1.50288400	0.12236800
O	9.66291800	-2.67472400	-0.10617300
H	10.23323200	-0.80242200	0.36812400
C	8.59943800	3.86392300	1.06233400
O	9.48501600	3.02512200	1.01828000
H	8.85149400	4.91189100	1.32218500
C	4.81886300	7.84805800	1.47553700
O	6.00779500	7.63687300	1.65319400
H	4.39863000	8.84589100	1.71451900
C	-0.40531300	9.22609800	0.42555300
O	0.64711800	9.70199500	0.82068400
H	-1.29521000	9.87923600	0.32174500
C	-5.31702500	7.39320200	-1.24602500
O	-4.75070400	8.41178400	-0.88197100
H	-6.39152700	7.43342500	-1.51675300
C	-8.21531000	2.93641700	-2.69765200
O	-8.35207000	4.12810100	-2.46962700
H	-9.10932000	2.32505500	-2.94270200
Cl			
C	13.03007300	2.64506500	-5.15449400
C	12.51335000	3.88787400	-5.37902300
C	11.72516000	4.34734800	-4.28503200
C	11.63763000	3.46377000	-3.22652800
S	12.57816600	2.02707400	-3.60196500
C	10.92856200	3.57704500	-1.96659200
C	10.64610100	4.70453700	-1.22955300
C	9.93528200	4.42107700	-0.03623900
C	9.66058600	3.08263600	0.17776700
S	10.30696000	2.14709600	-1.16104600
C	8.96355200	2.42810400	1.26457600
C	8.84468700	2.81883300	2.58037100
C	8.08993600	1.90910200	3.36111600
C	7.61642100	0.80261000	2.67846400
S	8.12872200	0.90589000	1.00152900
C	6.81624500	-0.31283700	3.13584200

C	6.72890700	-0.85986800	4.39757100
C	5.84008700	-1.96063000	4.46276200
C	5.22870900	-2.29320300	3.26652100
S	5.78190300	-1.18774000	2.01839700
C	4.27074400	-3.33187600	2.95632100
C	4.06651800	-4.54264100	3.58237800
C	3.03456100	-5.30699500	2.98542100
C	2.42459700	-4.71795800	1.89140400
S	3.16575700	-3.15150100	1.60323700
C	1.34622200	-5.18566600	1.04846800
C	0.94498000	-6.47743100	0.78290400
C	-0.15326000	-6.54335500	-0.10870700
C	-0.62160900	-5.32012000	-0.55601300
S	0.34341900	-4.04339900	0.16840900
C	-1.71083000	-4.99791300	-1.45165500
C	-2.26982500	-5.76279700	-2.45302400
C	-3.31945400	-5.10017700	-3.13462900
C	-3.59310000	-3.81910700	-2.68850400
S	-2.50265700	-3.43229300	-1.36690200
C	-4.57984400	-2.85863800	-3.13228600
C	-5.16326400	-2.72347100	-4.37354600
C	-6.08418100	-1.64937400	-4.43767600
C	-6.23086900	-0.93076600	-3.26436400
S	-5.18430400	-1.62599400	-2.03684500
C	-7.06857700	0.20851600	-2.95745700
C	-7.54007900	1.19097500	-3.80060600
C	-8.32877700	2.16032900	-3.13323800
C	-8.48039000	1.95831600	-1.77320500
S	-7.61021600	0.50376300	-1.31312000
C	-9.20874100	2.72361900	-0.78351600
C	-9.49249100	4.07133500	-0.76592400
C	-10.22586400	4.46613900	0.38124800
C	-10.51768300	3.44825400	1.26918800
S	-9.85675900	1.94439400	0.65093900
C	-11.24339500	3.47305200	2.52452100
C	-11.33164100	4.48823100	3.45072000
C	-12.13484800	4.13140600	4.56924400
C	-12.65289600	2.86806400	4.51262000
S	-12.17595200	2.07660200	3.04951700
H	13.65742700	2.05504400	-5.80748800
H	12.66863200	4.46557600	-6.28112000
H	10.94189000	5.70354100	-1.51653600
H	9.28854400	3.71614800	2.98673700
H	7.28630400	-0.50216200	5.25105100
H	4.63740100	-4.88806300	4.43188100
H	1.42019600	-7.35536000	1.19547800
H	-1.94017300	-6.75924100	-2.70839600
H	-4.93833500	-3.35647400	-5.21952600

H	-7.32499200	1.23406500	-4.85847500
H	-9.18198500	4.76508700	-1.53387300
H	-10.83115200	5.44101500	3.35343100
H	-13.28257700	2.37032000	5.23478600
Cl	10.91927800	5.89311300	-4.35072000
Cl	9.44458700	5.71106300	1.03045600
Cl	7.78785700	2.23010100	5.04879900
Cl	5.55554300	-2.77798900	5.97721400
Cl	2.59076900	-6.85889500	3.64682600
Cl	-0.84058800	-8.08493000	-0.54948600
Cl	-4.18771700	-5.90515200	-4.41571400
Cl	-6.96281300	-1.32440800	-5.90934700
Cl	-9.05579900	3.47430100	-4.02114700
Cl	-10.72229900	6.12600300	0.58994400
Cl	-12.41408800	5.22265700	5.90329000
CN			
C	7.04459500	-5.65855600	6.36136200
C	7.09354600	-6.85898500	5.71864000
C	7.35932000	-6.72540700	4.31753600
C	7.51688300	-5.40163500	3.90928600
S	7.34013900	-4.33552100	5.28154400
C	7.79450600	-4.88763100	2.58375600
C	8.35241500	-5.53725800	1.50590200
C	8.47533700	-4.71490600	0.34997600
C	8.01395700	-3.40988900	0.53546800
S	7.41683600	-3.22324000	2.16479200
C	7.98433100	-2.31103600	-0.40260900
C	8.65940700	-2.17306800	-1.59684600
C	8.38694900	-0.94643100	-2.26333100
C	7.49433800	-0.11736900	-1.57852900
S	6.99524300	-0.88977700	-0.09526900
C	6.99268500	1.18577100	-1.94724200
C	7.46322700	2.05565100	-2.90873700
C	6.71498700	3.26163400	-2.99654900
C	5.65315500	3.33514000	-2.09056900
S	5.59824900	1.87952000	-1.12988400
C	4.69099900	4.39420000	-1.89645800
C	4.74200300	5.70141400	-2.33351200
C	3.60811500	6.47034300	-1.95331100
C	2.66548700	5.75643000	-1.20770600
S	3.21025800	4.11299000	-0.98969400
C	1.40734600	6.20462000	-0.65935800
C	0.95385400	7.49386600	-0.47332800
C	-0.34559600	7.56029100	0.09955400
C	-0.91009100	6.30934900	0.36734500
S	0.20021900	5.04982200	-0.10879900
C	-2.19729600	5.99219200	0.93860100
C	-3.07994300	6.82090900	1.59983700

C	-4.26259300	6.16070900	2.03137000
C	-4.30166800	4.80094400	1.70714800
S	-2.84428600	4.35846500	0.85507000
C	-5.33093600	3.82635800	1.97894600
C	-6.40548500	3.91750600	2.83932800
C	-7.22010200	2.75276400	2.85436100
C	-6.77438300	1.73957400	1.99956500
S	-5.32730500	2.26137100	1.17526100
C	-7.34180400	0.43522000	1.75516800
C	-8.30841300	-0.23813500	2.47282100
C	-8.62378500	-1.51711200	1.93819500
C	-7.89309600	-1.84761700	0.79237200
S	-6.81073200	-0.53897100	0.39005500
C	-7.94439100	-3.04512300	-0.01289100
C	-8.57101800	-4.24422500	0.25601100
C	-8.41126200	-5.21008600	-0.77625700
C	-7.64932900	-4.76085600	-1.85621600
S	-7.12951800	-3.12138700	-1.57040800
C	-7.28566800	-5.45897000	-3.07098900
C	-7.25269500	-6.81729400	-3.30313600
C	-6.83496000	-7.14285800	-4.63002000
C	-6.55295400	-6.03001300	-5.39397800
S	-6.80474600	-4.57495200	-4.51588900
H	6.85848500	-5.46583100	7.40830300
H	6.94163600	-7.81607000	6.20137500
H	8.67215300	-6.57045200	1.52424700
H	9.33457600	-2.91646600	-1.99835000
H	8.31739800	1.85325000	-3.54046300
H	5.55963900	6.11455600	-2.90833800
H	1.52224800	8.37706900	-0.73146200
H	-2.90172500	7.87217400	1.78072400
H	-6.61853600	4.78398100	3.45046800
H	-8.78695700	0.15510200	3.35939300
H	-9.13435200	-4.44392700	1.15730100
H	-7.50338000	-7.56441900	-2.56245100
H	-6.22131100	-6.00341400	-6.42208500
C	-6.70667200	-8.47820000	-5.12012700
N	-6.59786800	-9.56503600	-5.51927600
C	-8.99421900	-6.50856900	-0.69954700
N	-9.46097200	-7.57239500	-0.63020900
C	-9.60214400	-2.36385000	2.53439000
N	-10.39501700	-3.06039700	3.02536900
C	-8.38295700	2.65289400	3.67137800
N	-9.32804400	2.56443000	4.34505200
C	-5.30190400	6.84532500	2.72483900
N	-6.14865400	7.39921200	3.30021200
C	-0.99479600	8.80088800	0.36276300
N	-1.52414700	9.81270000	0.58826100

C	3.46386800	7.84063900	-2.31606500
N	3.34698300	8.96295400	-2.60158500
C	7.03249500	4.28511700	-3.93559200
N	7.29440100	5.12789100	-4.69448800
C	8.97809700	-0.61691400	-3.51712800
N	9.46608300	-0.33966800	-4.53687100
C	9.01981500	-5.20116600	-0.87348100
N	9.47382000	-5.59258400	-1.87131300
C	7.43146900	-7.84592400	3.44166900
N	7.49802100	-8.76748600	2.73319800
COOH			
C	4.77396500	6.50333500	-4.08836000
C	5.73737900	7.45334600	-3.94616400
C	6.83440200	7.03764400	-3.12104900
C	6.68036400	5.73997700	-2.62864600
S	5.17739800	5.05913200	-3.22250200
C	7.49297100	4.92123100	-1.74385300
C	8.85510700	4.85797300	-1.58148800
C	9.29105700	3.87547900	-0.64506000
C	8.24016100	3.15297200	-0.07085100
S	6.71539700	3.74414200	-0.69258300
C	8.19916500	2.05462100	0.87746500
C	9.04368300	1.72753900	1.91224100
C	8.62970400	0.59706800	2.67448500
C	7.43765400	0.02530500	2.21537200
S	6.84763000	0.92652400	0.83732900
C	6.67615200	-1.13744600	2.63258300
C	6.51626900	-1.70885500	3.87283600
C	5.62821000	-2.82220000	3.89785500
C	5.07863500	-3.12151600	2.64639500
S	5.71271000	-2.01531100	1.44869200
C	4.12480000	-4.11997300	2.20055700
C	3.86722300	-5.38809600	2.66603600
C	2.89346200	-6.10528900	1.91314800
C	2.37263600	-5.37304300	0.83936800
S	3.12786500	-3.79601700	0.78579000
C	1.35176200	-5.66164800	-0.15153100
C	0.95187800	-6.85277900	-0.71061800
C	-0.04515800	-6.72525200	-1.72026900
C	-0.44085500	-5.40122700	-1.94510000
S	0.46145100	-4.33474400	-0.89235200
C	-1.43781200	-4.80040600	-2.81233700
C	-1.93161900	-5.20281000	-4.03112200
C	-2.86660300	-4.30099900	-4.61628700
C	-3.11664300	-3.17118600	-3.82717900
S	-2.15578600	-3.25480300	-2.36749200
C	-4.01100400	-2.03765700	-3.97436100
C	-4.51737700	-1.42742900	-5.09844800

C	-5.32655200	-0.28456100	-4.83601500
C	-5.46120800	-0.00133700	-3.47128300
S	-4.55537000	-1.17224100	-2.53926400
C	-6.20669400	1.00348700	-2.73695500
C	-6.58642100	2.27855800	-3.08705000
C	-7.26431500	2.99376700	-2.05875500
C	-7.41073100	2.25997000	-0.87683500
S	-6.72158100	0.66650100	-1.08590900
C	-7.98489100	2.57873700	0.41644600
C	-9.03462100	3.39955400	0.75507900
C	-9.34655700	3.40924200	2.14530900
C	-8.52455300	2.57048800	2.90386900
S	-7.34662400	1.80479600	1.86386400
C	-8.49378000	2.23736400	4.31805500
C	-8.79777300	2.99864900	5.42475600
C	-8.57651100	2.31135600	6.65432900
C	-8.10735900	1.02990700	6.48041700
S	-7.93302800	0.63483900	4.82097400
H	3.85389600	6.55690800	-4.65285700
H	5.69276300	8.43296000	-4.40246800
H	9.53537400	5.50219500	-2.11484800
H	9.94836300	2.27708600	2.11752100
H	7.02461500	-1.34334900	4.75029300
H	4.35758200	-5.79628100	3.53516700
H	1.35510000	-7.80295400	-0.39943500
H	-1.63975900	-6.13077500	-4.49580300
H	-4.32376000	-1.79497500	-6.09326100
H	-6.38681900	2.69669800	-4.06039700
H	-9.57211800	3.98590000	0.02730400
H	-9.17250200	4.00772400	5.35507400
H	-7.87536800	0.31705300	7.25896300
C	-8.80190400	2.85698500	8.00981100
O	-8.61716700	2.24681500	9.04406500
C	-10.47763100	4.20854200	2.66498800
O	-10.70292400	4.49296600	3.82613500
C	-7.69677000	4.39267700	-2.26484700
O	-8.46528200	5.03314500	-1.57206800
C	-5.96341300	0.45832700	-5.94445800
O	-6.52633500	1.53583300	-5.88088500
C	-3.49665100	-4.61175400	-5.91746800
O	-4.15611800	-3.86230800	-6.61402500
C	-0.58876400	-7.92182200	-2.39820600
O	-1.29101400	-7.94858000	-3.39199600
C	2.49382100	-7.47391100	2.30624500
O	1.82142700	-8.25712100	1.66144100
C	5.32031600	-3.51551200	5.16745500
O	4.77873900	-4.59696600	5.30080600
C	9.44672300	0.10333500	3.80401200

O	9.12507300	-0.72389800	4.63693100
C	10.73134000	3.65722500	-0.39255900
O	11.23025200	3.03311300	0.52522000
C	7.95489100	7.95018800	-2.81967100
O	9.01656900	7.66889300	-2.29622800
O	-9.24299000	4.13786700	7.98391100
H	-9.36198200	4.39784000	8.91254200
O	-11.29204500	4.62788800	1.66361600
H	-11.97971500	5.16372800	2.09241600
O	-7.12597800	4.93335900	-3.37106700
H	-7.48971400	5.83152900	-3.44035200
O	-5.85133900	-0.20776200	-7.12086600
H	-6.27801000	0.35934600	-7.78417500
O	-3.25012800	-5.88995900	-6.29834300
H	-3.68186900	-5.99366800	-7.16237100
O	-0.20164700	-9.06491600	-1.77914200
H	-0.58585800	-9.78843900	-2.30126100
O	2.98299100	-7.80510400	3.52718600
H	2.68793100	-8.71639400	3.68905400
O	5.72740300	-2.79294600	6.24126200
H	5.51309200	-3.33520400	7.01838200
O	10.66490100	0.69898300	3.83630600
H	11.11632200	0.32996700	4.61331700
O	11.49992700	4.25982400	-1.33509900
H	12.41909000	4.08968300	-1.07045200
O	7.67963500	9.22344200	-3.21191900
H	8.47340600	9.73892500	-2.99273900
F			
C	-4.41275800	-8.58037900	-0.46742100
C	-5.62740800	-9.20215400	-0.39268600
C	-6.68047700	-8.26146100	-0.27911200
C	-6.30248400	-6.93418800	-0.26499800
S	-4.55403100	-6.85304700	-0.39946200
C	-7.15376000	-5.77390300	-0.16108400
C	-8.53100200	-5.74041400	-0.05720400
C	-9.02133800	-4.42340800	0.02439400
C	-8.07848400	-3.41395500	-0.01117200
S	-6.48888200	-4.14777300	-0.15475400
C	-8.29689900	-1.99419100	0.05312600
C	-9.50264300	-1.32330500	0.15900500
C	-9.32734700	0.07146600	0.19366500
C	-8.02502400	0.53053300	0.11981500
S	-6.95602400	-0.85840700	-0.00118400
C	-7.56326700	1.89104400	0.13347000
C	-8.32353700	3.04578900	0.20250200
C	-7.52521800	4.20283400	0.19304900
C	-6.15802400	4.00773100	0.11947600
S	-5.84988100	2.27885400	0.05730300

C	-5.12117300	5.00169000	0.09555600
C	-5.26194000	6.37892600	0.10808200
C	-4.01928200	7.03521000	0.07688900
C	-2.89654700	6.22825000	0.04058200
S	-3.42195700	4.55150400	0.04636900
C	-1.51777300	6.62956500	0.00509600
C	-1.00662300	7.91606600	-0.01303000
C	0.39860300	7.92372800	-0.04659300
C	1.02201200	6.68913600	-0.05506400
S	-0.21840300	5.44476500	-0.02063300
C	2.43042000	6.40817200	-0.08478900
C	3.47738400	7.31256900	-0.13565100
C	4.72768100	6.67023100	-0.15002300
C	4.71132700	5.28788500	-0.11232400
S	3.03697800	4.75757800	-0.05461200
C	5.83142900	4.38831200	-0.11596200
C	7.17785800	4.70662300	-0.16192700
C	7.99089800	3.55975200	-0.14903700
C	7.33835000	2.34183000	-0.09433400
S	5.60787400	2.64477800	-0.05635100
C	7.91735800	1.02684600	-0.07044200
C	9.25885200	0.68840300	-0.09815800
C	9.45188100	-0.70420400	-0.06101500
C	8.31166600	-1.48325100	-0.00469800
S	6.91527600	-0.41667000	0.00222600
C	8.21744000	-2.91758900	0.04124100
C	9.24862600	-3.83877600	0.03147400
C	8.77279800	-5.16312900	0.08702800
C	7.40320700	-5.32329600	0.14103200
S	6.66063900	-3.73197900	0.11798700
C	6.64990300	-6.55235700	0.20533200
C	7.13416800	-7.84468700	0.17111100
C	6.09141700	-8.80123800	0.25771900
C	4.83145900	-8.28668600	0.35652100
S	4.89687900	-6.55216300	0.34890000
H	-3.43531600	-9.03158600	-0.55902200
H	-5.78337500	-10.27296800	-0.41576400
H	-9.16165400	-6.61707700	-0.04060900
H	-10.46696000	-1.80729000	0.20960600
H	-9.40205400	3.06155300	0.25656600
H	-6.21096700	6.89374800	0.13696700
H	-1.61094700	8.81109600	-0.00251300
H	3.35421800	8.38523100	-0.16237300
H	7.56405600	5.71444000	-0.20273200
H	10.06603900	1.40455500	-0.14285000
H	10.29649400	-3.58043100	-0.01292600
H	8.18044700	-8.09909100	0.08560600
H	3.89453200	-8.81618300	0.43281100

F	6.34648100	-10.12020300	0.24024500
F	9.61843500	-6.21053000	0.08838400
F	10.68545900	-1.24125100	-0.07949700
F	9.33273600	3.65088800	-0.18818300
F	5.87520000	7.37049900	-0.19834300
F	1.09342400	9.07528000	-0.06823300
F	-3.93503500	8.37755200	0.08191100
F	-8.06959300	5.43111100	0.25326000
F	-10.37577200	0.90787200	0.29376100
F	-10.33777100	-4.17073600	0.13083900
F	-7.96583100	-8.64883300	-0.19103700
H			
C	11.49143200	-5.31104200	4.74973900
C	11.30828400	-6.47395600	4.05261200
C	10.92590900	-6.25504400	2.69978000
C	10.82634800	-4.92051700	2.36536000
S	11.21720100	-3.91891000	3.75320300
C	10.48304400	-4.35482000	1.07546900
C	10.64507400	-4.92988100	-0.16879300
C	10.17250800	-4.12768700	-1.23422700
C	9.65459200	-2.91256300	-0.82894300
S	9.74008200	-2.77355900	0.91844900
C	9.12427800	-1.85656400	-1.66259000
C	9.34862800	-1.65152400	-3.01095800
C	8.65398600	-0.54154000	-3.54277300
C	7.89410500	0.14012600	-2.61048000
S	8.03552900	-0.62951100	-1.03957600
C	7.09485300	1.32583900	-2.82015800
C	7.18761900	2.23415900	-3.85846400
C	6.23465900	3.27518700	-3.79265500
C	5.39935700	3.19894200	-2.69342300
S	5.80056100	1.78871200	-1.72899200
C	4.34786200	4.11687600	-2.31954500
C	4.17519100	5.42114100	-2.74525600
C	3.02394900	6.04616500	-2.21633400
C	2.29533900	5.24158900	-1.35969100
S	3.05408400	3.66507000	-1.22308400
C	1.09319400	5.58602800	-0.63580700
C	0.61293000	6.85119200	-0.35152300
C	-0.61397600	6.85164000	0.34876500
C	-1.09535100	5.58682000	0.63272500
S	-0.00160000	4.36976100	-0.00168300
C	-2.29776900	5.24326100	1.35654900
C	-3.02601300	6.04846800	2.21292400
C	-4.17760200	5.42423200	2.74196400
C	-4.35099600	4.11992900	2.31659400
S	-3.05736600	3.66710900	1.22038200
C	-5.40306000	3.20272100	2.69057300

C	-6.23992000	3.28089700	3.78851100
C	-7.19291300	2.23996800	3.85488200
C	-7.09869800	1.32977100	2.81833400
S	-5.80288700	1.79071800	1.72816000
C	-7.89765800	0.14371400	2.60961000
C	-8.65953700	-0.53585000	3.54182400
C	-9.35319300	-1.64691700	3.01101800
C	-9.12610700	-1.85492500	1.66355800
S	-8.03591400	-0.62938400	1.04011100
C	-9.65486600	-2.91265000	0.83112300
C	-10.17398700	-4.12673700	1.23798500
C	-10.64446100	-4.93117700	0.17332700
C	-10.47958100	-4.35893400	-1.07186400
S	-9.73643700	-2.77757100	-0.91677000
C	-10.82037200	-4.92734000	-2.36122800
C	-10.91979400	-6.26256000	-2.69292200
C	-11.29946700	-6.48429000	-4.04605300
C	-11.48068100	-5.32283600	-4.74611300
S	-11.20797000	-3.92862600	-3.75207000
H	11.76832200	-5.18429100	5.78682200
H	11.42813300	-7.45724000	4.49269200
H	11.10606000	-5.90099600	-0.30601300
H	10.00802200	-2.28193000	-3.59582800
H	7.93386300	2.15394600	-4.64010900
H	4.87090300	5.91467500	-3.41359500
H	1.14197200	7.75394600	-0.63328600
H	-2.72664900	7.06032500	2.45947300
H	-6.15780000	4.06557400	4.53136300
H	-8.70521800	-0.24346500	4.58429600
H	-10.20362100	-4.43086100	2.27768900
H	-10.69765600	-7.05667500	-1.98980800
H	-11.75535500	-5.19825400	-5.78404800
H	-11.41881500	-7.46849200	-4.48421400
H	-11.10604500	-5.90184000	0.31173200
H	-10.01385100	-2.27597200	3.59591400
H	-7.94020800	2.16115500	4.63566500
H	-4.87299400	5.91835900	3.41019400
H	-1.14219000	7.75479600	0.63079400
H	2.72511300	7.05811800	-2.46312700
H	6.15137500	4.05849800	-4.53681900
H	8.69751700	-0.25143500	-4.58597500
H	10.19977800	-4.43414200	-2.27331000
H	10.70201500	-7.05061700	1.99887100
*i*Pr			
C	17.64145400	-1.20599000	1.64942000
C	17.24446600	-2.50999900	1.57750000
C	16.05850500	-2.71552500	0.79882100
C	15.57669800	-1.52888400	0.27365400

S	16.58681000	-0.16965400	0.75151600
C	14.41474600	-1.29748100	-0.57575300
C	14.00746500	-1.99433700	-1.68962500
C	12.80655100	-1.51321800	-2.29545900
C	12.30216700	-0.40518500	-1.63485400
S	13.30115400	0.01621600	-0.25194400
C	11.12263200	0.39310600	-1.94013000
C	10.68632000	0.85666300	-3.16015900
C	9.47171200	1.60663600	-3.12092700
C	8.98545500	1.73621600	-1.83000100
S	10.02091000	0.90608500	-0.67756100
C	7.79722700	2.43106200	-1.35475300
C	7.31093600	3.66566600	-1.72056200
C	6.10250900	4.05501200	-1.06686500
C	5.67299700	3.09894200	-0.16063800
S	6.75230500	1.71233800	-0.14494200
C	4.51509700	3.11323400	0.72210700
C	4.02324900	4.14562800	1.48812800
C	2.85761000	3.82854000	2.24983100
C	2.46813800	2.51062300	2.07507900
S	3.52952100	1.68137300	0.94617900
C	1.36539600	1.78237600	2.68724300
C	0.95218300	1.78542100	3.99996100
C	-0.17074600	0.94885900	4.27988900
C	-0.60784200	0.27488900	3.15116500
S	0.35601800	0.70278600	1.74548600
C	-1.69007400	-0.68993600	3.01202700
C	-2.01536300	-1.74450200	3.83406700
C	-3.14565200	-2.50950800	3.41429000
C	-3.67940200	-2.03497600	2.22712000
S	-2.79504100	-0.62859200	1.65327300
C	-4.80746200	-2.53699700	1.45471300
C	-5.12834900	-3.83765400	1.13963200
C	-6.31813800	-3.99384700	0.36554700
C	-6.90333000	-2.77482600	0.06405700
S	-5.99213900	-1.44534800	0.76446400
C	-8.10088000	-2.49048300	-0.71442600
C	-8.50571000	-3.03926300	-1.90983500
C	-9.74718700	-2.54217800	-2.41126800
C	-10.28777700	-1.57125800	-1.58411500
S	-9.26938100	-1.29971800	-0.17777200
C	-11.51562600	-0.80287000	-1.73769400
C	-12.02008900	-0.19289800	-2.86328800
C	-13.26453500	0.48316400	-2.67520600
C	-13.70435400	0.40295700	-1.36480400
S	-12.58928600	-0.53046300	-0.37906400
C	-14.89955600	0.96980500	-0.75208700
C	-15.42527900	2.23362600	-0.88558900

C	-16.62645500	2.46163500	-0.13818600
C	-16.99320400	1.34573900	0.56893500
S	-15.90620600	0.01634400	0.32780900
H	18.49062700	-0.79295700	2.17590900
H	17.77439800	-3.31313400	2.07727800
H	14.57531300	-2.82977300	-2.08207500
H	11.24296500	0.67954700	-4.07286400
H	7.82729800	4.29792100	-2.43293900
H	4.50809300	5.11395100	1.52487700
H	1.46534600	2.36084900	4.76139200
H	-1.43425200	-1.98393600	4.71677200
H	-4.50440100	-4.67128600	1.43939600
H	-7.90698400	-3.77372800	-2.43551100
H	-11.49269200	-0.20803600	-3.80975400
H	-14.94615800	2.99980900	-1.48493600
H	-17.84495500	1.22015300	1.22184900
C	12.17187600	-2.17514300	-3.50405100
H	11.19261800	-1.70914200	-3.65514300
C	11.93536000	-3.67893300	-3.26961700
H	12.87862600	-4.22083300	-3.14076900
H	11.32550200	-3.84909800	-2.37747700
H	11.41608400	-4.12137100	-4.12646500
C	15.40894500	-4.07546000	0.62617500
H	14.44951600	-3.91879400	0.12226000
C	15.11483600	-4.74043800	1.98412300
H	14.58675300	-5.68881900	1.83810300
H	16.03681100	-4.95806800	2.53432900
H	14.49251400	-4.09745700	2.61335800
C	8.80607700	2.15958500	-4.36708000
H	7.82094700	2.53618500	-4.07267300
C	8.58241100	1.06433500	-5.42657700
H	9.53129800	0.66306100	-5.79892300
H	8.00181900	0.23151100	-5.01897300
H	8.03775500	1.47083700	-6.28546000
C	5.38705600	5.35845200	-1.36895700
H	4.41425500	5.32131000	-0.86774500
C	5.12216600	5.52602100	-2.87696800
H	6.05582800	5.60403100	-3.44469500
H	4.55589500	4.67932900	-3.27610100
H	4.54652600	6.43887900	-3.06356100
C	2.14113300	4.84506400	3.11898000
H	1.20934900	4.38368900	3.46215700
C	1.76313100	6.10719500	2.32111800
H	2.65142700	6.64180400	1.96732000
H	1.15241700	5.85599600	1.44889400
H	1.19134900	6.79854000	2.94938500
C	-0.79728300	0.84657800	5.65785900
H	-1.72190500	0.26916500	5.55511000

C	-1.17763700	2.23175200	6.21349800
H	-0.29384600	2.85931000	6.37213600
H	-1.84782800	2.76170500	5.53022900
H	-1.68608700	2.12922300	7.17809000
C	-3.68458800	-3.68369900	4.20996900
H	-4.63205300	-3.98409200	3.75062300
C	-3.98352300	-3.29176800	5.66921500
H	-3.07177900	-3.00474600	6.20452800
H	-4.68116900	-2.45051400	5.71862900
H	-4.42902500	-4.13563600	6.20677100
C	-6.86226500	-5.35019200	-0.04227800
H	-7.85571300	-5.18801900	-0.47321500
C	-7.03274000	-6.28261900	1.17165800
H	-6.06982600	-6.51665200	1.63874900
H	-7.67415100	-5.82873400	1.93288600
H	-7.48784300	-7.23004100	0.86363200
C	-10.38283500	-3.05399400	-3.69020500
H	-11.38874500	-2.62553100	-3.74966900
C	-10.53567100	-4.58636600	-3.67640800
H	-9.56264300	-5.08916900	-3.65270600
H	-11.10759100	-4.91950300	-2.80531200
H	-11.05878500	-4.92506100	-4.57707700
C	-14.00289300	1.17944500	-3.80274500
H	-14.98378900	1.47529200	-3.41648800
C	-14.24216300	0.23345800	-4.99452300
H	-13.29941200	-0.08186400	-5.45485100
H	-14.78130200	-0.66626300	-4.68344300
H	-14.83412600	0.73521300	-5.76748500
C	-17.34503000	3.79898500	-0.12616900
H	-17.42598700	4.12668800	-1.17286100
C	-16.51605900	4.86082700	0.62497400
H	-16.41167100	4.59008300	1.68114100
H	-15.50992600	4.95788500	0.20623100
H	-17.00149200	5.84129700	0.57038300
C	16.26303600	-4.99954400	-0.26377900
H	15.76539200	-5.96469200	-0.40886300
H	16.44076300	-4.55543300	-1.24838200
H	17.24085700	-5.19337600	0.19128200
C	13.00592800	-1.94174500	-4.77894400
H	12.51393200	-2.38837600	-5.64993100
H	13.14648900	-0.87422900	-4.97592100
H	14.00039000	-2.39298000	-4.68949200
C	9.60482300	3.33870400	-4.95673600
H	9.09370400	3.75271200	-5.83281900
H	9.73309400	4.14220800	-4.22451800
H	10.60425300	3.01950000	-5.27270400
C	6.16033600	6.56871600	-0.80928300
H	5.61431500	7.49896200	-1.00077900

H	6.31680700	6.48021300	0.27045400
H	7.14692600	6.65786500	-1.27753700
C	2.97312900	5.21028100	4.36374200
H	2.42528400	5.91138900	5.00275300
H	3.21551200	4.32362100	4.95802100
H	3.91914300	5.68545300	4.08130900
C	0.12168200	0.09353400	6.63980200
H	-0.35881600	-0.00741800	7.61911300
H	0.36434900	-0.90875000	6.27286000
H	1.06710900	0.62780800	6.78519900
C	-2.72799600	-4.89082300	4.15085800
H	-3.14749100	-5.74350300	4.69595400
H	-2.54022000	-5.20351300	3.11883500
H	-1.75939800	-4.64966200	4.60263500
C	-5.98199100	-6.00972300	-1.12208200
H	-6.40655700	-6.97045900	-1.43325400
H	-5.89013300	-5.37330700	-2.00788200
H	-4.97004200	-6.19774000	-0.74645600
C	-9.59894700	-2.59086000	-4.93387200
H	-10.09137900	-2.93245000	-5.85094500
H	-9.51997300	-1.49987200	-4.97400400
H	-8.58023700	-2.99435600	-4.93165300
C	-13.26912700	2.45739400	-4.25386400
H	-13.83518200	2.97256900	-5.03773100
H	-13.12798500	3.15307100	-3.42075600
H	-12.27806800	2.22248800	-4.65791000
C	-18.76634300	3.71570400	0.44488600
H	-19.26648600	4.68604100	0.36715200
H	-19.37216200	2.97767500	-0.08979500
H	-18.75202600	3.43942800	1.50536200
Li			
C	-18.93130400	-1.55077100	-0.02900400
C	-18.43661900	-2.61029100	0.68683200
C	-17.14912500	-2.40931400	1.29071400
C	-16.71151400	-1.12550700	0.99327100
S	-17.84706600	-0.19507800	-0.02277800
C	-15.43286800	-0.56761000	1.42662400
C	-14.81051500	-0.77959300	2.66128700
C	-13.47687100	-0.27357000	2.81601000
C	-13.11128400	0.37477200	1.64495100
S	-14.35656100	0.32147800	0.36638300
C	-11.83294600	1.05801700	1.44816200
C	-11.16480700	1.84903800	2.38774900
C	-9.83734500	2.27829800	2.05399300
C	-9.52762900	1.80721500	0.78645400
S	-10.81601400	0.82357000	0.04078600
C	-8.26938400	2.08712400	0.09260500
C	-7.59875600	3.31285200	0.06315600

C	-6.28668100	3.32469200	-0.51638100
C	-5.99357000	2.04641700	-0.96823400
S	-7.27848000	0.84995000	-0.65144400
C	-4.75335500	1.68385600	-1.65688300
C	-4.10544200	2.43662800	-2.63976200
C	-2.80479900	1.99187600	-3.04952000
C	-2.49798900	0.83088900	-2.35543800
S	-3.75580200	0.32089600	-1.19758200
C	-1.26534900	0.06323400	-2.54215000
C	-0.64680800	-0.22079400	-3.76248300
C	0.65057300	-0.83021100	-3.70884700
C	0.98539300	-1.03532800	-2.37876500
S	-0.24216600	-0.45658900	-1.22089200
C	2.22150700	-1.67995600	-1.93156100
C	2.81549800	-2.81017200	-2.49911900
C	4.11775200	-3.17189700	-2.01894300
C	4.48237400	-2.27928200	-1.02220000
S	3.27637800	-1.00198300	-0.71103400
C	5.73082100	-2.35972200	-0.26223100
C	6.32202800	-3.52469100	0.23366600
C	7.63306000	-3.39864700	0.80132500
C	8.00825100	-2.06464400	0.74967900
S	6.80103400	-0.99736800	-0.01642300
C	9.26537300	-1.54260000	1.28706500
C	9.86643500	-1.91767200	2.49155500
C	11.18045400	-1.40497000	2.75069700
C	11.54816700	-0.58376900	1.69577700
S	10.33018200	-0.47759200	0.39612400
C	12.80571800	0.16194200	1.63417300
C	13.41727900	0.83792700	2.69280000
C	14.72948400	1.36711800	2.45544700
C	15.08328100	1.09379000	1.14371100
S	13.85679900	0.17051600	0.23470800
C	16.33207800	1.53625300	0.51679300
C	16.90953700	2.80064800	0.62202100
C	18.22543100	2.96825900	0.04465400
C	18.60249900	1.76643700	-0.51125400
S	17.42563700	0.46831600	-0.33397200
H	-19.87264800	-1.47967500	-0.56044200
H	-18.99732200	-3.53832200	0.77181400
H	-15.38800100	-1.20519100	3.48411300
H	-11.70405700	2.20001600	3.27018200
H	-8.12570800	4.21770500	0.37380700
H	-4.64208700	3.25285000	-3.12856700
H	-1.20367700	-0.07656700	-4.69112300
H	2.23718900	-3.42939800	-3.18871400
H	5.73596600	-4.44557700	0.27788800
H	9.28526900	-2.47543900	3.22944400

H	12.84478700	1.04267700	3.60030800
H	16.31465800	3.62548900	1.01843900
H	19.50996300	1.52148600	-1.05351400
Li	19.32532900	4.56483500	-0.31075300
Li	16.51864200	2.12406600	2.77545700
Li	12.95249600	-1.18473000	3.59269200
Li	9.40271300	-3.90198700	1.51825800
Li	5.88204400	-4.05230500	-1.91582700
Li	2.42371000	-1.48446800	-4.28079400
Li	-1.01125200	1.99999600	-3.87322800
Li	-4.47227500	3.93195200	-0.99750100
Li	-8.01124600	2.99411300	2.25101400
Li	-11.65898800	-0.02345100	3.52597300
Li	-15.35970500	-2.84986600	1.96269600
*n*Bu			
C	-18.42742600	0.86743600	1.64994800
C	-18.05844300	2.17558900	1.52525500
C	-16.85889800	2.37220300	0.76557200
C	-16.33774500	1.17487600	0.30781200
S	-17.32772300	-0.18424600	0.82646000
C	-15.15706600	0.93453200	-0.51284700
C	-14.76137600	1.57819700	-1.66203500
C	-13.54153800	1.09902100	-2.23051900
C	-13.01058900	0.04629100	-1.50505400
S	-14.00756400	-0.32415200	-0.10607600
C	-11.81339100	-0.74196300	-1.76674800
C	-11.39338200	-1.30045700	-2.95166800
C	-10.16142900	-2.01866900	-2.87539700
C	-9.64505000	-2.02645000	-1.59027400
S	-10.67352900	-1.12002400	-0.49039000
C	-8.43717900	-2.66477700	-1.08515000
C	-7.96270600	-3.93232800	-1.33311300
C	-6.73109800	-4.25190500	-0.68481900
C	-6.27220200	-3.20631500	0.09960700
S	-7.35291200	-1.82363100	0.00504900
C	-5.08866600	-3.13506200	0.94444800
C	-4.58108400	-4.08386800	1.80255400
C	-3.39367900	-3.69573200	2.49434900
C	-3.00419000	-2.40630800	2.17252200
S	-4.09214700	-1.69389800	0.99043300
C	-1.88834500	-1.62322400	2.68559600
C	-1.46565100	-1.47638200	3.98672100
C	-0.33460600	-0.62149500	4.15867300
C	0.09832400	-0.08654300	2.95779900
S	-0.87823500	-0.66816000	1.61769000
C	1.18317900	0.85381400	2.70573400
C	1.46807300	2.03106100	3.35734800
C	2.61233600	2.72937200	2.86422000

C	3.19712700	2.07466900	1.79377200
S	2.34437700	0.58432600	1.42110000
C	4.35113400	2.46657200	0.99441900
C	4.63523100	3.69138400	0.43686700
C	5.86557100	3.75238100	-0.28617100
C	6.52062200	2.53278800	-0.29739000
S	5.62313300	1.32422600	0.60926000
C	7.77675400	2.16600600	-0.93868300
C	8.20465700	2.43739900	-2.21756900
C	9.50333900	1.93515500	-2.53621200
C	10.06559000	1.24311200	-1.47707200
S	8.99559100	1.24162300	-0.08346800
C	11.34846600	0.55605700	-1.40010400
C	11.91954600	-0.30907200	-2.30415900
C	13.20201100	-0.81283800	-1.92613500
C	13.60373300	-0.33456300	-0.69137400
S	12.40465300	0.75667500	-0.01541400
C	14.81791400	-0.62731900	0.06159400
C	15.38927400	-1.84750100	0.33801100
C	16.58951800	-1.77596000	1.11616800
C	16.90867000	-0.48039200	1.42928800
S	15.77775200	0.66016400	0.77659000
H	-19.28102600	0.45940400	2.17314000
H	-18.61914400	2.99152900	1.96811800
H	-15.35160300	2.36887200	-2.11122800
H	-11.97495900	-1.22162100	-3.86304200
H	-8.50465100	-4.63843500	-1.95136800
H	-5.06939100	-5.03839800	1.96085600
H	-1.97737500	-1.94943000	4.81719600
H	0.84904400	2.40803200	4.16358800
H	3.95776500	4.53349100	0.52169400
H	7.58524900	2.96746600	-2.93215500
H	11.41573600	-0.60494700	-3.21742100
H	14.93653700	-2.78210400	0.02577000
H	17.74723000	-0.13075400	2.01656700
C	17.39545100	-2.98119800	1.53294500
C	18.13324100	-3.66451400	0.36562800
H	16.73285900	-3.71446300	2.01307900
H	18.12498700	-2.68310200	2.29531100
C	18.94259200	-4.89140100	0.80180200
H	17.40510800	-3.96069100	-0.40152500
H	18.79806600	-2.93377000	-0.11348400
C	19.67761200	-5.57113200	-0.35727000
H	19.66820100	-4.59104700	1.56996600
H	18.27205200	-5.61471300	1.28554100
H	20.24762500	-6.44061800	-0.01445100
H	18.97521000	-5.91574600	-1.12459400
H	20.37997300	-4.88194300	-0.83950900

C	14.01104000	-1.72306000	-2.81886400
C	14.37643500	-1.08628300	-4.17383400
H	13.44775400	-2.64930500	-3.00145600
H	14.93048100	-2.01706400	-2.30511700
C	15.19235300	-2.02304400	-5.07265500
H	13.46168200	-0.78365700	-4.70070900
H	14.94272100	-0.16349100	-3.99240500
C	15.56610600	-1.39203300	-6.41717500
H	16.10589500	-2.32593300	-4.54342900
H	14.62236300	-2.94614200	-5.24620500
H	16.14692200	-2.08348000	-7.03610300
H	14.67228300	-1.10875100	-6.98455900
H	16.16728300	-0.48685000	-6.27582000
C	10.16768200	2.18929200	-3.86826700
C	10.35179900	3.68558000	-4.18752500
H	9.57191400	1.72588100	-4.66773900
H	11.14494500	1.69904700	-3.88890400
C	11.02914700	3.92694500	-5.54191900
H	9.37721700	4.19131100	-4.17485300
H	10.94633800	4.14969100	-3.38988300
C	11.21529800	5.41259200	-5.86407500
H	12.00549000	3.42411700	-5.55208200
H	10.43472300	3.45133500	-6.33414100
H	11.69776000	5.55396100	-6.83656000
H	10.25260700	5.93565600	-5.89127700
H	11.83738600	5.90647300	-5.10922600
C	6.37846300	5.02991200	-0.90690100
C	6.58805500	6.16560700	0.11374900
H	5.67269900	5.37051600	-1.67799400
H	7.32457400	4.83443000	-1.41917500
C	7.10634000	7.45770600	-0.52842800
H	5.64484100	6.37380400	0.63614600
H	7.29404300	5.82693600	0.88312900
C	7.31980600	8.58792600	0.48297100
H	8.05076100	7.24934400	-1.04902700
H	6.39949700	7.78679900	-1.30252100
H	7.68938600	9.49596100	-0.00424000
H	6.38556200	8.84288200	0.99611200
H	8.04905200	8.30110500	1.24882300
C	3.11814600	4.00432200	3.49606700
C	3.51867500	3.83906600	4.97507100
H	2.34309200	4.78048800	3.42189700
H	3.98025900	4.37482200	2.93474900
C	4.03147200	5.14108800	5.60202700
H	2.66091200	3.47115100	5.55361000
H	4.29223900	3.06381000	5.05000900
C	4.43488700	4.98339200	7.07099200
H	4.89040400	5.50641600	5.02322200

H	3.25607400	5.91466300	5.51550500
H	4.79545300	5.92823500	7.49024200
H	3.58767900	4.65088600	7.68155700
H	5.23417900	4.24258800	7.18498100
C	0.31114600	-0.39711500	5.50510100
C	0.89144400	-1.68366400	6.12454800
H	-0.42903000	0.02930700	6.19692700
H	1.11045700	0.34305100	5.41217100
C	1.52868600	-1.44975400	7.49928000
H	0.10088800	-2.44061800	6.21398000
H	1.63837500	-2.10310100	5.43813800
C	2.12053600	-2.72235600	8.11234000
H	2.31469900	-0.68794800	7.40822100
H	0.77632500	-1.02933300	8.18063800
H	2.56832700	-2.52349700	9.09134400
H	1.35248900	-3.49220100	8.24859900
H	2.90070700	-3.14512400	7.46952500
C	-2.65618700	-4.62637500	3.42765900
C	-2.22469500	-5.94898300	2.76552900
H	-3.29174700	-4.85399700	4.29567700
H	-1.76930800	-4.12108000	3.82002300
C	-1.46704500	-6.87544300	3.72412700
H	-3.10576900	-6.47517600	2.37495800
H	-1.59416000	-5.72334500	1.89569300
C	-1.03381200	-8.19152100	3.07173700
H	-0.58348100	-6.35009500	4.11092300
H	-2.09911300	-7.08896100	4.59710100
H	-0.49430700	-8.82983700	3.77879600
H	-1.89868700	-8.75703500	2.70660000
H	-0.37339800	-8.01043400	2.21628900
C	-6.02499200	-5.57194400	-0.88609800
C	-5.75682600	-5.91297800	-2.36443400
H	-6.62304000	-6.37922100	-0.43861000
H	-5.07326100	-5.56159500	-0.34751600
C	-5.03322800	-7.25294300	-2.54339800
H	-6.70358500	-5.93709600	-2.91997000
H	-5.16005300	-5.10868000	-2.81397700
C	-4.75989600	-7.59811700	-4.01025600
H	-4.08464500	-7.22805400	-1.99025100
H	-5.63126800	-8.05179600	-2.08369100
H	-4.24052900	-8.55713400	-4.10510100
H	-5.69213700	-7.66710200	-4.58227000
H	-4.13628700	-6.83368800	-4.48718300
C	-9.51279700	-2.64735000	-4.08562200
C	-9.21955000	-1.64760500	-5.22093800
H	-10.16190700	-3.44519900	-4.47455200
H	-8.57721500	-3.12973300	-3.78959800
C	-8.56655900	-2.30997600	-6.44001600

H	-10.14923700	-1.15334400	-5.53242300
H	-8.56567700	-0.85352200	-4.83779400
C	-8.26520200	-1.32359600	-7.57218300
H	-7.63671300	-2.80397500	-6.12752900
H	-9.22319100	-3.10789100	-6.81317500
H	-7.80122700	-1.82578400	-8.42718000
H	-9.18035100	-0.83731600	-7.92891700
H	-7.58150000	-0.53506900	-7.23849700
C	-12.91605200	1.72508100	-3.45402700
C	-12.58099400	3.21874500	-3.27639800
H	-13.59653900	1.61266300	-4.31032000
H	-12.00142000	1.18530200	-3.71441000
C	-11.94545100	3.83918400	-4.52619400
H	-13.49119700	3.77591300	-3.01768500
H	-11.90114800	3.33221200	-2.42201400
C	-11.60654200	5.32291600	-4.35541800
H	-11.03393800	3.28231200	-4.78189700
H	-12.62649100	3.71410700	-5.37928200
H	-11.15216000	5.73612800	-5.26167700
H	-12.50394100	5.91227000	-4.13528500
H	-10.90148100	5.47557600	-3.53063600
C	-16.23684300	3.73330000	0.56263900
C	-15.89033200	4.45418000	1.87958400
H	-16.92406900	4.36573100	-0.01789200
H	-15.32846200	3.63456800	-0.03848300
C	-15.26711800	5.83743200	1.65636500
H	-16.79361300	4.55960900	2.49484000
H	-15.19987800	3.82728600	2.45848200
C	-14.92307500	6.55928500	2.96252400
H	-14.36019400	5.73125600	1.04606800
H	-15.95854500	6.45537400	1.06697500
H	-14.48217100	7.54304900	2.77193600
H	-15.81639600	6.70893000	3.57954200
H	-14.20540500	5.98251800	3.55659800
NH_2			
C	14.99189800	-2.26134800	4.27799500
C	14.47906400	-3.48895700	4.58231100
C	13.50738600	-3.94387300	3.63404900
C	13.30172300	-3.03529400	2.60367000
S	14.32648600	-1.61930700	2.81650500
C	12.39380200	-3.16333400	1.48579500
C	12.01203500	-4.31554200	0.82864100
C	11.08145700	-4.09947100	-0.23060400
C	10.75196400	-2.75951000	-0.39697000
S	11.59214800	-1.76663500	0.79139100
C	9.85291500	-2.18864500	-1.37038900
C	9.57266200	-2.64514000	-2.64423200
C	8.61692800	-1.85523100	-3.34757500

C	8.16248200	-0.76795300	-2.60966500
S	8.91846000	-0.74559500	-1.01826800
C	7.20555200	0.23211000	-3.01448700
C	6.94994600	0.70374500	-4.28835800
C	5.92005400	1.68713400	-4.34851900
C	5.38121500	1.98695300	-3.10212500
S	6.15265700	1.01651100	-1.84964800
C	4.33317100	2.92998700	-2.80043100
C	4.01188600	4.09510600	-3.47157600
C	2.89692700	4.79331100	-2.92350000
C	2.35756100	4.16529800	-1.80614400
S	3.23645800	2.67931500	-1.45282900
C	1.23693000	4.59740800	-1.00910600
C	0.79396500	5.88777100	-0.78455500
C	-0.36395200	5.96942500	0.04199700
C	-0.81452300	4.72606400	0.47211100
S	0.20388700	3.44458100	-0.18112600
C	-1.94622000	4.44336000	1.31875700
C	-2.49946300	5.24107700	2.30342800
C	-3.63340200	4.66705800	2.94786200
C	-3.95329100	3.40345500	2.46368400
S	-2.84236000	2.94042600	1.17613400
C	-5.02215000	2.53833600	2.89627700
C	-5.60670100	2.45995800	4.14680300
C	-6.65516300	1.49925200	4.23923700
C	-6.87650700	0.81826900	3.04747400
S	-5.78092000	1.40044700	1.79567600
C	-7.84106700	-0.22351500	2.79517000
C	-8.35097400	-1.14885700	3.68656900
C	-9.30527300	-2.04098000	3.11643900
C	-9.52730000	-1.80881100	1.76425100
S	-8.55192400	-0.45124500	1.20623600
C	-10.41267500	-2.52874300	0.88137000
C	-10.78662400	-3.85738600	0.94528900
C	-11.69720200	-4.25522600	-0.07806400
C	-12.02214200	-3.22299000	-0.94730500
S	-11.19764600	-1.73928600	-0.47638200
C	-12.90311100	-3.27932600	-2.09241500
C	-13.13086700	-4.34225000	-2.94241800
C	-14.09094700	-4.06801500	-3.97033600
C	-14.58648600	-2.78989000	-3.89301200
S	-13.89675200	-1.90700100	-2.55998200
H	15.73326300	-1.69846400	4.82744900
H	14.77321800	-4.06463100	5.45330100
H	12.43792600	-5.28733800	1.05282500
H	10.09290100	-3.48298100	-3.09489100
H	7.53607000	0.40933100	-5.15173300
H	4.60546500	4.48281400	-4.29191400

H	1.32832200	6.76187100	-1.13942100
H	-2.05883700	6.18214100	2.61287300
H	-5.24295200	3.01963800	5.00118900
H	-7.99423100	-1.23913600	4.70649400
H	-10.36071300	-4.55300700	1.65975700
H	-12.56685600	-5.26690900	-2.88918300
H	-15.29598800	-2.30958600	-4.55191600
N	-14.38885200	-4.99972700	-4.97685100
H	-14.39040600	-5.95776900	-4.64948600
H	-15.24806800	-4.79965500	-5.47285700
N	-12.16173800	-5.56923200	-0.19723800
H	-12.22353700	-6.05301600	0.69010600
H	-13.03454600	-5.63686200	-0.70943700
N	-9.90895400	-3.06334200	3.85455800
H	-9.98628900	-2.85174500	4.84163100
H	-10.80262600	-3.35573200	3.47470300
N	-7.34549900	1.25619900	5.42999200
H	-7.37728900	2.05906000	6.04593300
H	-8.27301000	0.87340100	5.28374100
N	-4.31116300	5.31249400	3.98562300
H	-4.24038200	6.32150600	3.94361400
H	-5.27768900	5.01970600	4.07666100
N	-0.94363000	7.18917400	0.40047000
H	-0.78050400	7.92472600	-0.27569100
H	-1.92948300	7.11463800	0.62572800
N	2.43835200	6.00297700	-3.45122300
H	2.63936400	6.11198100	-4.43740700
H	1.45883500	6.18018100	-3.25794700
N	5.53852800	2.29943400	-5.54479500
H	5.69834400	1.72168700	-6.36056600
H	4.58853700	2.65361300	-5.52992600
N	8.22552500	-2.14297700	-4.65735000
H	8.29816000	-3.12501400	-4.89217600
H	7.30839100	-1.77950600	-4.89162600
N	10.59950400	-5.14015200	-1.02798800
H	10.58226700	-6.03483800	-0.55462300
H	9.70280700	-4.93685000	-1.45552700
N	12.86781800	-5.18128100	3.74641100
H	12.77526100	-5.49861900	4.70360700
H	11.97425200	-5.21373500	3.26780000
NMe_2			
C	-9.23698200	-4.49794000	-6.81060800
C	-9.26869600	-5.79586900	-6.38905900
C	-9.13807700	-5.93976100	-4.96851200
C	-9.01786100	-4.71049100	-4.32110100
S	-9.04651300	-3.39928200	-5.49015400
C	-8.95482200	-4.42537700	-2.90257400
C	-9.37443200	-5.21010600	-1.84851800

C	-9.19384300	-4.61776200	-0.56514700
C	-8.62891100	-3.34221200	-0.63293300
S	-8.28937400	-2.91808800	-2.30302500
C	-8.37989700	-2.39481700	0.42835300
C	-8.91958000	-2.36343800	1.69987700
C	-8.48718000	-1.26572800	2.49647900
C	-7.59646800	-0.42528700	1.82335500
S	-7.28022800	-1.04531800	0.21042600
C	-7.01463100	0.82358900	2.25370000
C	-7.43243500	1.66643700	3.26615000
C	-6.64829000	2.84511400	3.41223000
C	-5.60270600	2.91958000	2.48791900
S	-5.59158900	1.48754700	1.47054100
C	-4.65488300	3.98436500	2.26294000
C	-4.73375100	5.30756000	2.65435400
C	-3.63119000	6.10922100	2.24609800
C	-2.67925800	5.39234200	1.51582400
S	-3.16108100	3.70787800	1.38453100
C	-1.47858600	5.86228500	0.86814800
C	-1.12578800	7.15642000	0.53412300
C	0.13003600	7.27280600	-0.12477100
C	0.76187700	6.04051700	-0.31289400
S	-0.21099400	4.75130900	0.37849000
C	1.98874200	5.73860100	-1.00959500
C	2.68402500	6.51978400	-1.91372700
C	3.85339300	5.90687500	-2.44495700
C	4.06533700	4.61818400	-1.94694100
S	2.81453600	4.20667300	-0.78356300
C	5.07580400	3.65261800	-2.30561300
C	5.88185000	3.62156400	-3.42817900
C	6.76128400	2.50433200	-3.49119700
C	6.62885100	1.64604200	-2.39658300
S	5.42900900	2.27451500	-1.27769600
C	7.27026700	0.37777000	-2.14521900
C	7.90692500	-0.46077400	-3.04023000
C	8.40894000	-1.66287300	-2.46641600
C	8.15181800	-1.76052800	-1.09707500
S	7.31436600	-0.32159700	-0.53591800
C	8.42383200	-2.85035800	-0.18918300
C	8.70081500	-4.17125000	-0.48313400
C	8.88668700	-5.00293500	0.65916400
C	8.74983700	-4.30950000	1.86151700
S	8.41988300	-2.61393200	1.54965900
C	8.78765000	-4.80703900	3.22171600
C	8.55985900	-6.08963800	3.66810400
C	8.62189100	-6.23188300	5.09494100
C	8.91197200	-5.03656600	5.71751300
S	9.10106500	-3.73564700	4.57991700

H	-9.29603400	-4.12258600	-7.82242000
H	-9.36702900	-6.63339200	-7.06823400
H	-9.80980600	-6.18620000	-2.01265500
H	-9.62285700	-3.11558900	2.02921600
H	-8.29710700	1.43191100	3.87104100
H	-5.58354800	5.67817900	3.21034400
H	-1.77831000	7.99007600	0.75253900
H	2.33347400	7.50453700	-2.18909800
H	5.81464400	4.38657900	-4.18879600
H	7.98543000	-0.20710900	-4.08825500
H	8.74537900	-4.52117500	-1.50494800
H	8.30861800	-6.88558800	2.98043500
H	9.03587800	-4.83783400	6.77038300
N	8.34938400	-7.44341700	5.75326700
N	9.17848000	-6.39063400	0.58944300
N	9.10403100	-2.66240400	-3.19720400
N	7.68298600	2.27829600	-4.54656300
N	4.72243800	6.53007800	-3.37759100
N	0.69580900	8.50507300	-0.54439600
N	-3.49778300	7.48966800	2.54785200
N	-6.89099700	3.84356200	4.39053900
N	-8.90664300	-1.04034900	3.83268300
N	-9.54157500	-5.24751600	0.65738500
N	-9.12284700	-7.18558000	-4.29266200
C	8.40481000	-7.40416800	7.20343000
H	7.74494300	-6.61612700	7.57747100
H	8.05303800	-8.35982800	7.60165700
H	9.42048400	-7.22029500	7.59789600
C	8.93511400	-8.65078200	5.18184200
H	8.74027400	-8.70126700	4.10977300
H	10.02634200	-8.71541800	5.33533600
H	8.47186900	-9.52685600	5.64585800
C	10.52939700	-6.75253400	1.02475700
H	11.30569200	-6.40399800	0.32225500
H	10.73359400	-6.32944000	2.00909000
H	10.60278200	-7.84265800	1.10048400
C	8.80922900	-7.04692000	-0.65735600
H	7.77672700	-6.80101900	-0.91660800
H	9.45636000	-6.77546400	-1.51068000
H	8.87969900	-8.13082400	-0.52068500
C	10.53706000	-2.73965000	-2.90069300
H	11.09216100	-1.86483900	-3.28008000
H	10.69440500	-2.81194600	-1.82365600
H	10.95418400	-3.63814500	-3.36762200
C	8.83792200	-2.68890000	-4.62943100
H	7.76000200	-2.68285900	-4.80760800
H	9.28672200	-1.84294200	-5.18007000
H	9.25013600	-3.61225800	-5.04887000

C	9.08885600	2.45564000	-4.17196900
H	9.34188300	3.51235200	-3.98114300
H	9.31620000	1.87587400	-3.27647500
H	9.72655000	2.09232400	-4.98467500
C	7.36593500	2.93620400	-5.80738500
H	6.32777800	2.73503400	-6.08198700
H	7.51499900	4.03051200	-5.78565200
H	8.01349800	2.52964900	-6.59077100
C	6.03057100	6.90386800	-2.83227700
H	5.96343500	7.74267000	-2.11886100
H	6.48526200	6.05159800	-2.32570100
H	6.69044000	7.20465700	-3.65285300
C	4.13103300	7.60353600	-4.16552000
H	3.18634200	7.26942500	-4.60087900
H	3.94193000	8.52485100	-3.58648400
H	4.81368100	7.86014600	-4.98187700
C	1.85381300	8.93716900	0.24350500
H	1.57446500	9.24418100	1.26563900
H	2.58591600	8.13125700	0.30960400
H	2.32904600	9.79034000	-0.25201000
C	-0.25373200	9.58891000	-0.76171000
H	-1.07577400	9.24250800	-1.39246600
H	-0.67931400	9.99920600	0.17134800
H	0.25635700	10.40695900	-1.28030400
C	-2.45865700	7.79582500	3.53541100
H	-2.72816100	7.45195600	4.54842200
H	-1.51624700	7.32758800	3.24827800
H	-2.30424800	8.87935100	3.57175700
C	-4.74321800	8.19236100	2.82871400
H	-5.47413500	7.98241500	2.04431800
H	-5.19075300	7.92742900	3.80303900
H	-4.54722400	9.26930400	2.83652800
C	-5.88960700	3.90526800	5.45914100
H	-5.94115900	3.03346500	6.13314500
H	-4.88668200	3.95758600	5.03362800
H	-6.05472100	4.80882800	6.05530900
C	-8.25096000	3.89475100	4.91118100
H	-8.96619600	3.90462900	4.08537700
H	-8.50155300	3.05182500	5.57957400
H	-8.37509200	4.81926600	5.48396500
C	-7.86411600	-1.25058200	4.84163100
H	-7.60268200	-2.31592300	4.95781000
H	-6.96280000	-0.69810900	4.57353100
H	-8.21791800	-0.87761200	5.80846900
C	-10.16647100	-1.66830600	4.20902900
H	-10.93161500	-1.44662400	3.46135300
H	-10.09762300	-2.76467600	4.32329500
H	-10.49440800	-1.25579500	5.16847400

C	-8.39669300	-5.66412400	1.47285500
H	-7.85491400	-6.51641900	1.02934200
H	-7.69996000	-4.83395700	1.59531200
H	-8.75055000	-5.96238800	2.46525400
C	-10.55436900	-6.29065600	0.56091300
H	-11.41340800	-5.92446200	-0.00625600
H	-10.19116100	-7.21842300	0.08419400
H	-10.89357200	-6.54827400	1.56924500
C	-7.83281500	-7.55020000	-3.69919800
H	-7.07915100	-7.80967600	-4.46137400
H	-7.44919600	-6.72700600	-3.09565800
H	-7.97055100	-8.41725700	-3.04489400
C	-9.73125000	-8.29800400	-5.00993000
H	-9.85847200	-9.13700600	-4.31871300
H	-10.71783800	-8.00868800	-5.38009800
H	-9.12879200	-8.65835400	-5.86286400
NO_2			
C	15.75631300	-1.43111800	1.40965000
C	15.49769100	-2.72708700	1.08553500
C	14.44185400	-2.84267300	0.13516200
C	13.88256600	-1.63602000	-0.26779800
S	14.71940900	-0.33680500	0.55719200
C	12.78806100	-1.28651800	-1.16280600
C	12.44772600	-1.78133900	-2.39624900
C	11.35508400	-1.09575300	-2.98453300
C	10.81668200	-0.06555600	-2.21985200
S	11.72627600	0.04787900	-0.73148800
C	9.69070800	0.83945800	-2.39585800
C	9.25351100	1.52404100	-3.50252900
C	8.16782600	2.39101100	-3.22282800
C	7.72889700	2.39032500	-1.90214100
S	8.72666400	1.28171500	-0.99122700
C	6.64223100	3.05530400	-1.19929300
C	6.17263900	4.34260000	-1.28454600
C	5.15210500	4.62213900	-0.34182800
C	4.79701500	3.56045000	0.48531800
S	5.79212500	2.18438400	0.07255600
C	3.79366900	3.38103700	1.52340000
C	3.38599400	4.21926900	2.53129700
C	2.44656000	3.61743000	3.40503900
C	2.09339500	2.30784400	3.09085100
S	2.98739900	1.82309100	1.66923700
C	1.15347400	1.35170400	3.65502100
C	0.84797400	1.05693100	4.96070200
C	-0.05840500	-0.02500100	5.08630800
C	-0.48631100	-0.58728000	3.88640400
S	0.29329000	0.26192900	2.57235800
C	-1.42465800	-1.64424100	3.54305800

C	-1.66086300	-2.86128000	4.13333800
C	-2.59504200	-3.65116100	3.41850500
C	-3.11434400	-3.06522900	2.26697900
S	-2.38716100	-1.48706300	2.07699300
C	-4.10871100	-3.47206700	1.28685400
C	-4.34706800	-4.69287300	0.70545300
C	-5.35296200	-4.64432800	-0.29127200
C	-5.92688100	-3.39270200	-0.49947400
S	-5.15904300	-2.24966400	0.57760500
C	-6.99846400	-2.90095100	-1.35061700
C	-7.32288900	-3.20308800	-2.65019300
C	-8.38989100	-2.41686900	-3.15141800
C	-8.92723900	-1.49752700	-2.25389200
S	-8.04562900	-1.61909900	-0.74914600
C	-10.03291700	-0.55499100	-2.31204800
C	-10.45319100	0.27361800	-3.32295000
C	-11.51858300	1.12350700	-2.93394400
C	-11.96048900	0.96584900	-1.62341400
S	-10.98794800	-0.26882500	-0.85978300
C	-13.03474500	1.55820500	-0.84064800
C	-13.47281100	2.85998200	-0.76203100
C	-14.49561800	3.01114100	0.20788300
C	-14.85220800	1.86259900	0.86482700
S	-13.92414200	0.53713200	0.30072300
H	16.50013600	-1.04678000	2.09311300
H	16.01164900	-3.58759700	1.48961600
H	12.94448600	-2.60722300	-2.87888300
H	9.67712600	1.42262500	-4.48833700
H	6.52867200	5.07521200	-1.99013300
H	3.73108100	5.23226100	2.65816700
H	1.24393900	1.58424500	5.81292500
H	-1.19624500	-3.19631300	5.04601400
H	-3.83527100	-5.60372400	0.96915400
H	-6.83138900	-3.95773400	-3.24186200
H	-10.03108300	0.28876600	-4.31428900
H	-13.08818200	3.67278000	-1.35641300
H	-15.61307900	1.75359700	1.62302900
N	13.99761000	-4.16333000	-0.27750700
O	13.23257800	-4.25749100	-1.23818100
O	14.42225500	-5.11742300	0.37506100
N	10.85605800	-1.53912400	-4.27862700
O	10.02734400	-0.83748800	-4.86023300
O	11.30310100	-2.59997700	-4.71026100
N	7.57431700	3.15396800	-4.31217100
O	6.76083500	4.03646000	-4.03534700
O	7.93219400	2.86202500	-5.45140800
N	4.53077300	5.93948400	-0.34385900
O	3.76942700	6.23065000	0.57943000

O	4.81376100	6.68312500	-1.28091600
N	1.89644600	4.40572400	4.49918300
O	1.21601600	3.83309800	5.35121100
O	2.15395700	5.60780000	4.49884000
N	-0.50339300	-0.41890100	6.41607700
O	-1.16845600	-1.44886300	6.53190000
O	-0.17934800	0.31497000	7.34766500
N	-2.97295500	-4.95152300	3.95499700
O	-3.65875500	-5.69863100	3.25643300
O	-2.57482100	-5.21944700	5.08693600
N	-5.73955500	-5.87849800	-0.96088400
O	-6.49691900	-5.80909000	-1.92935100
O	-5.27578300	-6.92269100	-0.50663600
N	-8.86818900	-2.66154000	-4.50484300
O	-9.67579800	-1.87128600	-4.99435300
O	-8.42530100	-3.65599600	-5.07670100
N	-12.09155900	2.02976500	-3.91894500
O	-12.87354800	2.89920400	-3.53314300
O	-11.74741100	1.86468100	-5.08811600
N	-15.12607900	4.28963500	0.49910700
O	-16.01707500	4.29970700	1.34829900
O	-14.71851500	5.26950100	-0.12196200
OH			
C	-3.20995500	-8.28155600	-0.54132600
C	-4.33212400	-9.05873400	-0.47893600
C	-5.52515600	-8.28627500	-0.37492300
C	-5.30935600	-6.91611300	-0.35774200
S	-3.58462400	-6.59497700	-0.47595000
C	-6.29039400	-5.86372800	-0.26111200
C	-7.66583100	-5.97921000	-0.19085500
C	-8.32775900	-4.73033100	-0.10467800
C	-7.48391100	-3.62753400	-0.10605200
S	-5.81541700	-4.17581300	-0.21786200
C	-7.83449500	-2.23670000	-0.03052200
C	-9.09526400	-1.67028200	0.05077600
C	-9.06967500	-0.25730300	0.11042200
C	-7.79805900	0.30231600	0.07686200
S	-6.60289300	-0.98560800	-0.03204700
C	-7.43341500	1.68937500	0.11965800
C	-8.26275600	2.79607700	0.19901500
C	-7.55776600	4.02149000	0.22017000
C	-6.17463900	3.89782800	0.15872100
S	-5.75119100	2.19080700	0.07158800
C	-5.18591900	4.93691300	0.16029600
C	-5.37557700	6.30828900	0.21506800
C	-4.16620900	7.04032400	0.19670700
C	-3.01603900	6.26263300	0.12835800
S	-3.47162500	4.56204600	0.08550800

C	-1.64884500	6.69410900	0.09328000
C	-1.15170300	7.98737600	0.10752500
C	0.26003300	8.04348600	0.05973300
C	0.89076100	6.80580100	0.00848700
S	-0.32995600	5.53614900	0.01901000
C	2.29586700	6.52369400	-0.04562900
C	3.35316500	7.41797500	-0.09103500
C	4.61710100	6.78664300	-0.13945800
C	4.57501600	5.39742300	-0.13252100
S	2.89517000	4.87225000	-0.06363900
C	5.67167200	4.47362300	-0.17223300
C	7.02634800	4.74874100	-0.26336100
C	7.83281100	3.58759100	-0.28012700
C	7.13191300	2.39025900	-0.20219700
S	5.40767900	2.73779100	-0.10607700
C	7.65259800	1.05307600	-0.19289700
C	8.97062300	0.64278700	-0.30359600
C	9.12520800	-0.76228900	-0.25561400
C	7.94182900	-1.47354300	-0.10648000
S	6.59546100	-0.34060700	-0.02642500
C	7.76213700	-2.89610700	-0.01958000
C	8.71942900	-3.89091100	-0.10977700
C	8.18538000	-5.19605900	0.02314200
C	6.81386000	-5.24590800	0.21820500
S	6.17519200	-3.60533900	0.23659200
C	5.97461600	-6.40554600	0.39067400
C	6.34156600	-7.73771100	0.38482200
C	5.24183500	-8.62182700	0.58989200
C	4.04229800	-7.98181600	0.75133700
S	4.24278700	-6.25721700	0.65428400
H	-2.18120500	-8.60174400	-0.62314300
H	-4.31836800	-10.14343400	-0.50524300
H	-8.17371100	-6.93492200	-0.20283700
H	-10.00147700	-2.26180500	0.06584400
H	-9.34127300	2.71526800	0.23971600
H	-6.35790100	6.75982500	0.26593200
H	-1.79267900	8.85847400	0.15033300
H	3.21000500	8.49079700	-0.09070900
H	7.41423200	5.75775400	-0.31706900
H	9.79083700	1.33999300	-0.41625600
H	9.76903200	-3.67876900	-0.26780100
H	7.36435900	-8.06042800	0.23829500
H	3.07217300	-8.42224000	0.92192700
O	5.34412000	-9.98678000	0.62572500
H	6.27151500	-10.22457100	0.50334900
O	8.93937300	-6.33726900	-0.03315700
H	9.86189000	-6.08663900	-0.16841200
O	10.33118800	-1.40156100	-0.34634400

H	11.02475800	-0.73517500	-0.43157000
O	9.19758500	3.60602200	-0.36520300
H	9.49031000	4.52577100	-0.39411000
O	5.80646600	7.45878100	-0.18968700
H	5.62423400	8.40699500	-0.17495900
O	0.98040300	9.20511100	0.06263000
H	0.36556000	9.94816700	0.11172200
O	-4.09649600	8.40442000	0.24112200
H	-4.99425000	8.75701800	0.28894200
O	-8.15559600	5.24794900	0.29411200
H	-9.11262900	5.12310200	0.32762800
O	-10.18572700	0.52636100	0.19426000
H	-10.96420900	-0.04496600	0.20820200
O	-9.68381000	-4.58603600	-0.02597100
H	-10.08974500	-5.46225100	-0.03372400
O	-6.78329500	-8.81339800	-0.29702200
H	-6.72179900	-9.77683500	-0.31446100
OMe			
C	-2.43974400	-8.09202200	-1.02719000
C	-3.46785000	-8.98075700	-0.87498100
C	-4.72415400	-8.33120300	-0.68137800
C	-4.64329200	-6.94186200	-0.68600800
S	-2.97646700	-6.45259500	-0.94321100
C	-5.70646400	-5.98333500	-0.51240600
C	-7.06489700	-6.21739100	-0.39317100
C	-7.82475400	-5.03024400	-0.23625100
C	-7.06869200	-3.85973100	-0.23127500
S	-5.36951900	-4.26557500	-0.42260400
C	-7.51511800	-2.50175400	-0.09574600
C	-8.80987900	-2.02673900	0.04557400
C	-8.87805700	-0.61610100	0.15298400
C	-7.64246100	0.02840800	0.09668500
S	-6.37147900	-1.17141600	-0.09242600
C	-7.35605600	1.43187000	0.17074600
C	-8.23957800	2.49027600	0.32379700
C	-7.59871500	3.75235300	0.35281800
C	-6.21052700	3.69983900	0.22562900
S	-5.70739700	2.02314200	0.05946900
C	-5.26676700	4.77908700	0.21998200
C	-5.50854500	6.14399300	0.27924600
C	-4.32634800	6.92209700	0.24694900
C	-3.14705800	6.18126400	0.16287300
S	-3.54175800	4.46786500	0.12824000
C	-1.79315200	6.64889600	0.10741300
C	-1.32524400	7.95522900	0.11247500
C	0.08566600	8.04291400	0.04116500
C	0.74105400	6.81268500	-0.01784700
S	-0.45167600	5.52041800	0.01146500

C	2.14788000	6.54709800	-0.09128300
C	3.19625000	7.45062000	-0.18889700
C	4.46632800	6.82855300	-0.24434400
C	4.43261500	5.43496900	-0.19049800
S	2.76113100	4.90200200	-0.06379300
C	5.52665700	4.50882400	-0.22607200
C	6.88290600	4.77524300	-0.34533300
C	7.68356400	3.60786800	-0.34952500
C	6.97071600	2.41491300	-0.23168000
S	5.25322400	2.77760500	-0.11766100
C	7.47035600	1.07071500	-0.19724800
C	8.77886700	0.63135400	-0.33112800
C	8.90736600	-0.77633800	-0.24786500
C	7.70813500	-1.45738500	-0.04630800
S	6.39092200	-0.29472000	0.03674200
C	7.49026900	-2.87055800	0.08732900
C	8.41304100	-3.89931500	-0.00922700
C	7.84145500	-5.18339700	0.17891800
C	6.47200400	-5.17742300	0.42320600
S	5.89264700	-3.51767100	0.42296800
C	5.59224000	-6.29581400	0.65553300
C	5.90932400	-7.64120100	0.72243000
C	4.77375500	-8.46905300	0.97223000
C	3.59994200	-7.76893300	1.09539100
S	3.86742500	-6.06549600	0.90309000
H	-1.39313900	-8.30791400	-1.18752500
H	-3.33110500	-10.05364200	-0.89952400
H	-7.47681200	-7.21391500	-0.42419400
H	-9.66068400	-2.68959500	0.06633500
H	-9.30358900	2.33533300	0.41002000
H	-6.50928400	6.54221600	0.33901800
H	-1.99498900	8.79921100	0.16555500
H	3.02783700	8.51552600	-0.22183300
H	7.25954000	5.78276000	-0.42612900
H	9.59642700	1.31792000	-0.48509600
H	9.45570000	-3.71147900	-0.21162800
H	6.92032500	-7.99641900	0.59724400
H	2.61450900	-8.16234600	1.29152100
O	4.77867000	-9.83095500	1.08969600
O	8.52150100	-6.36691100	0.13414600
O	10.07651700	-1.47307100	-0.34850400
O	9.04331600	3.57907900	-0.45501300
O	5.66287600	7.47509700	-0.34384400
O	0.80983900	9.19810000	0.02927600
O	-4.27021000	8.28349700	0.28870500
O	-8.22165700	4.95658900	0.49256000
O	-10.01880300	0.11499900	0.29965000
O	-9.17900500	-4.96664600	-0.09927900

O	-5.92459100	-8.95039800	-0.50104500
C	6.03362600	-10.48175000	0.97707700
H	6.48973700	-10.31909800	-0.00825900
H	6.73558100	-10.14906000	1.75277300
H	5.83618100	-11.54736300	1.10731200
C	9.91478000	-6.30629500	-0.13451100
H	10.26879900	-7.33844500	-0.13299600
H	10.11640300	-5.85644600	-1.11451900
H	10.44950200	-5.73971200	0.63783200
C	11.26752300	-0.72117800	-0.53625400
H	12.07971200	-1.44799200	-0.58680200
H	11.23682300	-0.14711700	-1.47041200
H	11.44617600	-0.03619500	0.30155100
C	9.71562400	4.82826500	-0.54202800
H	9.41278800	5.38561200	-1.43684400
H	9.53178900	5.44489500	0.34607400
H	10.77966200	4.59564300	-0.60573700
C	5.63311400	8.89538200	-0.39737300
H	5.07884900	9.25180300	-1.27408400
H	5.18678000	9.32160100	0.50924400
H	6.67280300	9.21728700	-0.47283300
C	0.08831100	10.41998400	0.11595200
H	-0.59003600	10.54626200	-0.73653200
H	-0.48742700	10.47993900	1.04736500
H	0.83594900	11.21437900	0.10230100
C	-5.50492300	8.98430800	0.36577400
H	-6.13594800	8.78069600	-0.50772300
H	-6.05624800	8.72553000	1.27779900
H	-5.25005200	10.04483200	0.38732800
C	-9.63548200	4.94519100	0.64442900
H	-10.12939500	4.51297300	-0.23422700
H	-9.93726600	4.38775200	1.53929500
H	-9.93675600	5.98829600	0.75045900
C	-11.24838600	-0.59795900	0.34837500
H	-11.27710500	-1.28842800	1.19987200
H	-12.02950200	0.15416300	0.46727700
H	-11.42283400	-1.15865800	-0.57780800
C	-9.89461200	-6.19594600	-0.10839700
H	-9.58385300	-6.84616900	0.71813900
H	-10.94636000	-5.93418100	0.01476400
H	-9.76259900	-6.72890400	-1.05764000
C	-5.92734700	-10.37232100	-0.46104100
H	-5.30458800	-10.75144900	0.35818700
H	-6.96335400	-10.66805000	-0.29116400
H	-5.58102000	-10.80122100	-1.40927300
Ph			
C	-16.83561000	0.78775300	-2.86487300
C	-16.47189600	0.07908200	-3.97264100

C	-15.32007100	-0.75323300	-3.77788600
C	-14.83189600	-0.66748200	-2.48104700
S	-15.79744500	0.44570000	-1.52445900
C	-13.74894600	-1.40434300	-1.84467000
C	-13.39617700	-2.72323300	-2.00665100
C	-12.26617800	-3.13885000	-1.23933300
C	-11.76142400	-2.11207000	-0.44993400
S	-12.67748800	-0.63465800	-0.69142500
C	-10.69690800	-2.13877400	0.54059900
C	-10.35837100	-3.14689500	1.41352000
C	-9.24516400	-2.85788800	2.25838900
C	-8.73775400	-1.58175700	2.04128200
S	-9.63141200	-0.76583700	0.77023500
C	-7.68595400	-0.86726500	2.74657700
C	-7.36678200	-0.90512000	4.08450300
C	-6.26129200	-0.08437800	4.45921700
C	-5.73953900	0.61999900	3.37993100
S	-6.61084200	0.23337000	1.90637600
C	-4.69012100	1.62559900	3.34608000
C	-4.38117100	2.58036300	4.28765900
C	-3.27652100	3.41795400	3.95002700
C	-2.74516800	3.11047500	2.70236900
S	-3.60568900	1.76461500	1.97566700
C	-1.69350200	3.77520900	1.95030200
C	-1.38634500	5.11566300	1.89916900
C	-0.27854700	5.44408600	1.06215100
C	0.25786900	4.32438700	0.43586500
S	-0.60258200	2.87229300	0.91683400
C	1.31363000	4.23174100	-0.55909500
C	1.62450000	5.11204900	-1.57012900
C	2.73508900	4.72658600	-2.37857200
C	3.26975700	3.50319100	-1.98958800
S	2.40427200	2.85983000	-0.60494500
C	4.32677600	2.71401200	-2.60049300
C	4.64289300	2.58208300	-3.93330400
C	5.75257800	1.72687900	-4.20323000
C	6.28162700	1.16959600	-3.04425900
S	5.41186100	1.73742900	-1.62962300
C	7.33596400	0.18133000	-2.88482900
C	7.64741800	-0.88460600	-3.69737000
C	8.75624100	-1.66796100	-3.25772600
C	9.28875600	-1.20112000	-2.06130100
S	8.42392800	0.22322900	-1.51063200
C	10.34399800	-1.76060200	-1.23181100
C	10.64725400	-3.08207700	-0.99955000
C	11.76145800	-3.29683100	-0.13310600
C	12.30642000	-2.10451700	0.32750000
S	11.44623300	-0.72904200	-0.33996000

C	13.37418600	-1.87909500	1.29150200
C	13.65747500	-2.57855400	2.44023400
C	14.79103500	-2.08640800	3.16538800
C	15.35106500	-0.99349600	2.54298400
S	14.52879000	-0.56925500	1.08290700
H	-17.64037800	1.50197000	-2.76087600
H	-16.97564500	0.16461000	-4.92849400
H	-13.91794100	-3.38560700	-2.68594700
H	-10.87950600	-4.09554400	1.43750200
H	-7.89694500	-1.52991400	4.79204200
H	-4.91835400	2.67073100	5.22323500
H	-1.92739500	5.85920200	2.47043700
H	1.08400100	6.03748200	-1.72297900
H	4.10704100	3.10724300	-4.71365200
H	7.10883000	-1.09594700	-4.61238100
H	10.09915800	-3.89461600	-1.45936500
H	13.07575500	-3.43709200	2.75061700
H	16.17992000	-0.39043600	2.88587700
C	15.28408400	-2.66777400	4.43251200
C	16.64865100	-2.62566900	4.76525200
C	14.39783600	-3.27294900	5.33931500
C	17.10913500	-3.15990700	5.96669600
H	17.35479100	-2.19121100	4.06382600
C	14.85984100	-3.81286800	6.53831900
H	13.33641200	-3.30099800	5.11276300
C	16.21695300	-3.75720200	6.85911300
H	18.16903200	-3.11966100	6.20124600
H	14.15586300	-4.27190000	7.22650400
H	16.57651000	-4.17781200	7.79349500
C	12.24857800	-4.66311800	0.18223400
C	13.60856100	-4.99931100	0.07973100
C	11.33635700	-5.66767100	0.54412400
C	14.04134800	-6.29766600	0.34031600
H	14.32379600	-4.23833100	-0.21435100
C	11.77081200	-6.96739500	0.80405000
H	10.28208100	-5.42205200	0.63331900
C	13.12511700	-7.28698000	0.70443000
H	15.09666900	-6.53925900	0.25233700
H	11.04972500	-7.72889300	1.08697900
H	13.46467900	-8.29859700	0.90634000
C	9.25391000	-2.82162100	-4.04819400
C	10.61565800	-2.96062900	-4.36367100
C	8.35094300	-3.77611200	-4.54381200
C	11.05936200	-4.02697700	-5.14264800
H	11.32352400	-2.22214800	-4.00190800
C	8.79646800	-4.84308700	-5.32402900
H	7.29553700	-3.68518100	-4.30379900
C	10.15240600	-4.97324300	-5.62515500

H	12.11572600	-4.11562800	-5.37928400
H	8.08275100	-5.57366200	-5.69378600
H	10.50045100	-5.80346700	-6.23260700
C	6.25465800	1.52031200	-5.58479100
C	7.61713300	1.65799900	-5.89760600
C	5.35471800	1.23257700	-6.62373100
C	8.06456600	1.50276600	-7.20778700
H	8.32251600	1.89632500	-5.10832400
C	5.80395400	1.07793500	-7.93518800
H	4.29876900	1.11794400	-6.39656800
C	7.16059700	1.21114900	-8.23183200
H	9.12146100	1.61646500	-7.43091600
H	5.09259300	0.85070600	-8.72397400
H	7.51151600	1.09117900	-9.25254800
C	3.23263700	5.59212000	-3.47717500
C	4.59289700	5.92434200	-3.58909200
C	2.33047900	6.14222200	-4.40211300
C	5.03617600	6.77255600	-4.60146800
H	5.29979500	5.52059400	-2.87172400
C	2.77558600	6.99178600	-5.41498100
H	1.27622200	5.89063000	-4.33135500
C	4.13015100	7.30886000	-5.51925100
H	6.09133500	7.02096600	-4.66976400
H	2.06267000	7.40322200	-6.12372000
H	4.47785000	7.97023300	-6.30734100
C	0.21378600	6.83910200	0.93887400
C	1.57316100	7.15353300	1.10123700
C	-0.69304900	7.88615500	0.70790000
C	2.01102500	8.47388100	1.02479000
H	2.28363300	6.35710500	1.29690100
C	-0.25335800	9.20773900	0.63196700
H	-1.74667800	7.65803800	0.57522600
C	1.10034700	9.50627000	0.78877700
H	3.06558900	8.69800700	1.15662800
H	-0.96980600	10.00307200	0.44761200
H	1.44382100	10.53485300	0.73014800
C	-2.78156300	4.46260800	4.88130800
C	-1.42075500	4.56116800	5.21527900
C	-3.68669600	5.34634300	5.49098500
C	-0.97984900	5.52107700	6.12364900
H	-0.71144200	3.87443800	4.76511400
C	-3.24396500	6.30669700	6.40065700
H	-4.74144200	5.28564700	5.23878000
C	-1.88883300	6.39851000	6.71918600
H	0.07582700	5.57967000	6.37232700
H	-3.95916300	6.98444800	6.85780800
H	-1.54294300	7.14570000	7.42732000
C	-5.75613800	-0.05264200	5.85466300

C	-4.39245200	-0.22462200	6.14440100
C	-6.65416500	0.09885900	6.92356700
C	-3.94192900	-0.23469000	7.46268900
H	-3.68851300	-0.35991300	5.32983700
C	-6.20180600	0.08821700	8.24301500
H	-7.71102800	0.23796100	6.71506600
C	-4.84400400	-0.07713700	8.51733700
H	-2.88419400	-0.37267600	7.66729800
H	-6.91160600	0.21198600	9.05585700
H	-4.49071700	-0.08575200	9.54422700
C	-8.71867300	-3.85970900	3.21890200
C	-7.35045700	-4.17282500	3.27459700
C	-9.60014200	-4.55693300	4.06092400
C	-6.87951500	-5.14654000	4.15263900
H	-6.65900500	-3.65463100	2.61834000
C	-9.12733700	-5.53185200	4.93938500
H	-10.66026700	-4.32206600	4.03320300
C	-7.76523600	-5.82924800	4.98946600
H	-5.81856000	-5.37764600	4.17871000
H	-9.82457600	-6.05653800	5.58623700
H	-7.39602200	-6.58824300	5.67283700
C	-11.72354100	-4.51669400	-1.33940100
C	-10.35453200	-4.75201500	-1.54808100
C	-12.59076800	-5.61953100	-1.27856200
C	-9.86894200	-6.05090300	-1.68143500
H	-9.67412400	-3.90949700	-1.61491900
C	-12.10335200	-6.91949600	-1.41265500
H	-13.65132600	-5.45404500	-1.11238600
C	-10.74052500	-7.14009000	-1.61308500
H	-8.80761900	-6.21269700	-1.84610700
H	-12.78977300	-7.75952800	-1.35758400
H	-10.35988900	-8.15178600	-1.71776200
C	-14.74060400	-1.55377600	-4.88438400
C	-13.36605000	-1.52228600	-5.17286900
C	-15.57872500	-2.32603500	-5.70535800
C	-12.84733600	-2.24913100	-6.24216700
H	-12.70774000	-0.91554900	-4.55999000
C	-15.05824000	-3.05293600	-6.77600000
H	-16.64302700	-2.36576500	-5.49154400
C	-13.69027600	-3.01832300	-7.04752900
H	-11.78235700	-2.20893500	-6.45166900
H	-15.72261900	-3.64851700	-7.39551800
H	-13.28361100	-3.58349600	-7.88093100
SH			
C	-15.33648700	-1.17405400	-4.10311800
C	-14.86257300	-2.30314800	-4.70512400
C	-13.84950400	-2.96234700	-3.93927300
C	-13.57474000	-2.32416700	-2.74128600

S	-14.59047600	-0.89805400	-2.56793000
C	-12.62239000	-2.68382800	-1.70970600
C	-12.23424700	-3.94140400	-1.30656300
C	-11.24698300	-3.93923600	-0.27927600
C	-10.89227000	-2.66596200	0.13700800
S	-11.78242100	-1.45807300	-0.78035100
C	-9.94695200	-2.26523500	1.15660700
C	-9.62066000	-2.91878900	2.32455600
C	-8.62322500	-2.25790100	3.09653300
C	-8.19639400	-1.06441400	2.53527800
S	-9.03204900	-0.77619200	1.01501400
C	-7.21820300	-0.11910800	3.02754300
C	-6.90819300	0.18737100	4.33449400
C	-5.86791600	1.14985900	4.47047600
C	-5.38992000	1.61647200	3.25569300
S	-6.23206000	0.82691000	1.92902400
C	-4.35766700	2.59541800	2.99358600
C	-4.01343400	3.69657800	3.74665200
C	-2.92246500	4.44112400	3.21517200
C	-2.43787200	3.92870000	2.02157300
S	-3.33984700	2.48815000	1.56989500
C	-1.35652800	4.40972500	1.19000500
C	-0.93375500	5.70741800	1.00113900
C	0.18953500	5.82826000	0.13473500
C	0.62061100	4.61442000	-0.37801000
S	-0.37338000	3.30537400	0.24733000
C	1.71168600	4.33816800	-1.28657400
C	2.21064300	5.13554500	-2.29350500
C	3.31903400	4.56788500	-2.98358800
C	3.66075100	3.30404700	-2.52790800
S	2.60178600	2.82931800	-1.20674000
C	4.70905700	2.41775300	-2.98420800
C	5.22227500	2.28350300	-4.25574800
C	6.27659500	1.33173700	-4.35492800
C	6.56037300	0.69957900	-3.15441800
S	5.51529600	1.31766500	-1.88220400
C	7.54389300	-0.32181100	-2.86678600
C	8.00330500	-1.32321100	-3.69369200
C	9.00317000	-2.14670400	-3.10204900
C	9.29696700	-1.79893900	-1.79308100
S	8.33102400	-0.41389200	-1.30258700
C	10.23672000	-2.40258900	-0.87281800
C	10.61074800	-3.72458300	-0.77739000
C	11.59104100	-3.97524500	0.22579600
C	11.95325000	-2.84232200	0.93424300
S	11.07946400	-1.44243900	0.32900100
C	12.89747200	-2.70797200	2.02581800
C	13.17043700	-3.60044100	3.03735100

C	14.18773900	-3.14699300	3.93306900
C	14.66404100	-1.90302600	3.61227700
S	13.89930500	-1.26957700	2.19408000
H	-16.09190000	-0.49287700	-4.46906200
H	-15.21172200	-2.65985900	-5.66702600
H	-12.66760300	-4.84883400	-1.70841900
H	-10.10669600	-3.83164300	2.64525600
H	-7.43501700	-0.23576000	5.18050800
H	-4.54913700	3.99124300	4.64024900
H	-1.43403800	6.56118700	1.44051600
H	1.77487600	6.08974000	-2.56154400
H	4.83265600	2.82351600	-5.10949600
H	7.61159700	-1.49270300	-4.68879100
H	10.17142100	-4.50772900	-1.38247400
H	12.63419900	-4.53101900	3.17356400
H	15.42497000	-1.33368400	4.12640100
S	14.71484700	-4.02385700	5.39946700
H	15.22539000	-5.10839800	4.77734700
S	12.27174100	-5.59172900	0.54312900
H	12.00003700	-6.09336400	-0.67785700
S	9.79029300	-3.49271800	-3.96481200
H	9.50709900	-3.04294400	-5.20290900
S	7.13788000	0.99010900	-5.87698900
H	6.81557400	2.15204100	-6.47806900
S	4.17454400	5.40570000	-4.30298100
H	3.76380400	6.64356600	-3.96508900
S	0.95661000	7.38998200	-0.24861600
H	0.47507700	8.04431900	0.82607800
S	-2.25582000	5.88891400	4.01113500
H	-2.73813600	5.60365400	5.23630900
S	-5.25029000	1.69642000	6.04970200
H	-5.69697000	0.63219200	6.74462800
S	-7.99492900	-2.90604600	4.63243600
H	-8.38139800	-4.17742100	4.41068000
S	-10.54679100	-5.42966700	0.40041200
H	-10.86548500	-6.21811600	-0.64462400
S	-13.01779400	-4.44256800	-4.47910100
H	-13.33127400	-4.30759400	-5.78250500
SiH_3			
C	-16.74467400	0.75676200	-2.77844000
C	-16.31999400	0.16357700	-3.93348500
C	-15.17392300	-0.68604900	-3.77054800
C	-14.75369000	-0.72195500	-2.44812300
S	-15.76888100	0.28660100	-1.42980400
C	-13.65349900	-1.47220000	-1.86177100
C	-13.23058400	-2.74750700	-2.16553800
C	-12.09228800	-3.20026800	-1.42811300
C	-11.65701600	-2.24106100	-0.52228100

S	-12.63902900	-0.78943100	-0.60796100
C	-10.56139200	-2.31798400	0.42939300
C	-10.13568900	-3.40833100	1.15730000
C	-9.00507800	-3.17089600	1.99858300
C	-8.57795200	-1.85099100	1.91887100
S	-9.55890100	-0.92849900	0.79423700
C	-7.49009800	-1.20203000	2.63075700
C	-7.07104100	-1.41306200	3.92688600
C	-5.94680400	-0.63098400	4.33485900
C	-5.51788900	0.21454100	3.31880100
S	-6.48964300	0.01899000	1.87119900
C	-4.43382300	1.18183000	3.33679700
C	-4.02162200	1.98420000	4.37915700
C	-2.89846000	2.81977900	4.09255300
C	-2.46321600	2.65783500	2.78270200
S	-3.42846800	1.46607200	1.93073700
C	-1.37642800	3.33378600	2.09486400
C	-0.96520400	4.64298900	2.22560700
C	0.16164500	5.00652100	1.42565400
C	0.60090300	3.94097300	0.64904500
S	-0.36546800	2.50385900	0.92969800
C	1.69154800	3.90223200	-0.31007100
C	2.10817500	4.89429500	-1.17173300
C	3.23705800	4.55875300	-1.98091500
C	3.67229400	3.26161400	-1.73677600
S	2.70002600	2.48275900	-0.50143900
C	4.76279700	2.53394200	-2.36313800
C	5.18232600	2.58497800	-3.67523600
C	6.30900800	1.76182100	-3.98304700
C	6.73956200	1.04928300	-2.87022200
S	5.76567500	1.41839000	-1.45843700
C	7.82584300	0.08977700	-2.76803200
C	8.24091100	-0.83381700	-3.70323600
C	9.36460200	-1.62627200	-3.31415600
C	9.79721600	-1.30417100	-2.03348100
S	8.82905900	-0.01803100	-1.33602500
C	10.88235200	-1.89025600	-1.26465700
C	11.28843100	-3.20669200	-1.22649100
C	12.41454500	-3.46760000	-0.38542700
C	12.85693100	-2.31349100	0.24730300
S	11.89684200	-0.92109300	-0.21544300
C	13.94860200	-2.15816900	1.19701400
C	14.32172700	-3.00942000	2.21273400
C	15.45756700	-2.56878600	2.96957100
C	15.92212400	-1.36057300	2.50430700
S	15.01528300	-0.76325800	1.15569500
H	-17.56742100	1.44445000	-2.63998400
H	-16.79691900	0.33930300	-4.89145600

H	-13.74246100	-3.36246800	-2.89708400
H	-10.64114700	-4.36596500	1.10657600
H	-7.57714100	-2.10667700	4.58859700
H	-4.53172100	1.99102500	5.33565400
H	-1.47852400	5.34465900	2.87313100
H	1.59722600	5.84789700	-1.24044800
H	4.67482500	3.18994200	-4.41792500
H	7.73234500	-0.95926300	-4.65234700
H	10.77222900	-3.98404900	-1.77826600
H	13.76464900	-3.91193700	2.43852200
H	16.75580700	-0.78042200	2.87722400
Si	16.22890400	-3.47179500	4.41898600
H	16.74213900	-4.80593200	4.00935500
H	17.35731900	-2.65459200	4.93454500
H	15.23856300	-3.67838800	5.50882000
Si	13.19292800	-5.17438100	-0.27135700
H	14.66942500	-5.09809300	-0.39616200
H	12.86667400	-5.86346100	1.00640500
H	12.63922700	-5.98872700	-1.38423100
Si	10.15063300	-2.86553700	-4.48813800
H	11.62727100	-2.72079300	-4.50720600
H	9.82396000	-4.27237200	-4.13065800
H	9.60448800	-2.59862300	-5.84393200
Si	7.10064200	1.77290700	-5.68745700
H	8.57647700	1.89161400	-5.58967000
H	6.78190400	0.54795700	-6.46957100
H	6.55208900	2.94173700	-6.42271700
Si	4.02499600	5.81153900	-3.13918100
H	5.50055900	5.82886200	-2.98337700
H	3.70978300	5.54063000	-4.56782100
H	3.46957000	7.14516900	-2.79210800
Si	0.94195500	6.71212700	1.54307100
H	2.41782400	6.61702100	1.66352600
H	0.62398700	7.56443400	0.36564400
H	0.38254700	7.37199700	2.75103500
Si	-2.11672400	3.90440200	5.41326100
H	-0.64028300	3.75638500	5.42140700
H	-2.44001100	5.34509700	5.22951700
H	-2.66995600	3.47395000	6.72328300
Si	-5.15734100	-0.85073900	6.02601700
H	-3.68106500	-0.95337600	5.91728700
H	-5.48013200	0.26766500	6.95282200
H	-5.70441400	-2.10247100	6.61039700
Si	-8.20633400	-4.55512100	2.98705700
H	-6.73107600	-4.54419100	2.82723500
H	-8.51817700	-4.46933000	4.43950400
H	-8.75507100	-5.83783400	2.47603300
Si	-11.29702900	-4.86753700	-1.77071200

H	-9.82212400	-4.74527300	-1.87968700
H	-11.60384900	-5.87131800	-0.71571100
H	-11.85284000	-5.36927200	-3.05416500
Si	-14.34385800	-1.52306800	-5.23123600
H	-12.86994500	-1.35473200	-5.18484900
H	-14.64191900	-2.97982600	-5.29982600
H	-14.87617100	-0.89287800	-6.46801000
SMe			
C	-15.10147800	-1.22968600	-4.03315600
C	-14.58298100	-2.29421600	-4.70851700
C	-13.54073300	-2.96845700	-3.99248700
C	-13.28956000	-2.40339700	-2.74968100
S	-14.36401300	-1.03820900	-2.47745100
C	-12.32255000	-2.77314000	-1.73183200
C	-11.88008700	-4.02326100	-1.37166300
C	-10.90566100	-4.02144600	-0.32969300
C	-10.61601800	-2.74750100	0.14244800
S	-11.55902200	-1.54866900	-0.73049200
C	-9.69775400	-2.32434300	1.18017100
C	-9.33558500	-2.97746000	2.33534200
C	-8.38571100	-2.27388700	3.13262000
C	-8.03336700	-1.04049500	2.59909500
S	-8.88494100	-0.76878300	1.08627500
C	-7.11350700	-0.03815400	3.09766600
C	-6.83035300	0.30338100	4.39944100
C	-5.85113900	1.33033600	4.53561400
C	-5.39309500	1.81189500	3.31498800
S	-6.18659900	0.96678900	1.99398400
C	-4.40815400	2.83406700	3.02634900
C	-4.10362100	3.96970800	3.74059500
C	-3.05396200	4.75229400	3.17655300
C	-2.56142100	4.22779600	1.98729200
S	-3.41161900	2.74337100	1.58229000
C	-1.50610900	4.70953900	1.12098400
C	-1.09889900	6.00194300	0.88132200
C	-0.00362300	6.11293600	-0.02439300
C	0.42276000	4.88612200	-0.51926800
S	-0.54675600	3.58943200	0.16465400
C	1.49090400	4.56844700	-1.44474500
C	1.98939900	5.31573300	-2.48723100
C	3.06247700	4.69584700	-3.19128100
C	3.37623500	3.43199700	-2.70564100
S	2.33140900	3.02743200	-1.35193200
C	4.38445200	2.48855800	-3.14257000
C	4.88143900	2.27704700	-4.40789400
C	5.88778000	1.27017600	-4.48138400
C	6.14890600	0.66912600	-3.25600200
S	5.13846600	1.38042000	-2.00575000

C	7.08316400	-0.38334300	-2.91286200
C	7.50916600	-1.44571600	-3.67586600
C	8.45379500	-2.29248100	-3.02513500
C	8.73763200	-1.89515500	-1.72436700
S	7.82649300	-0.44739000	-1.32142200
C	9.62851500	-2.48016900	-0.74267400
C	9.95031000	-3.80270500	-0.54614900
C	10.88410600	-4.02731400	0.50851700
C	11.26202400	-2.86063800	1.15881400
S	10.45570900	-1.47558000	0.43877200
C	12.17379600	-2.66645400	2.27050400
C	12.40295000	-3.48186200	3.35418300
C	13.39089400	-2.97220000	4.25488900
C	13.89205000	-1.75900400	3.85655800
S	13.18346500	-1.22446300	2.37043700
H	-15.89003900	-0.55842700	-4.34342300
H	-14.92372100	-2.60692000	-5.68835100
H	-12.25751400	-4.92978000	-1.82422000
H	-9.75584900	-3.92928300	2.62939100
H	-7.32871900	-0.14923900	5.24534000
H	-4.63353400	4.25518300	4.63843300
H	-1.58520600	6.86284400	1.31863400
H	1.58414800	6.27882400	-2.76368300
H	4.51905900	2.80798800	-5.27709300
H	7.13777900	-1.63853700	-4.67271100
H	9.51106200	-4.60437900	-1.12325500
H	11.85967900	-4.40046900	3.53005400
H	14.63694300	-1.15670000	4.35673700
S	13.86312900	-3.75852800	5.78326800
S	11.45871500	-5.65468900	0.94709900
S	9.15068800	-3.72504100	-3.82142900
S	6.68175900	0.83402800	-6.01486400
S	3.87337100	5.47866500	-4.57005000
S	0.69431800	7.68923900	-0.47310400
S	-2.48255900	6.26291600	3.92477000
S	-5.32010700	1.93147800	6.12527200
S	-7.76014600	-2.93333000	4.66442900
S	-10.18187000	-5.52019100	0.30232000
S	-12.69467200	-4.39157400	-4.64991100
C	14.76930600	-5.22423300	5.15113200
H	14.12908500	-5.83386900	4.51074300
H	15.66476700	-4.92249300	4.60457000
H	15.05901500	-5.80854100	6.02753500
C	12.07104800	-6.26203000	-0.67158000
H	11.27315300	-6.29835700	-1.41523200
H	12.89088500	-5.63880500	-1.03240800
H	12.43695000	-7.27568900	-0.49292400
C	9.88770700	-2.97015400	-5.32218200

H	9.13160800	-2.46798400	-5.92802500
H	10.67815800	-2.26813800	-5.05186200
H	10.31492600	-3.79539200	-5.89642700
C	7.33937600	2.46223100	-6.54610400
H	6.53964500	3.19488600	-6.66625500
H	8.08007200	2.82944000	-5.83391300
H	7.81721200	2.29354600	-7.51382600
C	4.38319300	7.07426500	-3.82256300
H	3.52213300	7.64161100	-3.46566400
H	5.08817700	6.90931700	-3.00622700
H	4.87471000	7.63650400	-4.61976700
C	1.20443300	8.32807100	1.16934700
H	0.35418400	8.39571600	1.85001200
H	1.98295800	7.69856100	1.60333600
H	1.60235600	9.33016000	0.99393800
C	-2.14854600	5.71107500	5.64135500
H	-3.05399700	5.34774700	6.12990000
H	-1.37881500	4.93790800	5.65499300
H	-1.78708300	6.59350000	6.17415600
C	-4.76291800	0.37705500	6.92460600
H	-5.57607300	-0.34663800	7.00161200
H	-3.92445700	-0.05758800	6.37813100
H	-4.43712100	0.65553300	7.92935600
C	-7.03266300	-4.51699600	4.09024900
H	-7.78272900	-5.14719400	3.60937600
H	-6.20278200	-4.33371500	3.40586800
H	-6.66171500	-5.02179700	4.98511200
C	-9.55146300	-6.28870200	-1.23920800
H	-10.35745000	-6.47599700	-1.95045800
H	-8.78507100	-5.66162300	-1.69747900
H	-9.11060700	-7.24256200	-0.94099900
C	-11.92518100	-3.68012500	-6.15614800
H	-11.40296600	-4.50368100	-6.64845400
H	-12.67937000	-3.28062200	-6.83666400
H	-11.20741500	-2.90155200	-5.89266900
*t*Bu			
C	20.13371000	-0.65826700	0.05462100
C	19.66088100	-1.68891300	-0.70083500
C	18.28523400	-1.55494300	-1.10667300
C	17.73816200	-0.37815100	-0.62685400
S	18.91701300	0.53812200	0.31094500
C	16.40643300	0.23612500	-0.74834400
C	15.97845200	1.15436200	-1.67249700
C	14.63494000	1.62628700	-1.50026200
C	14.04840200	1.05276300	-0.38144700
S	15.14832700	-0.05368800	0.42321300
C	12.72113100	1.21954600	0.22864200
C	12.25747600	2.24749500	1.01133200

C	10.92038700	2.09375900	1.50677700
C	10.37446600	0.88653300	1.09446800
S	11.50217400	-0.02210900	0.10354300
C	9.06747100	0.26002400	1.34428100
C	8.63446800	-0.41097900	2.46069300
C	7.31279400	-0.96165700	2.37843800
C	6.74806100	-0.71190100	1.13597200
S	7.83898700	0.19917600	0.10691600
C	5.44351400	-1.06896400	0.55896900
C	5.02773300	-2.27310900	0.04770500
C	3.69851600	-2.29064800	-0.49099400
C	3.11035100	-1.03724800	-0.40085400
S	4.18863200	0.12494500	0.35100600
C	1.79149400	-0.53571400	-0.81524700
C	1.35338400	-0.19552400	-2.07090400
C	0.01795200	0.32290800	-2.14110300
C	-0.55298100	0.39522200	-0.87864000
S	0.54909500	-0.18435600	0.35782600
C	-1.86811600	0.86264700	-0.41566000
C	-2.31051200	2.14951500	-0.23633300
C	-3.63667700	2.28004400	0.29439100
C	-4.19438600	1.03492400	0.54632300
S	-3.09339200	-0.26118200	0.11305500
C	-5.49401800	0.63476800	1.10524500
C	-5.90712300	0.64696700	2.41414400
C	-7.23082900	0.14770500	2.64975800
C	-7.81718700	-0.28009700	1.46720800
S	-6.74412100	-0.04762200	0.09812900
C	-9.13036700	-0.87552100	1.17936100
C	-9.55736500	-2.16368200	1.38569000
C	-10.89298800	-2.45039300	0.94941600
C	-11.47531800	-1.33311200	0.36779200
S	-10.38343400	0.04058500	0.38313900
C	-12.79808900	-1.11463500	-0.23580900
C	-13.26272400	-1.51148500	-1.46510800
C	-14.59939500	-1.10010100	-1.78320500
C	-15.14228600	-0.33985900	-0.75718200
S	-14.01366800	-0.16118500	0.57439600
C	-16.44571000	0.32304200	-0.60681700
C	-16.88951600	1.49423900	-1.16710700
C	-18.20553300	1.89689200	-0.75602200
C	-18.73334900	1.00402800	0.14124200
S	-17.66131300	-0.31210500	0.49042400
H	21.12483600	-0.53569800	0.46934800
H	20.27751700	-2.53785300	-0.97128700
H	16.62751000	1.47485600	-2.47658900
H	12.88239200	3.10102400	1.23618200
H	9.26918300	-0.51301100	3.33021600

H	5.68052200	-3.13518900	0.05235000
H	1.99498400	-0.30865700	-2.93413100
H	-1.67812200	2.99375600	-0.47459600
H	-5.25628000	1.00445700	3.20020200
H	-8.90849300	-2.89955700	1.84049100
H	-12.64046400	-2.08671900	-2.13681200
H	-16.27028400	2.06842000	-1.84629500
H	-19.69778700	1.03927800	0.62673000
C	13.98915000	2.64881200	-2.45366100
C	14.82427400	2.79476600	-3.74466400
H	15.81913500	3.20663200	-3.54876100
H	14.94457700	1.83625600	-4.26013700
H	14.31780400	3.48142400	-4.43029200
C	17.63899600	-2.65408900	-1.96500500
C	17.67998300	-3.98580900	-1.17607100
H	17.24901100	-4.79413100	-1.77729500
H	18.70114100	-4.27534400	-0.91104300
H	17.10299100	-3.90781400	-0.24916900
C	10.23999700	3.15156400	2.39602700
C	11.05757200	4.46132000	2.41296800
H	12.04598400	4.32421500	2.86270800
H	11.19093200	4.87027900	1.40613500
H	10.52992000	5.21158200	3.01014900
C	6.66467700	-1.73354000	3.54307000
C	7.48428800	-1.55866600	4.84026100
H	8.48634600	-1.99155800	4.75815000
H	7.58645600	-0.50441300	5.11778300
H	6.97575600	-2.07156300	5.66268300
C	3.06511400	-3.55953500	-1.09219300
C	3.91322500	-4.80587800	-0.75771500
H	4.90833900	-4.76226700	-1.21143700
H	4.03248600	-4.93820500	0.32265300
H	3.41673600	-5.69912600	-1.14947000
C	-0.63614100	0.74817900	-3.46898200
C	0.19424100	0.25070000	-4.67213000
H	1.18833100	0.70763500	-4.70462300
H	0.31571400	-0.83739000	-4.65981200
H	-0.31650600	0.51844900	-5.60241600
C	-4.29069500	3.65031700	0.55242300
C	-3.50407700	4.77383900	-0.15798000
H	-2.49209100	4.88750200	0.24301000
H	-3.42961300	4.59790900	-1.23611600
H	-4.01998900	5.72781100	-0.01039500
C	-7.85918100	0.10184000	4.05534300
C	-7.02248400	0.92842400	5.05618700
H	-6.02125800	0.51077500	5.20168600
H	-6.91799300	1.96998200	4.73517600
H	-7.51900900	0.92878500	6.03160300

C	-11.53800000	-3.83897700	1.11818500
C	-10.69726300	-4.72220700	2.06602000
H	-9.70444200	-4.93714400	1.65836000
H	-10.57264500	-4.25874000	3.05015800
H	-11.20268800	-5.68207700	2.21158200
C	-15.28563400	-1.47223700	-3.11125600
C	-14.48061100	-2.55793600	-3.85795100
H	-13.49142000	-2.20193200	-4.16250600
H	-14.35038300	-3.45817400	-3.24852900
H	-15.01649100	-2.84618900	-4.76772900
C	-18.89053500	3.16692500	-1.25865500
C	-19.02459000	3.10405000	-2.79768100
H	-18.04830600	3.01917300	-3.28511300
H	-19.62831600	2.24400400	-3.10458800
H	-19.50753800	4.01204800	-3.17593900
C	18.45804400	-2.80997000	-3.26995200
H	18.02973300	-3.60544200	-3.88987100
H	18.44570600	-1.88280500	-3.85182900
H	19.50277600	-3.06768800	-3.07374500
C	13.92700400	4.03448900	-1.76790100
H	13.53277700	4.78412600	-2.46379800
H	13.27684200	4.01548600	-0.89056900
H	14.92255600	4.35971300	-1.44844600
C	10.14507100	2.63175700	3.84998300
H	9.72245000	3.40558000	4.50124500
H	9.50405300	1.75024800	3.91553800
H	11.13370400	2.36579900	4.23830800
C	6.61819000	-3.24419000	3.21190700
H	6.22442100	-3.80592600	4.06684100
H	5.97370600	-3.44437600	2.35355600
H	7.61832100	-3.62880300	2.98651500
C	2.99484300	-3.43181900	-2.63240100
H	2.61016000	-4.35929600	-3.07210200
H	2.33265600	-2.61748000	-2.93335000
H	3.98608500	-3.24212000	-3.05727100
C	-0.70861100	2.29173700	-3.54731700
H	-1.11118300	2.60180000	-4.51855600
H	-1.35710600	2.69969000	-2.76926600
H	0.28431100	2.73914000	-3.43408100
C	-4.28704800	3.95324800	2.06964300
H	-4.68565700	4.95728500	2.25587800
H	-4.90476000	3.23957300	2.61900500
H	-3.27148300	3.91163100	2.47687300
C	-7.90114900	-1.35877000	4.56422100
H	-8.28023700	-1.39010800	5.59217900
H	-8.55543300	-1.97674800	3.94588600
H	-6.90195100	-1.80661100	4.55872000
C	-11.61537300	-4.55334500	-0.25178500

H	-12.00718200	-5.56944400	-0.12677500
H	-12.27514800	-4.02173400	-0.94036700
H	-10.62557200	-4.62681300	-0.71413100
C	-15.36797800	-0.22850500	-4.02779300
H	-15.78899400	-0.50360200	-5.00176800
H	-16.00478600	0.54470300	-3.59337300
H	-14.37508400	0.20068100	-4.19821900
C	-18.03616000	4.39597700	-0.86997700
H	-18.50688700	5.31759900	-1.23031300
H	-17.92863900	4.46999700	0.21678500
H	-17.03234500	4.34343600	-1.30261800
C	-20.29339400	3.32602000	-0.64581700
H	-20.24894200	3.39908700	0.44576300
H	-20.76453900	4.24022200	-1.02070400
H	-20.94416600	2.48511900	-0.90743300
C	-16.70559700	-2.02928800	-2.86707500
H	-16.67547500	-2.90988600	-2.21721100
H	-17.36249800	-1.29068600	-2.40537800
H	-17.15534700	-2.32817200	-3.82056600
C	-12.95561400	-3.72545600	1.72168900
H	-12.93147500	-3.21319300	2.68898500
H	-13.63769000	-3.18071200	1.06735400
H	-13.37201600	-4.72637500	1.88029800
C	-9.28859700	0.68720000	4.04740600
H	-9.28785600	1.72076700	3.68639100
H	-9.96604900	0.10938100	3.41706400
H	-9.69528800	0.68520000	5.06484500
C	-5.74107500	3.69043800	0.02248200
H	-5.77552900	3.46667900	-1.04869500
H	-6.38652300	2.97778100	0.53775400
H	-6.16169400	4.69101400	0.17174800
C	-2.05721000	0.15789400	-3.60621100
H	-2.03634700	-0.93520100	-3.54877600
H	-2.73100100	0.52426000	-2.83031800
H	-2.48146800	0.43700200	-4.57698800
C	1.64651000	-3.79493000	-0.52842000
H	1.66588300	-3.87637900	0.56318800
H	0.96006400	-2.99020700	-0.79567100
H	1.23806800	-4.72933400	-0.92931000
C	5.23414000	-1.22203300	3.82314900
H	5.23774200	-0.15193600	4.05454100
H	4.56714300	-1.38217300	2.97482100
H	4.81287200	-1.75221700	4.68454400
C	8.82534000	3.49021000	1.87614000
H	8.86228700	3.85292700	0.84373900
H	8.15881100	2.62713000	1.90744100
H	8.38134600	4.27794800	2.49492500
C	12.56593100	2.21311000	-2.86613100

H	12.57894900	1.22906800	-3.34581600
H	11.88770300	2.16378800	-2.01315400
H	12.15153400	2.93205400	-3.58157300
C	16.17665500	-2.37834000	-2.35679800
H	15.52397600	-2.30548700	-1.48401100
H	16.07374100	-1.45609400	-2.93301900
H	15.81166300	-3.20477100	-2.97632000
